# The tobacco genome sequence and its comparison with those of tomato and potato

**DOI:** 10.1038/ncomms4833

**Published:** 2014-05-08

**Authors:** Nicolas Sierro, James N.D. Battey, Sonia Ouadi, Nicolas Bakaher, Lucien Bovet, Adrian Willig, Simon Goepfert, Manuel C. Peitsch, Nikolai V. Ivanov

**Affiliations:** 1 Philip Morris International R&D, Philip Morris Products S.A., 2000 Neuchatel, Switzerland; 2 Present address: 25b Quai Charles-Page, CH-1205 Genève, Switzerland

## Abstract

The allotetraploid plant *Nicotiana tabacum* (common tobacco) is a major crop species and a model organism, for which only very fragmented genomic sequences are currently available. Here we report high-quality draft genomes for three main tobacco varieties. These genomes show both the low divergence of tobacco from its ancestors and microsynteny with other Solanaceae species. We identify over 90,000 gene models and determine the ancestral origin of tobacco mosaic virus and potyvirus disease resistance in tobacco. We anticipate that the draft genomes will strengthen the use of *N. tabacum* as a versatile model organism for functional genomics and biotechnology applications.

## Abstract (web summary)

Common tobacco (*Nicotiana tabacum*) is a widely cultivated and economically important non-food crop. Here, the authors report the draft genome sequences for three of the most common tobacco varieties and provide insights into the evolution of tobacco through a comparative analysis with closely related species.

## Graphical abstract

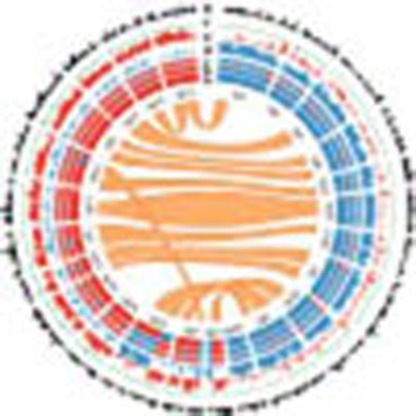

Common tobacco (*Nicotiana tabacum*) is one of the most widely cultivated non-food crops worldwide and is grown in ~120 countries^1^. It belongs to the Nicotiana genus, which is named after Jean Nicot de Villemain who, in 1560, became the first person to import these plants from the Americas to Europe. The term Nicotiana was originally used by Adam Lonitzer to describe tobacco plants in 1630 (ref. 2) and in 1788 by Carl von Linné (Linnaeus) to designate the entire genus^3^. Over 75 naturally occurring *Nicotiana* species, including 49 native to America and 25 native to Australia^4^, have been classified by Goodspeed^5^ and Knapp^6^. Most commercial tobaccos cultivated today belong to the species *Nicotiana tabacum* L., for which >1,600 *N. tabacum* cultivated varieties (cultivars) are listed in the National Plant Germplasm System^7^. The three most commonly used tobacco types are Flue-Cured (or Virginia), Burley and Oriental, which are traditionally grown and harvested under different agricultural practices^8^.

Tobacco is a model plant organism for studying fundamental biological processes^9^, and is the source of the BY-2 plant cell line, which is a key tool for plant molecular research^10^. It is also used as a model for plant disease susceptibility, which it shares with other Solanaceae plants including potato, tomato and pepper. Diseases affecting tobacco include the tobacco mosaic virus (TMV), the tobacco vein mottling virus (TVMV), the tobacco etch virus (TEV), and the potato virus Y (PVY); the TN90 variety of tobacco, which we sequenced here, is notable in that is has been bred to resist these viral infections.

Considerable interest has centred on understanding the origin, organization and evolution of the *N. tabacum* genome. Tobacco stands out as a complex allotetraploid with a large 4.5 Gb genome with significant proportion (>70%) of repeats^11^,^12^. As a species, *N. tabacum* (2*n*=4*x*=48) evolved through the interspecific hybridization of the ancestors of *Nicotiana sylvestris* (2*n*=24, maternal donor) and *Nicotiana tomentosiformis* (2*n*=24, paternal donor) about 200,000 years ago^13^. Because of its complexity and importance, the tobacco genome is a target for the SOL-100 sequencing project^14^, which aims to decipher the genomes of the most important Solanaceae species. The genome sequences of modern varieties of ancestral species were recently reported^15^, and limited evidence suggests that *Nicotiana otophora* is an alternative paternal donor^16^,^17^. In this report, however, we demonstrate that this is unlikely because of the higher sequence identity of the *N. tabacum* T-genome with that of *N. tomentosiformis*. We show the chromosomal rearrangements between the ancestral and tobacco chromosomes, and provide an explanation for an apparent genome reduction following the hybridization. In addition, we present a genomic comparison of tobacco to two other solanaceous species, tomato and potato. Significant chromosomal reshuffling is clearly observed for all chromosomes despite the conservation of their overall count, confirming previous reports^18^.

Tobacco’s rich metabolism (involving >4,000 chemical components) and exceptional ability to express proteins (>40% of its dry weight) have prompted numerous initiatives to harness its potential for the production of biologically active substances^19^. Here, we describe the major alkaloid biosynthesis pathway in *Nicotiana* species, as well as glutamate/aspartate pathways in the three main tobacco types.

In this work, we sequence the genomes of key representatives of the three major types of tobacco and combine them with genetic and physical maps of tobacco^20^,^21^. We verify genome assembly accuracy by mapping transcriptomics and Exon Array^22^ data of corresponding varieties, we assess the consistency of assemblies and published physical and genetic maps, and we compare *N. tabacum* S- and T-genomes with those of *N. sylvestris* and *N. tomentosiformis*.

## Results

### Sequencing and assembly

Genome assembly of polyploid species, such as coffee (*Coffea arabica*), potato (*Solanum tuberosum*) and wheat (*Triticum aestivum*) is challenging. Even the assembly of the relatively small *Brassica napus* genome (1.2 Gb), which has well-annotated ancestral reference sequences, is still ongoing^23^. We sequenced the genomes of three inbred varieties of the allotetraploid *N. tabacum*, K326 (Flue-cured), TN90 (Burley) and Basma Xanthi (BX, Oriental), using a whole-genome shotgun sequencing approach with 100 bp Illumina HiSeq-2000 paired-end and mate-pair reads.

The size of the tobacco genome has been estimated to be 4.46 Gb by flow cytometry using the fluorochrome propidium iodide^24^ and 5.06 Gb by Feulgen microdensitometry^13^. Our estimations based on 17-mer depth distributions of raw sequencing reads^25^,^26^ were 4.41 Gb for *N. tabacum* TN90, 4.60 Gb for *N. tabacum* K326 and 4.57 Gb for *N. tabacum* BX. These represent a reduction of 4–8% of the tobacco genome compared with the sum of the ancestral *N. sylvestris* (2.59 Gb) and *N. tomentosiformis* (2.22 Gb), which is consistent with the previously published downsizing of 3.7% (ref. 13).

The genomes were assembled using SOAPdenovo 1.05 (ref. 27) with a k-mer of 63 by creating contigs from the variety-specific paired-end reads, scaffolding them with *Nicotiana* mate-pair libraries, closing the scaffolding gaps using variety-specific paired-end reads and filtering gap closing artifacts. The resulting final assemblies, described in Table 1, each amount to 3.7 Gb, representing a coverage of >80% of the tobacco genome. The remaining ~20% is likely to consist of repetitive regions that could not be resolved using the short read *de novo* shotgun approach. This represents a great improvement from the previous publicly available tobacco genome assembly ( ftp://ftp.solgenomics.net/tobacco_genome/assembly/), which contains 294,750 sequences (8.5% of the tobacco genome). We also sequenced libraries from *N. otophora* to investigate its contribution to *N. tabacum*.

We evaluated the quality of the scaffolding by mapping the assembly scaffolds to long BAC sequences (altogether 1.8 Mb) obtained from *Nicotiana tabacum* Hicks Broadleaf. We did not observe any sequence inversions due to scaffolding, and identified only three cases where a contig was not incorporated in a scaffold and remained as singleton (Supplementary Data 1). We also identified 13 cases of insertions or deletions. Altogether this represents 8.6 structural differences per megabase, if we assume that the Hicks Broadleaf sequence is the same as the TN90, K326 and BX sequence for the selected BACs.

**Figure 1:**
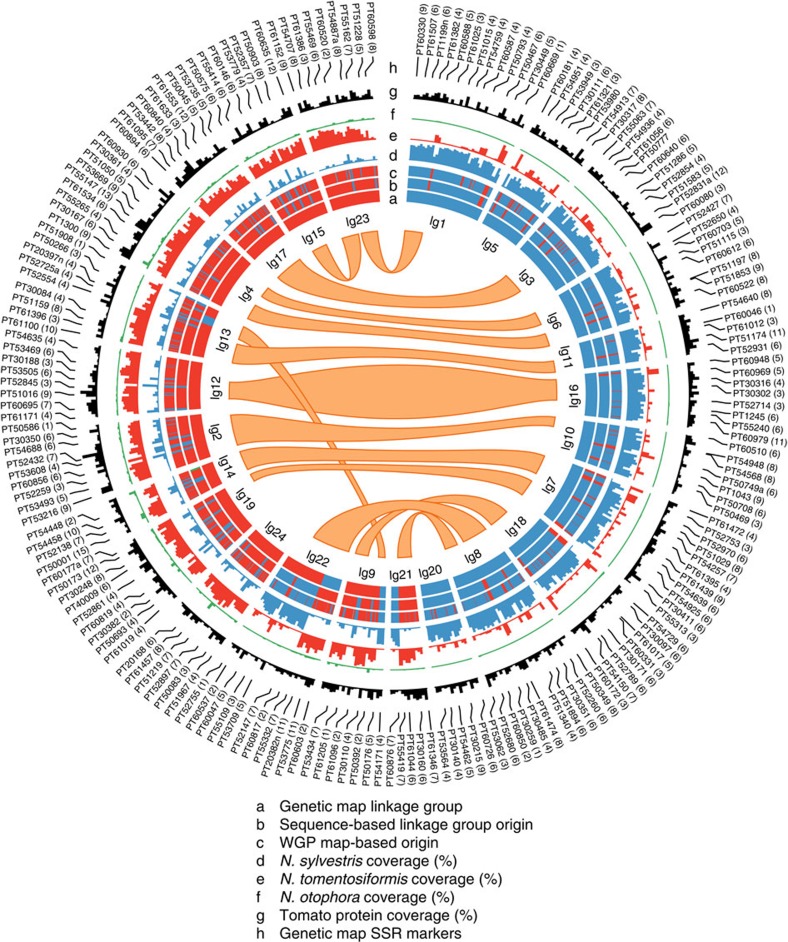
Tobacco genome. Blue and red represent features of S and T origins in the *Nicotiana tabacum* TN90 genome, respectively. Track a indicates the origin of the linkage group previously determined by SSR amplification. Track b shows the assignment of the linkage group origin based on sequence identity with *N. sylvestris*, *N. tomentosiformis* and *N. otophora*. Track c shows the origin of the WGP physical map contig used for super-scaffolding. The blue, red and green histograms (tracks d, e and f, respectively) indicate the percentage of each 2 Mb region covered by an *N. sylvestris*, *N. tomentosiformis* or *N. otophora* sequence of at least 1,000 bp and 98% identity. Track g shows the density of coding regions identified by mapping of reference tomato proteins. Track h gives the position of SSR markers mapped to the genome sequences. The centre links regions of the *N. tabacum* genome based on sequence homology, as determined by reference tomato proteins mapping at two locations.

The sequence-based Whole Genome Profiling (WGP) physical map was used to super-scaffold each genome assembly, and simple sequence repeats (SSRs) were mapped to super-scaffolded assemblies to anchor them to the 24 tobacco linkage groups. This resulted in 84% of the *de novo* assembly of TN90 being anchored to the WGP physical map (83 and 84% for K326 and BX, respectively), and 19% to the genetic map (17 and 16% for K326 and BX, respectively). Figure 1 shows the composition of the *N. tabacum* TN90 genome anchored to the linkage groups of the tobacco genetic map.

The three methods for assigning an ancestral origin to the linkage groups, SSR amplification in *N. sylvestris* and *N. tomentosiformis*^20^, sequence identity with donor species and using the WGP physical map^21^, are concordant. The latter two confirmed the previously reported colour inversion in linkage group 22 (ref. 21), and the central part of Fig. 1 clearly shows the correspondence between regions of different ancestral origins within regions of the *N. tabacum* genome linked by sequence homology.

By mapping SSR markers to the sequenced genomes of *N. tabacum* K326, TN90 and BX, we were able to predict SSR length differences between the three varieties. When comparing TN90 to K326, 57% of the predictions obtained exactly matched experimental measurements, and 10% had only 2-bp differences. The larger differences observed between experimental and *in silico* SSR measurements are likely to be caused by difficulties in resolving the SSR using short read assembly.

We used sequence identity to assign an ancestral origin to each 2-Mb region. The only region for which *N. otophora* appears as the most likely ancestor is at the end of linkage group 14 (Supplementary Fig. 1), indicating that if it contributed to the *N. tabacum* genome, then only marginally. This observation favours the hypothesis that the predominant paternal donor was *N. tomentosiformis*^28^.

We evaluated the completeness of our assemblies by mapping reference gene sequences to each genome using BLAT^29^ (Supplementary Table 1). For this we used NCBI and SGN tobacco Unigene constructs, as well as the SGN tobacco transcriptome^30^. We also used the coding sequences of tomato (ITAG v2.3) and potato (PGSC v3.4). We mapped 82–86% of the tobacco transcriptome; the remainder is likely to consist of genes spanning different genome sequences that have not yet been scaffolded. Between 50–59% of the Unigene constructs from NCBI and SGN could be mapped, presumably reflecting sequence diversity in the Unigene set^31^. Approximately 60% of the tomato and potato coding sequences could be mapped to the genomes, which compares favourably with the rate of transcript mapping to the *Nicotiana benthamiana* genome^31^ (56.5 and 58.5% for tomato and potato, respectively). In 92.8% of the cases, the best possible alignment of a tomato protein overlaps with a tobacco gene model from our TN90 transcriptome (nine tissues). The genetic and physical map anchoring results as well as alignment to the *N. sylvestris* and *N. tomentosiformis* genomes confirmed the quality of the three *N. tabacum* genome assemblies.

### Synteny with other Solanaceae

**Figure 2:**
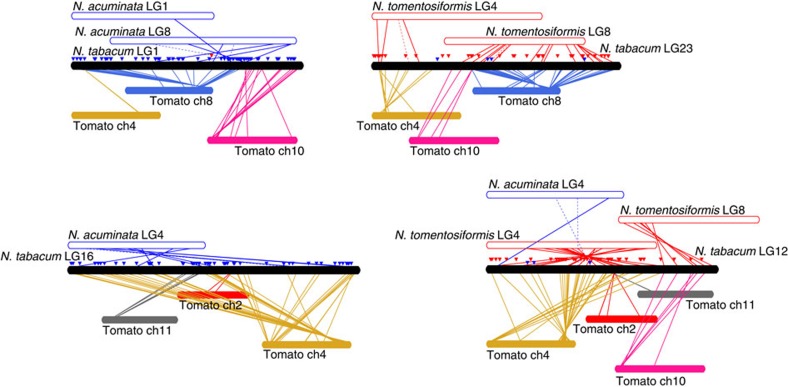
Synteny between *Nicotiana tabacum*, *Nicotiana tomentosiformis* and *Nicotiana acuminata* genetic linkage groups and tomato chromosomes. Links between tomato and tobacco are based on the mapping of tomato proteins to linkage group-anchored tobacco sequences. Note that not all regions identified by this method should necessarily be considered as syntenic. Links between tobacco and *N. tomentosiformis* (red) and *N. acuminata* (blue) are based on shared SSR (full lines) and COSII (dotted lines) markers. Triangles indicate the amplification of the corresponding SSR in *N. tomentosiformis* (red) or *N. sylvestris* (blue).

The linkage groups from the genetic maps of both *N. tomentosiformis* and *N. acuminata*^18^, and the results of SSR amplification in *N. tomentosiformis* and *N. sylvestris*^20^, were used to show rearrangements that are found in tobacco (Supplementary Data 2 and 3). Figure 2 illustrates one such rearrangement, where part of linkage groups 4 and 8 of *N. tomentosiformis* are fused to give *N. tabacum* linkage groups 12 and 23, whereas *N. acuminata* linkage groups 4 and 8 did not fuse, and gave *N. tabacum* linkage groups 16 and 1, respectively.

The synteny between the genomes of *N. tabacum* TN90, K326 and BX and those of tomato and potato was evaluated at the protein level by mapping tomato and potato proteins to tobacco sequences anchored to the linkage groups of the genetic map^20^ to detect homologous genes (Supplementary Data 2 and 3), and by detecting further homologous DNA blocks in genomes masked for repeats (Supplementary Data 4–7). Similar results were obtained using MCScanX^32^, which is a toolkit specifically designed for the detection and analysis of gene synteny and collinearity (Supplementary Data 8 and 9). Not all the regions identified by either of these methods should be considered as truly syntenic, as some of them rely only on a limited number of anchors. Likewise, additional syntenic regions are likely to exist, which cannot be detected by the methods used here. Nevertheless, these approaches confirm results reported earlier based on COSII (ref. 18) and SSR markers^20^.

### Repetitive elements in the genomes

The repeat content of the *N. tabacum* K326, TN90 and BX genomes is summarized in Supplementary Table 2. Between 72 and 79% of the sequenced genomes are reported as repeat elements by RepeatMasker. This estimation is lower than that reported for barley (84%) and close to the original estimate (~80%) by Zimmerman^11^.

Based on the amount of the sequenced genome covered by repeats, we evaluated the DNA portion of the tobacco genome containing non-repeat coding regions to about 1 Gb. This is equivalent to the sum of the same DNA portion from the descendants of both ancestral genomes. The observed 4–8% reduction in genome size is thus likely to have occurred in the repetitive region of the genome. The sum of the genome sizes from the descendants of both ancestors is of 5.04 Gb, *N. sylvestris* accounting for 53% of it, and *N. tomentosiformis* for 47%. The tobacco scaffolds to which an origin could be assigned show 55–57% of S origin and 43–45% of T origin. The genome reduction thus is likely to have been more important in the T part of the genome than in the S part, which corresponds to what was reported by Renny-Byfield *et al.*^12^

The reported assemblies of three tobacco varieties cover >80% of the genome, which is comparable to that for smaller diploid genomes (76–90%)^25^,^33^ and for the smaller (3 Gb) allotetraploid *N. benthamiana* (81–87%)^30^,^34^. They represent some of the largest assembled plant genomes together with barley (5.1 Gb)^35^, Norway spruce (20 Gb)^36^ and the partial wheat genome (17 Gb)^37^. Contrary to other allotetraploid genomes, for which ancestral information is either not available (*N. benthamiana*) or limited (wheat), the ancestral origin of the tobacco sequences was identified and confirms previously reported assignments based on genetic markers^20^ and the physical map^21^.

### Tobacco root and leaf transcriptome analysis

For each variety, three biological replicates were obtained from roots and leaves, which are two metabolically highly active tobacco tissues. In addition, nine tissues were sampled for TN90, so as to get good coverage of the gene regions. For each RNA-Seq sample, 86.5–94.7% of reads were mapped to the genome of the corresponding variety (Supplementary Table 3). Using RNA-seq data from two tissue types, we generated gene models for each of the three varieties, the gene number estimate being 81,000 for TN90 (Supplementary Table 4). Using data from nine tissue samples increases this estimate by 14.7% to >93,000 genes. Of the 134,694–188,510 transcripts, approximately half this number of unique open reading frames can be found (Supplementary Table 4). These numbers do not represent the whole transcriptome of tobacco, as we sampled RNA from only nine tissues, and hence will be missing transcripts not expressed in any of them. For the root and leaf transcriptomes, gene ontology (GO) terms could be assigned by InterProScan^38^ to ~40,000 proteins (from ~28,000 genes) (Supplementary Table 5); for the nine tissue samples, this number increases to >50,000, although there is notable increase in the number of unique GO terms assigned. A recent analysis of 454 *N. sylvestris*, *N. tomentosiformis* and *N. tabacum* next-generation sequencing transcriptomes showed neither differences in gene expression nor the creation of a new function for homoeologous genes between *N. tabacum* S- and T genomes^30^. Similarly, we found no new genes or new functionality of existing genes in the three varieties.

We analysed the GO term enrichment for differentially expressed genes in each tissue. Photosynthesis- and biosynthetic-related genes were highly expressed and enriched in leaf tissue, as were genes involved in oxidation–reduction processes (Supplementary Fig. 2). Root tissue has a very distinctive profile of upregulated genes (Supplementary Fig. 3). Besides lipid transport and regulatory gene overexpression, lignin and cell wall metabolism genes were prominent. Moreover, defence and oxidative stress response genes were heavily upregulated, which may be related to cell death processes apparent from the expression profile. The overexpression of ‘pollination’ genes reflects the electronic annotation of several upregulated proteins within the PFAM domain PF00954, the so-called ‘S locus glycoprotein-like’ domain. While this protein family is best known for being involved in the pollination process, some members are involved in defence response regulation^39^. It is thus more likely that the proteins identified here are involved in defence, rather than in pollination.

**Figure 3:**
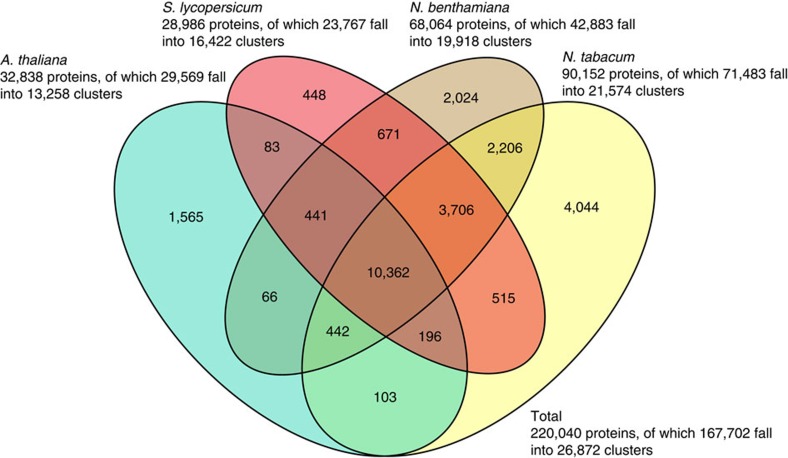
Protein clusters shared between tobacco and other increasingly more distant species phylogenetically. *Nicotiana benthamiana* is a representative of the Nicotiana genus, *Solanum lycopersicum* (tomato) is a representative of the Solanum genus and *Arabidopsis thaliana* is a representative of the eudicotyledons.

We used OrthoMCL to analyse the functional overlap between tobacco and three other, increasingly divergent plant species: *N. benthamiana* as a further representative of the Nicotiana genus, *Solanum lycopersicum* (tomato) as a further species in the Solanaceae family and *Arabidopsis thaliana*, another species in the eudicotyledons (Fig. 3). The bulk of the protein clusters are shared between all dicotyledons (10,362), we observed 2,024 and 4,044 clusters specific to *N. benthamiana* and *N. tabacum*, respectively, and 2,206 Nicotiana-specific clusters shared between both species. We also observed 3,706 clusters shared between all Solanaceae but not with Arabidopsis.

### Classical tobacco pathways

We extracted the sequences of biochemical pathway enzymes and observed copy numbers and expression in roots and leaves under different growth conditions (Supplementary Note 1). No major differences in gene expression were observed, but one new putrescine N-methyltransferase gene was identified in the alkaloid pathway in addition to the four already reported^40^. Furthermore, the two quinolinate phosphoribosyltransferase (QPT) genes of S origin were not found in *N. tabacum* K326 (Supplementary Table 6 and Supplementary Table 7). Most described alkaloid genes are expressed in roots where most alkaloids are synthesized; however, some transcripts from *AO*, *QPT*, *QS* and *MPO* genes were also detected in leaf, suggesting that they have different functions (Supplementary Fig. 4 and Supplementary Note 1).

In addition, we have mapped the putative steroidal alkaloid biosynthesis genes from Nicotiana genomes to the syntenic regions recently discovered on chromosomes 7 and 12 of *S. lycopersicum*^41^. In *N. tabacum* we identified two copies of each region, corresponding to their ancestral origin, indicating that these regions are conserved within Nicotiana species (Supplementary Fig. 5).

For Burley tobacco, which is known for its high nitrogen requirement, nitrogen assimilation is stronger than for Flue-cured tobacco (Supplementary Fig. 6). We therefore compared the number and expression levels of genes related with the glutamate/aspartate pathway (Supplementary Table 8 and Supplementary Note 2). With the exception of one AAT5 isoform missing in K326 but present in BX and TN90, we observed neither CNVs nor major transcriptomic variations, thereby suggesting that the nitrogen assimilation at the level of the glutamate/aspartate pathway is close between Burley tobacco and Flue-cured tobacco. However, it is not enough to conclude that the efficiency of ammonium assimilation is similar in both tobaccos, other downstream gene products from root to leaf being involved in the pathway as well as adaptations to environmental conditions. The analyses of the complete set of genes related to amino-acid assimilation will certainly help to understand the effect of human artificial selection on the high nitrogen requirement of Burley tobacco.

### Disease resistance

TMV resistance was introduced in tobacco in the 1930s as a single dominant locus from an interspecific hybrid with *Nicotiana glutinosa*^42^,^43^,^44^. This locus was shown to harbour the *N* gene (NGU15605) encoding a (TIR)-NBS-LRR protein^45^ that triggers a hypersensitive response following recognition of the viral helicase^46^.

Among the three varieties sequenced here, only TN90 is TMV-resistant. TN90 has inherited the *N* gene from the variety Burley 21, which in turn inherited it from the variety Kentucky 56, which in turn inherited it from *N. glutinosa* hybrids^47^. Indeed, a search for the *N. glutinosa N* gene sequence in the draft assemblies showed weak identity in K326 and BX genomes (~90% identity on <35% of *N. glutinosa* genomic DNA), whereas a nearly perfect match was found for TN90 (99.9% identity over 7,158 bp).

PVY is a potyvirus, which is transmitted by aphids, and has a broad host range including potato and tomato. *N. tabacum* is naturally susceptible to PVY and other potyviruses such as TVMV and TEV; interestingly, the ancestors *N. tomentosiformis* and *N. sylvestris* are resistant and susceptible, respectively, to PVY. Most modern tobacco varieties bred for PVY resistance carry alleles of the Va locus. The recessive *va* allele, which was first obtained by deletion^48^ in the line TI 1406 (Virgin A Mutant) (Supplementary Note 3), confers resistance by preventing virus cell-to-cell movement in a similar way to the recessive resistances of pepper, potato and tomato, suggesting the involvement of a eukaryotic initiation factor^49^. The characterization of the PVY resistance gene using a recombinant inbred line population was recently presented by Julio *et al*.^50^ The eukaryotic translation initiation factor eIF4E was shown to be strongly expressed in susceptible plants, but not in resistant plants.

We carried out a sequence comparison of the genome of TN90, which is resistant to the potyviruses TVMV, TEV and PVY, and the genomes of K326 and BX, which are susceptible. We identified *eIF4E1*, *eIF4E2* and *eIF(iso)4E* genes in the TN90, K326 and BX genome assemblies by first mapping corresponding tomato genes to *N. sylvestris* and *N. tomentosiformis* genomes, then mapping the respective genes to the tobacco genomes. One copy of each gene was found in the *N. sylvestris* genome, whereas two copies of *eIF4E1* and one copy of the other genes were found in *N. tomentosiformis*. With the exception of the *N. sylvestris eIF4E1* gene in TN90, all identified *N. sylvestris* and *N. tomentosiformis* genes were found in TN90, K326 and BX genomes. Gene-specific PCR verification (Supplementary Fig. 7) showed that the absence of the *N. sylvestris eIF4E1* in TN90 is not an artefact of genome assembly, but rather that it is missing from this variety. This confirms the observation that TVMV, TEV and PVY resistance in TN90 is caused by genomic deletion of the S-form *eIF4E1* locus^50^. This is in line with the pattern of resistance observed in tobacco ancestors, suggesting that the dominant sensitivity of *N. tabacum* to potyviruses via the eIF4E1 mechanism was conferred by *N. sylvestris*. The fact that both S and T copies of *N. tabacum eIF4E1* are expressed concomitantly (Supplementary Table 9) allows us to speculate that either the interaction between the *N. tomentosiformis* eIF4E1 copies and viral proteins are compromised, or that the viral–host protein complex cannot provide the cell-to-cell movement required for systemic infection.

## Discussion

Next-generation sequencing data were used to construct the genomes of three tobacco varieties, representing the three main market classes of commercially grown tobacco. Assembly accuracy was verified by mapping tobacco transcriptomics data, by assessing the consistency with tobacco physical and genetic maps, and through comparison of *N. tabacum* S- and T genomes to ancestral (*N. sylvestris* and *N. tomentosiformis*) genomes. The assemblies cover >80% of the genome, 75% being annotated as repeats. Sequencing and mapping of *N. otophora* in addition to mapping *N. sylvestris* and *N. tomentosiformis* genome sequences to the tobacco genomes confirmed that *N. sylvestris* and *N. tomentosiformis* are the most likely progenitors of *N. tabacum*. We observed a 4–8% genome reduction in the allotetraploid *N. tabacum* compared with its ancestral species.

Between 81,000 and 94,000 gene models were identified for the three varieties. The alkaloid biosynthesis pathway characteristic of *Nicotiana* species and the glutamate/aspartate pathway did not show marked CNVs or preferential expression of genes from one ancestor within the investigated genes; indeed, only four of 32 identified homoeologous pairs were differentially expressed. This suggests that there was no adaptation affecting these two pathways at the genome or transcriptome level. The comparison of the genomes of the three varieties might shed some light on the effect of human selection, in addition to natural selection, resulting in resistance to TMV and potyviruses. However, more analyses about the evolution of R genes after the formation of allotetraploid will be necessary to understand this phenomenon.

The draft genomes are sufficiently complete to both make and test hypotheses at the biological level, as exemplified by the analysis of virus resistance and alkaloid pathway genes. Together with the genome of *N. otophora*, they represent an important contribution to the SOL-100 genome project. Alongside genomes of the ancestral species *N. sylvestris* and *N. tomentosiformis*, the *N. tabacum* genome illustrates the evolutionary history of a complex allotetraploid. Finally, they strengthen *N. tabacum* as a plant model system, and as a platform for plant molecular farming.

## Methods

### Plant material and nucleic acid isolation

Seeds of *N. tabacum* TN90, K326 and Basma Xanthi (Supplementary Note 4) were sterilized using chlorine gas and grown axenically on MS medium^51^ for 4 months under artificial light (16 h day per 8 h night). DNA extraction was performed on aerial parts of one single plant of each variety using the Qiagen DNAeasy Plant Maxi Kit (Qiagen, Hilden, Germany). RNA extraction was performed using the Qiagen RNAeasy Mini Kit (Qiagen) on roots and leaves of three independent plants. In addition, nine TN90 tissues samples were analysed by the same method (see Supplementary Table 3 for details), whereby for 8 tissues biological triplicates were used and for one tissue biological duplicates were used. The DNA and RNA quantity and quality was verified using a Bioanalyzer (Agilent Technologies, Santa Clara, CA, USA).

### Genome and root and leaf transcriptome sequencing

Short-insert ‘paired-end’ libraries were prepared using the Illumina TruSeq DNA Sample Preparation Kit version 2 (Illumina, San Diego, CA, USA). Long-insert ‘mate-pair’ libraries were prepared according to the Illumina Mate Pair Library Prep Kit version 2 (Illumina), or using a protocol developed by Fasteris SA (Geneva, Switzerland). In this case, 10 mg of genomic DNA was broken into fragments of ~2–5 kb using the Covaris E220 Focused-ultrasonicators (LGC Genomics, Berlin, Germany) and purified on a 0.7% agarose gel to recover 3 kb and 5 kb fragments. After ends-repair, a Fasteris-designed spacer was ligated and the fragments were circularized. After elimination of non-circular fragments, the DNA was again broken using the Covaris E220 Focused-ultrasonicators to generate fragments of 400 bp that were end-repaired ans ligated with Illumina adapters. RNA-seq libraries were constructed using the Illumina TruSeq RNA Sample prep Kit (Illumina). All libraries were sequenced on an Illumina HiSeq-2000 using version 3 chemistry and flow cells with runs of 2 × 100 bases. Base calling and sample demultiplexing were performed using Illumina HiSeq Control Software and CASAVA pipeline software.

### *De novo* genome assembly

Raw DNA reads (Supplementary Table 10) were preprocessed with FASTX toolkit utilities^52^ by first trimming 3′ bases with qualities lower than 30, and then discarding reads shorter than 50 bases or with <90% of the bases with qualities lower than 30. The paired-end libraries with insert sizes shorter than 200 bases were further preprocessed using FLASH^53^ to merge the paired-end reads into extended single reads. The paired and single reads from the paired-end libraries were then assembled into contigs using SOAPdenovo^27^ with a k-mer of 63. Paired reads from paired-end and mate-pair libraries were used for scaffolding by increasing library size. To improve scaffolding, mate-pair libraries from the closely related *Nicotiana* species *N. sylvestris* and *N. tomentosiformis*^15^ were also used (Supplementary Table 11). SOAPdenovo was instructed to use these libraries only during its scaffolding step, and not during the building of contigs or during gap closing. Gaps resulting from the scaffolding were closed using GapCloser^27^ and all sequences shorter than 200 bases were discarded from the final assemblies. After closing the gaps, singletons were blasted against the scaffolds and removed if matching was higher than 97% to avoid artificial duplication of short sequences. Figure 1 shows details of the *N. tabacum* TN90 genome.

### Synteny with other Solanaceae

The tobacco assemblies were superscaffolded using the sequence-based WGP tobacco physical map. The obtained superscaffolds were then anchored to the tobacco genetic map by mapping of SSR markers. Synteny with the tomato and potato chromosomes (Supplementary Data 2 and 3) was determined by mapping of tomato and potato reference proteins (ITAG2.3 and PGSC_DM_4.03) to the genetic map-anchored superscaffolds using BLAT^29^. The mapping was filtered to retain hits with at least 50% coverage and 80% identity. Supplementary Table 12 lists the number of proteins from each tomato and potato chromosome that are mapped to each linkage group. In addition to using the SSR markers shared between the *N. tabacum*, *N. tomentosiformis* and *N. acuminata* genetic maps, TAIR10 proteins for COSII markers present on two latter maps were also mapped with BLAT and filtered to retain hit with at least 50% coverage and 50% identity. To determine synteny between tomato or potato and tobacco using whole genomes (Supplementary Data 4–7), we masked the genomes using tantan^54^, tandem repeat finder^55^ and RepeatMasker and used nucmer^56^ to detect syntenic DNA blocks. Analysis using MCScanX^32^ was performed using the tomato and potato reference proteins and set of predicted proteins derived from the *N. tabacum* TN90 RNA-seq data (Supplementary Data 8 and 9).

### Estimation of the quality of the assembly

*N. tabacum* Hicks Broadleaf BAC sequences were sequenced by Roche 454 and assembled with newbler. In total 1,864,003 bases were obtained in sequences longer than 80 kb. The TN90, K326 and BX assemblies were mapped to these BACs sequences using blastn and a threshold of 98% identity, and the identified scaffolds remapped to the BAC sequences using LAST^57^,^58^.

### Repeat content estimation

The repeat content of the genome assemblies was estimated using RepeatMasker with the eudicot repeat library available from the Sol Genomics Network, the TIGR Solanaceae repeat library and RepeatScout^59^ libraries created using sequences of at least 200 kb from the draft genome assemblies. Classification of the repeat types was performed using blastn^60^ hits to known repeat elements.

### Transcriptome assembly and quantitative analysis

The transcriptomes were derived by mapping RNA-Seq reads from each variety to the corresponding reference genome. For each variety, three biological replicates were obtained for each tissue. Mapping was performed using the ‘tuxedo’ suite of short read mapping tools (Bowtie v2.0.0 beta^61^, Tophat v2.0.4 (ref. 62) and Cufflinks v2.0.2 (ref. 63). The resulting fragments were used as input for the Trinity software suite^64^ open reading frame finder; the resulting predicted protein sequences were filtered for uniqueness and used for further analysis. GO terms were assigned to the unique subset of predicted protein sequences using InterProScan^38^. Protein clusters were determined using OrthoMCL^65^ with standard parameters. Besides the predicted proteins from the TN90 transcriptome, we used the following sources to obtain proteins from other species: SGN for *N. benthamiana*, ITAG (version 2.3) for *S. lycopersicum* and TAIR (version 10) for *A. thaliana*. All protein sets were filtered for a minimum protein size of 100 amino-acid residues.

For the quantitative analysis, the mapped RNA-seq reads were counted using HTSeq, and DESeq^66^ was used to calculate differences in expression between the two tissues. Genes with a 10-fold upregulation and an associated adjusted *P*-value cutoff of 0.001 were deemed to be significantly differentially regulated. The set of overexpressed genes in leaves and roots was analysed for GO term enrichment. The software tool BiNGO^67^ was used to calculate and visualize the enrichment. For determining enriched terms, the hypergeometric test was used with subsequent Benjamini and Hochberg false discovery rate corrections; a *P*-value threshold of 0.001 was used as a cutoff for inferring enrichment.

### Pathway gene identification

Genes of interest were identified by mapping TAIR10 or UniProt proteins using BLAT^29^ to the genomes of *N. tabacum* ancestors *N. sylvestris* and *N. tomentosiformis*^15^, extracting the identified genomic regions and 5 kb flanking regions, remapping the query protein using Exonerate^68^ and predicting the target gene using SNAP^69^ with the Exonerate hints. The thus-predicted *N. sylvestris* and *N. tomentosiformis* coding DNA sequences were then mapped to *N. tabacum* assemblies, and the *N. tabacum* coding DNA sequences and proteins were extracted using the above method.

### PCR *eIF4* gene family detection

Sequences related to *eIF4E* were identified by mapping tomato homologues as described above. In addition to the previously described PVY test primers^70^, specific primer pairs were designed for each *eIF4E* member (Supplementary Table 13). Genomic DNA of different varieties was isolated as described in the plant section. PCR of genomic DNA was conducted using a 2-ng μl^−1^ template, 0.25 μM of each primer and GoTaq Hot Start polymerase (Promega AG, Switzerland). PCR was performed at 95 °C for 8 min, then 40 cycles of 30 s at 95 °C, 55 °C and 72 °C, sequentially, with a final extension at 5 min at 72 °C. PCR products were separated on a 1.2% agarose gel in 0.5 × TAE and visualized using ethidium bromide.

## 

## Author contributions

N.S., N.V.I. and M.C.P. conceived and designed the study. S.G. and L.B. provided plant material and contributed to the biological interpretation of the results. A.W. extracted nucleic acid material, and designed and performed *eIF4* PCR tests. N.B. and S.G. contributed to the disease resistance section. S.O. prepared the libraries and performed Illumina sequencing. N.S. and J.N.D.B. carried out genome and transcriptome analysis. N.S., J..N.D.B., L.B., S.G. and N.V.I. wrote the manuscript. N.V.I. supervised the study. All authors read and approved the final manuscript.

## Additional information

**Accession codes:** Genome sequence data for *Nicotiana tabacum* TN90 has been deposited in DDBJ/EMBL/GenBank nucleotide core database under the accession code AYMY00000000. Genome and transcriptome sequencing data for *N. tabacum* TN90 have been deposited in GenBank Sequence Read Archive (SRA) under the accession code SRP029183. Genome sequence data for *Nicotiana tabacum* K326 has been deposited in DDBJ/EMBL/GenBank nucleotide core database under the accession code AWOJ00000000. Genome and transcriptome sequencing data for *N. tabacum* K326 have been deposited in GenBank Sequence Read Archive (SRA) under the accession code SRP029184. Genome sequence data for *Nicotiana tabacum* Basma Xanthi has been deposited in DDBJ/EMBL/GenBank nucleotide core database under the accession code AWOK01000000. Genome and transcriptome sequencing data for *N. tabacum* Basma Xanthi have been deposited in GenBank Sequence Read Archive (SRA) under the accession code SRP029185. Genome sequence data for *Nicotiana otophora* has been deposited in DDBJ/EMBL/GenBank nucleotide core database under the accession code AWOL00000000. Genome sequence data for *N. otophora* have been deposited in GenBank Sequence Read Archive (SRA) under the accession code SRP028836.

**How to cite this article:** Sierro, N. *et al.* The tobacco genome sequence and its comparison with those of tomato and potato. *Nat. Commun.* 5:3833 doi: 10.1038/ncomms4833 (2014).

## Supplementary Material

Supplementary Figures, Tables, Notes and References Supplementary Figures 1-7, Supplementary Tables 1-13, Supplementary Notes 1-4 and Supplementary References

Supplementary Data 1 Alignment of assembly scaffolds to *Nicotiana tabacum* Hicks Broadleaf BAC sequences.

Supplementary Data 2 Synteny of the 24 *Nicotiana tabacum* linkage groups with the 12 tomato chromosomes, based on tomato protein mapping.

Supplementary Data 3 Synteny of the 24 *Nicotiana tabacum* linkage groups with the 12 potato chromosomes, based on potato protein mapping.

Supplementary Data 4 Synteny of the 24 *Nicotiana tabacum* linkage groups with the 12 tomato chromosomes, based on whole genome sequences. Syntenic DNA blocks in each plot are positioned on the x axis according to their location in the *Nicotiana tabacum* linkage group, and on the y axis according to their location on the tomato chromosome.

Supplementary Data 5 Synteny of the 24 *Nicotiana tabacum* linkage groups with the 12 tomato chromosomes, based on whole genome sequences. Syntenic DNA blocks in each plot are positioned on the x axis according to their location in the *Nicotiana tabacum* linkage group. Each line on the y axis represents one tomato chromosome.

Supplementary Data 6 Synteny of the 24 *Nicotiana tabacum* linkage groups with the 12 potato chromosomes, based on whole genome sequences. Syntenic DNA blocks in each plot are positioned on the x axis according to their location in the *Nicotiana tabacum* linkage group, and on the y axis according to their location on the potato chromosome.

Supplementary Data 7 Synteny of the 24 *Nicotiana tabacum* linkage groups with the 12 potato chromosomes, based on whole genome sequences. Syntenic DNA blocks in each plot are positioned on the x axis according to their location in the *Nicotiana tabacum* linkage group. Each line on the y axis represents one potato chromosome.

Supplementary Data 8 Synteny of the 24 *Nicotiana tabacum* linkage groups with the 12 tomato chromosomes determined using MCScanX.

Supplementary Data 9 Synteny of the 24 *Nicotiana tabacum* linkage groups with the 12 potato chromosomes determined using MCScanX.

## Figures and Tables

**Table 1:** Assembly statistics of tobacco genomes.

**Statistic**	***N. tabacum*** **K326**	***N. tabacum*** **TN90**	***N. tabacum*** **BX**	***N. otophora***
Sequencing coverage	38 ×	49 ×	29 ×	66 ×
Contigs	711,416	478,142	797,404	1,120,175
Scaffolds	582,565	351,740	643,545	927,926
Superscaffolds	375,007	245,935	411,325	ND
Average length (bp)	9,954	15,121	9,082	2,901
Maximum length (bp)	3,856,062	3,846,415	3,726,272	1,052,670
N50 length (bp)	345,167	385,886	350,983	27,928
Total length (bp)	3,732,635,995	3,718,833,281	3,735,815,445	2,692,099,598
Undefined bases	134,062,984 (3.6%)	105,854,533 (2.8%)	182,189,689 (4.9%)	215,452,715 (8.0%)
Genome size (Gb)	4.60	4.41	4.57	2.70
Genome coverage	81.1%	84.3%	81.8%	99.7%
Repeat content	73.1%	78.9%	72.7%	ND

ND, not determined.

Genome sizes and coverage are estimated from 17-mer depth distributions.

## References

[b1] Peedin G. F. Tobacco cultivation. in:Specialty Crops (ed. Myers M. L. International Labor Organization (2011).

[b2] Lonitzer A. & Uffenbach P. Kreuterbuch The Bavarian State Library (1630).

[b3] von Linné C. & Houttuyn M. Allgemeines Register über die in den Sämtlichen Dreyzen Theilen des Linneischen Pflanzensystems Beschriebenen Gattungen und Arten Nebst Einem Besondern die Denselben Eigenen Synonymen Erläuternden Gabriel Nicolaus Raspe (1788).

[b4] Chase M. W. *et al.* Molecular systematics, GISH and the origin of hybrid taxa in Nicotiana (Solanaceae). Ann. Bot. (Lond). 92, 107–127 (2003). 10.1093/aob/mcg087 PMC4243627 12824072

[b5] Goodspeed T. H. The genus Nicotiana. Chron. Bot. 16, 102–135 (1954).

[b6] Knapp S., Chase M. W. & Clarkson J. J. Nomenclatural changes and a new sectional classification in Nicotiana (Solanaceae). Taxon 53, 73–82 (2004).

[b7] USDA. National Plant Germplasm System. Available at http://www.ars-grin.gov/npgs/ (2013).

[b8] Davis D. L. & Nielsen M. T. Tobacco: Production, Chemistry and Technology Blackwell Science Ltd (1999).

[b9] Zhang J., Zhang Y., Du Y., Chen S. & Tang H. Dynamic metabonomic responses of tobacco (*Nicotiana tabacum*) plants to salt stress. J. Proteome Res. 10, 1904–1914 (2011). 21323351 10.1021/pr101140n

[b10] Nagata T., Nemoto Y. & Hasezawa S. in:International Review of Cytology eds Jeon K. W., Friedlander M. Academic Press (1992).

[b11] Zimmerman J. L. & Goldberg R. B. DNA sequence organization in the genome of *Nicotiana tabacum*. Chromosoma 59, 227–252 (1977).

[b12] Renny-Byfield S. *et al.* Next generation sequencing reveals genome downsizing in allotetraploid *Nicotiana tabacum*, predominantly through the elimination of paternally derived repetitive DNAs. Mol. Biol. Evol. 28, 2843–2854 (2011). 21512105 10.1093/molbev/msr112

[b13] Leitch I. J. *et al.* The ups and downs of genome size evolution in polyploid species of Nicotiana (Solanaceae). Ann. Bot. (Lond). 101, 805–814 (2008). 10.1093/aob/mcm326 PMC2710205 18222910

[b14] Causse M. *et al.* The SOL-100 sequencing project. Available at http://solgenomics.net/organism/sol100/view.

[b15] Sierro N. *et al.* Reference genomes and transcriptomes of *Nicotiana sylvestris* and *Nicotiana tomentosiformis*. Genome. Biol. 14, R60 (2013). 23773524 10.1186/gb-2013-14-6-r60 PMC3707018

[b16] Kenton A., Parokonny A. S., Gleba Y. Y. & Bennett M. D. Characterization of the *Nicotiana tabacum* L. genome by molecular cytogenetics. Mol. Gen. Genet. 240, 159–169 (1993). 8355650 10.1007/BF00277053

[b17] Riechers D. E. & Timko M. P. Structure and expression of the gene family encoding putrescine N-methyltransferase in *Nicotiana tabacum*: new clues to the evolutionary origin of cultivated tobacco. Plant Mol. Biol. 41, 387–401 (1999). 10598105 10.1023/a:1006342018991

[b18] Wu F. *et al.* COSII genetic maps of two diploid Nicotiana species provide a detailed picture of synteny with tomato and insights into chromosome evolution in tetraploid *N. tabacum*. Theor. Appl. Genet. 120, 809–827 (2010). 19921141 10.1007/s00122-009-1206-z

[b19] Horn M. E., Woodard S. L. & Howard J. A. Plant molecular farming: systems and products. Plant Cell Rep. 22, 711–720 (2004). 14997337 10.1007/s00299-004-0767-1 PMC7079917

[b20] Bindler G. *et al.* A high density genetic map of tobacco (*Nicotiana tabacum* L.) obtained from large scale microsatellite marker development. Theor. Appl. Genet. 123, 219–230 (2011). 21461649 10.1007/s00122-011-1578-8 PMC3114088

[b21] Sierro N. *et al.* Whole genome profiling physical map and ancestral annotation of tobacco Hicks Broadleaf. Plant J. 75, 880–889 (2013). 23672264 10.1111/tpj.12247 PMC3824204

[b22] Martin F. *et al.* Design of a Tobacco Exon Array with application to investigate the differential cadmium accumulation property in two tobacco varieties. BMC Genomics 13, 674 (2012). 23190529 10.1186/1471-2164-13-674 PMC3602038

[b23] Bancroft I. *et al.* Dissecting the genome of the polyploid crop oilseed rape by transcriptome sequencing. Nat. Biotechnol. 29, 762–766 (2011). 21804563 10.1038/nbt.1926

[b24] Zonneveld B., Leitch I. & Bennett M. First nuclear DNA amounts in more than 300 angiosperms. Ann. Bot. (Lond). 96, 229–244 (2005). 10.1093/aob/mci170 PMC4246870 15905300

[b25] Xu X. *et al.* Genome sequence and analysis of the tuber crop potato. Nature 475, 189–195 (2011). 21743474 10.1038/nature10158

[b26] Varshney R. K. *et al.* Draft genome sequence of pigeonpea (*Cajanus cajan*), an orphan legume crop of resource-poor farmers. Nat. Biotechnol. 30, 83–89 (2012). 22057054 10.1038/nbt.2022

[b27] Li R. *et al.* *De novo* assembly of human genomes with massively parallel short read sequencing. Genome Res. 20, 265–272 (2010). 20019144 10.1101/gr.097261.109 PMC2813482

[b28] Murad L. *et al.* The origin of tobacco’s T genome is traced to a particular lineage within *Nicotiana tomentosiformis* (Solanaceae). Am. J. Bot. 89, 921–928 (2002). 21665691 10.3732/ajb.89.6.921

[b29] Kent W. J. BLAT—the BLAST-like alignment tool. Genome Res. 12, 656–664 (2002). 11932250 10.1101/gr.229202 PMC187518

[b30] Bombarely A., Edwards K. D., Sanchez-Tamburrino J. & Mueller L. A. Deciphering the complex leaf transcriptome of the allotetraploid species *Nicotiana tabacum*: a phylogenomic perspective. BMC Genomics 13, 406 (2012). 22900718 10.1186/1471-2164-13-406 PMC3582432

[b31] Bombarely A. *et al.* A draft genome sequence of *Nicotiana benthamiana* to enhance molecular plant-microbe biology research. Mol. Plant Microbe Interact. 25, 1523–1530 (2012). 22876960 10.1094/MPMI-06-12-0148-TA

[b32] Wang Y. *et al.* MCScanX: a toolkit for detection and evolutionary analysis of gene synteny and collinearity. Nucleic Acids Res. 40, e49–e49 (2012). 22217600 10.1093/nar/gkr1293 PMC3326336

[b33] Sato S. *et al.* The tomato genome sequence provides insights into fleshy fruit evolution. Nature 485, 635–641 (2012). 22660326 10.1038/nature11119 PMC3378239

[b34] Naim F. *et al.* Advanced engineering of lipid metabolism in *Nicotiana benthamiana* using a draft genome and the V2 viral silencing-suppressor protein. PLoS ONE 7, e52717 (2012). 23300750 10.1371/journal.pone.0052717 PMC3530501

[b35] Mayer K. *et al.* A physical, genetic and functional sequence assembly of the barley genome. Nature 491, 711–716 (2012). 23075845 10.1038/nature11543

[b36] Nystedt B. *et al.* The Norway spruce genome sequence and conifer genome evolution. Nature 497, 579 (2013). 23698360 10.1038/nature12211

[b37] Brenchley R. *et al.* Analysis of the bread wheat genome using whole-genome shotgun sequencing. Nature 491, 705–710 (2012). 23192148 10.1038/nature11650 PMC3510651

[b38] Zdobnov E. M. & Apweiler R. InterProScan–an integration platform for the signature-recognition methods in InterPro. Bioinformatics 17, 847–848 (2001). 11590104 10.1093/bioinformatics/17.9.847

[b39] Maimbo M., Ohnishi K., Hikichi Y., Yoshioka H. & Kiba A. S-glycoprotein-like protein regulates defense responses in Nicotiana plants against *Ralstonia solanacearum*. Plant Physiol. 152, 2023–2035 (2010). 20118275 10.1104/pp.109.148189 PMC2850023

[b40] Biastoff S., Brandt W. & Drager B. Putrescine N-methyltransferase—the start for alkaloids. Phytochemistry 70, 1708–1718 (2009). 19651420 10.1016/j.phytochem.2009.06.012

[b41] Itkin M. *et al.* Biosynthesis of antinutritional alkaloids in solanaceous crops is mediated by clustered genes. Science 341, 175–179 (2013). 23788733 10.1126/science.1240230

[b42] Lewis R. S., Milla S. R. & Levin J. S. Molecular and genetic characterization of *Nicotiana glutinosa* L. chromosome segments in tobacco mosaic virus-resistant tobacco accessions. Crop Sci. 45, 2355–2362 (2005).

[b43] Holmes F. O. Inheritance of resistance to tobacco-mosaic disease in tobacco. Phytopathology 28, 553–561 (1938).

[b44] Dunigan D. D., Golemboski D. B. & Zaitlin M. Analysis of the N gene of Nicotiana. in: *Plant Resistance to Viruses*. (eds Evered, D. & Harnett, S.) (John Wiley & Sons Ltd., 1987).

[b45] Whitham S. *et al.* The product of the tobacco mosaic virus resistance gene N: similarity to toll and the interleukin-1 receptor. Cell 78, 1101–1115 (1994). 7923359 10.1016/0092-8674(94)90283-6

[b46] Les Erickson F. *et al.* The helicase domain of the TMV replicase proteins induces the N-mediated defence response in tobacco. Plant J. 18, 67–75 (1999). 10341444 10.1046/j.1365-313x.1999.00426.x

[b47] Heggestad H. E., Clayton E., Neas M. & Skoog H. Development of Burley 21, the first wildfire-resistant tobacco variety, including results of variety trials. Bulletin 321, University of Tennessee, Agricultural Experiment Station (1960).

[b48] Noguchi S., Tajima T., Yamamoto Y., Ohno T. & Kubo T. Deletion of a large genomic segment in tobacco varieties that are resistant to potato virus Y (PVY). Mol. Gen. Genet. 262, 822–829 (1999). 10628866 10.1007/s004380051146

[b49] Acosta-Leal R. & Xiong Z. Complementary functions of two recessive R-genes determine resistance durability of tobacco ‘Virgin A Mutant’ (VAM) to Potato virus Y. Virology 379, 275–283 (2008). 18682305 10.1016/j.virol.2008.06.026

[b50] Julio E. *et al.* Characterization of PVY (Potato Virus Y) resistance in tobacco: potential role of an eIF4E gene identified by high throughput sequencing technologies. Plant and Animal Genome XXI, San Diego, CA, USA (2013).

[b51] Murashige T. & Skoog F. A revised medium for rapid growth and bio assays with tobacco tissue cultures. Physiol. Plant. 15, 473–497 (1962).

[b52] Gordon A. & Hannon G. Fastx-toolkit. Available at http://hannonlab.cshl.edu/fastx_toolkit (2012).

[b53] Magoč T. & Salzberg S. L. FLASH: fast length adjustment of short reads to improve genome assemblies. Bioinformatics 27, 2957–2963 (2011). 21903629 10.1093/bioinformatics/btr507 PMC3198573

[b54] Frith M. C. A new repeat-masking method enables specific detection of homologous sequences. Nucleic Acids Res. 39, e23–e23 (2011). 21109538 10.1093/nar/gkq1212 PMC3045581

[b55] Benson G. Tandem repeats finder: a program to analyze DNA sequences. Nucleic Acids Res. 27, 573 (1999). 9862982 10.1093/nar/27.2.573 PMC148217

[b56] Kurtz S. *et al.* Versatile and open software for comparing large genomes. Genome Biol. 5, R12 (2004). 14759262 10.1186/gb-2004-5-2-r12 PMC395750

[b57] Frith M. C., Hamada M. & Horton P. Parameters for accurate genome alignment. BMC Bioinformatics 11, 80 (2010). 20144198 10.1186/1471-2105-11-80 PMC2829014

[b58] Kiełbasa S. M., Wan R., Sato K., Horton P. & Frith M. C. Adaptive seeds tame genomic sequence comparison. Genome Res. 21, 487–493 (2011). 21209072 10.1101/gr.113985.110 PMC3044862

[b59] Price A. L., Jones N. C. & Pevzner P. A. *De novo* identification of repeat families in large genomes. Bioinformatics 21, i351–i358 (2005). 15961478 10.1093/bioinformatics/bti1018

[b60] Altschul S. F., Gish W., Miller W., Myers E. W. & Lipman D. J. Basic local alignment search tool. J. Mol. Biol. 215, 403–410 (1990). 2231712 10.1016/S0022-2836(05)80360-2

[b61] Langmead B. & Salzberg S. L. Fast gapped-read alignment with Bowtie 2. Nat. Methods 9, 357–359 (2012). 22388286 10.1038/nmeth.1923 PMC3322381

[b62] Kim D. *et al.* TopHat2: accurate alignment of transcriptomes in the presence of insertions, deletions and gene fusions. Genome Biol. 14, R36 (2013). 23618408 10.1186/gb-2013-14-4-r36 PMC4053844

[b63] Trapnell C. *et al.* Transcript assembly and quantification by RNA-Seq reveals unannotated transcripts and isoform switching during cell differentiation. Nat. Biotechnol. 28, 511–515 (2010). 20436464 10.1038/nbt.1621 PMC3146043

[b64] Grabherr M. G. *et al.* Full-length transcriptome assembly from RNA-Seq data without a reference genome. Nat. Biotechnol. 29, 644–652 (2011). 21572440 10.1038/nbt.1883 PMC3571712

[b65] Li L., Stoeckert C. J. & Roos D. S. OrthoMCL: identification of ortholog groups for eukaryotic genomes. Genome Res. 13, 2178–2189 (2003). 12952885 10.1101/gr.1224503 PMC403725

[b66] Anders S. & Huber W. Differential expression analysis for sequence count data. Genome Biol. 11, R106 (2010). 20979621 10.1186/gb-2010-11-10-r106 PMC3218662

[b67] Maere S., Heymans K. & Kuiper M. BiNGO: a Cytoscape plugin to assess overrepresentation of gene ontology categories in biological networks. Bioinformatics 21, 3448–3449 (2005). 15972284 10.1093/bioinformatics/bti551

[b68] Slater G. S. C. & Birney E. Automated generation of heuristics for biological sequence comparison. BMC Bioinformatics 6, 31 (2005). 15713233 10.1186/1471-2105-6-31 PMC553969

[b69] Korf I. Gene finding in novel genomes. BMC Bioinformatics 5, 59 (2004). 15144565 10.1186/1471-2105-5-59 PMC421630

[b70] Julio E., Verrier J. & de Borne F. D. Development of SCAR markers linked to three disease resistances based on AFLP within *Nicotiana tabacum* L. Theor. Appl. Genet. 112, 335–346 (2006). 16283232 10.1007/s00122-005-0132-y

