# Supplementary material for: The tobacco genome sequence and its comparison with those of tomato and potato
Source: Nat Commun. 2014 May 8;5:3833. doi: 10.1038/ncomms4833 (PMC4024737; doi:10.1038/ncomms4833)
Supplement: Supplementary Figures, Tables, Notes and References — Supplementary Figures 1-7, Supplementary Tables 1-13, Supplementary Notes 1-4 and Supplementary References [file ncomms4833-s1.pdf]

Supplementary Figure 1

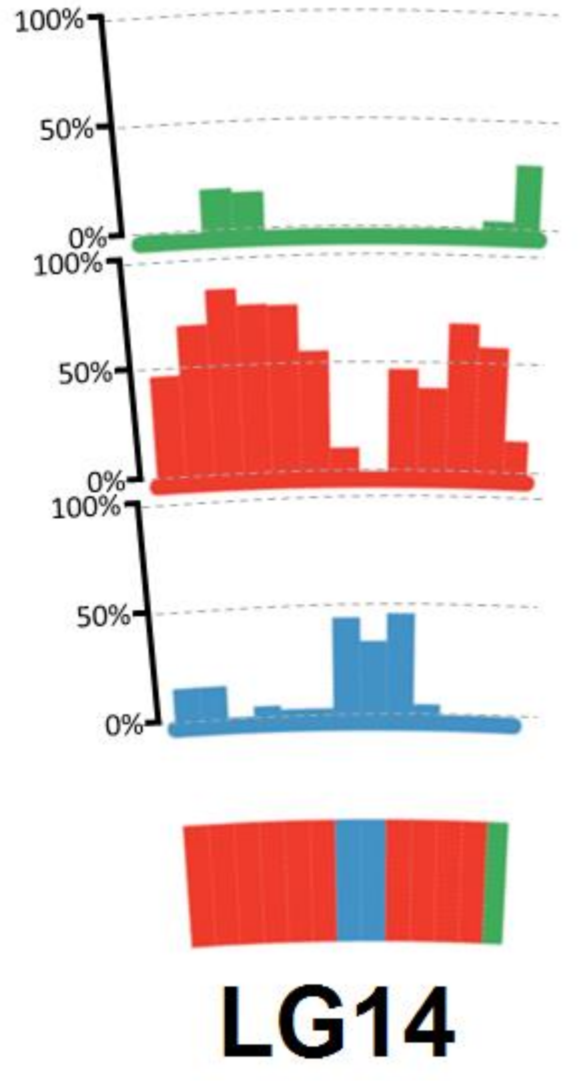

***Nicotiana otophora* sequences mapped to the tobacco linkage group 14.**

Enlarged tobacco linkage group 14 showing the percentage of the genome for which more bases are covered by sequence identity to *N. otophora* (green) than to *N. sylvestris* (blue) or *N. tomentosiformis* (red).

Supplementary Figure 2

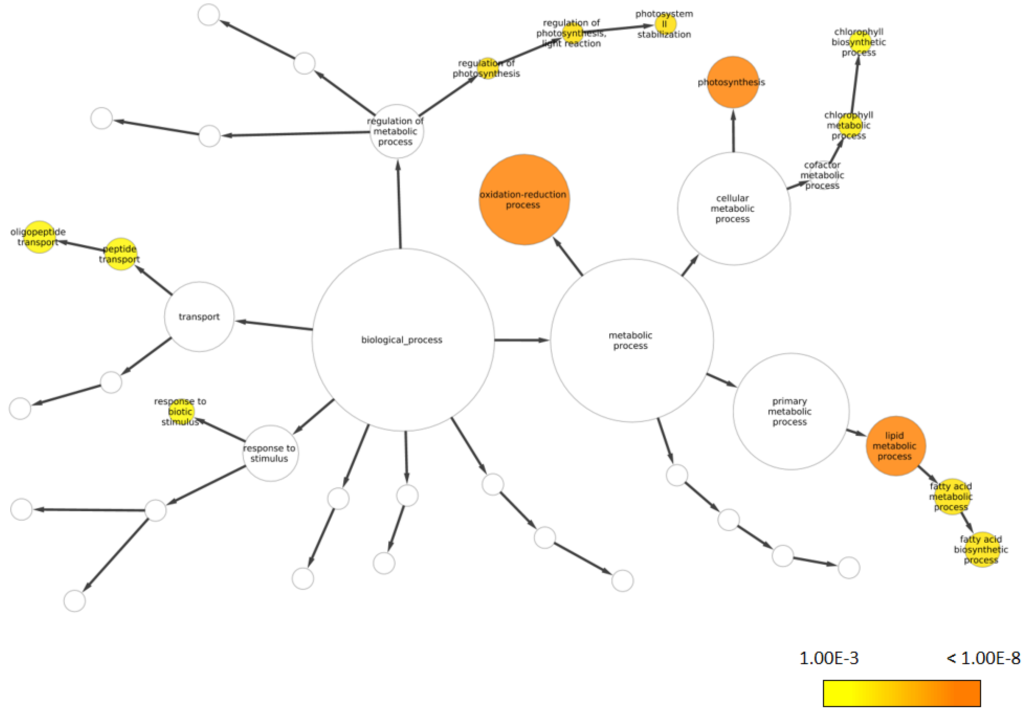

GO term enrichment in *N. tabacum* TN90 leaf tissue, relative to root tissue.

The circles are shaded based on significance level. GO terms represented by the white nodes are not significantly overrepresented, and nodes without labeling are enriched in root and are included here to ease comparison with Supplementary Figure 3. The radius of each circle denotes the number of genes in each category.

Supplementary Figure 3

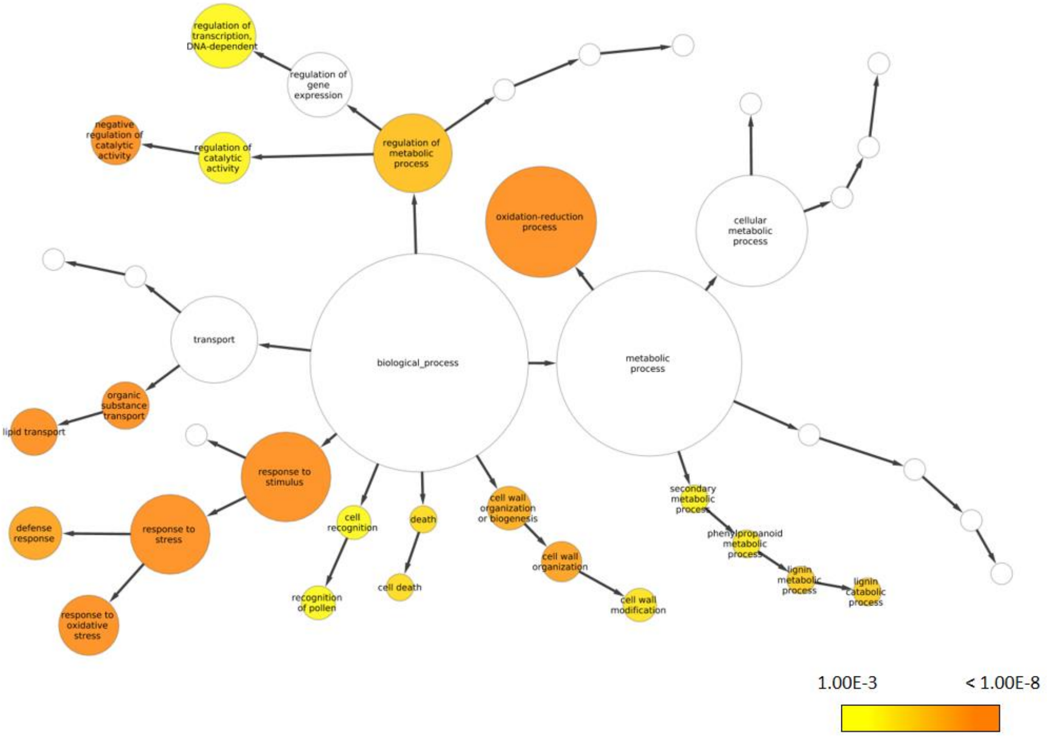

GO term enrichment in *N. tabacum* TN90 root tissue, relative to leaf tissue.

The circles are shaded based on significance level. GO terms represented by the white nodes are not significantly overrepresented, and nodes without labeling are enriched in leaf and are included here to ease comparison with Supplementary Figure 2. The radius of each circle denotes the number of genes in each category.

Supplementary Figure 4

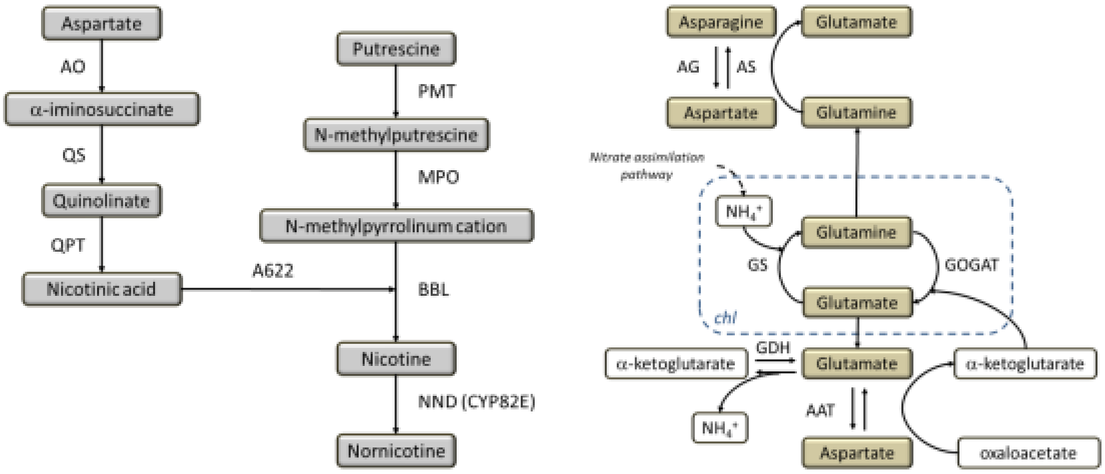

a b

Classical tobacco pathways

a, Alkaloid pathway in *N. tabacum*. b, Glutamate/aspartate pathway in *N. tabacum*.

Supplementary Figure 5

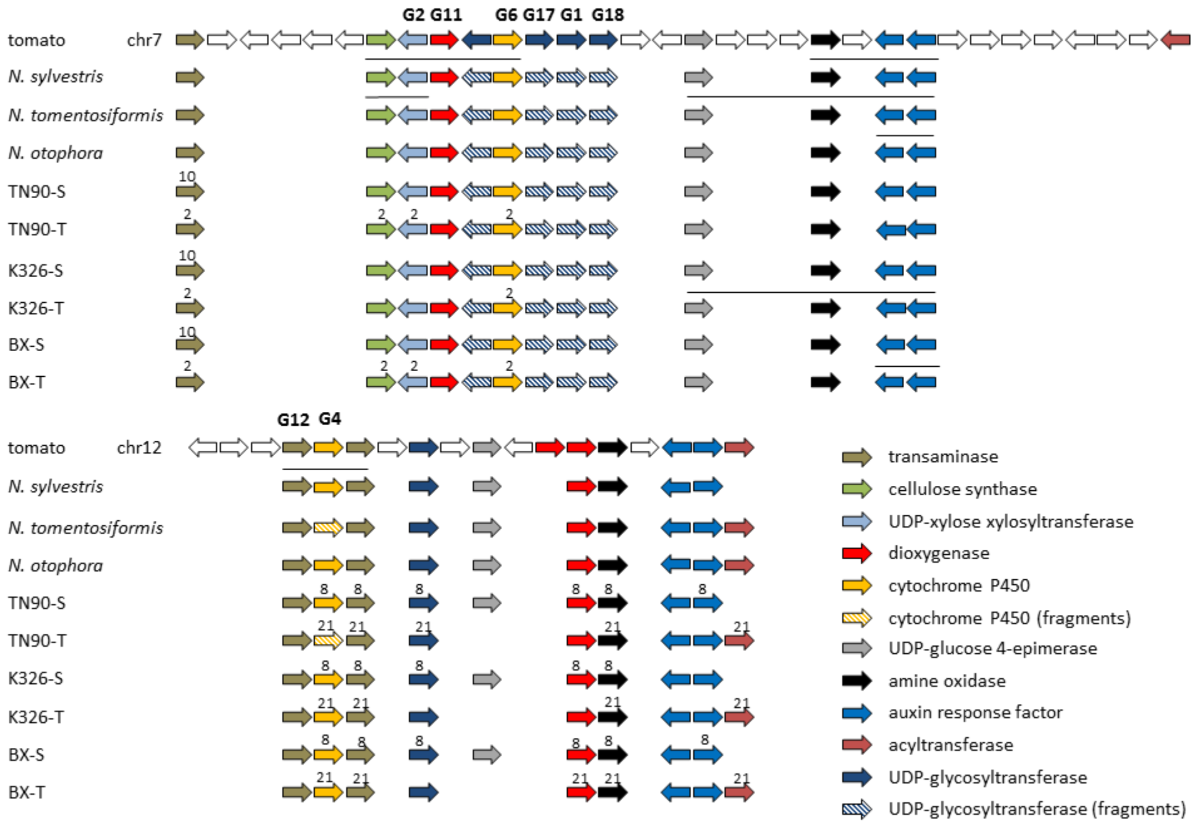

Putative steroidal alkaloids biosynthesis genes from Nicotiana genomes mapped to the syntenic regions on chromosomes 7 and 12 of *Solanum lycopersicum*. Gene families are indicated by colored arrows, a hatch pattern is used in the case of partial mapping. The arrowheads represent the direction of transcript in *S. lycopersicum*. The numbers above the arrows indicate the linkage group from the tobacco genetic map for the gene, and black lines above the arrows group genes located on the same sequencing scaffold. G, GAME.

Supplementary Figure 6

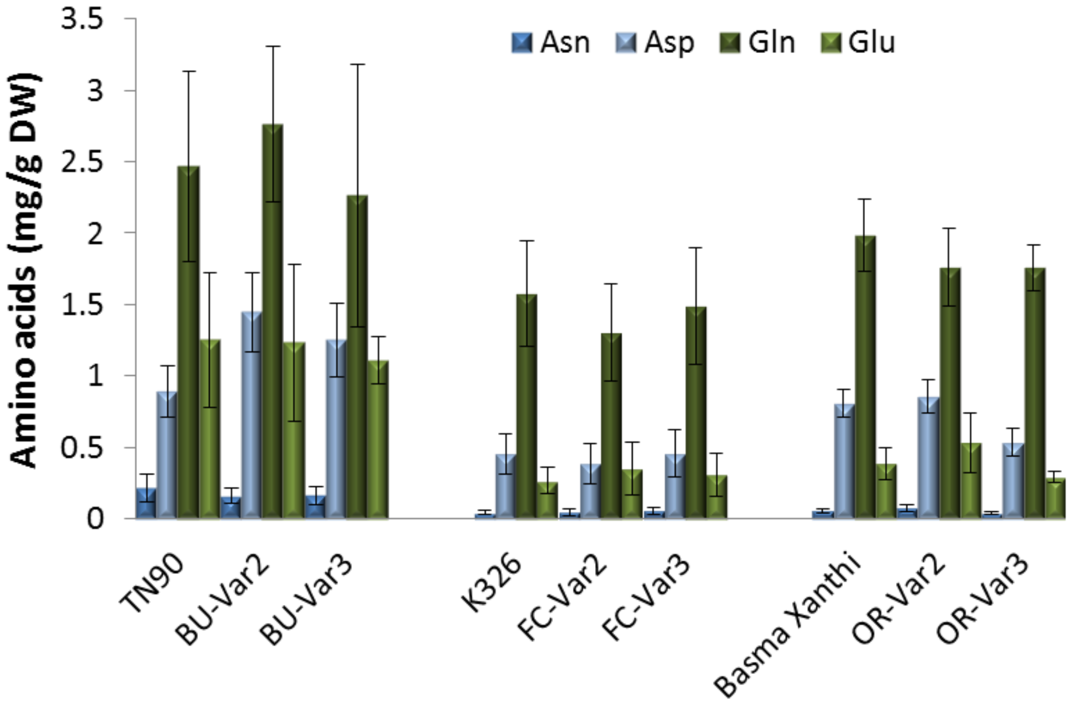

Total free amino acids in green leaves of Burley, Flue-cured and Oriental tobacco.

Error bars indicate the standard deviation on a sample size of N=5 field replicates Burley (TN90: 10.4 ± 4.2 mg/g), Flue-cured (K326: 2.8 ± 0.8 mg/g), and Oriental (Basma Xanthi: 4.6 ± 0.9 mg/g).

Supplementary Figure 7

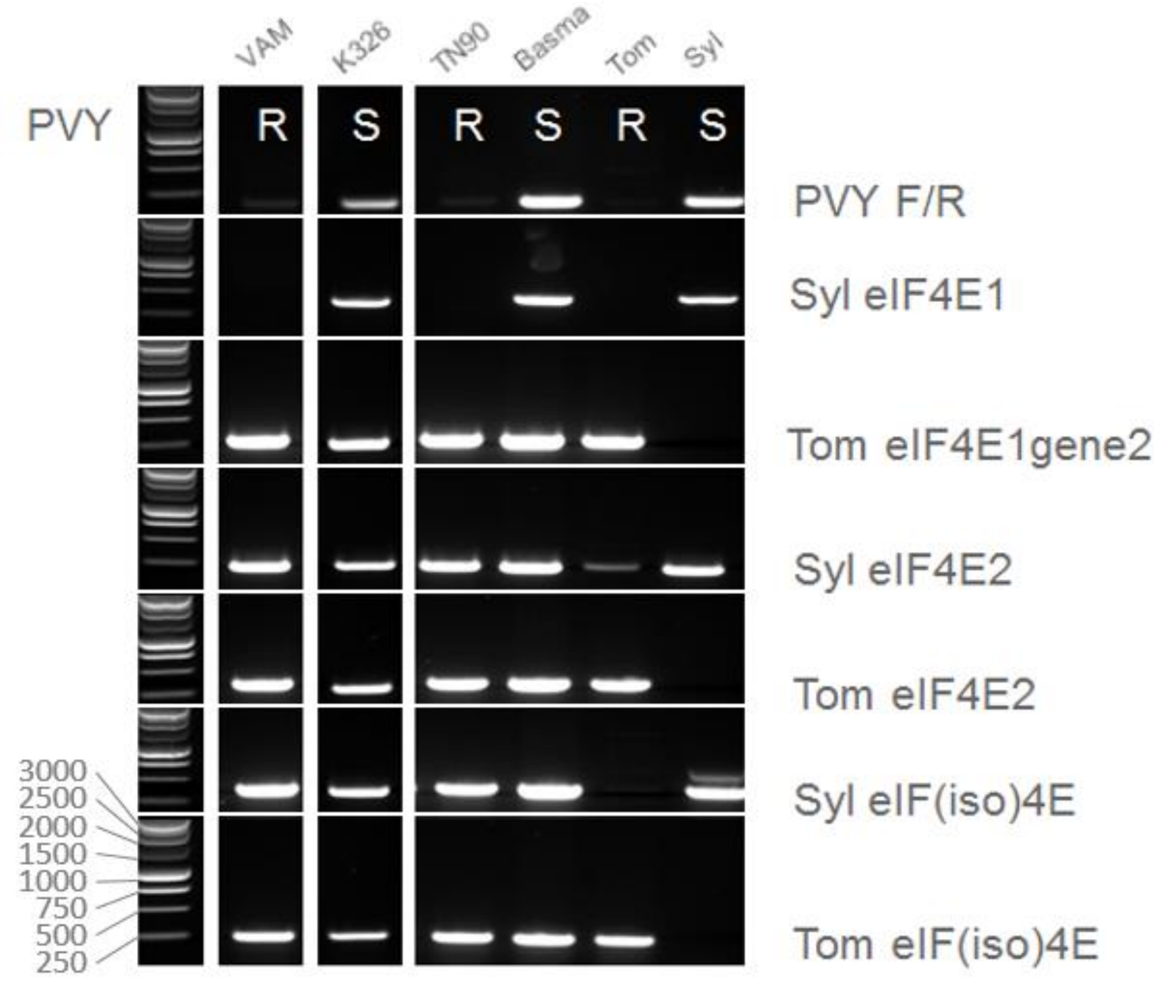

PVY resistance in *N. tabacum* varieties. Detection by PCR amplification of eIF4E gene family from genomic DNA isolated from tobacco varieties either PVY resistant (Virgin A mutante (VAM) and TN90) or PVY susceptible (K326 and Basma Xanthi (Basma)), as well as the DNA of the two species related to tobacco ancestors *N. sylvestris* (Syl) and *N. tomentosiformis* (Tom). Gels for each eIF4E gene family member are stacked. The marker sizes are given in bases.

Supplementary Table 1: Reference sequences mapped to the tobacco genomes with at least 80% coverage

Reference set	Genome	Minimum % identity	% of reference genes mapped
Tobacco Unigene (NCBI, build 17) (N=24432)	BX	97	0.555706
	K326	97	0.559062
	TN90	97	0.58730
Tobacco Unigene (SGN, version 2) (N=84602)	BX	97	0.49922
	K326	97	0.500827
	TN90	97	0.522257
Tobacco transcriptome (SGN) (N=28568)	BX	97	0.824209
	K326	97	0.83597
	TN90	97	0.856238
Potato transcriptome (N=56218)	BX	60	0.614572
	K326	60	0.614625
	TN90	60	0.624889
Tomato transcriptome (itag 2.3) (N=34675)	BX	60	0.590425
	K326	60	0.591146
	TN90	60	0.605999

Supplementary Table 2: Tobacco genome repeat analysis

Repeat type	*N. tabacum* K326		*N. tabacum* TN90		*N. tabacum* BX	
	Number of bases	%	Number of bases	%	Number of bases	%
LINE	14,148,791	0.3	42,654,837	1.2	14,868,110	0.4
SINE	12,040,488	0.3	9,919,487	0.3	9,685,881	0.3
LTR/copia	454,635,803	12.6	562,278,774	15.6	481,440,114	13.6
LTR/gypsy	691,108,840	19.2	725,369,362	20.1	711,742,068	20.0
LTR/others	310,382,819	8.6	342,591,545	9.4	354,162,915	10.0
Transposons	119,232,636	3.3	82,730,798	2.3	55,111,415	1.6
Retrotransposons	397,591,765	11.1	465,589,680	12.9	281,071,335	7.9
Simple repeats	8,388,564	0.2	8,072,771	0.2	7,849,477	0.2
Low complexity	25,334,664	0.7	18,488,786	0.5	24,800,993	0.7
Others	275,681,927	7.7	280,209,254	7.8	324,979,859	9.1
Unknown	323,038,990	9.0	312,327,882	8.6	316,121,222	8.9
**Total**	**2,631,585,287**	**73.1**	**2,850,233,176**	**78.9**	**2,581,833,389**	**72.7**

Over 70% of the sequenced genomes are repeat elements, with gypsy-like long terminal repeats (LTRs) representing about 20% of repeats and copia-like LTRs representing 13–15%. A smaller fraction of transposons was observed in Oriental (1.6%) compared with other varieties, eventually due to an assembly induced bias. Differences in repeat superfamilies were previously reported among *Nicotiana* species genomes; however, no significant deviations were found between *N. tabacum* and its ancestral species, which could be because of our broader definition of repeat superfamilies.

Supplementary Table 3: RNA-Seq read mapping statistics

Sample	Tissue		Reads	Reads mapped	Reads unmapped	% mapped	% unmapped
Basma Xanthi	leaf	1	123,452,574	115,157,103	8,295,471	93.28%	6.72%
Basma Xanthi	leaf	2	109,523,869	102,094,117	7,429,752	93.22%	6.78%
Basma Xanthi	leaf	3	82,944,896	76,973,764	5,971,132	92.80%	7.20%
Basma Xanthi	root	1	169,529,928	154,101,015	15,428,913	90.90%	9.10%
Basma Xanthi	root	2	149,218,023	135,771,135	13,446,888	90.99%	9.01%
Basma Xanthi	root	3	189,723,805	172,921,787	16,802,018	91.14%	8.86%
K326	leaf	1	89,013,625	81,683,865	7,329,760	91.77%	8.23%
K326	leaf	2	120,002,672	110,620,875	9,381,797	92.18%	7.82%
K326	leaf	3	145,597,369	135,044,428	10,552,941	92.75%	7.25%
K326	root	1	137,236,059	122,701,555	14,534,504	89.41%	10.59%
K326	root	23	141,896,638	128,443,962	13,452,676	90.52%	9.48%
K326	root	4	78,260,108	72,811,250	5,448,858	93.04%	6.96%
TN90	leaf	1	66,965,360	62,548,052	4,417,308	93.40%	6.60%
TN90	leaf	2	163,085,389	154,556,657	8,528,732	94.77%	5.23%
TN90	leaf	3	124,396,098	115,831,842	8,564,256	93.12%	6.88%
TN90	root	1	97,505,605	88,295,539	9,210,066	90.55%	9.45%
TN90	root	2	91,777,342	82,794,213	8,983,129	90.21%	9.79%
TN90	root	4	79,177,778	73,040,003	6,137,775	92.25%	7.75%
TN90	Dry Capsule	1	38,962,084	35,692,365	3,269,719	91.61%	8.39%
TN90	Dry Capsule	2	52,430,895	47,849,852	4,581,043	91.26%	8.74%
TN90	Dry Capsule	3	38,833,426	34,819,589	4,013,837	89.66%	10.34%
TN90	Immature Flower	1	101,246,759	93,525,069	7,721,690	92.37%	7.63%
TN90	Immature Flower	2	82,744,754	76,473,670	6,271,084	92.42%	7.58%
TN90	Immature Flower	3	72,481,303	65,795,469	6,685,834	90.78%	9.22%
TN90	Mature Flower	1	102,913,251	94,883,722	8,029,529	92.20%	7.80%
TN90	Mature Flower	2	88,289,854	81,534,552	6,755,302	92.35%	7.65%
TN90	Mature Flower	3	78,263,935	70,973,687	7,290,248	90.69%	9.31%
TN90	Mature Leaf Middle Stalk	1	72,464,309	66,878,005	5,586,304	92.29%	7.71%
TN90	Mature Leaf Middle Stalk	2	51,536,209	47,025,043	4,511,166	91.25%	8.75%
TN90	Mature Leaf Middle Stalk	3	79,749,353	71,763,136	7,986,217	89.99%	10.01%
TN90	Root	1	68,806,152	61,344,009	7,462,143	89.15%	10.85%
TN90	Root	2	22,187,378	19,198,472	2,988,906	86.53%	13.47%
TN90	Root	3	88,874,554	78,519,406	10,355,148	88.35%	11.65%
TN90	Senescent Flower	1	51,868,571	47,992,194	3,876,377	92.53%	7.47%
TN90	Senescent Flower	2	49,687,818	45,639,859	4,047,959	91.85%	8.15%
TN90	Senescent Leaf Lower Stalk	1	72,477,148	66,897,347	5,579,801	92.30%	7.70%
TN90	Senescent Leaf Lower Stalk	2	77,035,275	69,967,452	7,067,823	90.83%	9.17%
TN90	Senescent Leaf Lower Stalk	3	91,238,092	82,860,495	8,377,597	90.82%	9.18%
TN90	Stem	1	47,825,302	44,100,407	3,724,895	92.21%	7.79%
TN90	Stem	2	58,384,926	53,085,199	5,299,727	90.92%	9.08%
TN90	Stem	3	84,731,044	77,294,392	7,436,652	91.22%	8.78%
TN90	Young Leaf Upper Stalk	1	48,932,070	45,346,419	3,585,651	92.67%	7.33%
TN90	Young Leaf Upper Stalk	2	62,966,746	58,258,041	4,708,705	92.52%	7.48%
TN90	Young Leaf Upper Stalk	3	75,528,699	68,275,993	7,252,706	90.40%	9.60%

Supplementary Table 4: Transcriptome statistics

Variety	Tissues*	Cufflinks gene models	Cufflinks transcripts	Unique predicted ORFs (>= 100aa)
Basma Xanthi	2	93,650	145,662	72,339
K326	2	91,870	144,687	72,646
TN90	2	81,404	134,694	71,308
TN90	9	93,330	188,510	90,152

Supplementary Table 5: Protein function statistics for proteins of at least 100 amino acids and annotated with InterProScan

Variety	Tissues	Total assignments	Total unique assignments	Proteins with GO terms assigned	Genes with GO terms assigned	Unique GO terms assigned
Basma Xanthi	2	142,588	104,596	40,460	28,128	1,446
K326	2	144,472	105,975	40,864	28,360	1,431
TN90	2	148,482	108,092	41,215	27,796	1,435
TN90	9	184,155	134,960	51,833	29,663	1,446

Supplementary Table 6: Alkaloid pathway in *N. tabacum*

UniProt Gene Name	Gene Name	BX		K326		TN90		Probe	FC-V21		BU-V5	
		L	R	L	R	L	R		L	R	L	R
	AO1-S	10.5	3.1	15.1	14.3	10.9	5.8	NtPMIa1g81967e3_st	5.2	4.7	5.7	5.1
	AO1-T	20.5	3.1	14.9	6.9	7.4	4.1	NtPMIa1g129811e1_st	5.9	6.9	6.2	5.9
	AO2-S	0.1	34.5	0.0	27.1	0.0	81.9	NtPMIa1g70983e2_st	2.1	9.3	2.3	10.7
	AO2-T	0.1	35.1	0.0	25.0	0.1	90.0	NtPMIa1g40350e1_st	1.9	9.5	2.1	11.0
BBLa	BBL1-S	0.1	26.1	0.1	29.3	0.1	68.9	NtPMIa1g63910e1_st	3.0	8.8	1.8	10.6
BBLb	BBL1-T	0.0	13.7	0.0	22.4	0.1	53.4	NtPMIa1g78615e1_st	2.3	6.1	2.5	7.4
BBLc	BBL2-S-1	0.3	3.1	0.2	2.1	0.3	9.1	NtPMIa1g18814e1_st	2.2	8.8	3.2	11.0
	BBL2-S-2	0.2	1.5	0.4	0.4	0.3	3.1	NtPMIa1g162828e1_st	1.3	4.8	2.0	7.2
	BBL2-T	0.3	4.8	0.3	4.7	0.2	7.1	NtPMIa1g49014e1_st	2.2	9.4	2.3	11.2
	BBL3-S	0.1	0.1	0.1	0.0	0.1	0.1	NtPMIa1g44351e1_st	3.1	2.9	3.3	2.2
BBLd	BBL3-T	0.2	0.2	0.3	0.0	0.4	0.1	NtPMIa1g108338e1_s_st	2.3	2.4	3.4	3.0
PMT1	PMT1-S	0.0	2.5	0.0	5.3	0.0	20.6	NtPMIa1g79624e6_x_st	1.8	5.8	2.0	8.7
PMT2	PMT2-T	0.0	28.1	0.0	32.6	0.0	101.6	n.a.	-	-	-	-
PMT3	PMT3-S	0.1	35.0	0.0	34.8	0.0	59.9	NtPMIa1g33681e1_st	3.2	7.3	2.6	9.8
PMT4	PMT4-S	0.0	5.5	0.0	19.8	0.0	36.1	NtPMIa1g7934e1_x_st	3.2	8.0	2.0	10.8
	PMT5-T	0.1	25.0	0.0	23.2	0.1	206.6	NtPMIa1g115369e1_st	2.0	6.6	3.3	9.3
	A622-S	0.0	29.0	0.1	38.7	0.0	93.6	NtPMIa1g134618e1_st	2.3	8.5	2.7	10.5
	A622-T	0.0	36.8	0.1	48.5	0.1	118.4	NtPMIa1g13686e2_st	1.0	5.4	1.4	8.4
	QPT1-S	13.7	12.5	-	-	12.3	9.2	n.a.	-	-	-	-
	QPT1-T	10.0	7.3	26.9	17.4	10.3	7.9	NtPMIa1g62560e1_st	7.9	8.5	7.9	10.3
	QPT2-S	6.3	70.7	-	-	9.6	135.8	n.a.	-	-	-	-
	QPT2-T	47.0	79.4	70.1	143.5	21.3	110.4	NtPMIa1g164647e1_st	4.3	5.6	2.4	7.3
	QS-S	27.1	80.3	27.0	55.8	16.9	107.4	NtPMIa1g19986e2_st	4.7	9.8	6.1	11.9
	QS-T	13.5	74.4	16.5	47.4	10.9	106.1	NtPMIa1g42292e1_st	8.2	11.4	8.7	12.7
MPO1	MPO1-S	0.0	17.4	0.0	22.8	0.1	27.9	NtPMIa1g96433e1_st	3.6	7.5	3.5	9.4
	MPO1-T	0.2	18.8	0.2	18.7	0.3	23.2	NtPMIa1g82759e4_st	5.1	9.5	4.8	11.2
	MPO2-S	0.0(na)	0.0(na)	0.0	0.1	0.0	0.1	NtPMIa1g79267e2_st	1.6	2.0	2.2	2.0
MPO2	MPO2-T	0.5	0.5	0.4	1.0	0.3	0.8	NtPMIa1g21701e2_st	3.6	4.7	4.0	4.9
	MPO3-S	12.0	1.3	16.4	1.2	27.3	2.7	NtPMIa1g169932e1_st	8.7	5.7	8.4	6.1
	MPO3-T	8.3	2.0	13.9	2.7	11.9	4.1	NtPMIa1g192469e1_st	7.2	5.7	6.9	5.9
	NND1	1.7	0.2	1.4	0.2	0.3	0.1	n.a	-	-	-	-
CYP82E2	NND2-S-1	44.2	2.5	1.6	4.5	0.3	4.3	n.a	-	-	-	-

	NND2-S-2	1.4	0.1	0.0	0.1	0.0	0.1	NtPMIa1g182830e1_x_st	3.8	3.1	3.8	2.6
CYP82E3	NND3-S-1	6.0	0.1	0.3	0.0	0.9	0.0	NtPMIa1g11557e1_st	2.6	2.8	2.2	1.9
	NND3-S-2	12.2	0.4	3.6	0.0	0.3	0.0	NtPMIa1g48099e1_st	5.6	2.7	4.9	3.7
CYP82E4v2	NND4-T	0.0	0.0	0.0	0.0	0.5	0.0	NtPMIa1g156277e1_x_st	1.3	1.3	1.0	1.3
CYP82E5v2	NND5-T	15.3	27.2	16.8	40.8	11.3	28.4	n.a.	-	-	-	-
CYP82E10	NND5-S	4.6	45.1	4.2	23.1	4.8	12.3	NtPMIa1g132193e1_st	4.1	5.7	4.6	6.0

R: root; L: leaf. Columns 3–8 give the average FPKM of three biological replicates.

Columns 10–13 give the measured Affymetrix $\log_2$ expression

Supplementary Table 7: Differential gene expression significance of alkaloid pathway in *N. tabacum*

gene	BX				K326				TN90			
	cuffdiff		HTSeq/DESeq		cuffdiff		HtSeq/DESeq		cuffdiff		HTSeq/DESeq	
	log2 FC	q_value	log2 FC	padj	log2 FC	q_value	log2 FC	padj	log2 FC	q_value	log2 FC	padj
AO1-S	-1.8	8.30E-06	-1.7	4.74E-02	-0.1	9.58E-01	-0.1	8.71E-01	-0.9	1.03E-02	-1.0	1.67E-04
AO1-T	-2.7	7.80E-14	-2.6	3.23E-09	-1.1	6.03E-02	-1.2	1.63E-01	-0.9	3.05E-02	-0.8	8.20E-03
AO2-S	9.0	0.00E+00	9.4	3.46E-18	10.2	2.97E-10	10.2	3.01E-52	11.0	0.00E+00	10.6	1.86E-30
AO2-T	9.0	0.00E+00	9.3	5.81E-28	11.8	1.26E-04	12.2	6.28E-54	10.3	0.00E+00	10.5	1.55E-30
BBL1-S	8.5	0.00E+00	8.5	4.09E-26	8.9	0.00E+00	9.1	1.62E-52	9.5	0.00E+00	9.6	2.57E-45
BBL1-T	8.2	6.57E-15	8.2	8.04E-30	9.2	0.00E+00	9.3	7.55E-52	9.4	0.00E+00	9.6	1.57E-30
BBL2-S-1	3.2	1.84E-04	3.4	1.82E-07	3.5	1.69E-04	3.5	2.86E-09	5.1	0.00E+00	7.6	3.98E-12
BBL2-S-2	3.1	5.36E-03	2.8	1.59E-07	0.1	9.82E-01	0.8	5.85E-01	3.3	2.65E-06	3.2	1.24E-09
BBL2-T	3.9	1.04E-08	3.9	4.88E-08	3.9	1.27E-08	3.7	3.05E-14	4.9	0.00E+00	4.8	1.30E-08
BBL3-S	-0.1	1.00E+00	0.1	1.00E+00	-1.1	1.00E+00	-0.8	8.66E-01	0.2	1.00E+00	0.5	7.70E-01
BBL3-T	-0.2	9.17E-01	-0.2	9.33E-01	-2.9	1.65E-02	-3.1	7.40E-04	-1.8	5.01E-02	-2.0	1.05E-02
PMT1-S	7.8	3.39E-03	8.3	4.83E-08	13.1	3.30E-01	Inf	2.82E-17	10.5	8.16E-07	10.9	2.12E-15
PMT2-T	9.7	2.12E-03	9.2	8.05E-21	9.7	6.28E-04	10.4	7.68E-18	11.6	1.66E-04	12.1	1.00E-35
PMT3-S	9.3	0.00E+00	9.1	1.34E-14	9.7	3.99E-12	10.0	7.64E-31	12.2	6.03E-09	12.7	8.78E-25
PMT4-S	7.9	3.00E-05	7.7	8.44E-11	9.0	2.29E-11	9.0	2.76E-22	9.7	0.00E+00	10.5	2.47E-11
PMT5-T	7.9	4.66E-13	8.0	5.55E-20	Inf	3.53E-13	Inf	2.75E-12	10.9	0.00E+00	11.1	3.30E-20
A622-S	9.8	3.63E-09	9.4	7.80E-17	9.5	1.47E-13	10.3	2.38E-50	11.1	0.00E+00	10.9	4.15E-31
A622-T	9.6	2.02E-12	9.6	1.59E-21	9.4	0.00E+00	9.7	1.59E-44	10.6	0.00E+00	10.9	9.76E-42
QPT1-S	-0.1	8.67E-01	-0.1	8.51E-01	-	-	-	-	-0.4	3.11E-01	-0.4	1.27E-01
QPT1-T	-0.5	4.24E-01	-0.5	2.57E-01	-0.6	3.31E-01	-0.6	3.20E-01	-0.4	3.85E-01	-0.3	4.76E-01
QPT2-S	3.5	0.00E+00	3.9	1.23E-15	-	-	-	-	3.8	0.00E+00	4.0	8.47E-24
QPT2-T	0.8	7.69E-02	0.9	9.79E-03	1.0	1.44E-01	1.1	2.13E-02	2.4	6.09E-12	2.4	3.17E-07
QS-S	1.6	2.29E-04	1.6	3.18E-03	1.0	1.59E-01	1.1	2.40E-02	2.7	3.31E-11	2.7	5.54E-07
QS-T	2.5	3.66E-11	2.5	8.49E-09	1.5	8.08E-03	1.6	2.47E-04	3.3	0.00E+00	3.3	2.52E-05
MPO1-S	8.5	0.00E+00	9.2	8.06E-53	8.9	0.00E+00	9.1	1.27E-53	9.0	0.00E+00	9.3	2.00E-90
MPO1-T	-	-	-	-	6.5	0.00E+00	6.6	2.40E-38	6.4	0.00E+00	6.4	1.06E-56
MPO2-S	1.2	1.00E+00	1.3	1.00E+00	3.2	1.00E+00	4.0	2.50E-03	2.2	1.00E+00	2.9	1.38E-02
MPO2-T	-0.1	9.31E-01	0.0	1.00E+00	1.3	1.06E-01	1.4	8.61E-03	1.4	3.18E-02	1.6	4.67E-04
MPO3-S	-3.2	0.00E+00	-3.2	8.24E-05	-3.7	1.53E-11	-3.6	3.58E-04	-3.4	0.00E+00	-3.4	3.45E-34
MPO3-T	-2.0	7.52E-08	-1.9	6.06E-08	-2.3	3.86E-06	-2.2	1.07E-04	-1.5	3.52E-06	-1.5	6.12E-08
NND1	-3.0	4.89E-04	-2.7	2.48E-02	-3.1	1.61E-03	-3.2	1.94E-05	-1.5	3.49E-01	-1.3	5.08E-01
NND2-S-1	-4.2	3.74E-14	-3.9	3.34E-01	1.5	4.26E-02	1.7	2.68E-01	3.7	6.42E-08	3.9	5.93E-06
NND2-S-2	-3.4	4.15E-05	-3.0	3.94E-01	Inf	1.00E+00	Inf	3.97E-03	Inf	1.00E+00	Inf	1.85E-02

NND3-S-1	-6.1	0.00E+00	-6.1	1.59E-01	-6.5	8.14E-02	-6.6	1.81E-02	-18.9	9.42E-01	-Inf	1.08E-02
NND3-S-2	-4.8	0.00E+00	-4.9	8.29E-04	-10.1	2.60E-03	-10.8	2.13E-02	-12.6	7.87E-01	-Inf	3.27E-07
NND4-T	2.9	1.00E+00	1.5	9.84E-01	9.7	1.00E+00	Inf	1.95E-01	-6.7	3.50E-02	-6.5	2.90E-01
NND5-S	3.3	0.00E+00	3.4	1.44E-27	2.4	5.18E-07	2.5	1.02E-08	1.4	2.14E-04	1.5	1.60E-04
NND5-T	0.8	6.14E-02	0.9	3.65E-02	1.3	2.51E-02	1.3	4.80E-03	1.3	7.76E-05	1.4	1.54E-06

Log 2 fold change between leaf and root, and the associated adjusted significance value, as calculated using Cuffdiff (version 2.0.2) and HTSeq/DESeq (version 0.5.3/1.6.1)

Supplementary Table 8: Glutamate/Aspartate pathway in *N. tabacum*

UniProt Gene Name	Gene Name	BX	K326	TN90	Probe	FC-V21	BU-V5	K326	TN90
	AAT1-S	27.5	20.3	17.7	NtPMIa1g82312e2_st	4.2	3.0	4.5	2.8
	AAT1-T	15.0	7.8	8.0	NtPMIa1g43954e2_st	3.7	4.0	1.7	2.0
	AAT2-S	15.5	20.1	31.4	NtPMIa1g29974e2_st	4.8	4.6	5.0	4.5
	AAT2-T	20.7	9.7	32.7	NtPMIa1g78837e1_st	4.6	5.6	4.6	4.2
ASP5	AAT3-S	47.3	46.4	33.0	n.a.	-	-	-	-
	AAT3-T	80.6	73.6	56.6	n.a.	-	-	-	-
ASP1	AAT4-S	9.4	11.4	10.8	NtPMIa1g144258e1_st	6.7	5.9	6.7	6.6
	AAT4-T	14.1	3.7	13.2	NtPMIa1g143061e1_st	6.6	6.6	5.6	6.2
	AAT5	3.3	-	5.0	NtPMIa1g210418e2_st	3.3	3.4	2.0	2.2
	AG1-S	0.3	0.0	0.3	NtPMIa1g66485e1_st	4.6	5.0	3.0	3.6
	AG1-T	2.0	2.1	2.4	NtPMIa1g24456e1_st	2.3	3.9	1.9	3.2
	AG2-S	4.0	4.6	4.9	n.a.	-	-	-	-
	AG2-T	16.6	30.8	23.4	n.a.	-	-	-	-
	AG3-S	1.0	2.9	7.8	n.a.	-	-	-	-
	AG3-T	3.6	3.1	5.5	n.a.	-	-	-	-
ASN1	ASN1-S	21.1	15.0	8.1	NtPMIa1g180116e2_st	4.2	4.3	5.5	3.9
ASN1	ASN1-T	37.3	36.9	23.6	NtPMIa1g47375e1_st	2.4	2.6	2.2	1.6
ASN2, ASN3	ASN2-S-1	13.3	30.6	27.6	NtPMIa1g121582e1_st	1.7	2.3	2.2	1.6
ASN2, ASN3	ASN2-S-2	32.3	24.7	24.9	NtPMIa1g152336e1_st	9.3	9.2	9.0	8.0
	ASN3-S	0.0	0.0	0.0	NtPMIa1g66826e3_st	5.6	5.6	5.2	4.5
	ASN4	0.0	0.0	0.0	NtPMIa1g11250e2_st	4.7	4.5	3.7	3.4
ASN1	ASN5-S	2.6	13.3	21.2	NtPMIa1g57337e1_st	6.1	6.0	6.3	4.6
ASN1	ASN5-T	2.9	2.0	11.8	NtPMIa1g25255e1_st	4.0	4.1	5.5	4.4
GDH1	GDH1-S	7.0	5.8	5.0	n.a.	-	-	-	-
GDH1	GDH1-T	10.8	8.4	10.4	NtPMIa1g24755e2_st	5.2	4.4	4.7	3.4
	GDH2-S	29.7	40.1	29.7	NtPMIa1g71072e1_s_st	6.3	6.6	6.0	6.7
GDH2	GDH2-T	5.7	11.1	8.1	NtPMIa1g38120e2_st	5.3	5.7	5.3	5.1
	GDH3-S	0.5	0.5	1.0	NtPMIa1g174724e1_st	2.0	2.6	1.8	2.2
	GDH3-T	0.1	0.3	0.1	NtPMIa1g171947e2_st	1.8	3.0	1.8	1.8
	GDH4-S	1.8	1.6	1.1	n.a.	-	-	-	-
	GDH4-T	3.5	3.0	2.6	NtPMIa1g112486e3_x_st	4.9	5.6	4.5	3.1
	GDH5-S	10.8	7.8	7.7	NtPMIa1g68584e2_st	4.1	4.9	5.8	2.5
	GDH5-T	2.1	0.6	0.7	NtPMIa1g68584e3_st	3.9	4.6	4.2	2.8
GLN1-3	GS1-S	28.7	29.4	45.4	NtPMIa1g157146e1_st	2.4	1.9	1.3	1.5
GLN1-3	GS1-T	44.7	41.4	82.9	NtPMIa1g56120e4_st	9.8	9.9	9.7	7.8
	GS2-S	6.1	2.8	5.9	NtPMIa1g135767e2_st	10.0	10.4	9.3	8.0

	GS2-T	1.3	0.8	2.6	NtPMIa1g118995e1_st	8.5	8.6	7.8	5.7
	GS3-S	450.2	511.6	-	NtPMIa1g19275e1_st	5.3	5.9	6.3	6.6
	GS3-T	311.2	-	180.6	NtPMIa1g57439e2_st	9.1	10.3	10.4	10.6
GLN1-5	GS4-S	3.3	1.1	2.0	NtPMIa1g45720e3_st	3.1	3.4	2.8	2.3
GLN1-5	GS4-T	4.4	2.8	0.9	n.a.	-	-	-	-
	NADH-GOGAT-S	35.2	23.5	11.7	NtPMIa1g47075e5_st	6.6	6.3	5.2	5.3
	NADH-GOGAT-T	15.3	23.8	14.0	NtPMIa1g38795e1_st	5.7	4.6	4.3	3.9
	Fd-GOGAT-S	172.2	222.8	111.5	n.a.	-	-	-	-
	Fd-GOGAT-T	155.7	173.4	89.2	NtPMIa1g65200e1_st	7.0	7.2	6.3	6.7

Columns 3–5 give the average FPKM of three biological replicates in leaves.

Columns 7–10 give the measured Affymetrix log_2 expression. n.a., not available.

Supplementary Table 9: Expression of *eIF4E1* gene in tobacco leaves and roots

eIF4E1	**K326**		**TN90**		**BX**	
	**R**	**L**	**R**	**L**	**R**	**L**
eIF4E1.S	27.3	28.8	n.g.	n.g.	31.2	34.8
eIF4E1.T1	13.2	15.4	27.6	30.0	17.7	18.3
eIF4E1.T2	0.9	1.4	3.9	4.4	0.6	1.5

Average FPKM from RNA-seq triplicates.

R: root; L: leaf; n.g.: no gene.

Supplementary Table 10: DNA sequencing libraries

Species	Accessions	Insert size [bp]	Raw reads
Nicotiana tabacum TN90	SRR959464	323	792406088
Nicotiana tabacum TN90	SRR955756	464	717381770
Nicotiana tabacum TN90	SRR955758	1128	155312819
Nicotiana tabacum TN90	SRR955759	1830	22342988
Nicotiana tabacum K326	SRR955769	335	573527270
Nicotiana tabacum K326	SRR955770	492	415906446
Nicotiana tabacum K326	SRR955771	1080	285337014
Nicotiana tabacum BX	SRR955780	332	503533618
Nicotiana tabacum BX	SRR955781	485	331359850
Nicotiana tabacum BX	SRR955782	990	171634064
Nicotiana otophora	SRR955752	311	745250375
Nicotiana otophora	SRR954963	437	426769720
Nicotiana otophora	SRR954962	1121	97478456
Nicotiana otophora	SRR952787	1912	57011953
Nicotiana otophora	SRR1171700	6000	67460219

Supplementary Table 11: Mate-pair sequencing libraries from related Nicotiana species

Species	Library	Insert size [bp]	Raw reads
Nicotiana sylvestris	ERS246902, ERS246903	3000	113611130
Nicotiana sylvestris	ERS246904, ERS246905, ERS246906	5000	145579088
Nicotiana sylvestris	ERS246907, ERS246908, ERS246909	5000	96004790
Nicotiana tomentosiformis	ERS246919	3000	16935274
Nicotiana tomentosiformis	ERS246920	5000	14909440

Supplementary Table 12: Number of ``tomato and potato proteins from each chromosome mapped to tobacco linkage groups

Tobacco linkage group	Tomato/potato chromosome	Mapped tomato proteins	Mapped potato proteins
1	ch04	2	2
1	ch08	75	73
1	ch10	33	21
2	ch07	60	51
2	ch09	24	20
3	ch02	95	93
3	ch06	13	11
4	ch03	89	90
4	ch06	20	13
4	ch11	4	2
5	ch05	12	12
5	ch08	8	13
5	ch10	34	37
6	ch03	116	110
6	ch06	2	8
6	ch11	0	6
7	ch01	102	97
7	ch09	32	33
8	ch01	0	4
8	ch06	64	61
8	ch12	21	23
9	ch01	12	9
9	ch07	24	29
9	ch11	37	26
10	ch07	51	49
10	ch09	47	41
11	ch03	4	4
11	ch04	9	12
11	ch05	38	36
11	ch06	13	14
11	ch11	15	13
12	ch02	4	4
12	ch04	93	78
12	ch08	0	2
12	ch10	12	12
12	ch11	4	0

13	ch03	2	2
13	ch04	20	11
13	ch05	43	44
13	ch11	13	9
14	ch01	22	12
14	ch03	19	19
14	ch09	19	19
15	ch04	4	0
15	ch07	6	6
15	ch08	45	53
15	ch10	6	10
15	ch12	34	40
16	ch02	4	0
16	ch04	100	82
16	ch11	8	13
17	ch02	126	118
17	ch06	11	10
18	ch01	11	8
18	ch07	49	43
18	ch10	2	2
18	ch11	34	27
19	ch01	93	91
19	ch09	32	27
19	ch12	2	2
20	ch05	25	22
20	ch12	43	37
21	ch03	18	16
21	ch06	6	6
21	ch12	60	64
22	ch01	19	15
22	ch06	68	79
22	ch09	36	37
22	ch11	4	0
22	ch12	2	0
23	ch04	28	28
23	ch08	107	108
23	ch10	4	2
24	ch05	35	45
24	ch10	45	35
24	ch12	0	6

Supplementary Table 13: PCR primers pairs used in *eIF4* gene family diagnostic

Gene target		PCR primers (5' - 3')	Product size (bp)
Nsyl-eIF4E1	*Forward*	AATGCTTATTGTTAGCCTTTGTTTCT	402
	Reverse	GTCAAGTGGCAGCCTTTCATA	
Nsyl-eIF4E2	*Forward*	TGCAGGTGAGCATTGGTAAA	268
	Reverse	AGCCACAGGTGACTGCTTCT	
Nsyl-eIF(iso)4E	*Forward*	TCCAATTTGCCTAAGCCTTG	394
	Reverse	AACAAACCCAGTATAATCCCACAT	
Ntom-eIF4E2	*Forward*	TGTTGCTGACAATTGGTGCT	301
	Reverse	CATCATGCTCGGGAAAGG	
Ntom-eIF4E1 gene1	*Forward*	TGTTGTTGTTGTTGTTGTTGC	565
	Reverse	TGATTCCTAGACAAGGTGGTG	
Ntom-eIF4E1 gene2	*Forward*	AGGAAATATGCCTATGTATGAAGCAA	285
	Reverse	AAGAATTGCAAAGACTTTTTGAACC	
Ntom-eIF(iso)4E	*Forward*	TTCTCGTGGTGTCAAATAGTATCTG	248
	Reverse	TCCATCCATGGGTCACAAT	

Supplementary Note 1: Alkaloid pathway

In Supplementary Table 6, the number of gene copies involved in the synthesis of nicotine (Supplementary Figure 4A) is presented, together with their affiliation to the genome of the ancestors *N. tomentosiformis* (T) and *N. sylvestris* (S) and RNA-seq data (mean of three replicates in root and leaf). On the right, transcript data were also compared with the root and leaf of BU-V5 and FC-V21, based on specific probes, exactly as reported by Martin *et al*[1]. Investigations of CNVs in the alkaloid pathways showed that, in general, the number of gene isoforms in the three *N. tabacum* varieties corresponds to the sum of genes inherited from the ancestors *N. tomentosiformis* (T) or *N. sylvestris* (S)[2].

For instance, seven *BBL* gene copies (three T and four S) were found in the tobacco genome. Phylogenetic diagnostics show that these can be structured as three pairs of gene isoforms, plus an additional duplicated gene for *BBL2*, namely *BBL2-S-2*. Interestingly, Flue-cured tobacco (K326) most likely lost two *QPT* copies originating from *N. sylvestris*, which may affect the oxidative deamination of N-methylputrescine in K326. In addition, we found that a fifth *PMT* gene exists in tobacco which is possibly a duplication of *PMT4*[3]. For the sake of clarity, we did not change the numbering of PMTs, but added their ancestral origin (S or T) to the former Uniprot annotation. The existence of five *PMTs* is in accordance with the identification of two and three genes in the *N. tomentosiformis* and *N. sylvestris* ancestors, respectively[2]. Although *PMT2* sequences identified in the three BX, K326, and TN90 *N. tabacum* genomes were only partial, sequencing data from other tobacco varieties (data not shown) and RNA-seq data clearly confirmed that *PMT2* can be annotated as a full sequence expressed in the roots of these three tobacco varieties (Supplementary Table 6), as published by Biastoff *et al*[3].

Concerning *CYP82E* sequences, five S copies and two T copies were clearly identified in *N. tabacum*, although the last one (*NND1*) was difficult to assign to a definite S or T ancestor. Nevertheless, eight *CYP82E* copies were found in total within these three tobacco accessions, which is in accordance with Sierro *et al*[2].. Interestingly, phylogenetic comparisons indicate that *CYP82E10* branched with *CYP82E5v2* as corresponding homoeologous S and T tobacco ancestors. In our data set, only one E4 isoform corresponding to *CYP82E4v2* was found in the BX, K326, and TN90 *N. tabacum* genome, with *CYP82E4v1* not represented as a distinct loci. We postulate that *CYP82E4v2* is a unique gene and that *CYP82E4v1* was artificially generated by changes introduced by PCR primer sequences, as described in Siminszky *et al*[4].

As nicotine is synthesized in the roots and then translocated to the upper part of the leaves where it functions as an antiherbivore chemical, most of the gene accessions linked to the alkaloid pathway have to be actively expressed within the roots[5]. Based on RNA-seq data (Supplementary Table 6 and Supplementary Table 7), we confirmed that most of the genes are transcribed in the roots, except for *BBL3.S* and *BBL3.T*, *MPO2.S*, and *NND4-T*. In our experimental conditions, *BBL3.S*, *BBL3.T*, and *MPO2.S* were weakly or not at all expressed in the leaf, suggesting that they were either expressed in other tissues or present as pseudogenes. However, it is not surprising to find a lack of E4 gene expression in green leaves and roots, since *NND4-T* is specifically and strongly up-regulated during leaf senescence[6, 7].

We observed that *PMTs, A622,* and most of the *BBLs* are exclusively expressed within the roots, so appear to be mainly devoted to alkaloid biosynthesis and regulated by *NIC* loci[8]. Nevertheless, some transcripts from *AO*, *QPT, QS*, and *MPO* genes were also detected in leaf, suggesting the presence of metabolic functions

different from alkaloid synthesis. Confirming this hypothesis, Macho *et al*.[9] recently reported the presence of a chloroplastic AO (*FIN4*) catalyzing an irreversible step in the *de novo* biosynthesis of *nicotinamide adenine dinucleotide* (NAD). In addition, Shoji and Hashimoto[10] and Ryan *et al*.[11] reported the expression of *QPT1* and *QPT2* within leaf tissues, although did not reveal their possible functions. The product of AO activity, α-iminosuccinate, is condensed with glyceraldehyde-3-phosphate and cyclized to produce quinolinic acid by quinolinate synthase (QS). Therefore, it is not surprising that *QS* is expressed in leaves since it is needed by other metabolic activities for the *de novo* synthesis of NAD from Asp[12]. Interestingly, the *Arabidopsis* QS mutant *old5* exhibits early developmental senescence triggered by cellular redox reactions[13]. *MPOs* are known to be less tightly regulated by *NIC* loci than the above-mentioned genes[14]. They belong to a subclass of diamine oxidases reported to exhibit activities in tobacco leaf[15]. This subclass may also include the products of the genes *MPO3.S* and *MPO3.T*, which do not contribute to the oxidative deamination of N-methylputrescine as this chemical compound is not abundant in tobacco leaf[16]. According to our data, *CYP82E5v2* and *CYP82E10* expression occurs in both green leaves and roots, although preferentially in the latter, and is not confined to one tissue type[17, 18]. This is not surprising since both genes originated from *N. tomentosiformis* and *N. sylvestris* (see above).

The observed RNA-seq expression is supported by gene-specific probes in Affymetrix expression profiles of a Burley (BU-V5) and a Flue-cured variety of tobacco (FC-V21) under different growth conditions[1]. This observation attests to the conservation of transcripts involved in the alkaloid biosynthesis pathway, although elements of the pathway are sensitive to the stress response. Notably, no Affymetrix probes were identified for *QPT1.S* and *QPT2.S* because probe sequences were

designed for TGI sequences from Hicks Broadleaf, a Flue-cured tobacco genetically similar to K326, in which the corresponding sequence could not be identified (see above text).

Supplementary Note 2: Glutamate/aspartate pathway

Tobacco types differ with respect to their nitrogen fertilizer needs for growth and yield (biomass per unit of applied nitrogen). Thus, Burley tobacco requires more nitrogen fertilizer than Flue-cured or Oriental tobacco. Consequently, Flue-cured tobaccos are considered low-biomass cultivars compared with Burley[19] and contain less total nitrogen, nitrate nitrogen, total alkaloids, free amino acids, and some protein components[20]. With the exception of proline, which varies between tobacco varieties and is dependent on drought and irrigation treatments[21], glutamate (Glu) and aspartate (Asp) are the amino acids that differ the most in green leaves between Burley and Flue-cured[22]. We repeated the amino acid analyses in TN90, K326, and BX grown under typical agricultural practices for each variety. Total free amino acids in green leaves were higher in Burley (TN90: 10.4 ± 4.2 mg/g, N=5) than in Flue-cured (K326: 2.8 ± 0.8 mg/g, N=5) and Oriental (BX: 4.6 ± 0.9 mg/g, N=5) (Supplementary Figure 6), and Burley tobacco had a higher content of glutamate (Glu), glutamine (Gln), and aspartate (Asp). The reason for the strong nitrogen requirement of Burley tobacco is unknown, but may derive from acquired genetic variations[23], which could affect key enzymes of the nitrate assimilation pathway as well as other routes involved in nitrate uptake, translocation, and storage within the tobacco plants.

Amino acid synthesis requires nitrogen assimilation within a carbon skeleton form, ammonium being the nitrogen donor and α-ketoglutarate the carbon acceptor. This essential step is achieved via a key metabolic activity starting within the chloroplast, the GS/GOGAT pathway (Supplementary Figure 4B), which produces glutamate and glutamine[24]. For further transport, storage and conversion to other amino acids, additional steps are catalyzed by asparagine synthetase (AS) and aspartate amino transferase (AAT). This ensures the synthesis of asparagine and

aspartate, with asparaginase (AG) and glutamate dehydrogenase (GDH) functioning as asparagine and glutamate catabolic enzymes, respectively. In Supplementary Table 8, the potential series of tobacco genes encoding enzymes involved in the nitrogen assimilation into amino acids is shown together with gene isoforms and their affiliation to the genome of *N. tomentosiformis* (T) and *N. sylvestris* (S) ancestors, leaf RNA-seq data (mean of three replicates), and Affymetrix data from the pairs BU-V5–FC-V21 and TN90–K326 cultivated in the greenhouse[1]. Oriental tobacco was not included in the Affymetrix data set or alkaloid analyses because its field growing conditions are difficult to mimic in the greenhouse.

The GS tobacco sub-family is composed of four pairs of genes that originated from *N. tomentosiformis* (T) and *N. sylvestris* (S) ancestors. The cytosolic isoforms (GS1) of these, corresponding to Gln1-3 and Gln1-5, are known as GS1.T-GS1.S and GS4.T-GS4.S, respectively. Phylogenetic diagnostics reveal that GS2.S and GS2.T are also cytosolic isoforms, whereas GS3.S and GS3.T are chloroplastic isoforms. Interestingly, the eight GS isoforms are present in the three tobacco varieties of this study and are expressed at similar levels in leaf tissues. The chloroplastic isoforms GS2 and Fd-GOGAT[25] have major roles in the GS/GOGAT pathway, while GS1 and NADH-GOGAT are the cytosolic isoforms of glutamine synthetase and glutamate synthase, respectively. Gene expression of the former is notably influenced by plant growth conditions and sampling time.

Similarly, *GS1.S* is poorly expressed in fully expanded green leaves (Affymetrix data) compared with *GS1.T*, whereas both genes are expressed at comparable levels in the leaves of young axenically grown plants (see RNA-seq data, Supplementary Table 8). Conversely, *GS2.S* and *GS2.T* are strongly expressed in fully expanded leaves (Affymetrix data), but are weakly expressed in *in vitro* cultivated

plants (RNA-seq data), indicating that *GS* isoforms are differentially expressed in plants at different growing stages. *GS4.S* and *GS4-T*, which correspond to *Gln1-5*, are strongly expressed in senescent leaf tissues[25], but weakly expressed in green leaves (Supplementary Table 8). *GS3.S* and *GS3.T* are strongly expressed in leaf tissue in accordance with their chloroplastic localization (as predicted by WoLF PSORT[26]), although they were identified as gene fragments in K326 and TN90.

Glutamate dehydrogenase (GDH) is a ubiquitous enzyme that catalyzes the reversible amination of 2-oxoglutarate to glutamate. It is composed of α and β subunits randomly associated within complex isoenzyme profiles[27]. Eight *GDH* subunit gene pairs were identified in the tobacco genome. With the exception of *GDH2.S*, most GDH isoforms are not strongly expressed in leaves. This supports the function of GDH in plant cells, which is glutamate deamination within the dark[28] and in root tissues[29].

Asparagine (ASN) is the major transport compound in plants, particularly for nitrogen recycling from source to sink leaves[30]. One major site for asparagine synthetase activity is apparently close to vascular tissues (phloem companion cell-sieve element complex) where *ASN*, *GS1*, and *GOGAT* genes are co-expressed[31]. In *Arabidopsis*, three ASN genes have been clearly identified: *ASN1*, *ASN2*, and *ASN3*. This compares with eight in tobacco: (1) two pairs similar to *ASN1*, *ASN1-S* and *ASN1-T*, and *ASN5-S* and *ASN5-T*, (2) *ASN2-S-1* and *ASN2-S-2* genes from the same tobacco ancestor and similar to both *ASN2* and *ASN3*, and (3) *ASN3-S* and *ASN4* genes that do not appear to be affiliated with a corresponding gene in *N tomentosiformis* or *N. sylvestris*. All these genes are expressed in leaves but with different levels of intensity. Asparaginase (AG) catalyzes the hydrolysis of asparagine to yield aspartate and ammonia[30]. Very limited AG activities were detected in leaves

of both the *in vitro* cultivated plant leaves (RNA-seq data) and mature green leaves (Affymetrix data), with the exception of *AG2-T* in the *in vitro* cultivated plant leaves. This is to be expected since the transcript levels of the two closest asparaginase genes from *Arabidopsis*, *ASPGA1* and *ASPGB1*, are highest in sink tissues, especially in early developing flowers and siliques, but not in source leaves[32]. Genetic comparison reveals that the *AG3* genes are similar to *ASPGB1*, and that *AG1* and *AG2* genes are similar to *ASPGA1* of *Arabidopsis*.

Aspartate aminotransferase (AAT) plays a crucial role in the metabolic regulation of carbon and nitrogen metabolism in all organisms. In eukaryotes, it is involved in the interchange of carbon and nitrogen pools between subcellular compartments. The reloading of the aspartate pool is essential to ensure asparagine production and nitrogen remobilization via ASN[33]. Four S and T gene pairs (*AAT1–AAT4*) and one single gene (*AAT5*) belong to the AAT gene family, although the *AAT5* isoform does not appear to be present in K326. By analogy with *Arabidopsis thaliana*, the cellular localization of the resulting tobacco AAT isoenzymes are likely to be in the cytosol (AAT1, AAT2), chloroplast (AAT3) or mitochondria (AAT4, AAT5)[34].

Our dataset suggests that glutamate/aspartate pathway is close between Burley and Flue-cured tobacco, since no additional gene copies of the key players GS and GOGAT were found in Burley compared with Flue-cured tobacco, and because gene expression profiles were comparable between both tobacco varieties. This is also true for the four other gene families involved in the GS/GOGAT cycle, namely *AAT*, *AG*, *ASN*, and *GDH*, with the exception of the *AAT5* isoform that is absent from K326. As such, gene expression does not differ within the full GS/GOGAT pathway, the origin

of the high nitrogen demand of Burley tobacco does not seem to be linked to gene alteration within the GS/GOGAT pathway, with the exception of the *AAT5* gene.

Supplementary Note 3: Disease resistance

Because the symptoms of TVMV, TEV, and PVY are very similar, it is usually impossible to distinguish between these diseases without performing serological assays[35]. The first tobacco varieties developed with partial resistance conferred by the single recessive factor *va* assigned to chromosome E[36, 37] were, however, susceptible to other disease because of the lack of trichome exudates[38]. "TN86" was the first Burley variety to produce normal trichome secretions and not to exhibit the insect susceptibility normally seen in PVY-resistant breeding lines registered in the US[39]. Some years later, a sister line of TN86 sharing the same resistance pattern for TVMV, TEV, and PVY was developed and released under the variety name "TN90" by Miller[40].

Supplementary Note 4: Historical description of tobacco varieties

K326 (TC 319 or PI 552505) is a Flue-cured tobacco developed by Novartis Seeds, Inc., that was registered in the US Plant Variety Protection Office under the certificate number 008300070 in 1983. It is known for its high quality and ease of processing, and remains one of the most important Flue-cured tobaccos worldwide. However, it has only a low level of resistance to black shank and Granville wilt, is susceptible to mosaic, but demonstrates resistance to root-knot nematodes.

TN90 (TC 586 or PI 543792) is one of the most successful Burley tobacco varieties grown worldwide. It was developed by R. D. Miller at the University of Tennessee and was registered in 1990. Its success was due to a high level of resistance to several diseases such as TMV, TVMV, PVY, *Thielaviopsis basicola*, and *Pseudomonas tabaci*, and it is moderately resistant to tobacco etch potyvirus (TEV) and races 0 and 1 of *Phytophthora nicotianae* var. *parasitica*.

Basma Xanthi (inventory number BX 2a) is an Oriental tobacco from Xanthi in northeastern Greece. It is thought to be a descendent of old local varieties following hybridization and breeding. It is actively used in plant research and molecular farming.

SUPPLEMENTARY REFERENCES

1. Martin F, *et al.* Design of a Tobacco Exon Array with application to investigate the differential cadmium accumulation property in two tobacco varieties. *BMC Genomics* **13**, 674 (2012).

2. Sierro N, *et al.* Reference genomes and transcriptomes of *Nicotiana sylvestris* and *Nicotiana tomentosiformis*. *Genome Biology* **14**, R60 (2013).

3. Biastoff S, Brandt W, Drager B. Putrescine N-methyltransferase--the start for alkaloids. *Phytochemistry* **70**, 1708-1718 (2009).

4. Siminszky B, Gavilano L, Bowen SW, Dewey RE. Conversion of nicotine to nornicotine in Nicotiana tabacum is mediated by CYP82E4, a cytochrome P450 monooxygenase. *Proc Natl Acad Sci U S A* **102**, 14919-14924 (2005).

5. Baldwin IT. Damage-induced alkaloids in tobacco: pot-bound plants are not inducible. *Journal of chemical ecology* **14**, 1113-1120 (1988).

6. Chakrabarti M, Bowen SW, Coleman NP, Meekins KM, Dewey RE, Siminszky B. CYP82E4-mediated nicotine to nornicotine conversion in tobacco is regulated by a senescence-specific signaling pathway. *Plant Mol Biol* **66**, 415-427 (2008).

7. Gavilano LB, Siminszky B. Isolation and characterization of the cytochrome P450 gene CYP82E5v2 that mediates nicotine to nornicotine conversion in the green leaves of tobacco. *Plant and Cell Physiology* **48**, 1567-1574 (2007).

8. Shoji T, Kajikawa M, Hashimoto T. Clustered transcription factor genes regulate nicotine biosynthesis in tobacco. *The Plant Cell Online* **22**, 3390-3409 (2010).

9. Macho AP, Boutrot F, Rathjen JP, Zipfel C. Aspartate oxidase plays an important role in Arabidopsis stomatal immunity. *Plant Physiol* **159**, 1845-1856 (2012).

10. Shoji T, Hashimoto T. Recruitment of a duplicated primary metabolism gene into the nicotine biosynthesis regulon in tobacco. *Plant J* **67**, 949-959 (2011).

11. Ryan SM, Cane KA, DeBoer KD, Sinclair SJ, Brimblecombe R, Hamill JD. Structure and expression of the quinolinate phosphoribosyltransferase (QPT) gene family in Nicotiana. *Plant Sci* **188-189**, 102-110 (2012).

12. Katoh A, Uenohara K, Akita M, Hashimoto T. Early steps in the biosynthesis of NAD in Arabidopsis start with aspartate and occur in the plastid. *Plant physiology* **141**, 851-857 (2006).

13. Schippers JH, Nunes-Nesi A, Apetrei R, Hille J, Fernie AR, Dijkwel PP. The Arabidopsis onset of leaf death5 mutation of quinolinate synthase affects nicotinamide adenine dinucleotide biosynthesis and causes early ageing. *Plant Cell* **20**, 2909-2925 (2008).

14. Shoji T, Hashimoto T. Why does anatabine, but not nicotine, accumulate in jasmonate-elicited cultured tobacco BY-2 cells? *Plant Cell Physiol* **49**, 1209-1216 (2008).

15. Gemperlova L, Novakova M, Vankova R, Eder J, Cvikrova M. Diurnal changes in polyamine content, arginine and ornithine decarboxylase, and diamine oxidase in tobacco leaves. *J Exp Bot* **57**, 1413-1421 (2006).

16. Sato F, *et al.* Metabolic engineering of plant alkaloid biosynthesis. *Proc Natl Acad Sci U S A* **98**, 367-372 (2001).

17. Gavilano LB, Coleman NP, Bowen SW, Siminszky B. Functional analysis of nicotine demethylase genes reveals insights into the evolution of modern tobacco. *J Biol Chem* **282**, 249-256 (2007).

18. Lewis RS, Bowen SW, Keogh MR, Dewey RE. Three nicotine demethylase genes mediate nornicotine biosynthesis in Nicotiana tabacum L.: Functional characterization of the CYP82E10 gene. *Phytochemistry* **71**, 1988-1998 (2010).

19. Davis DL, Nielsen MT. *Tobacco : Production, Chemistry and Technology*. Blackwell Science Ldt (1999).

20. Lowe R, Sheen S. Accumulation of Soluble Proteins and Nitrogenous Compounds in the Leaf of Bright and Burley Tobaccos during the Growing Season *Beitrige zur Tabakforschung International* **11**, 161-169 (1982).

21. Yoshiba Y, Kiyosue T, Nakashima K, Yamaguchi-Shinozaki K, Shinozaki K. Regulation of levels of proline as an osmolyte in plants under water stress. *Plant Cell Physiol* **38**, 1095-1102 (1997).

22. Weybrew J. Changes in free amino acids of tobacco during curing. In: *Proceedings of the Fourth International Tobacco Scientific Congress*) (1966).

23. Fricano A, *et al.* Molecular diversity, population structure, and linkage disequilibrium in a worldwide collection of tobacco (Nicotiana tabacum L.) germplasm. *BMC Genet* **13**, 18 (2012).

24. Hodges M. Enzyme redundancy and the importance of 2-oxoglutarate in plant ammonium assimilation. *J Exp Bot* **53**, 905-916 (2002).

25. Masclaux-Daubresse C, Daniel-Vedele F, Dechorgnat J, Chardon F, Gaufichon L, Suzuki A. Nitrogen uptake, assimilation and remobilization in plants: challenges for sustainable and productive agriculture. *Annals of Botany* **105**, 1141-1157 (2010).

26. Horton P, *et al.* WoLF PSORT: protein localization predictor. *Nucleic acids research* **35**, W585-W587 (2007).

27. Watanabe M, Yumi O, Itoh Y, Yasuda K, Kamachi K, Ratcliffe RG. Deamination role of inducible glutamate dehydrogenase isoenzyme 7 in Brassica napus leaf protoplasts. *Phytochemistry* **72**, 587-593 (2011).

28. Miyashita Y, Good AG. Glutamate deamination by glutamate dehydrogenase plays a central role in amino acid catabolism in plants. *Plant Signal Behav* **3**, 842-843 (2008).

29. Fontaine JX, *et al.* Further insights into the isoenzyme composition and activity of glutamate dehydrogenase in Arabidopsis thaliana. *Plant Signal Behav* **8**, (2013).

30. Lea PJ, Sodek L, Parry MAJ, Shewry PR, Halford NG. Asparagine in plants. *Ann Appl Biol* **150**, 1–26 (2007).

31. Potel F, *et al.* Assimilation of excess ammonium into amino acids and nitrogen translocation in Arabidopsis thaliana--roles of glutamate synthases and carbamoylphosphate synthetase in leaves. *FEBS J* **276**, 4061-4076 (2009).

32. Ivanov A, *et al.* Arabidopsis mutants lacking asparaginases develop normally but exhibit enhanced root inhibition by exogenous asparagine. *Amino Acids* **42**, 2307-2318 (2012).

33. de la Torre F, Suarez MF, Santis L, Canovas FM. The aspartate aminotransferase family in conifers: biochemical analysis of a prokaryotic-type enzyme from maritime pine. *Tree Physiol* **27**, 1283-1291 (2007).

34. Miesak BH, Coruzzi GM. Molecular and physiological analysis of Arabidopsis mutants defective in cytosolic or chloroplastic aspartate aminotransferase. *Plant Physiol* **129**, 650-660 (2002).

35. Gooding GVJ, Lapp NA. Distribution, incidence and strains of potato virus Y in North Carolina. *Tob Sci* **24**, 89-92 (1980).

36. Gupton CL, Burk LG. Location of the Factor for Resistance to Potato Virus Y in Tobacco. *Journal of Heredity* **64**, 289-290 (1973).

37. Yamamoto Y. Studies on breeding tobacco varieties resistant to veinal necrosis disease by potato virus Y strain T. *Bull Leaf Tobacco Res Lab* **2**, 1-85 (1992).

38. Nielsen MT, Jones GA, Collins GB. Inheritance Pattern for Secreting and Nonsecreting Glandular Trichomes in Tobacco. *Crop Science* **22**, 1051-1053 (1982).

39. Miller RD. Registration of 'TN 86' Burley Tobacco. *Crop Science* **27**, 365-366 (1987).

40. Miller RD. Registration of 'TN 90' Burley Tobacco. *Crop Science* **31**, 852-852 (1991).
