# Supplementary material for: The tobacco genome sequence and its comparison with those of tomato and potato
Source: Nat Commun. 2014 May 8;5:3833. doi: 10.1038/ncomms4833 (PMC4024737; doi:10.1038/ncomms4833)

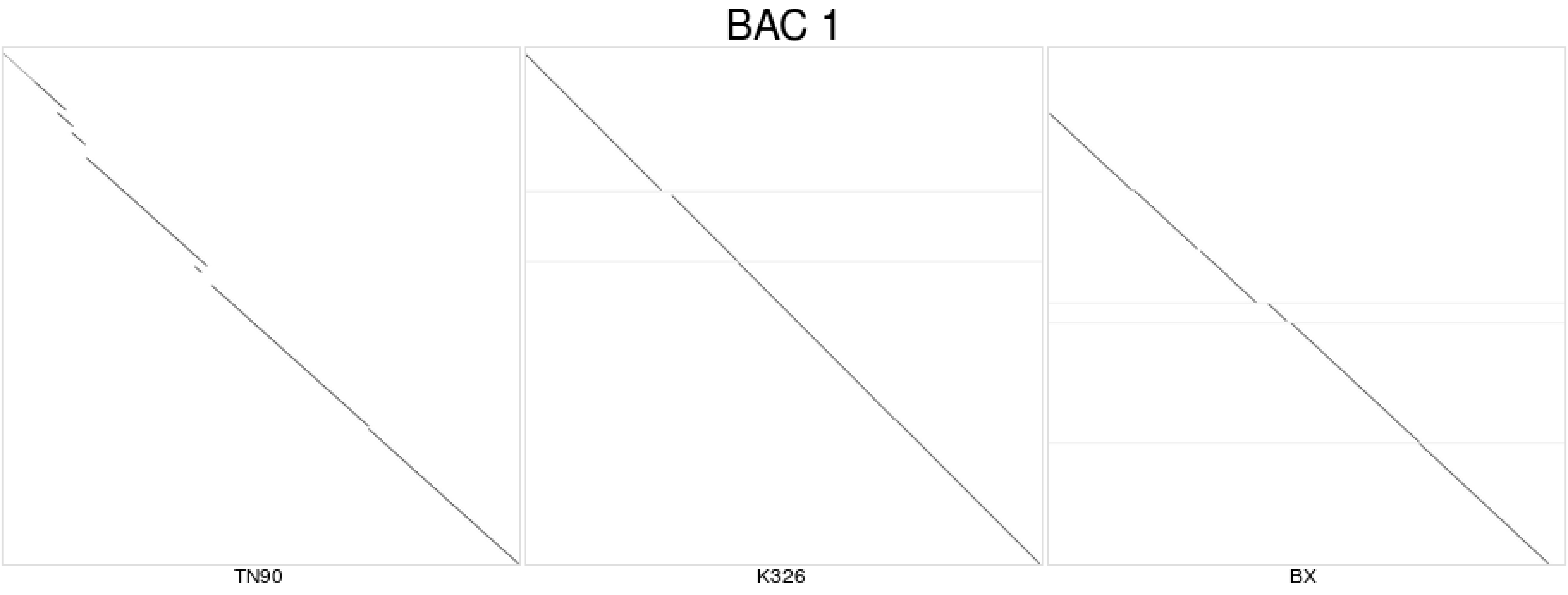
BAC 1
TN90
K326
BX

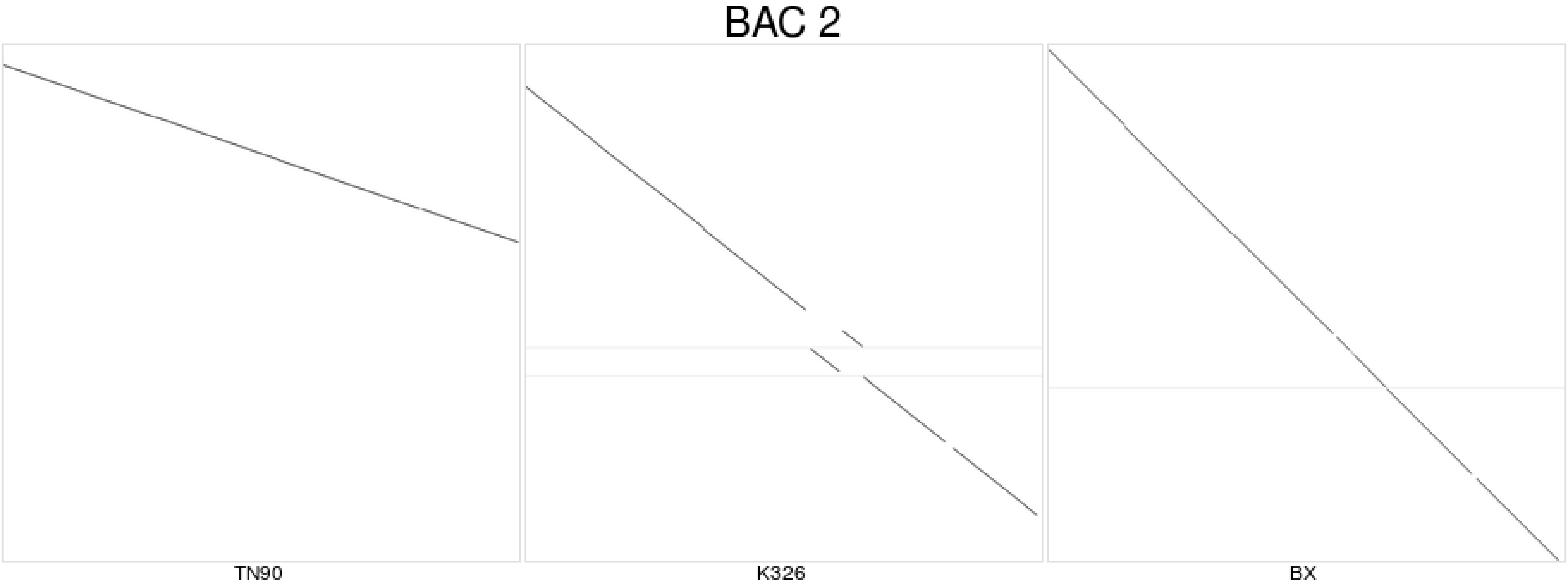

BAC 2
TN90
K326
BX

BAC 3

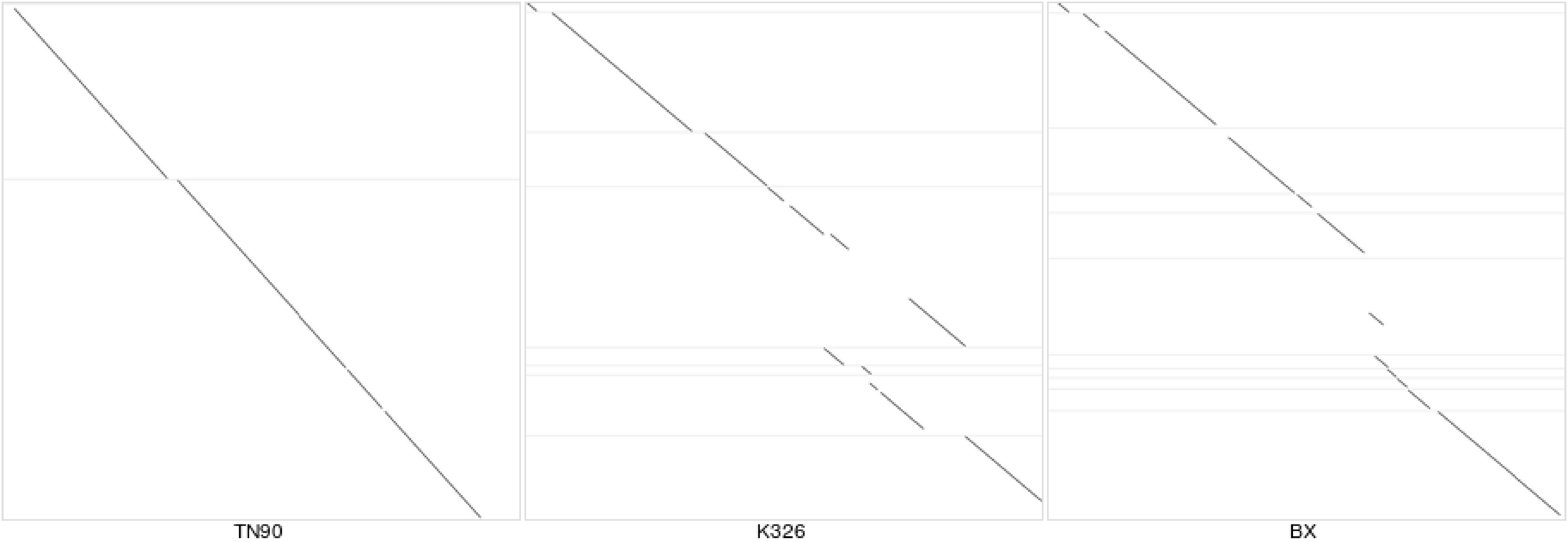

# BAC 4

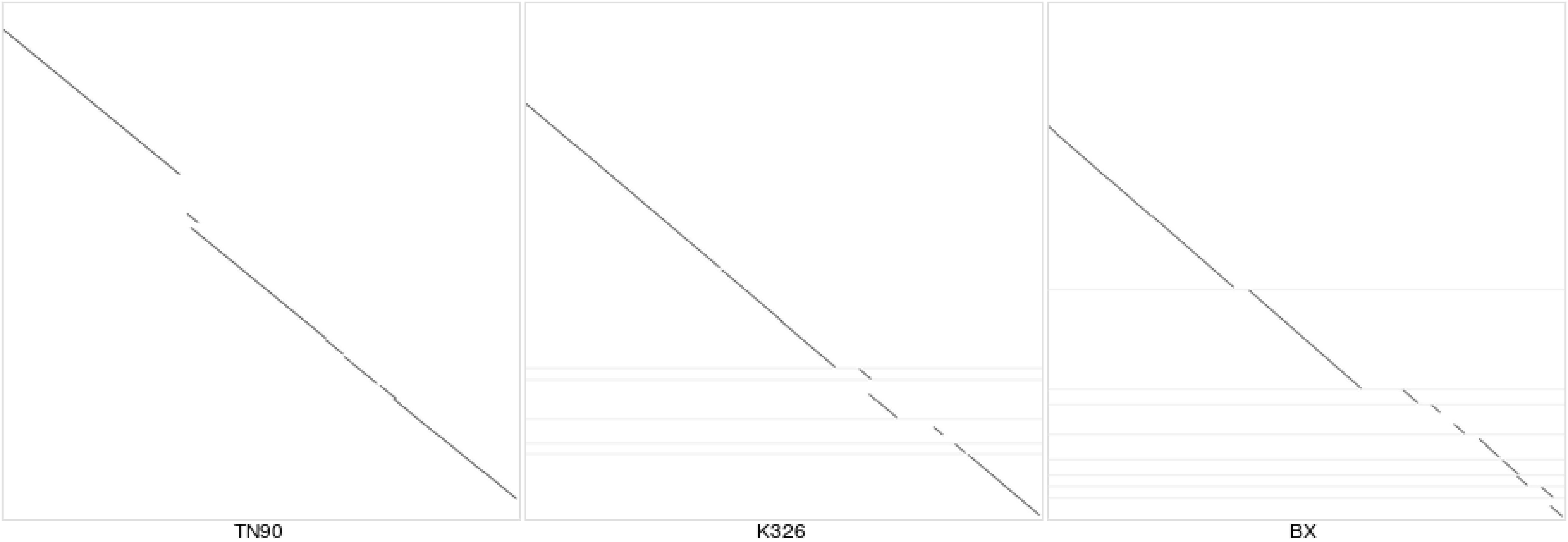

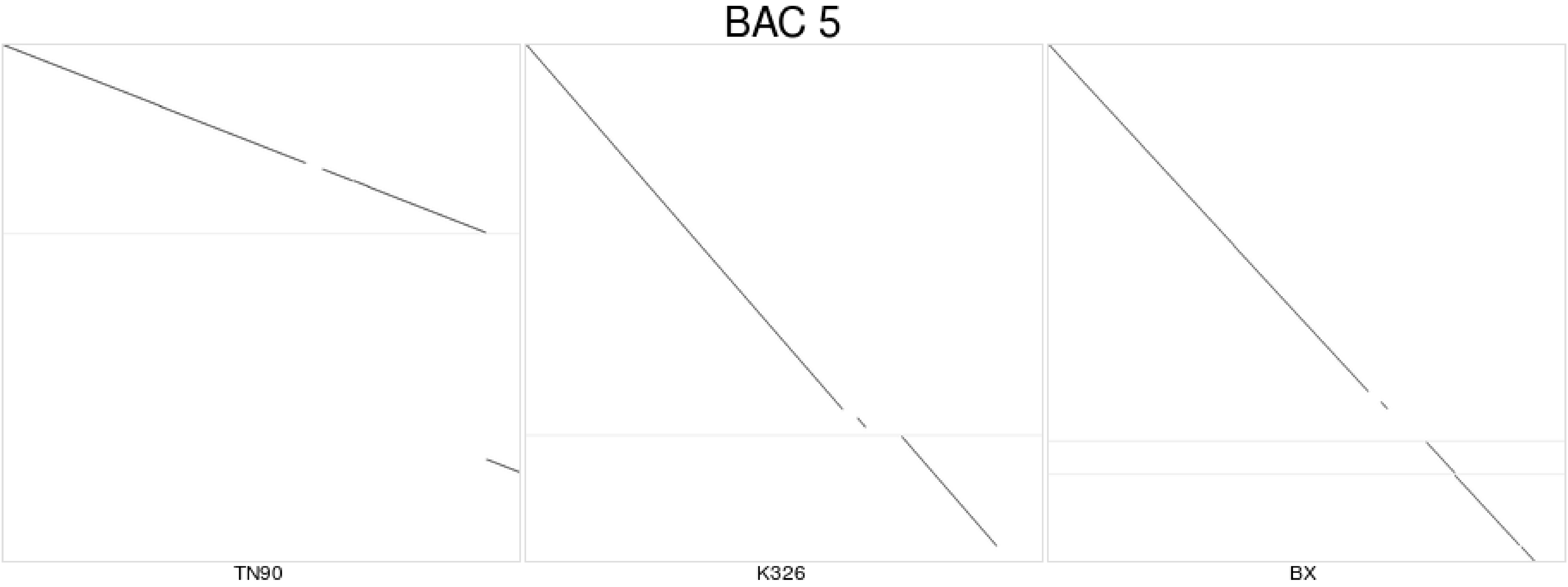

BAC 5
TN90
K326
BX

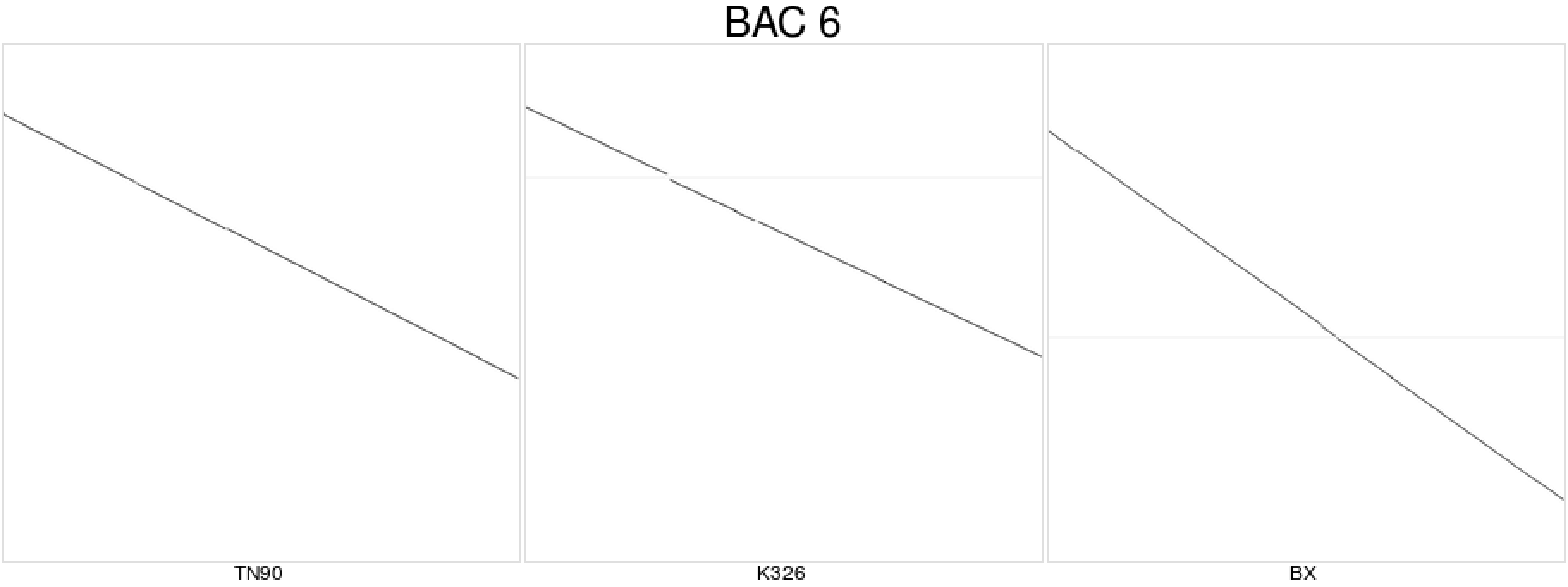

BAC 6
TN90
K326
BX

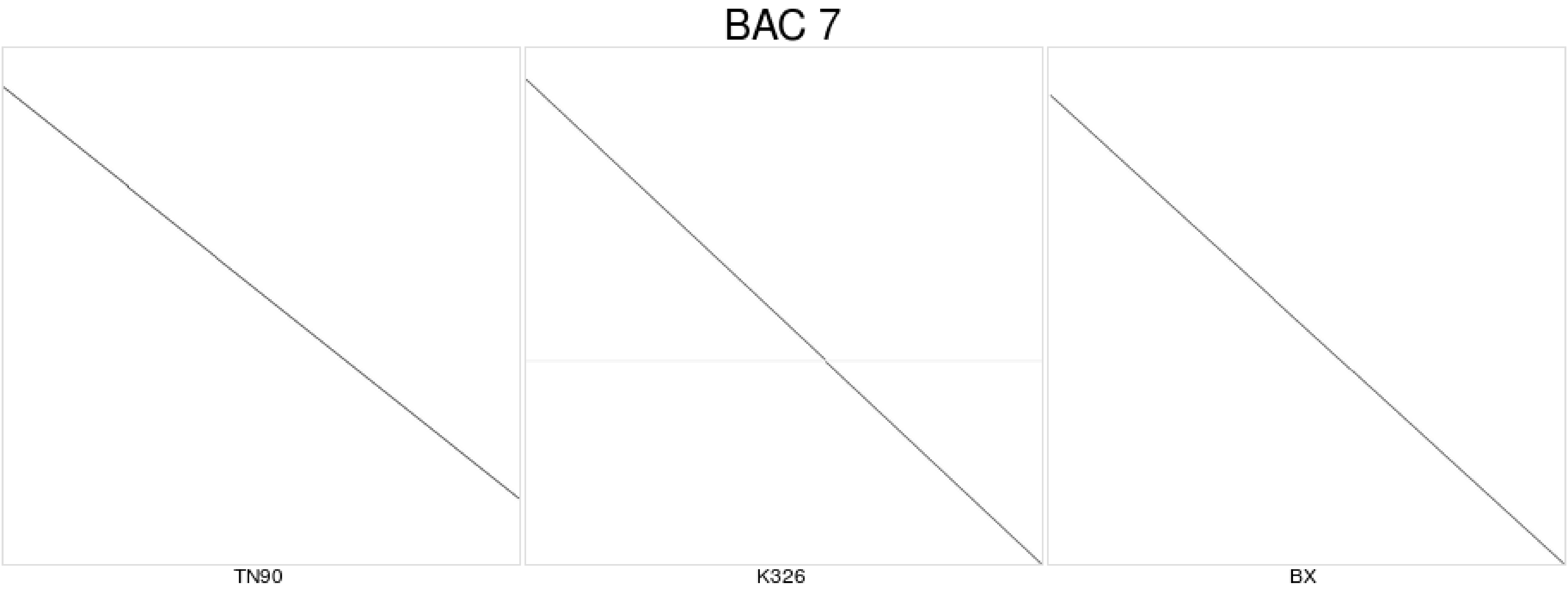
BAC 7
TN90
K326
BX

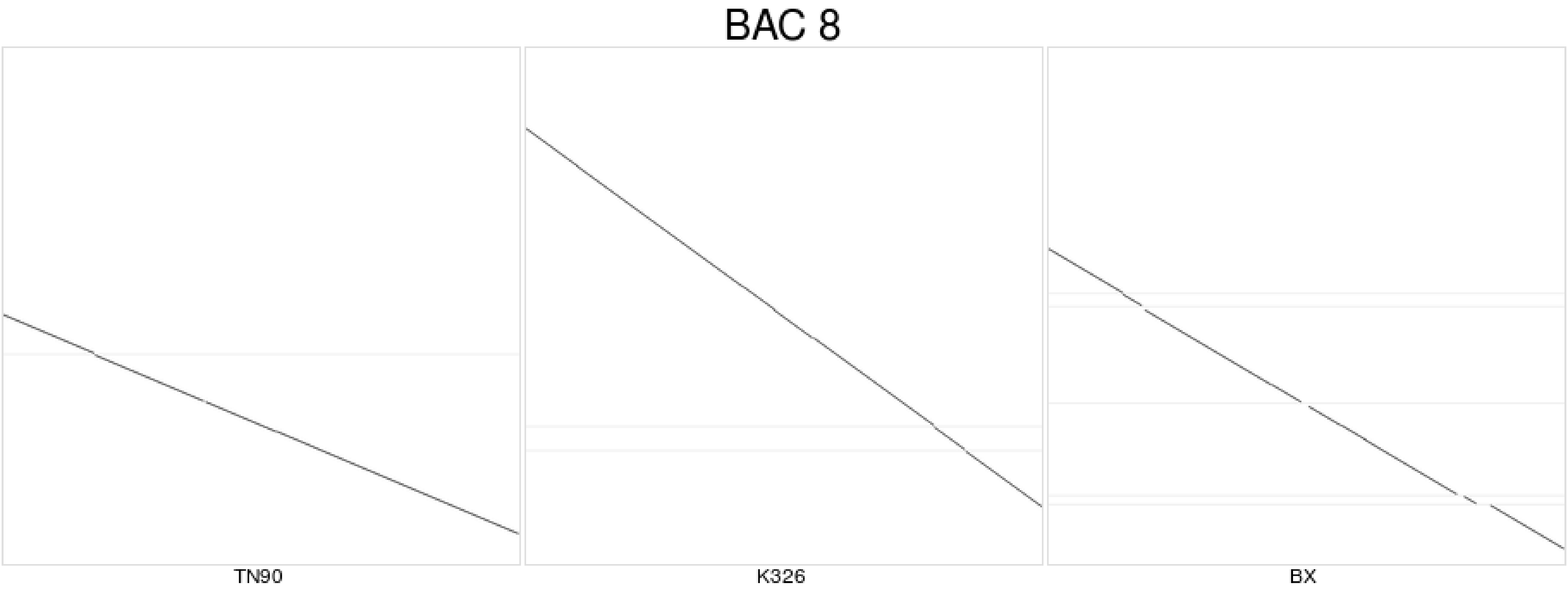
BAC 8
TN90
K326
BX

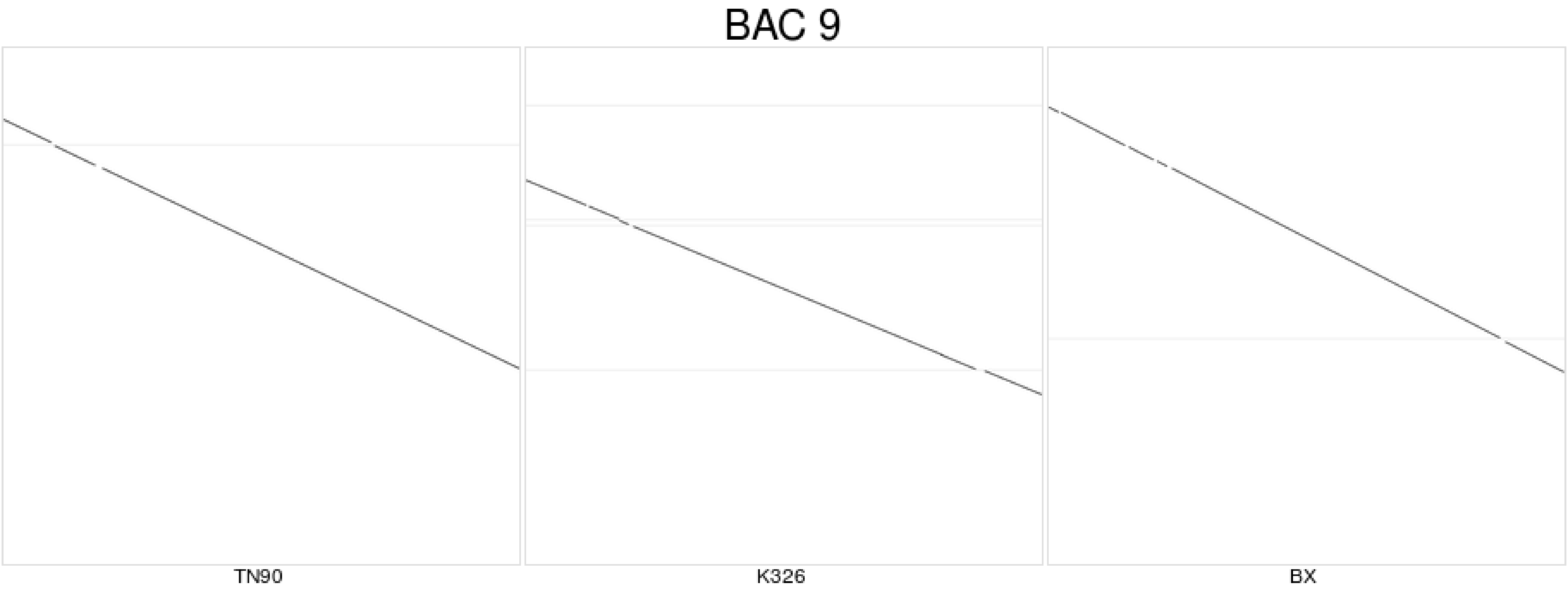
BAC 9
TN90
K326
BX

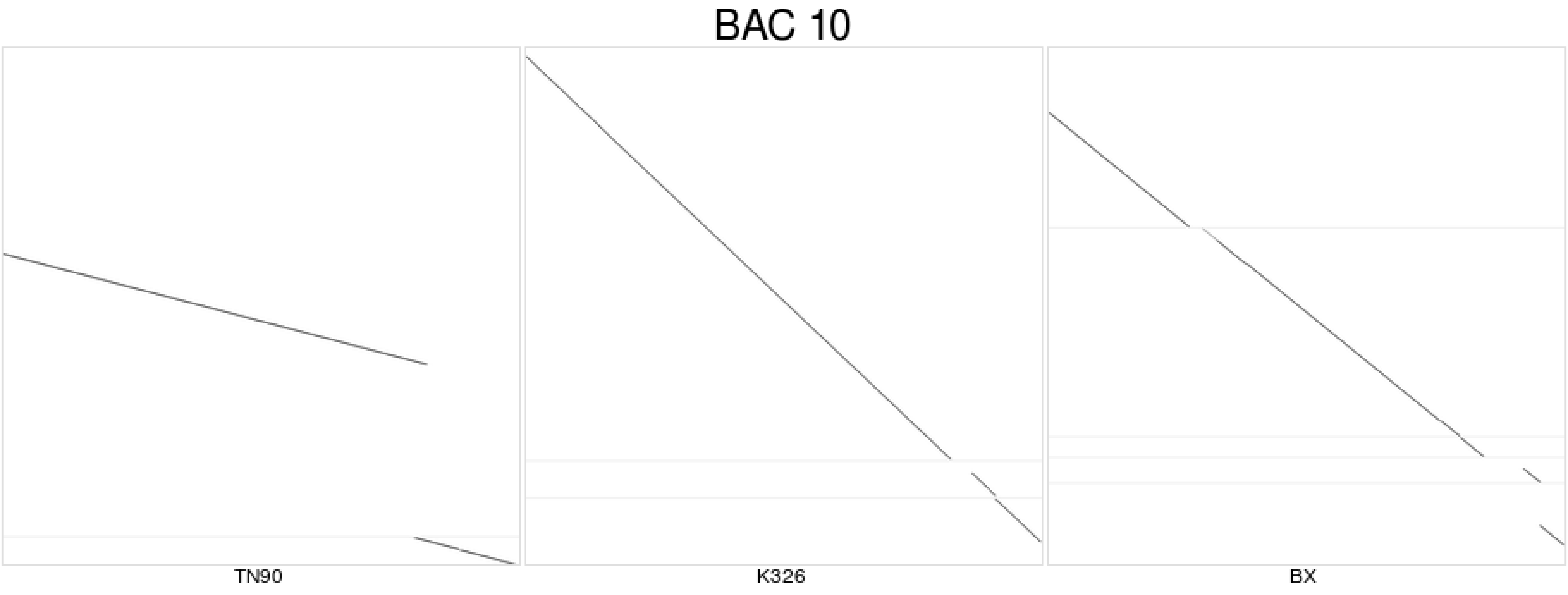
BAC 10
TN90
K326
BX

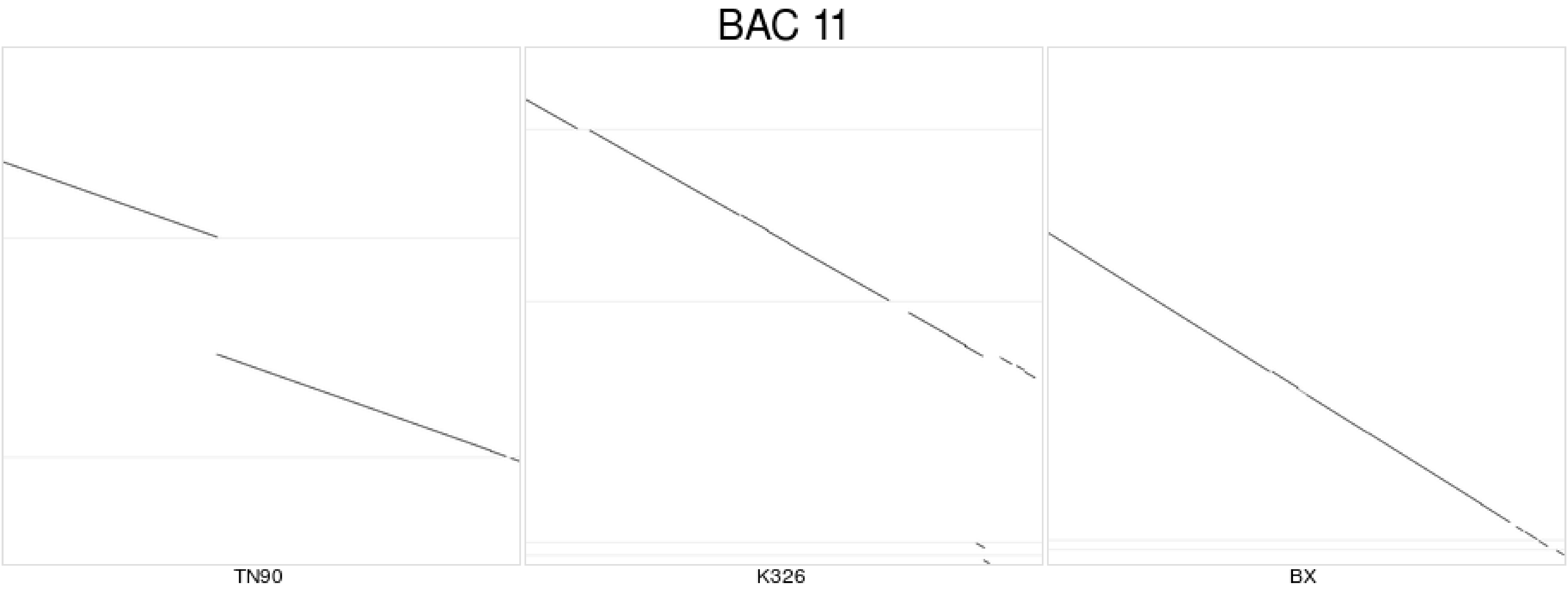
BAC 11
TN90
K326
BX

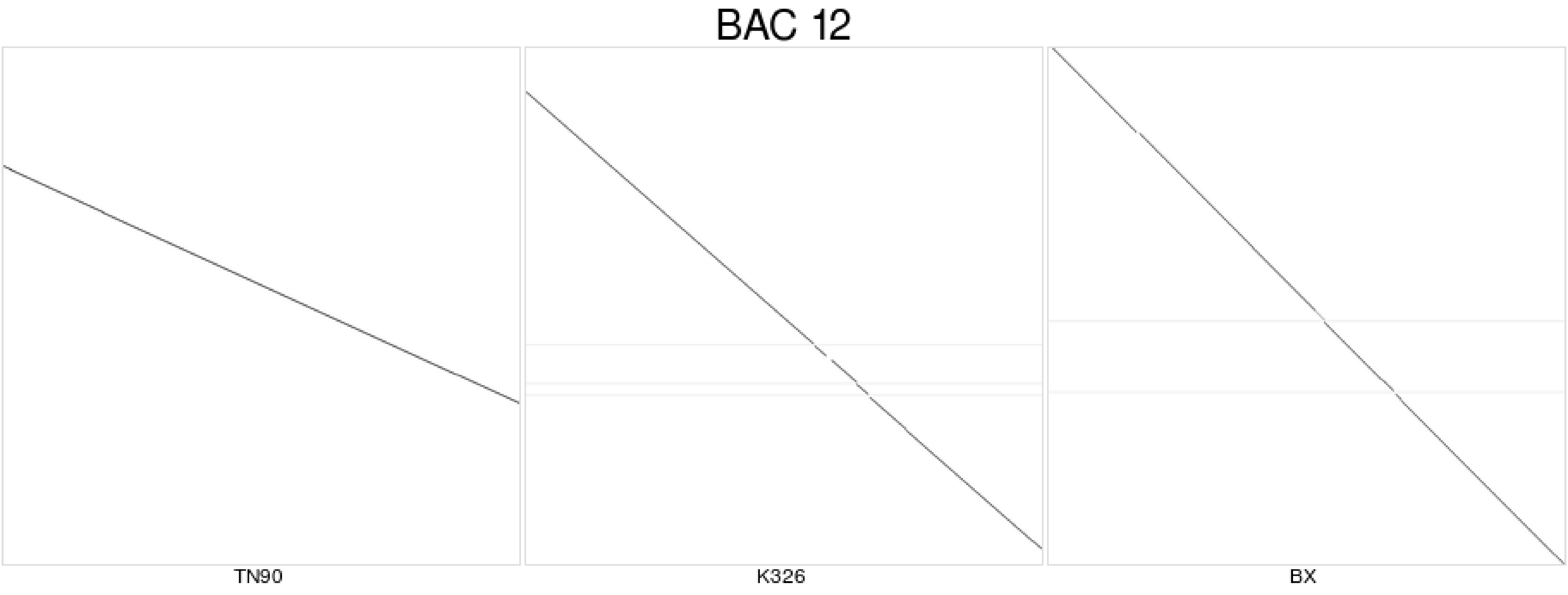

BAC 12
TN90
K326
BX

# BAC 13

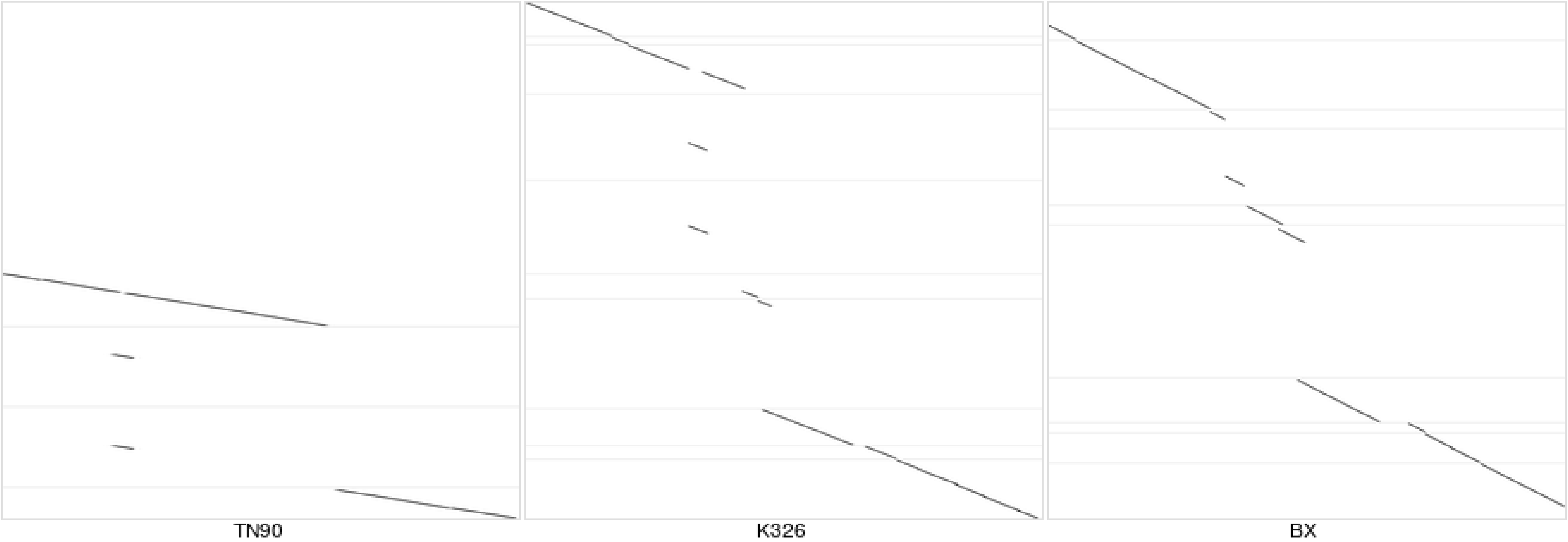

# BAC 14

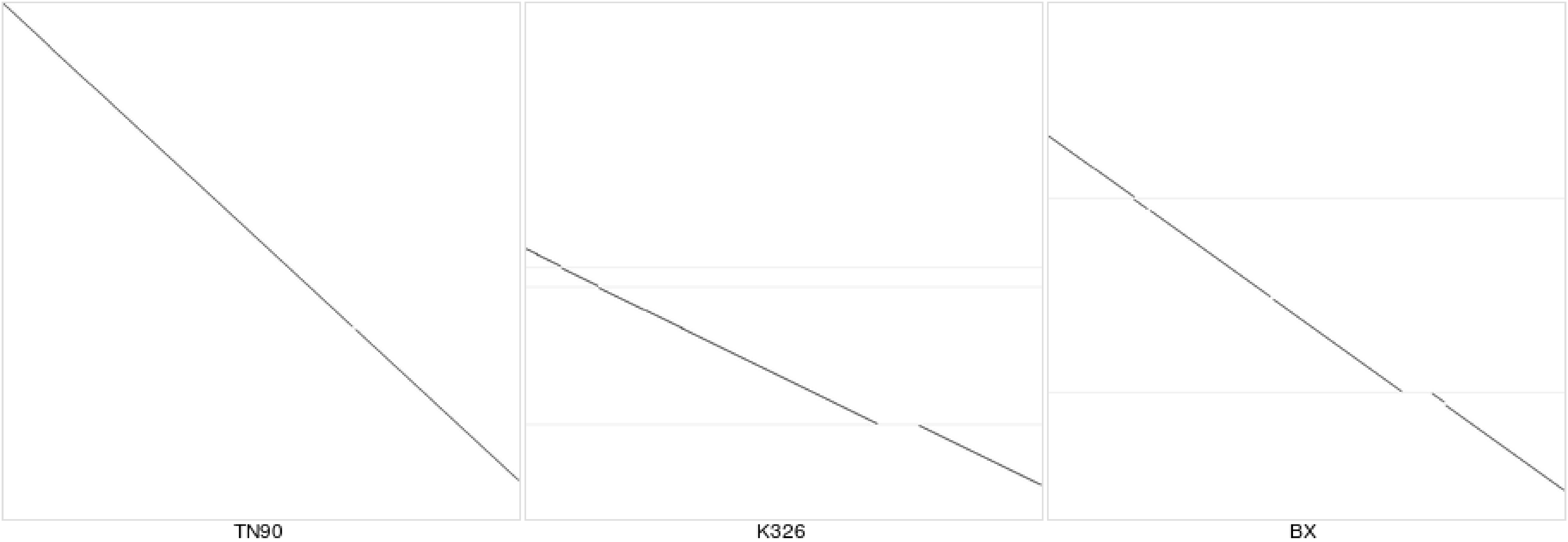

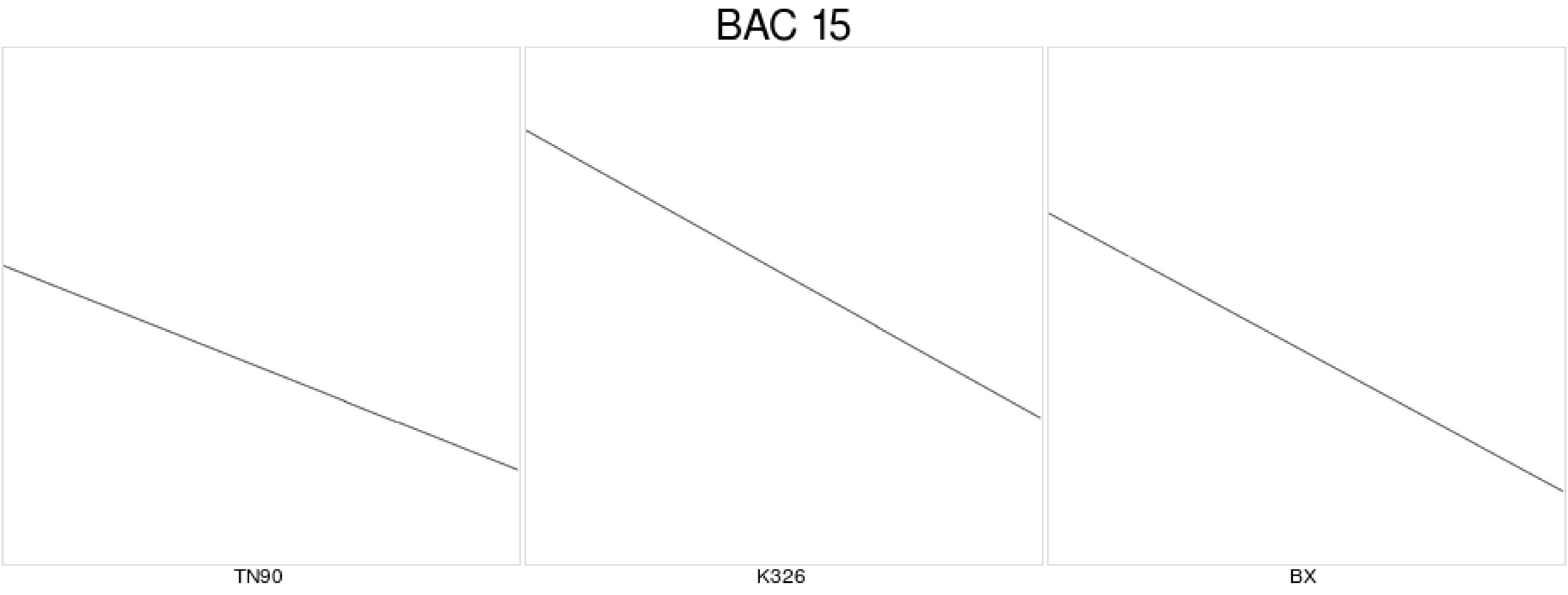
BAC 15
TN90
K326
BX

BAC 16

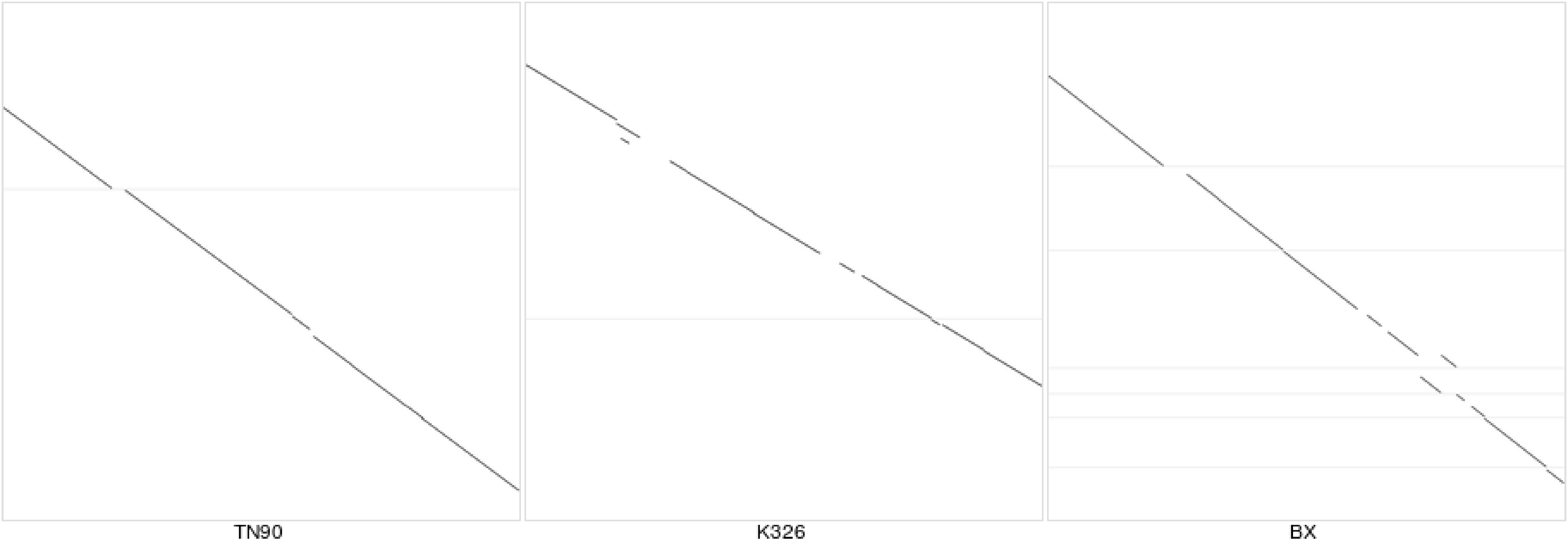

# BAC 17

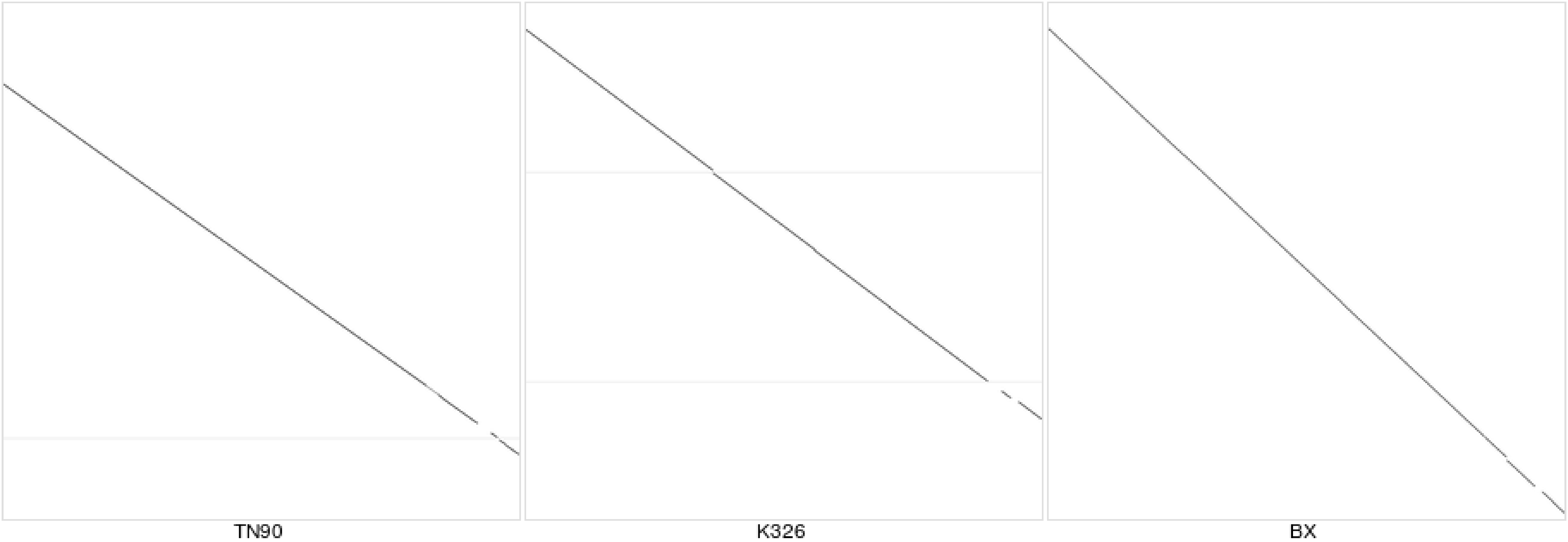

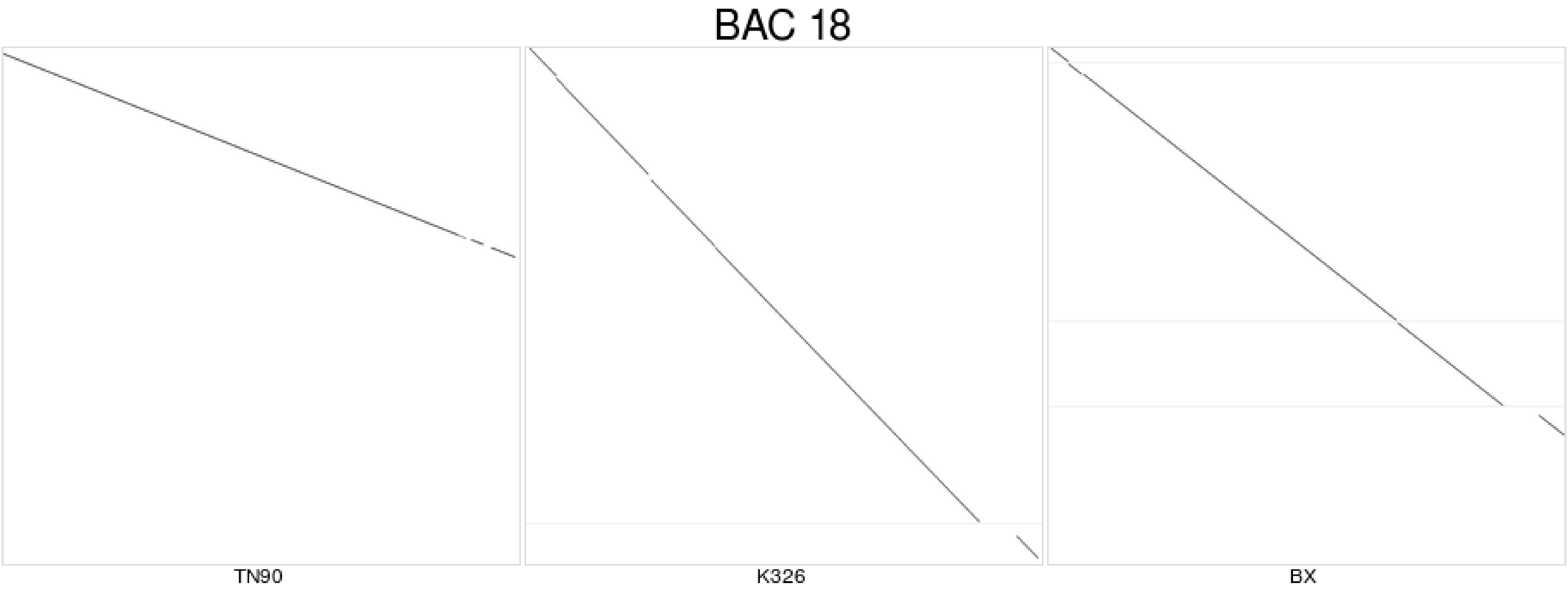
BAC 18
TN90
K326
BX

BAC 19

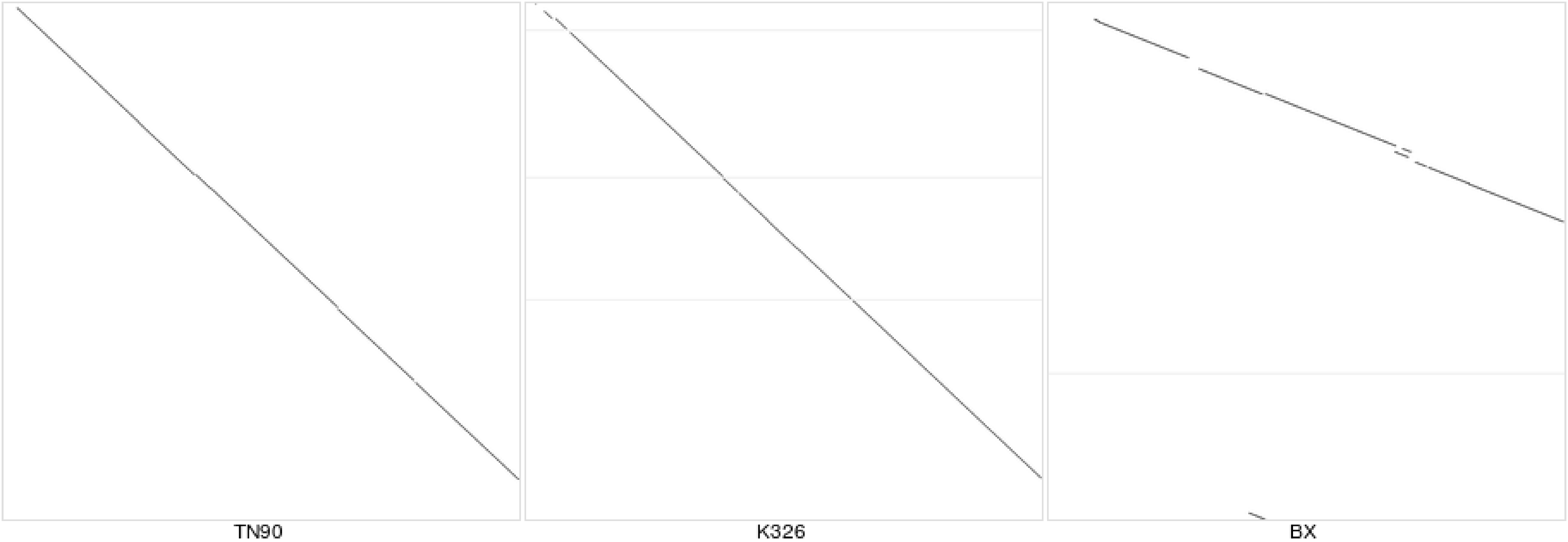

# BAC 20

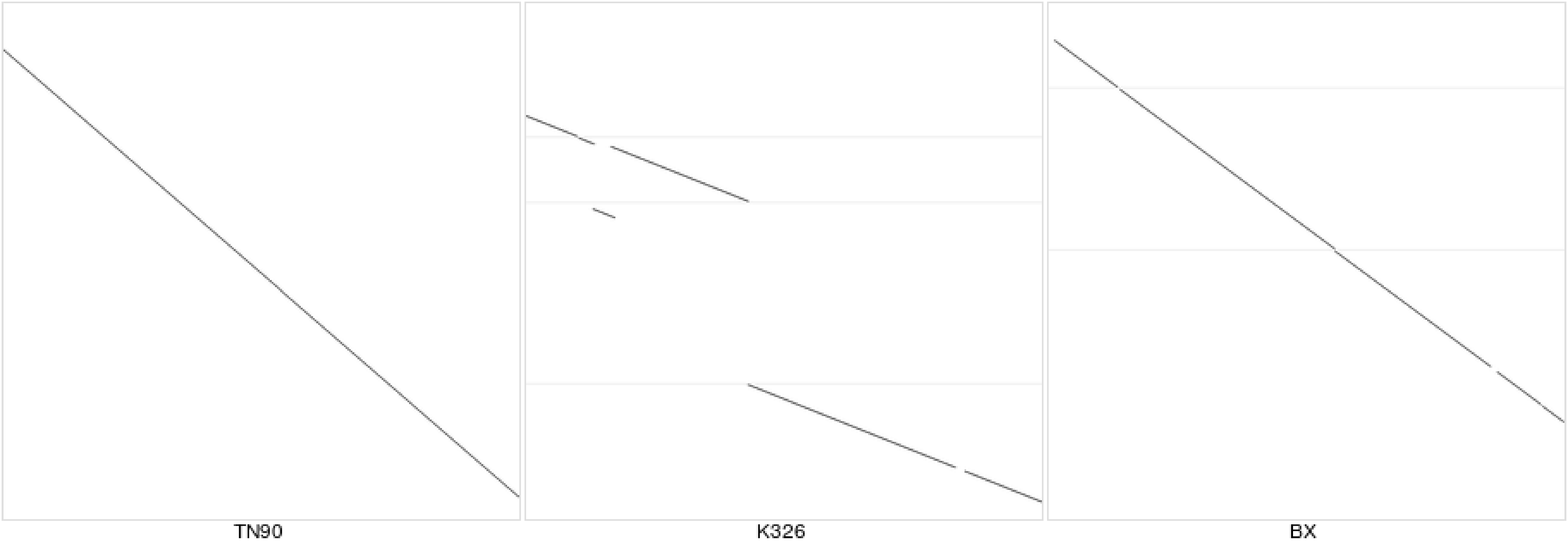

Supplement: Supplementary Data 1 — Alignment of assembly scaffolds to Nicotiana tabacum Hicks Broadleaf BAC sequences. [file ncomms4833-s2.pdf]
