# Supplementary figures and images for: The tobacco genome sequence and its comparison with those of tomato and potato
Source: Nat Commun. 2014 May 8;5:3833. doi: 10.1038/ncomms4833 (PMC4024737; doi:10.1038/ncomms4833)

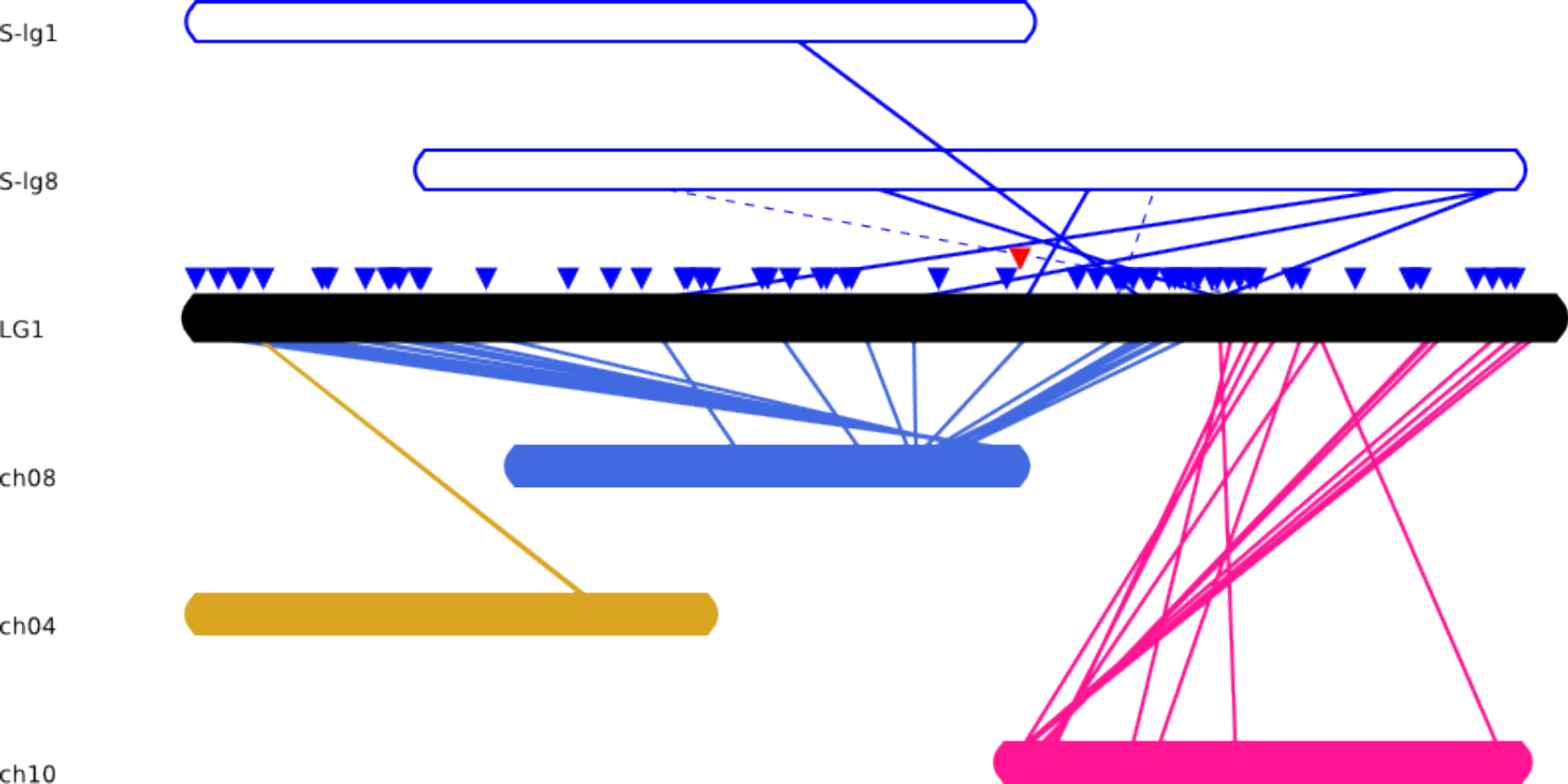

S-Ig7

T-Ig7

LG2

ch07

ch09

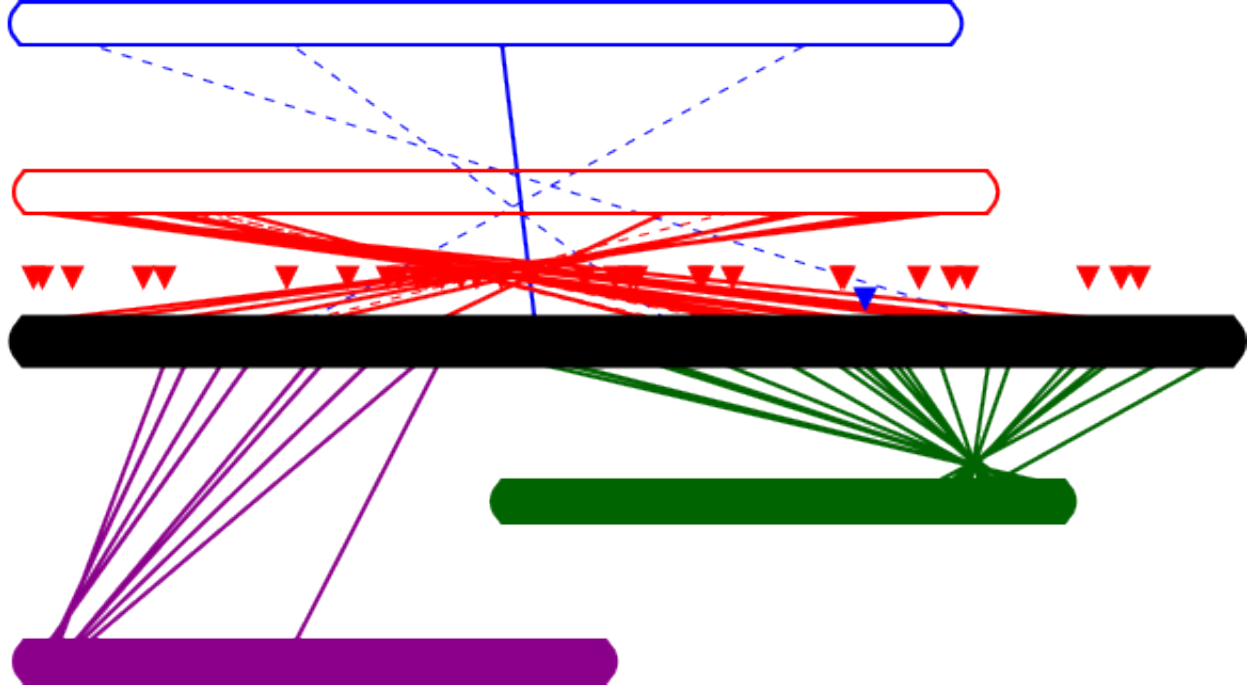

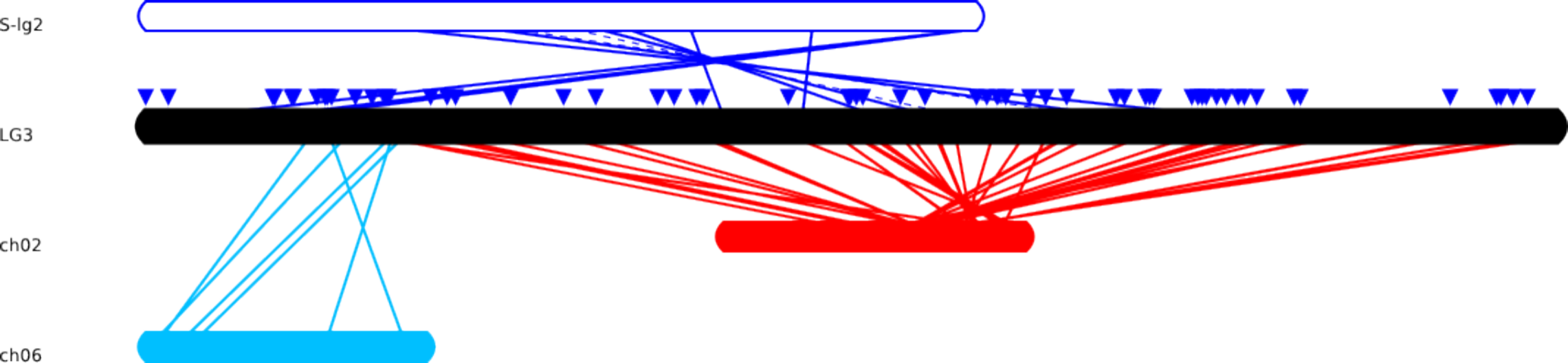

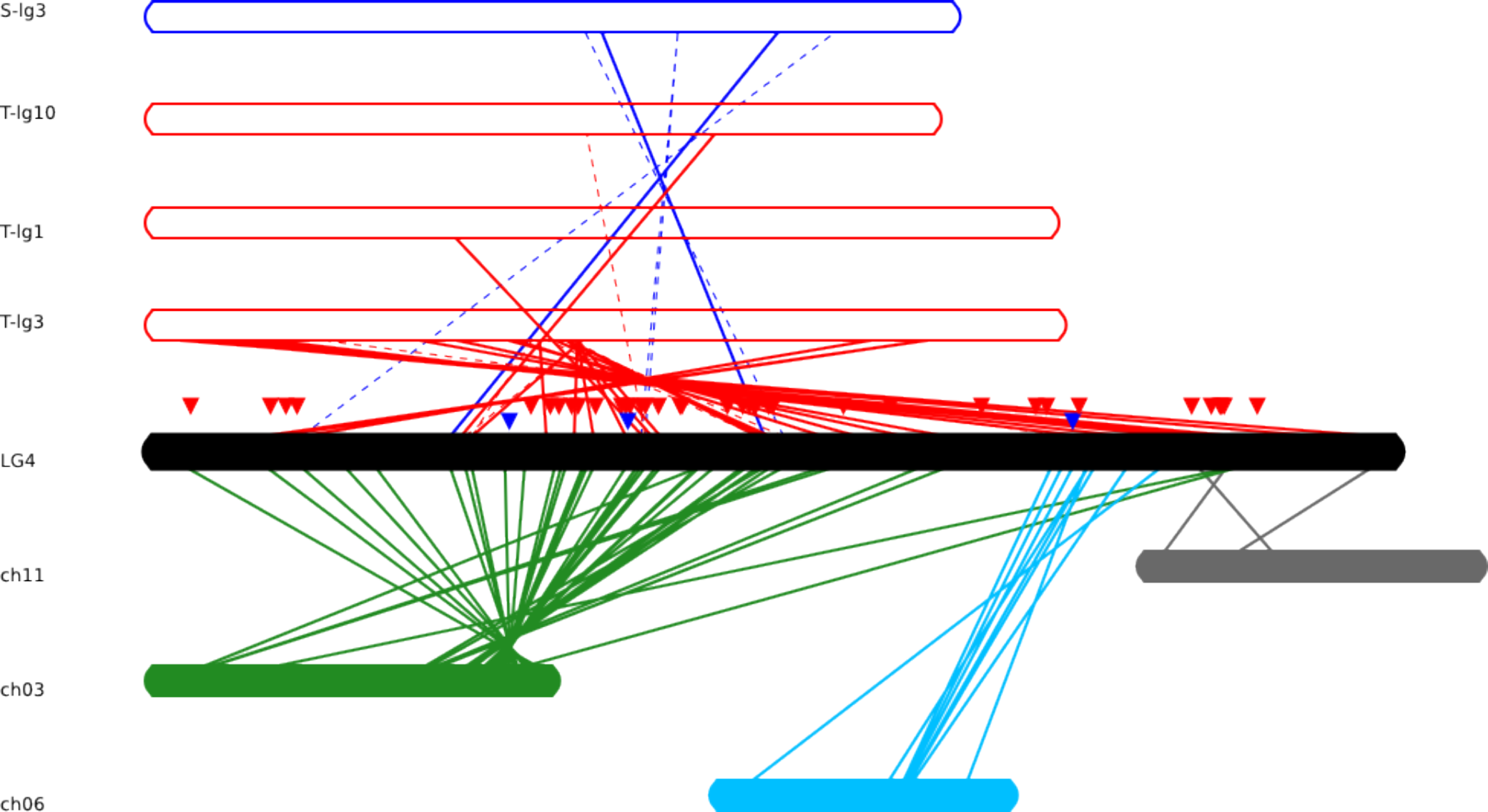

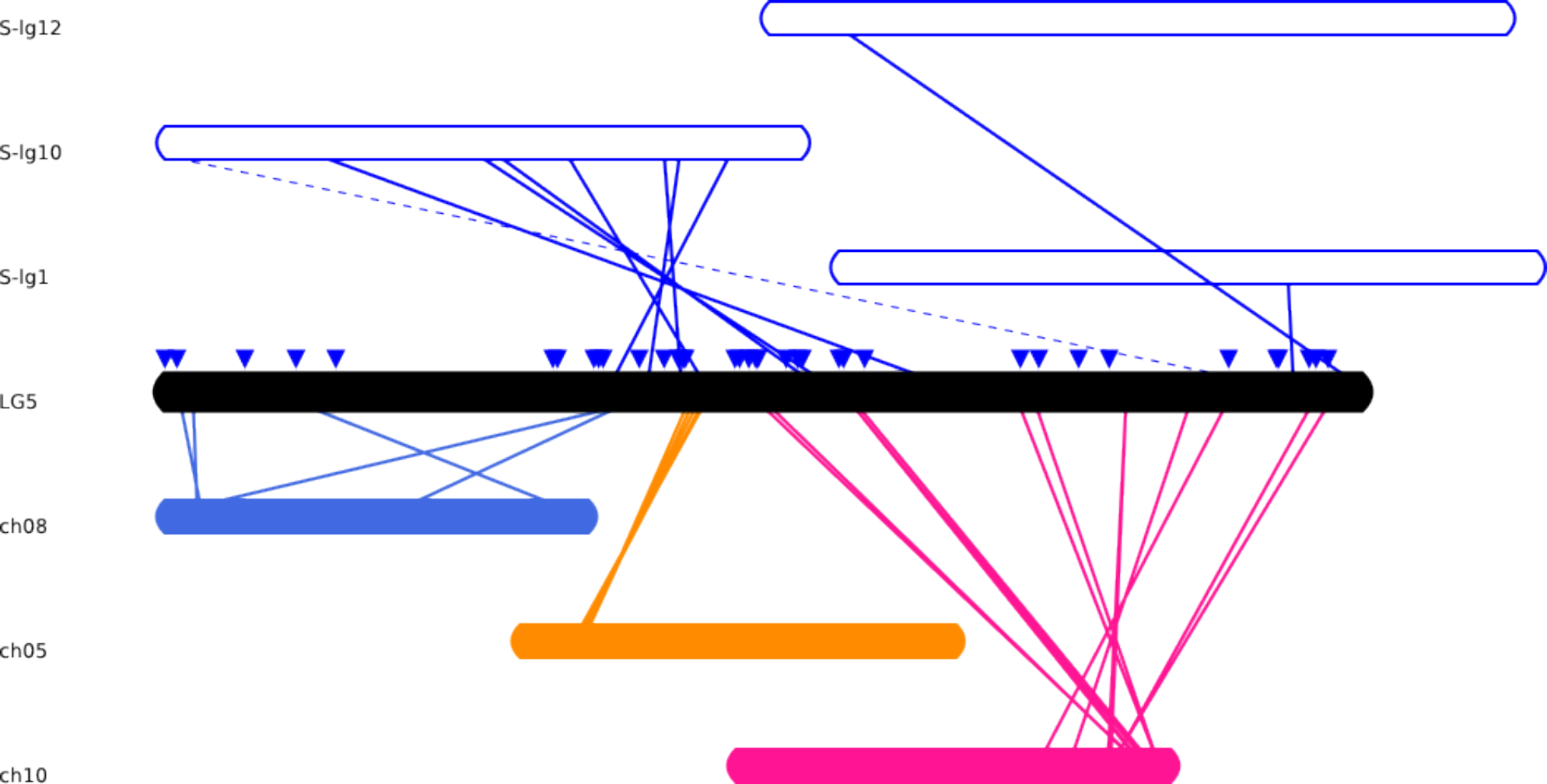

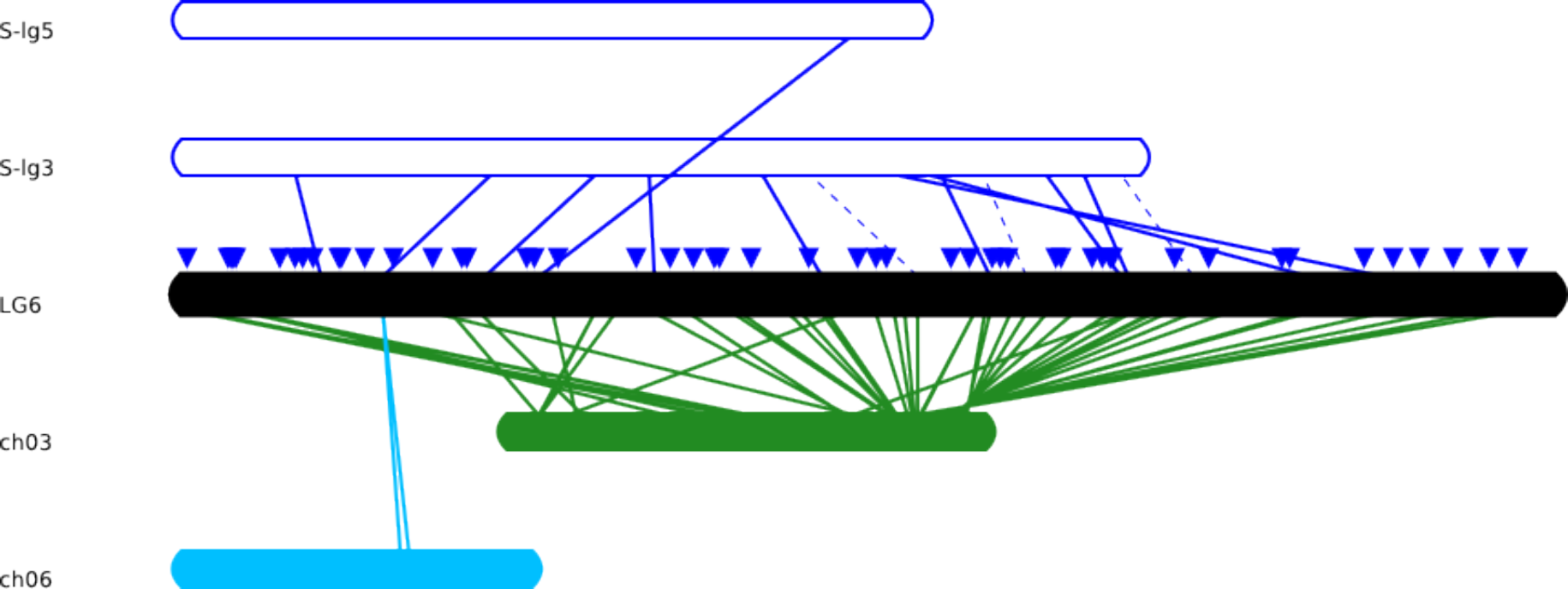

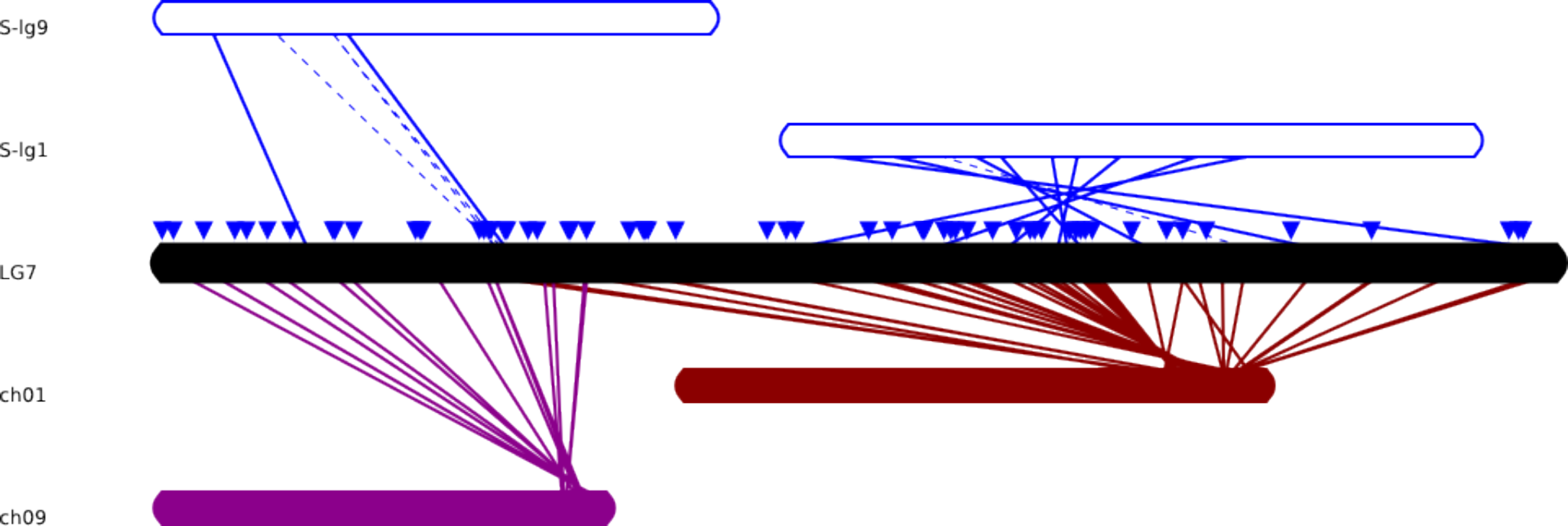

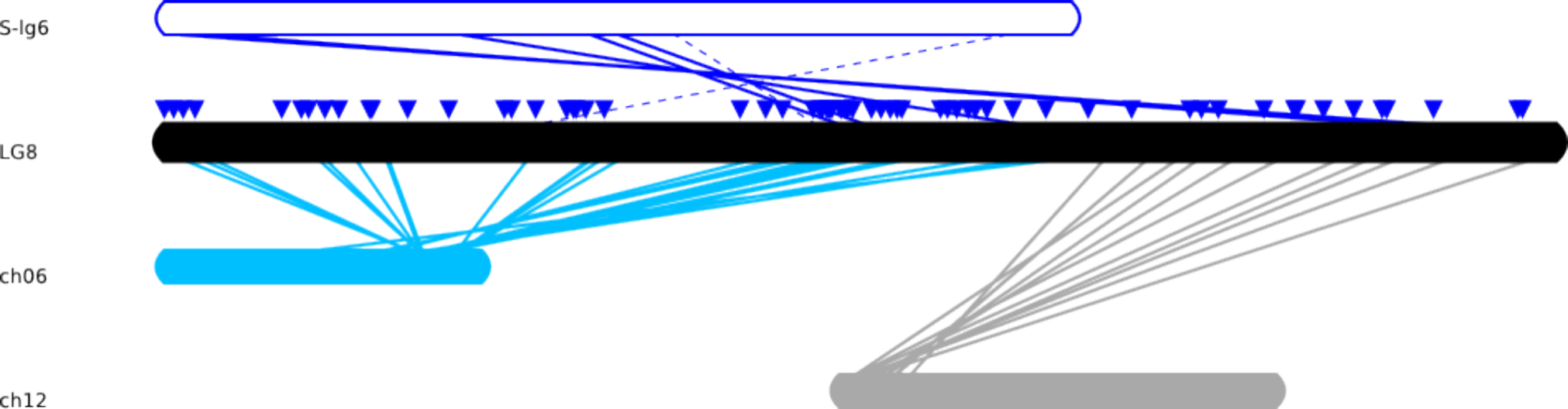

S-Ig11

T-Ig11

LG9

ch11

ch01

ch07

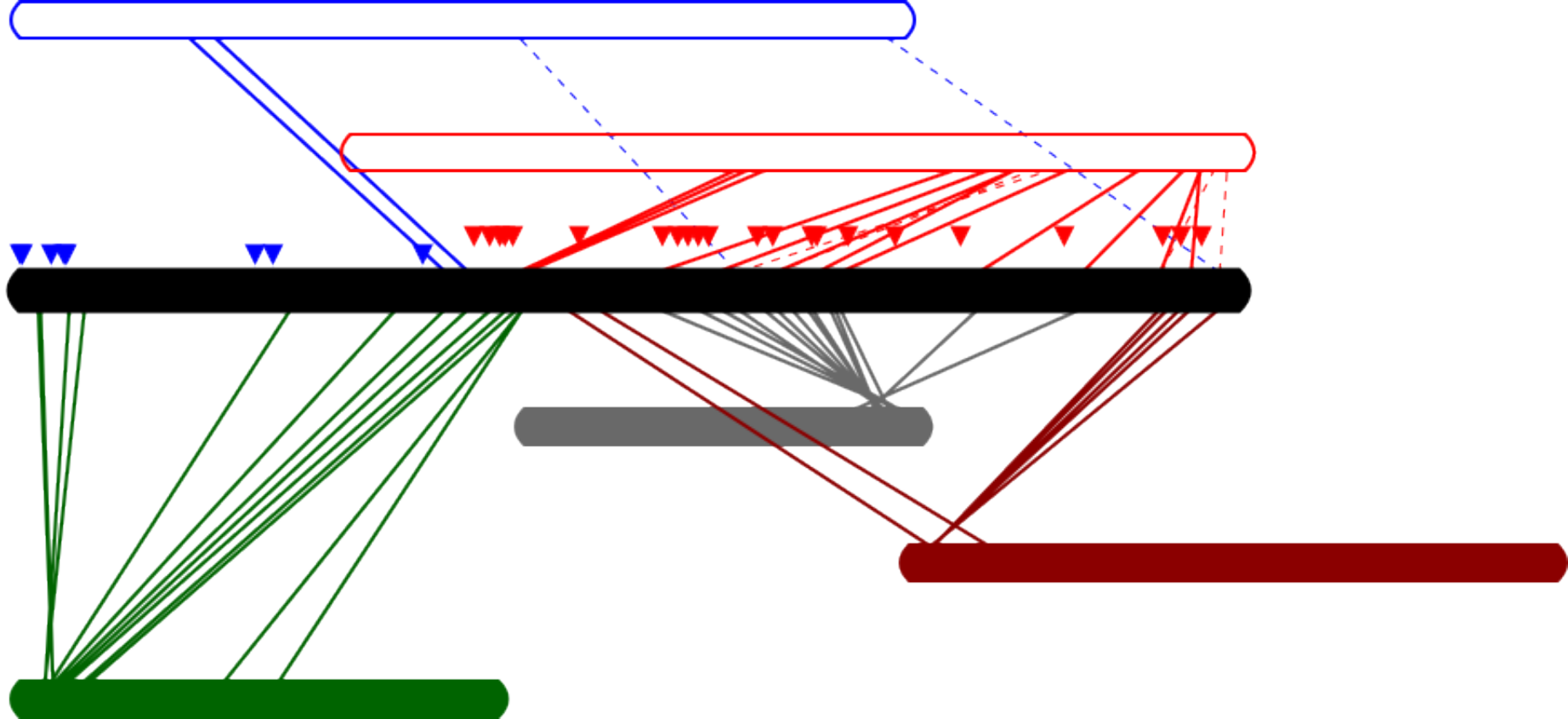

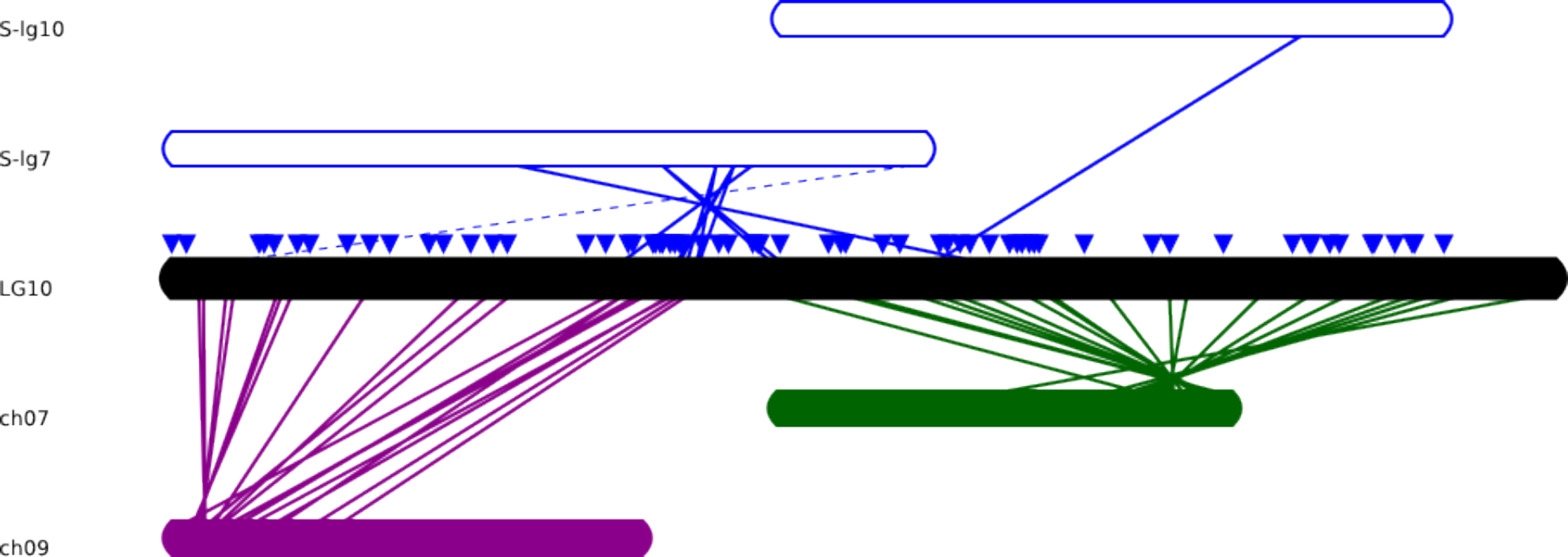

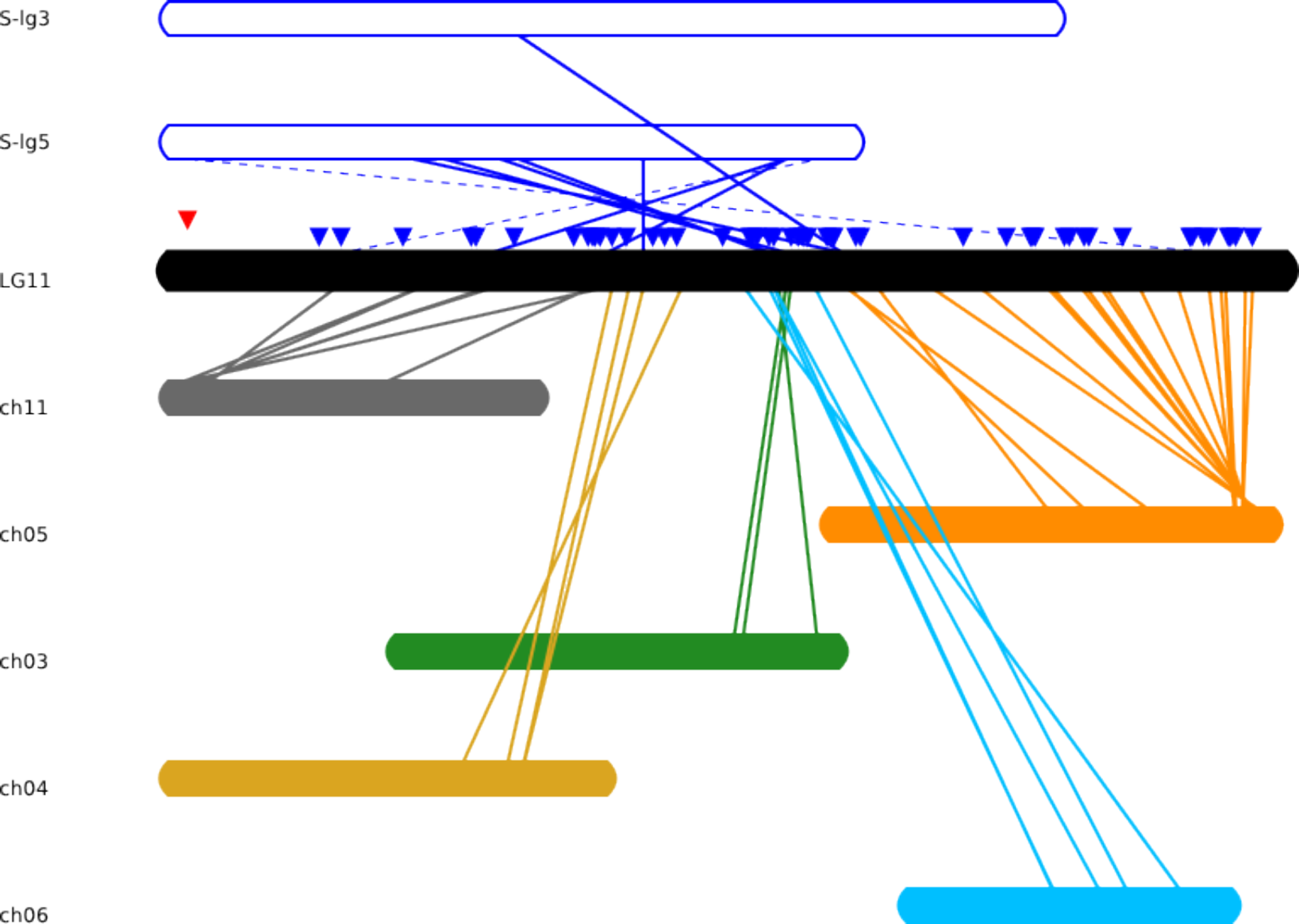

S-Ig4

T-Ig8

T-Ig4

LG12

ch11

ch02

ch04

ch10

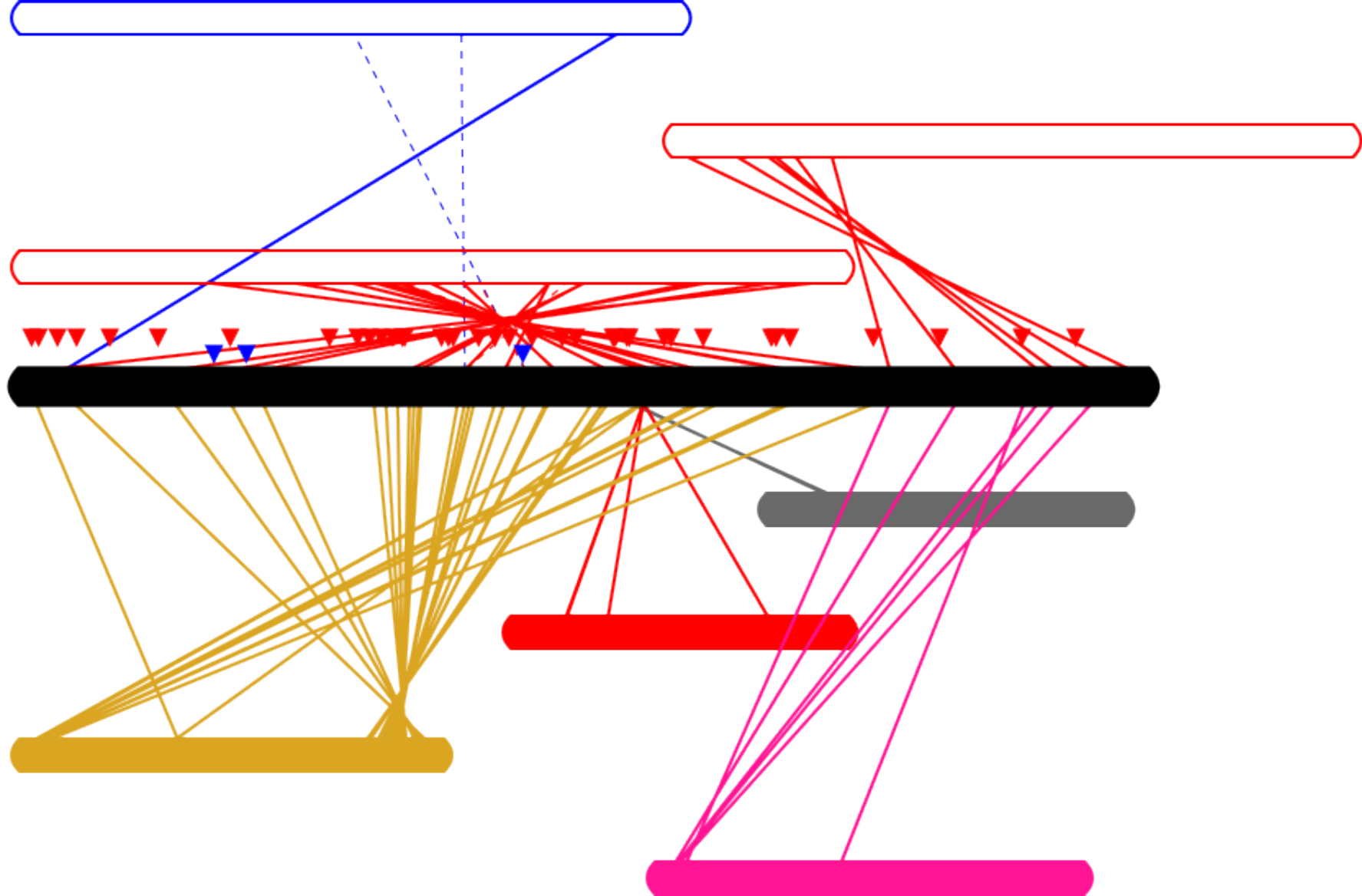

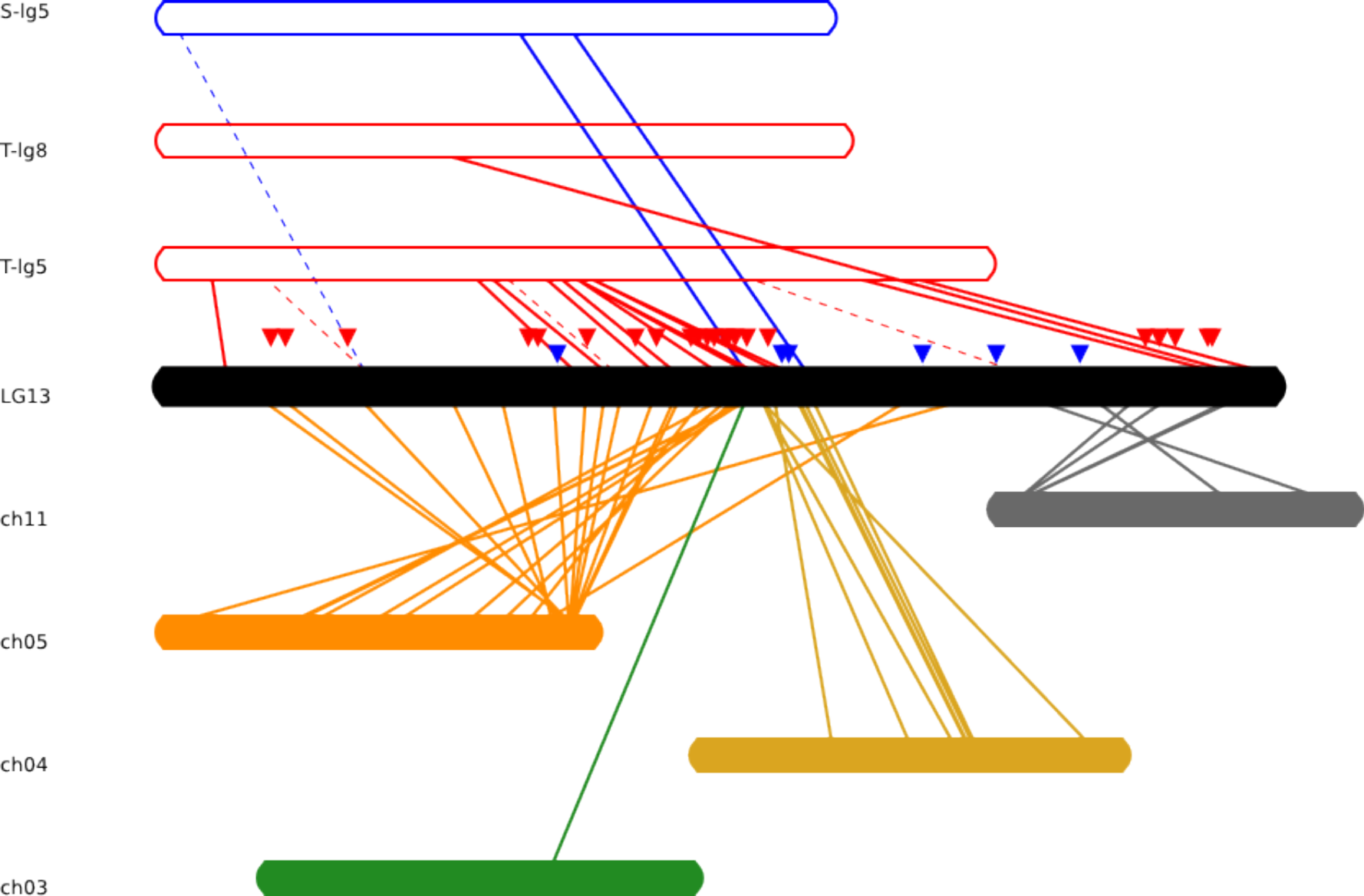

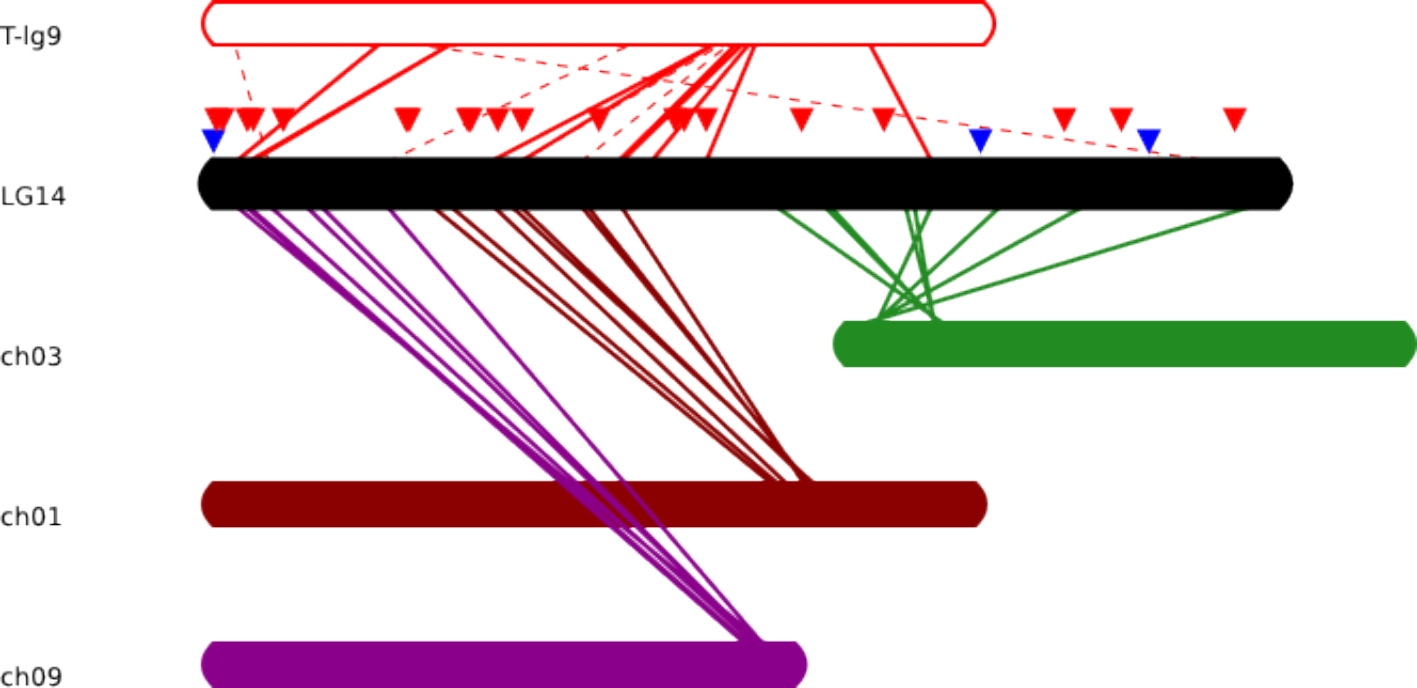

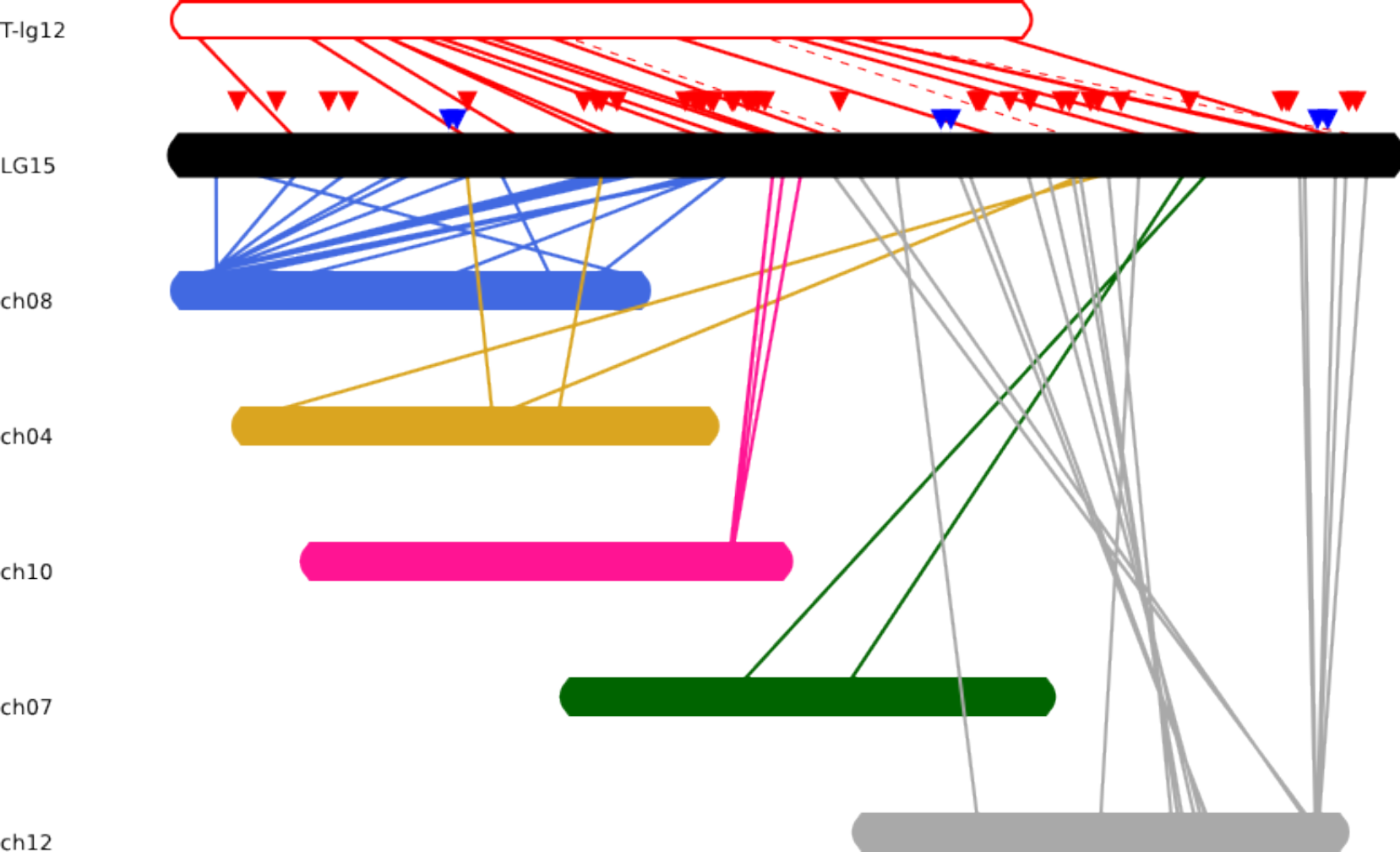

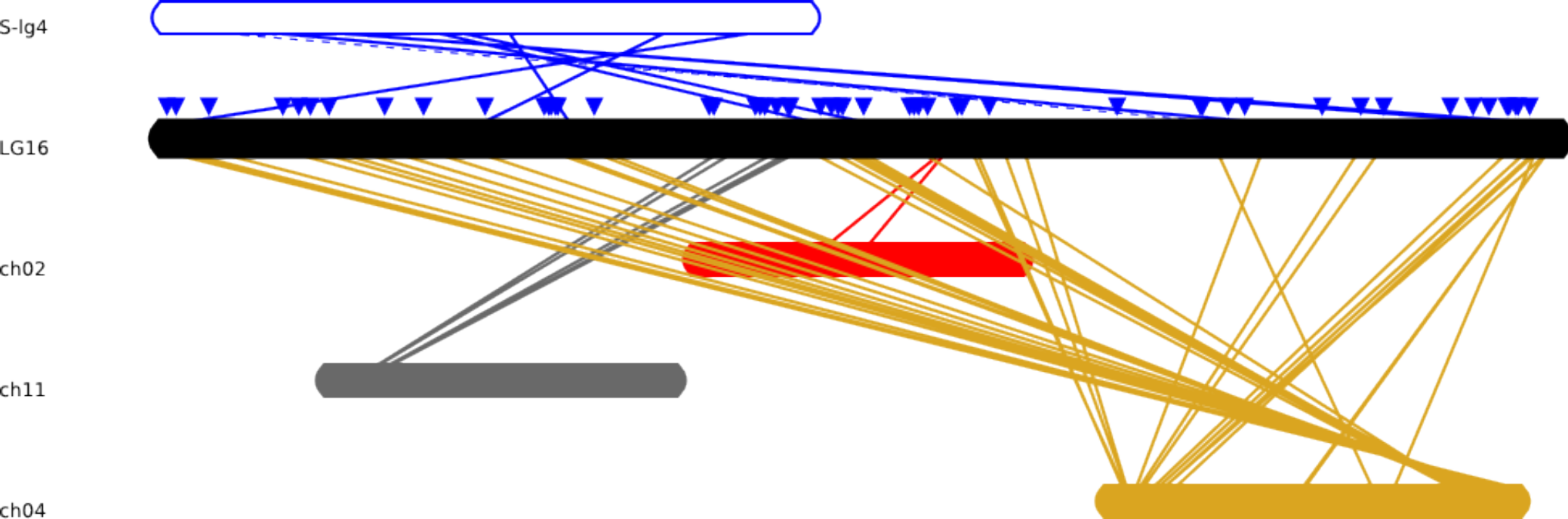

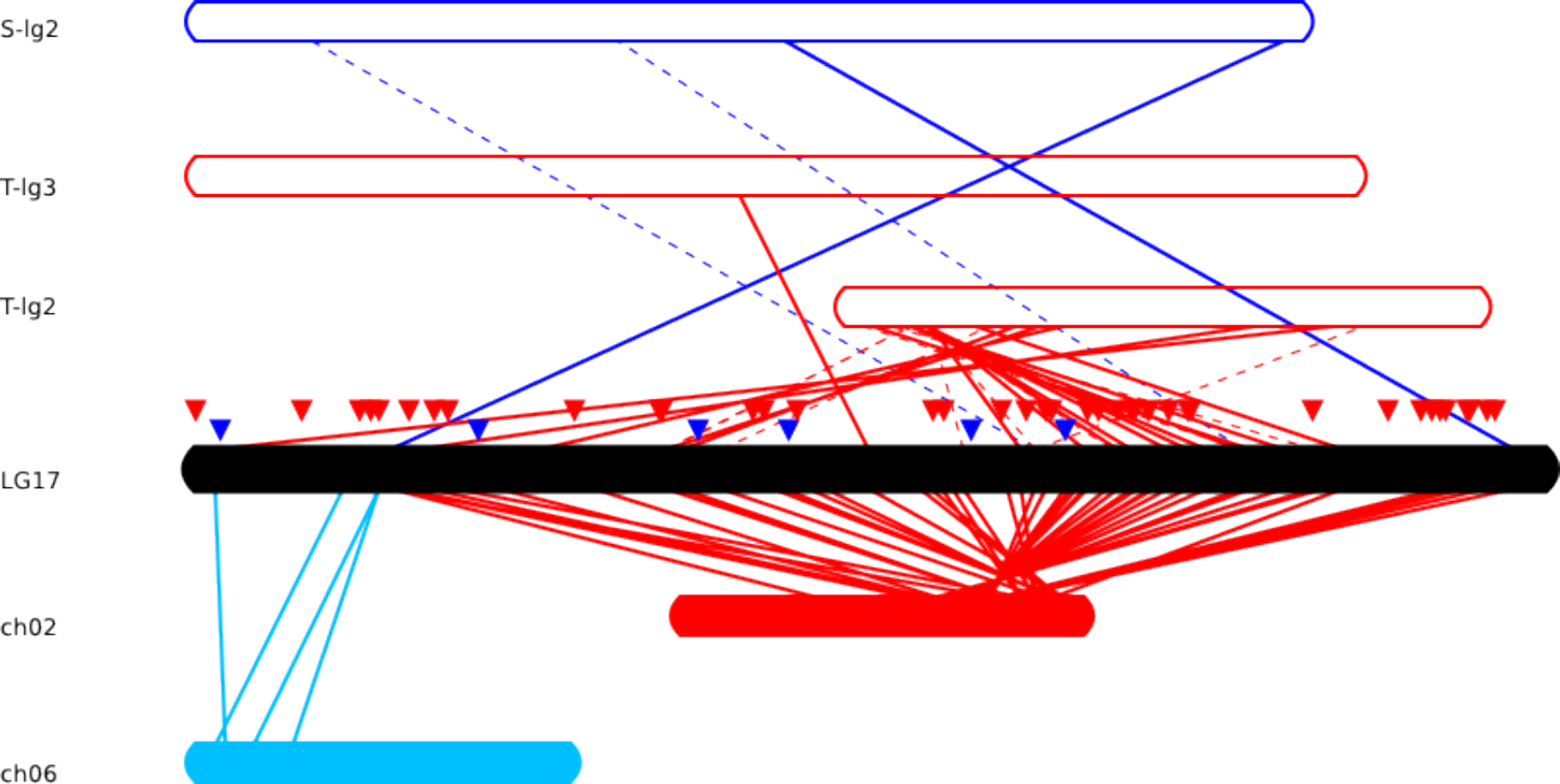

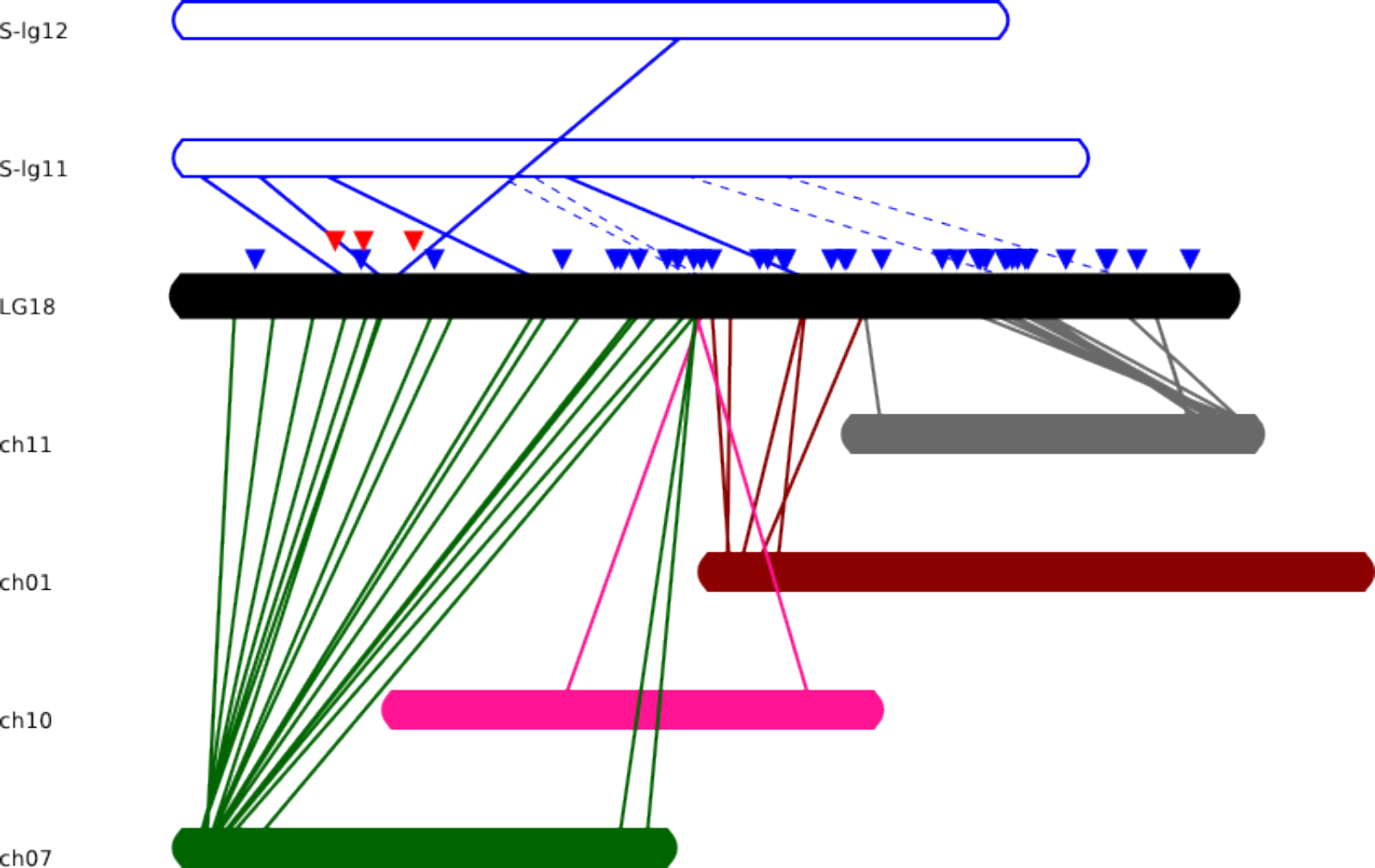

S-Ig1

T-Ig1

LG19

ch01

ch12

ch09

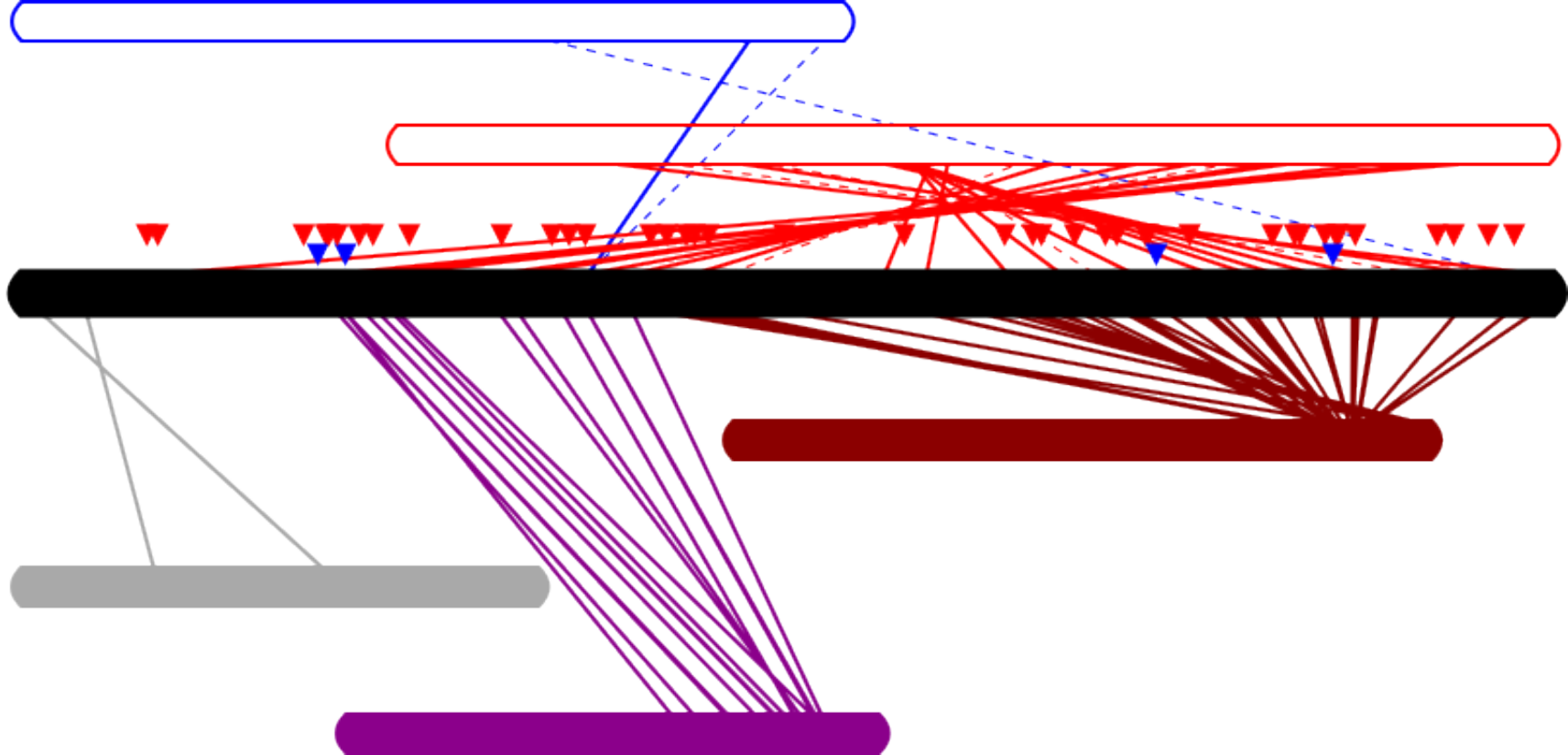

S-Ig12

LG20

ch05

ch12

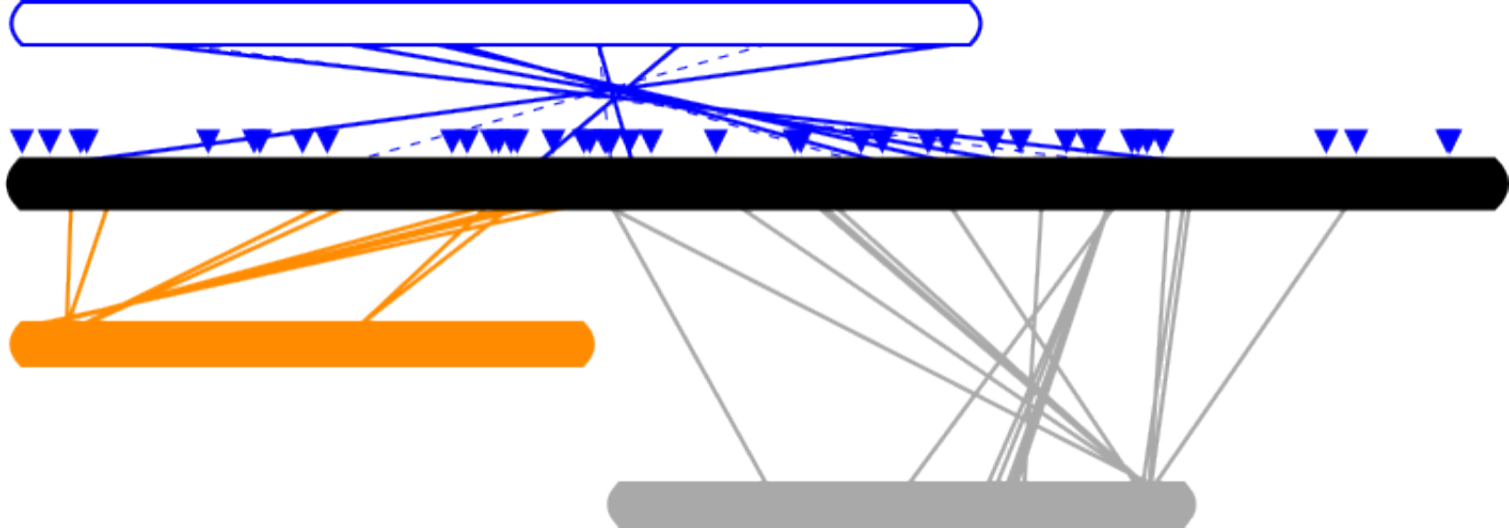

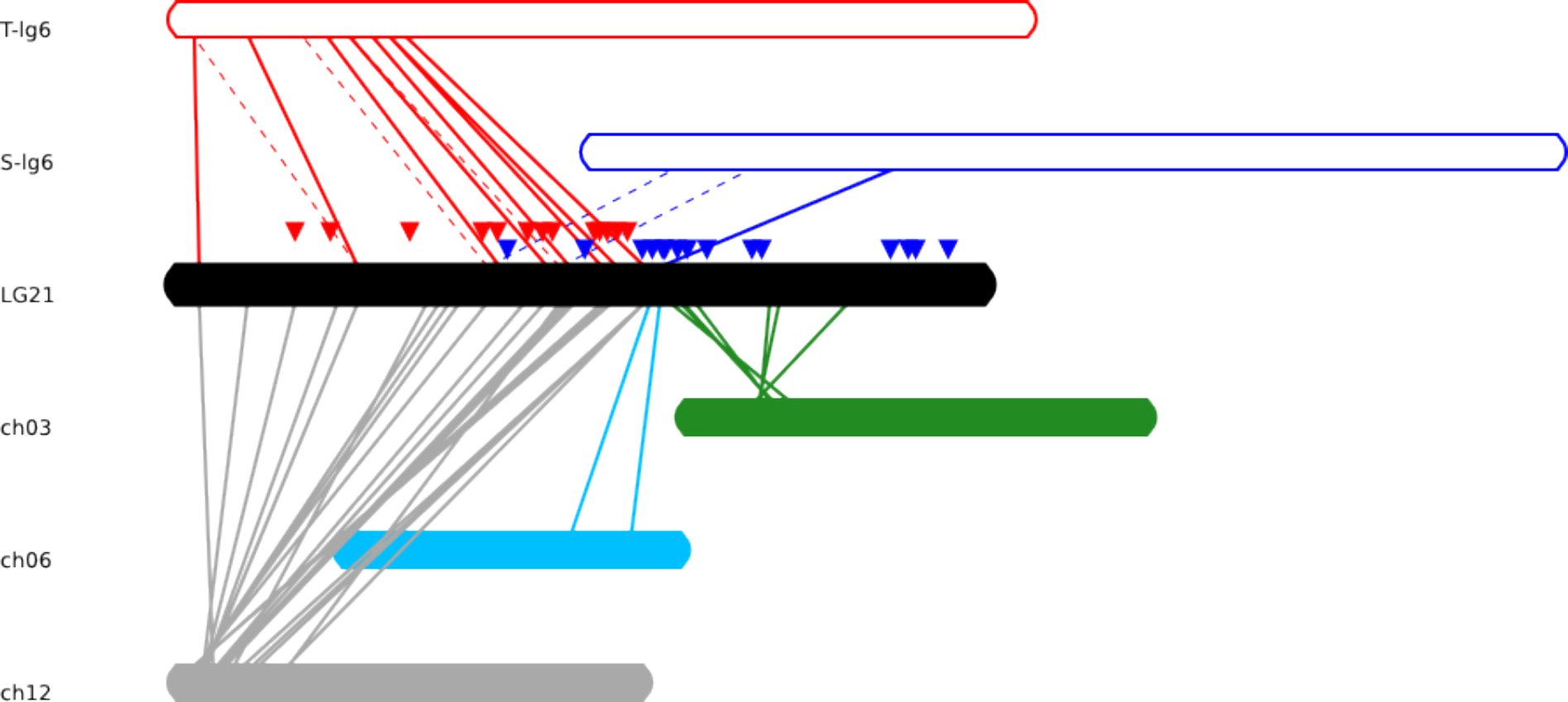

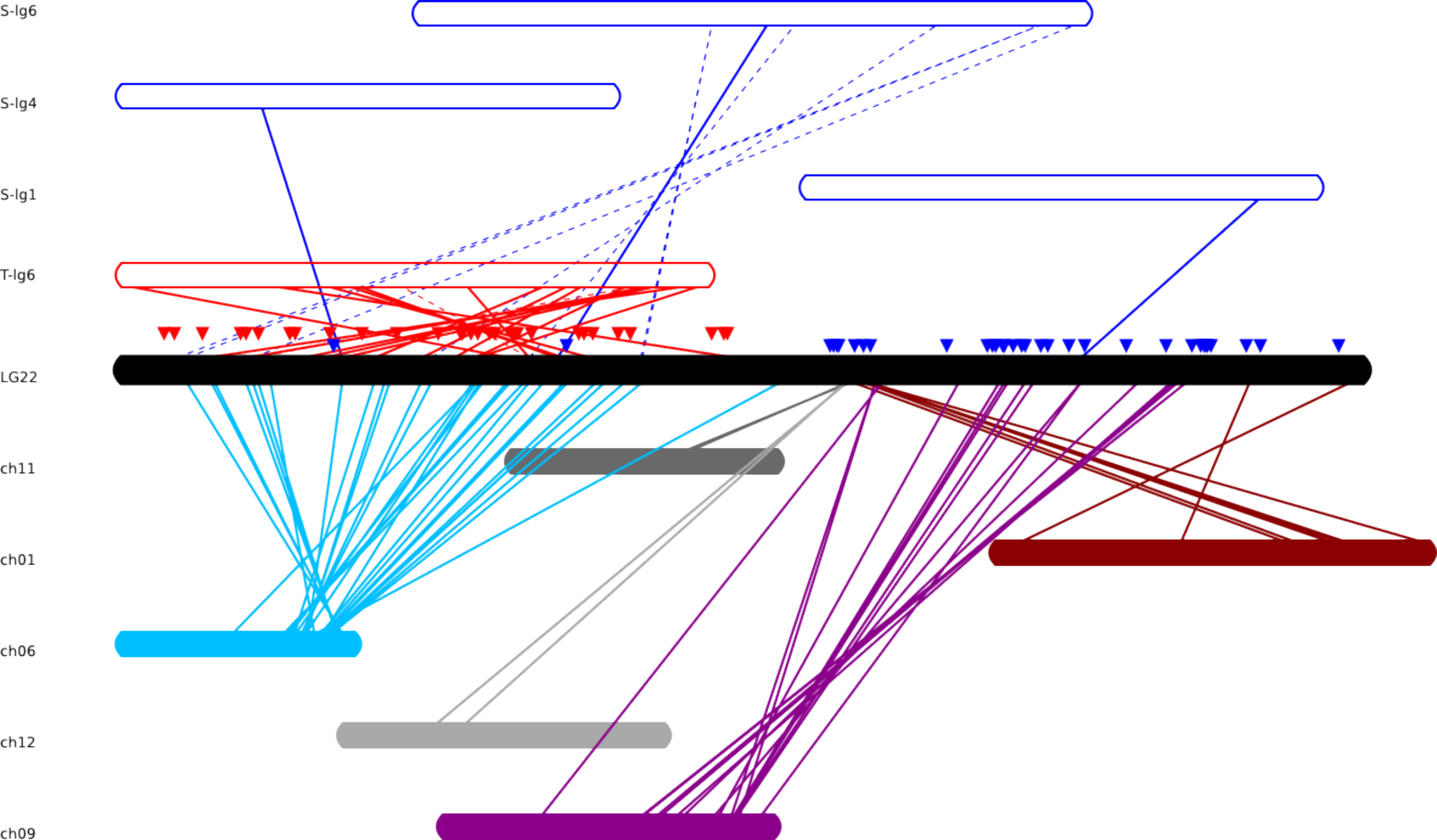

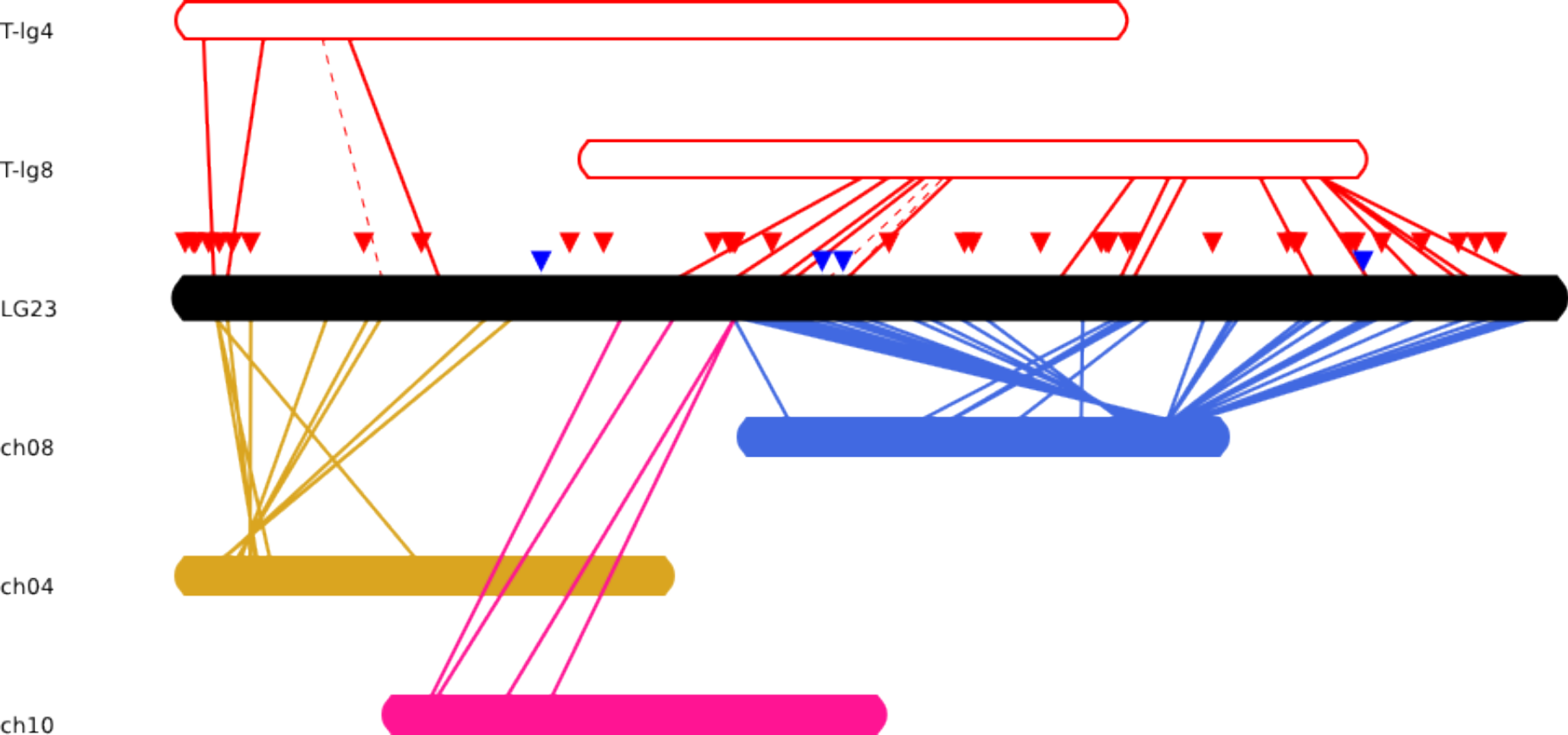

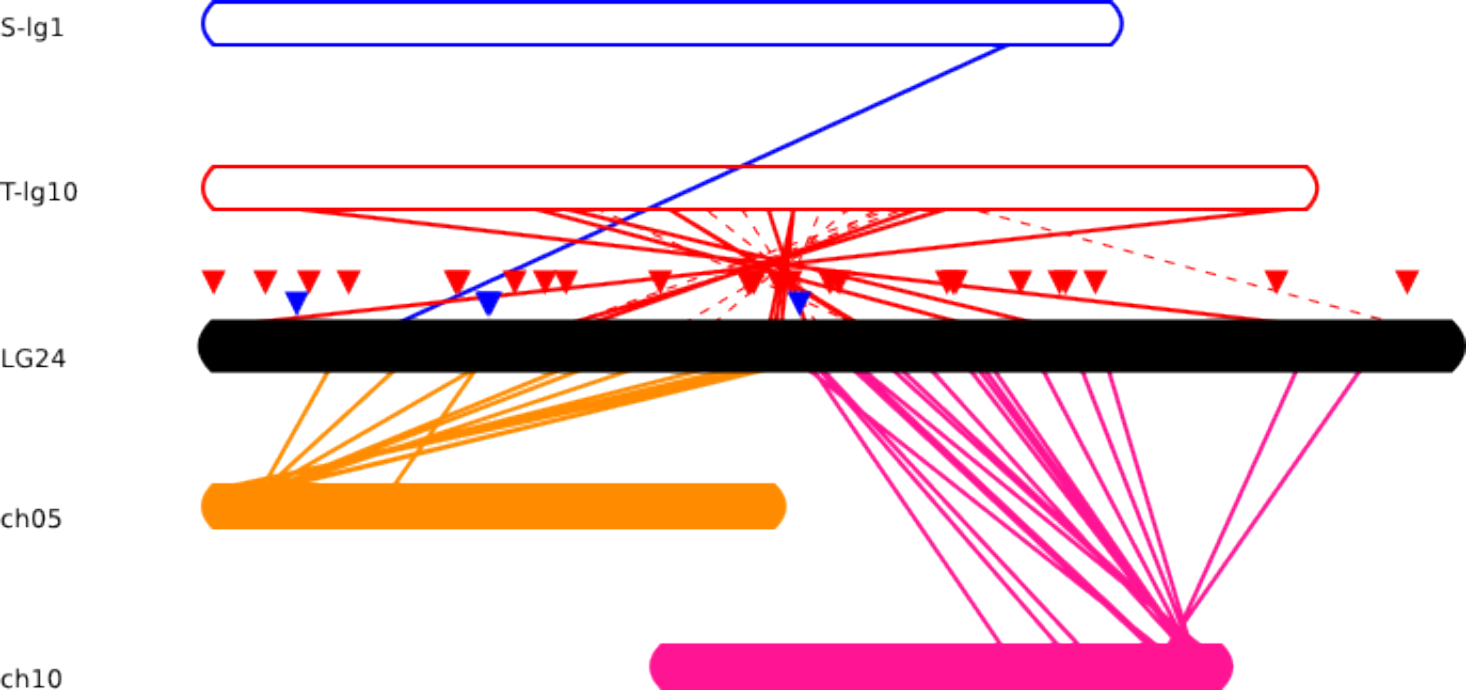

Supplement: Supplementary Data 2 — Synteny of the 24 Nicotiana tabacum linkage groups with the 12 tomato chromosomes, based on tomato protein mapping. [file ncomms4833-s3.pdf]

S-Ig1

S-Ig8

LG1

ch08

ch04

ch10

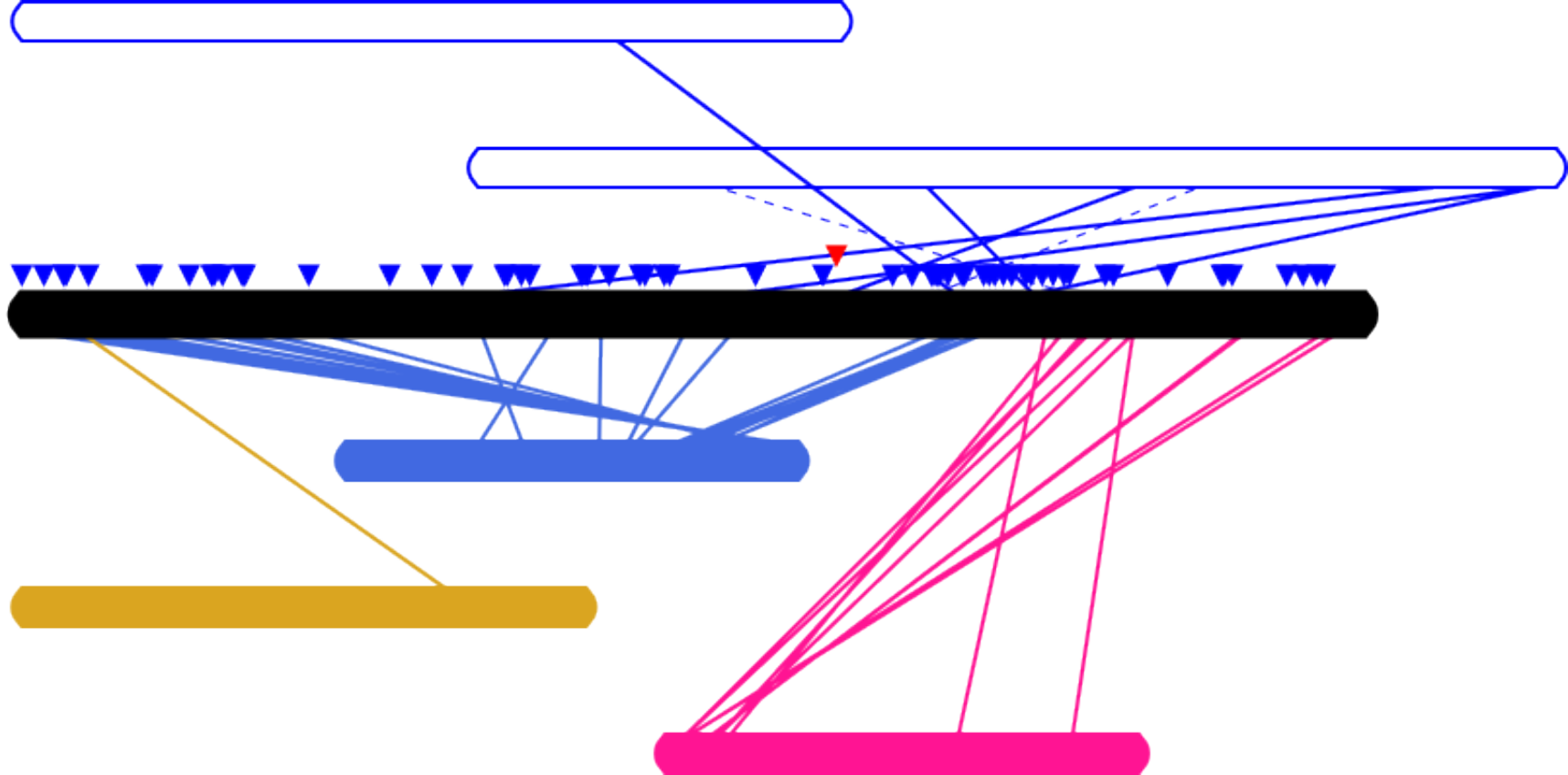

S-Ig7

T-Ig7

LG2

ch07

ch09

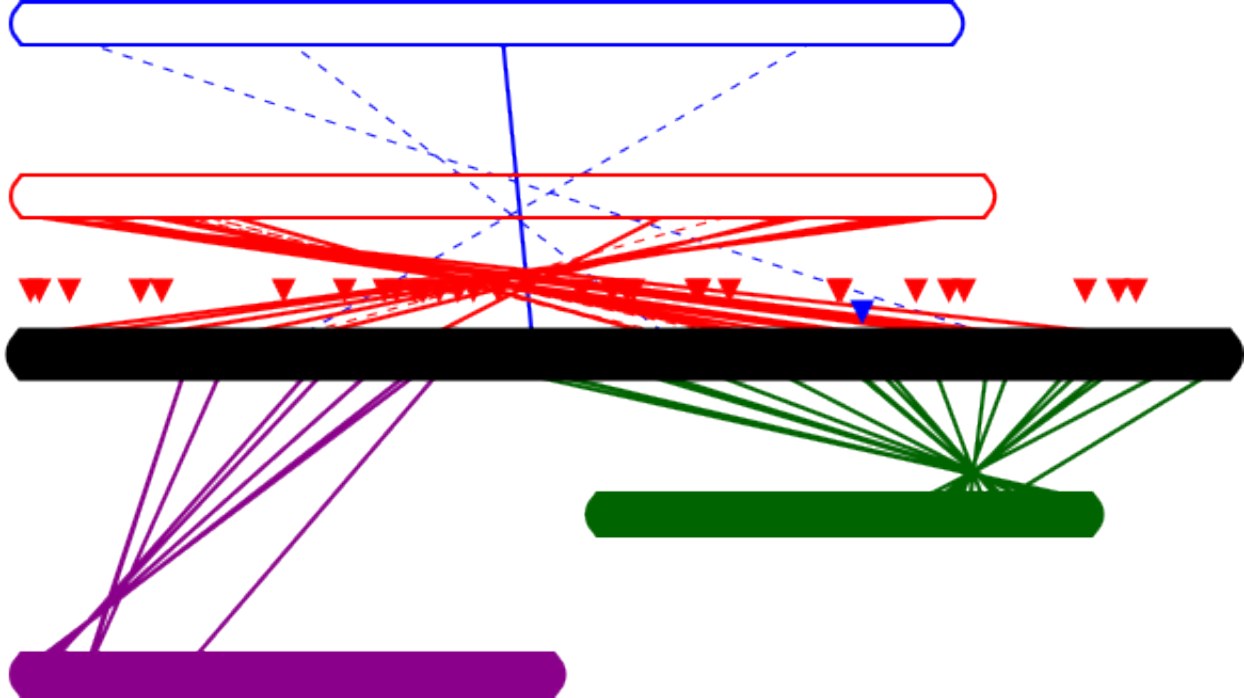

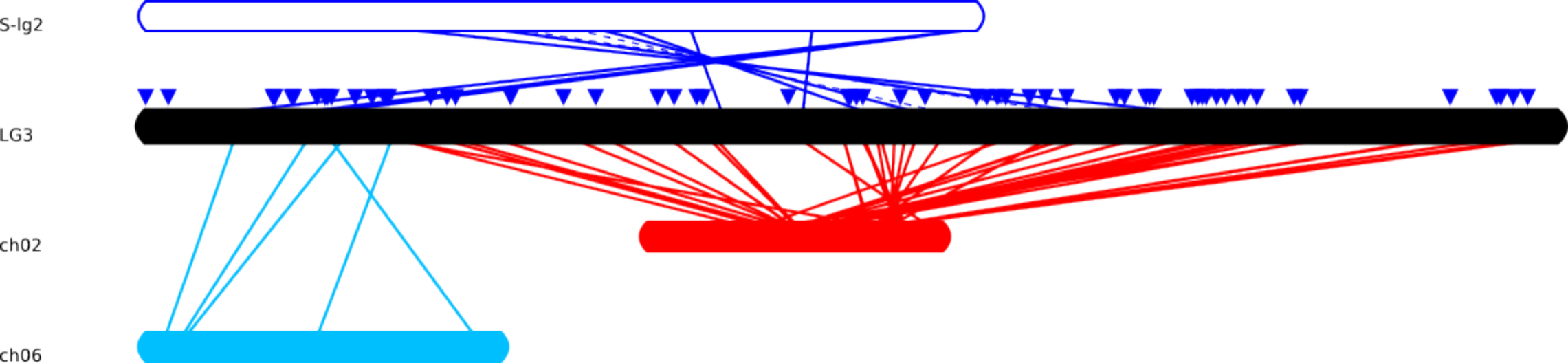

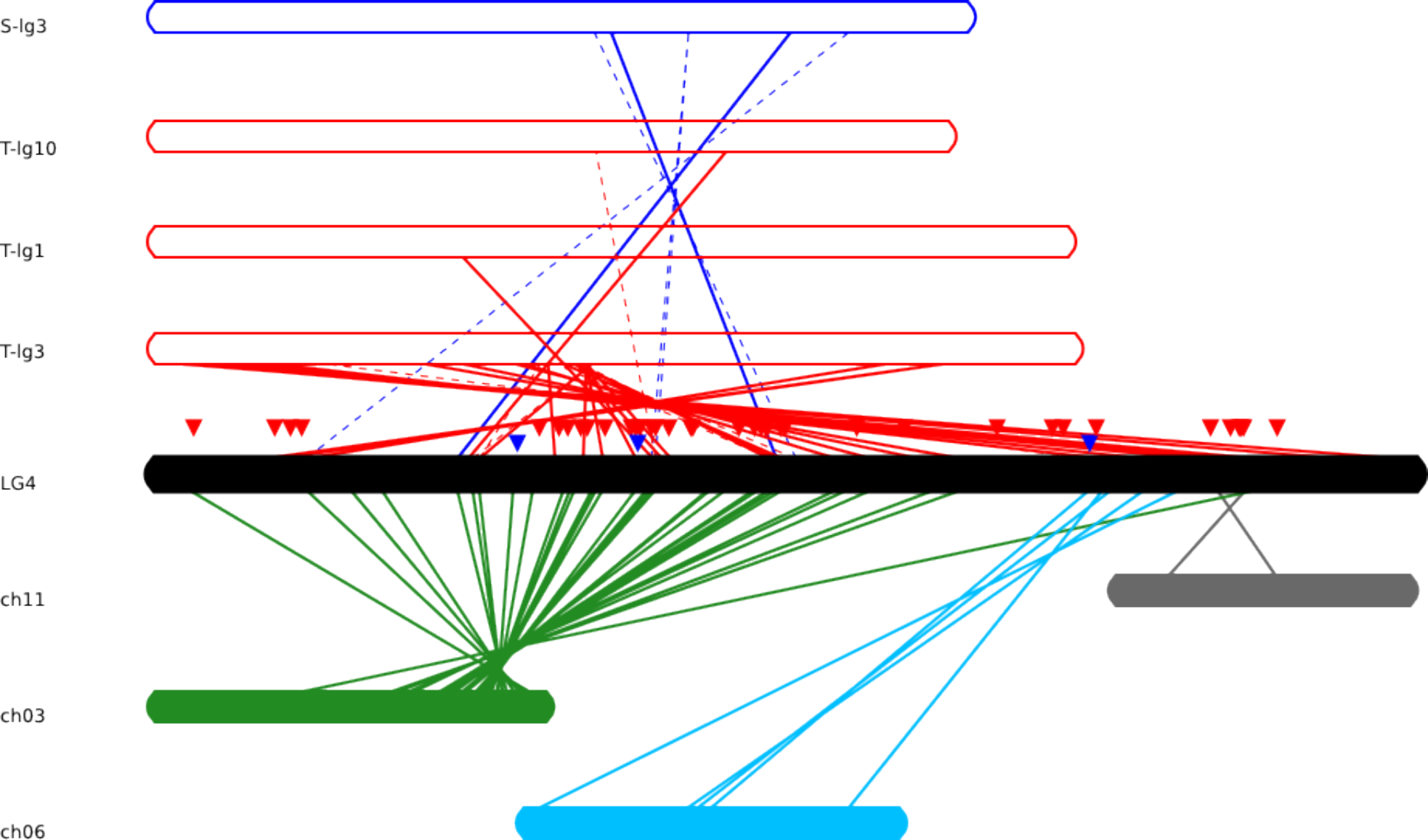

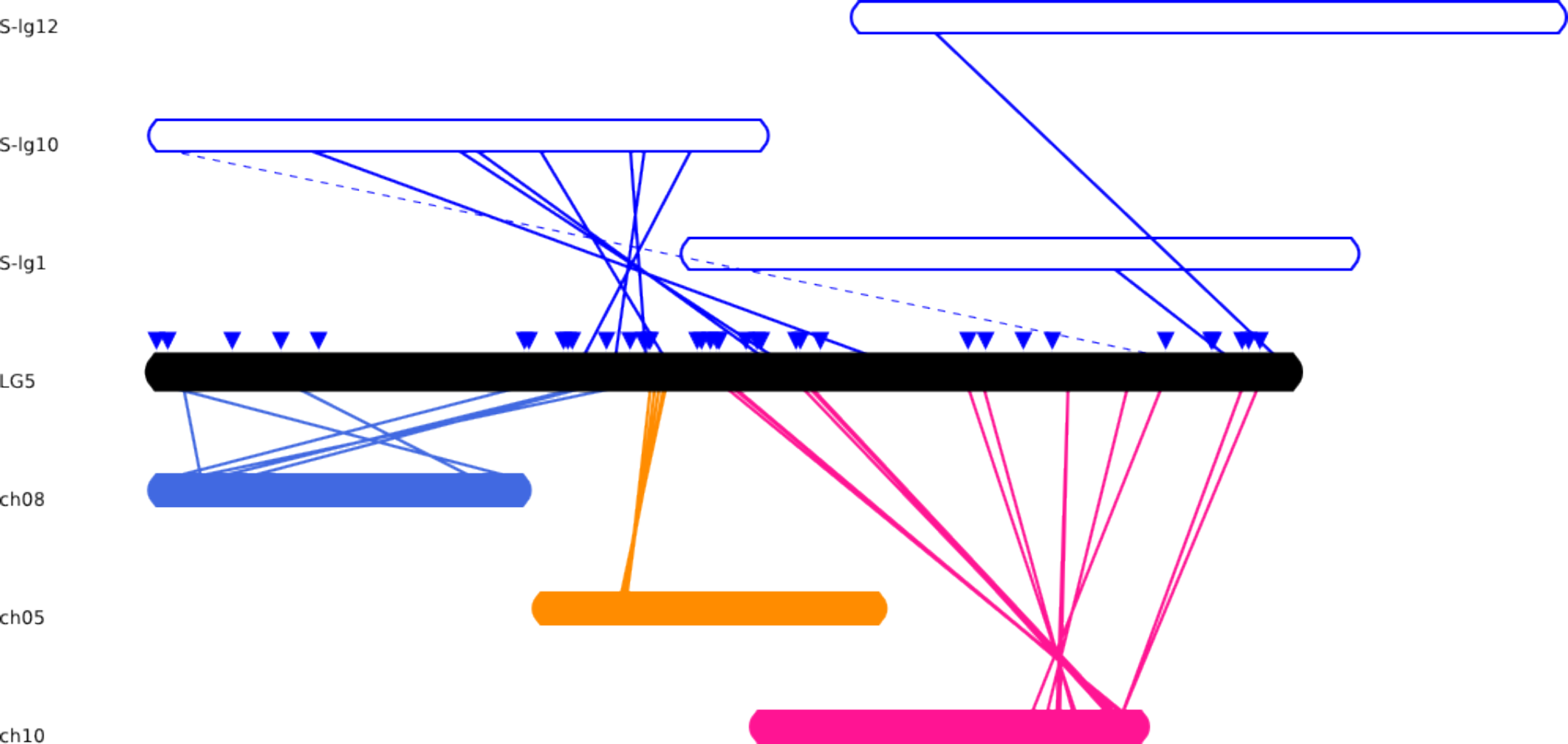

S-Ig5

S-Ig3

LG6

ch11

ch03

ch06

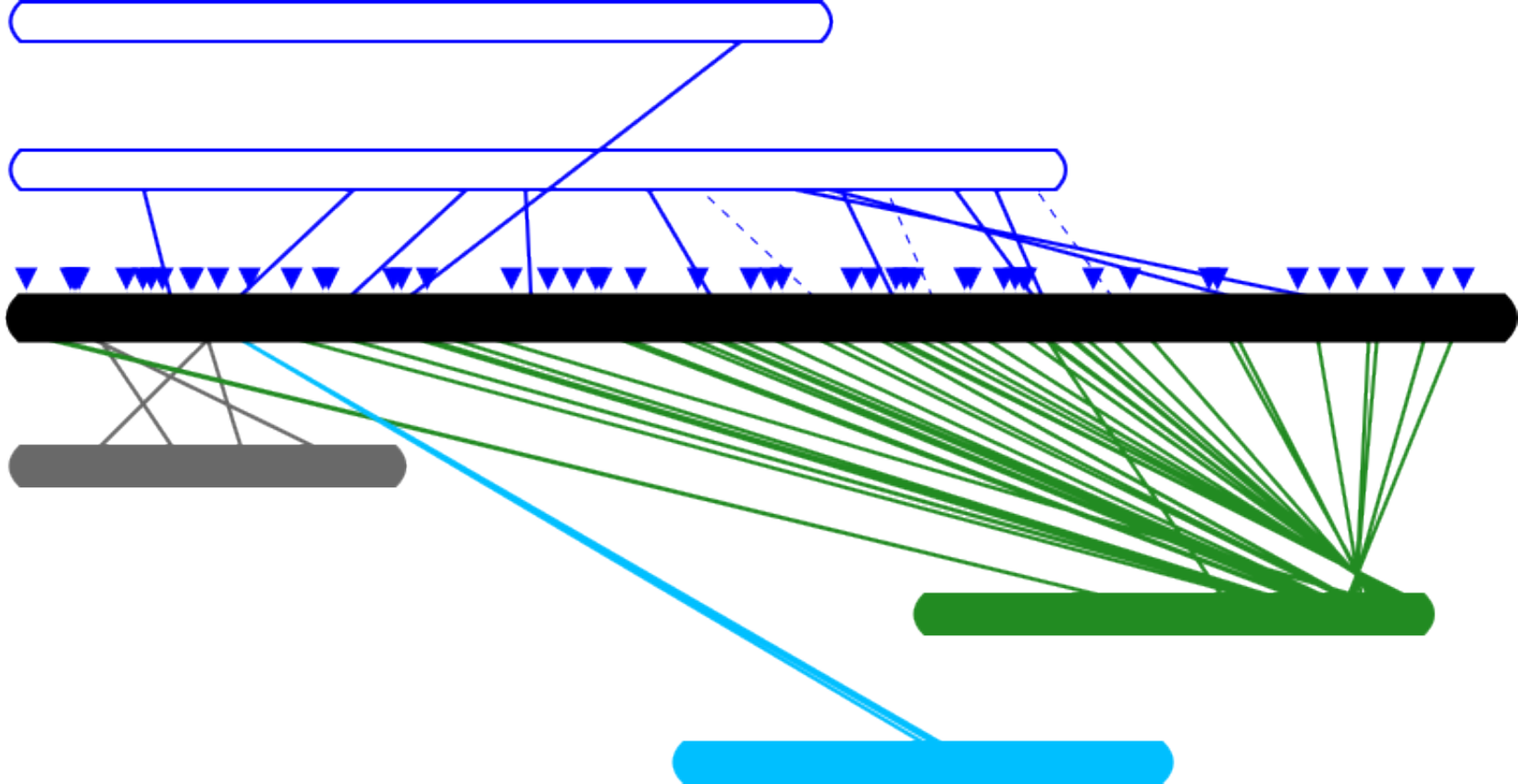

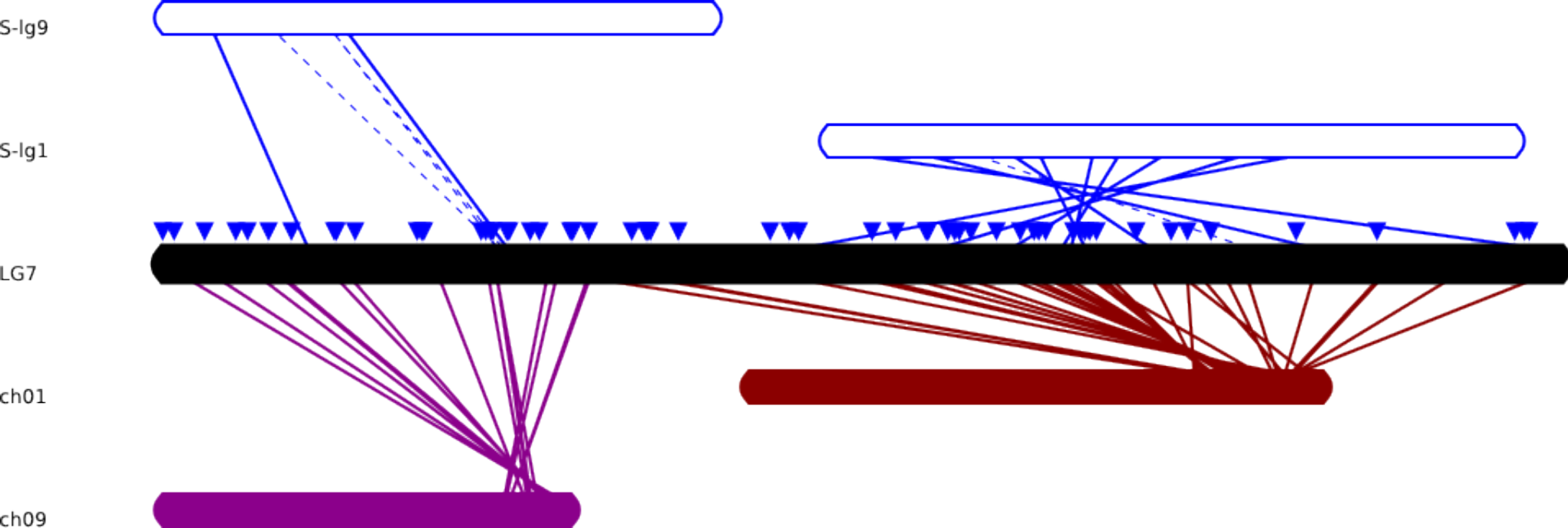

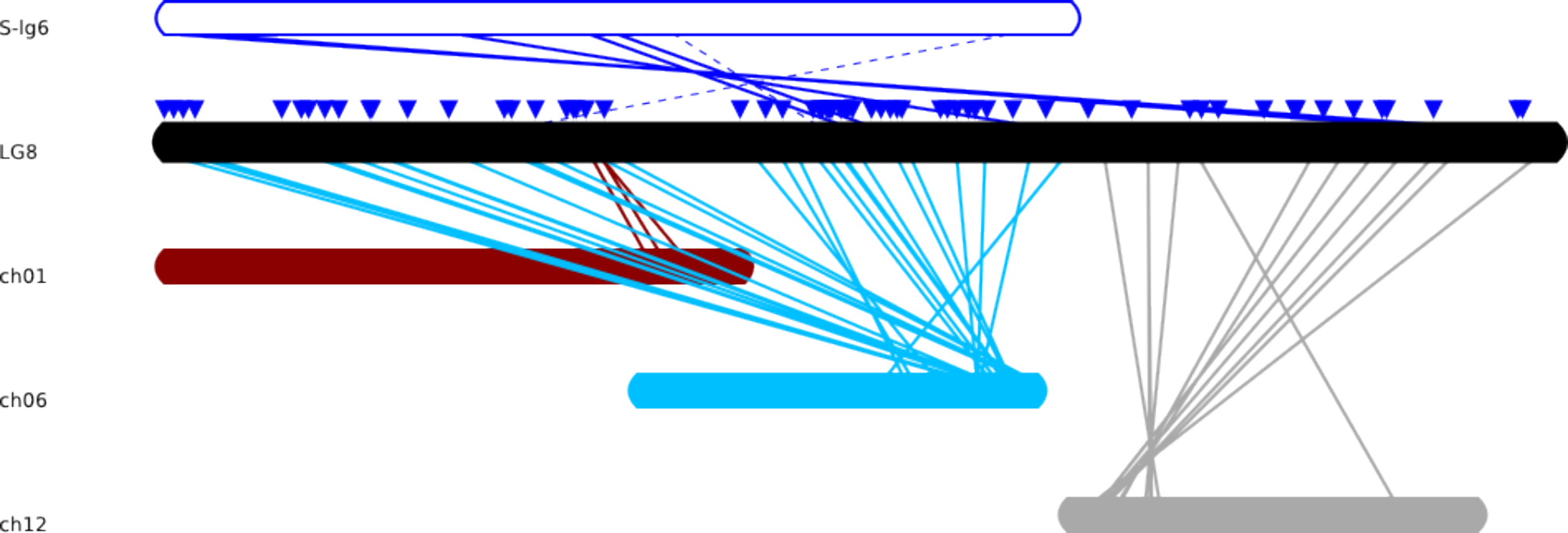

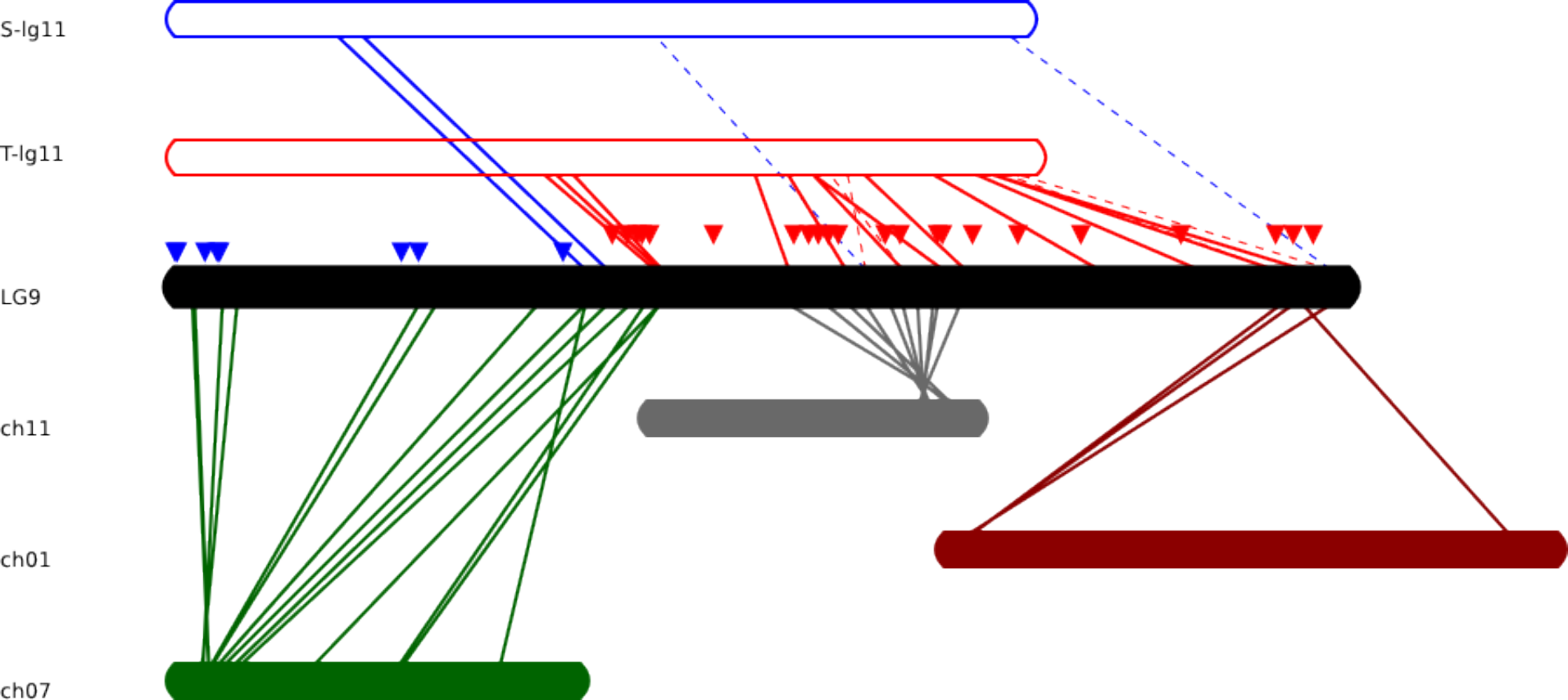

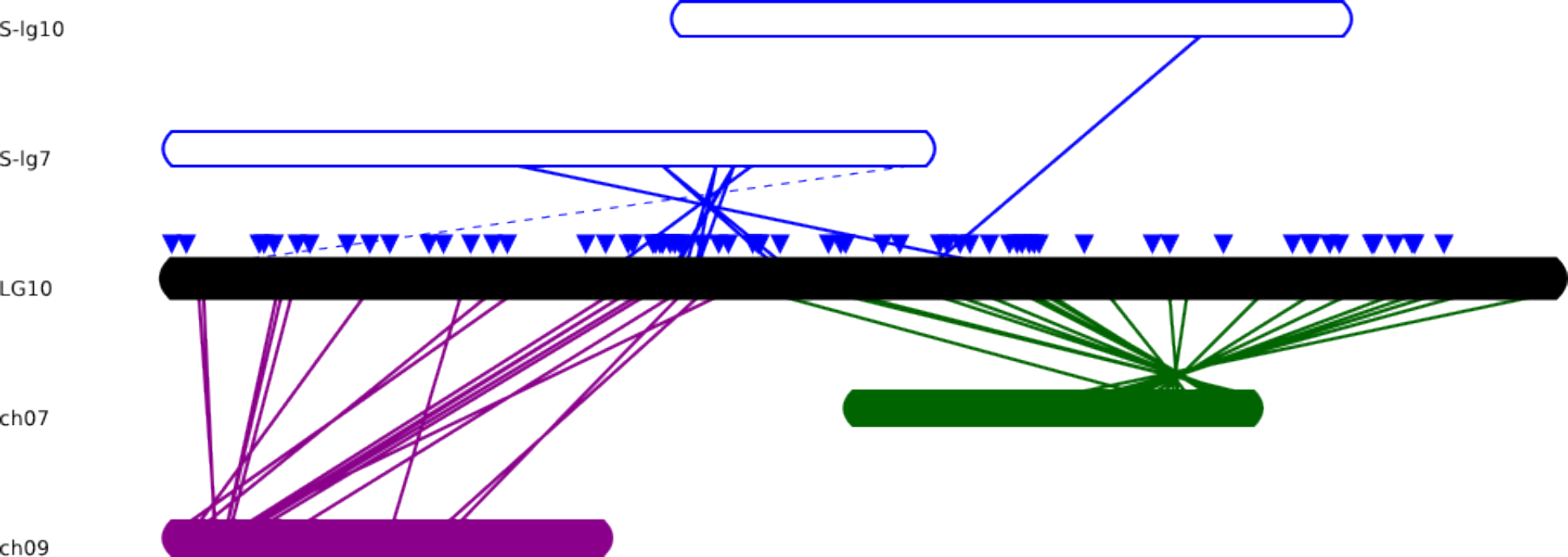

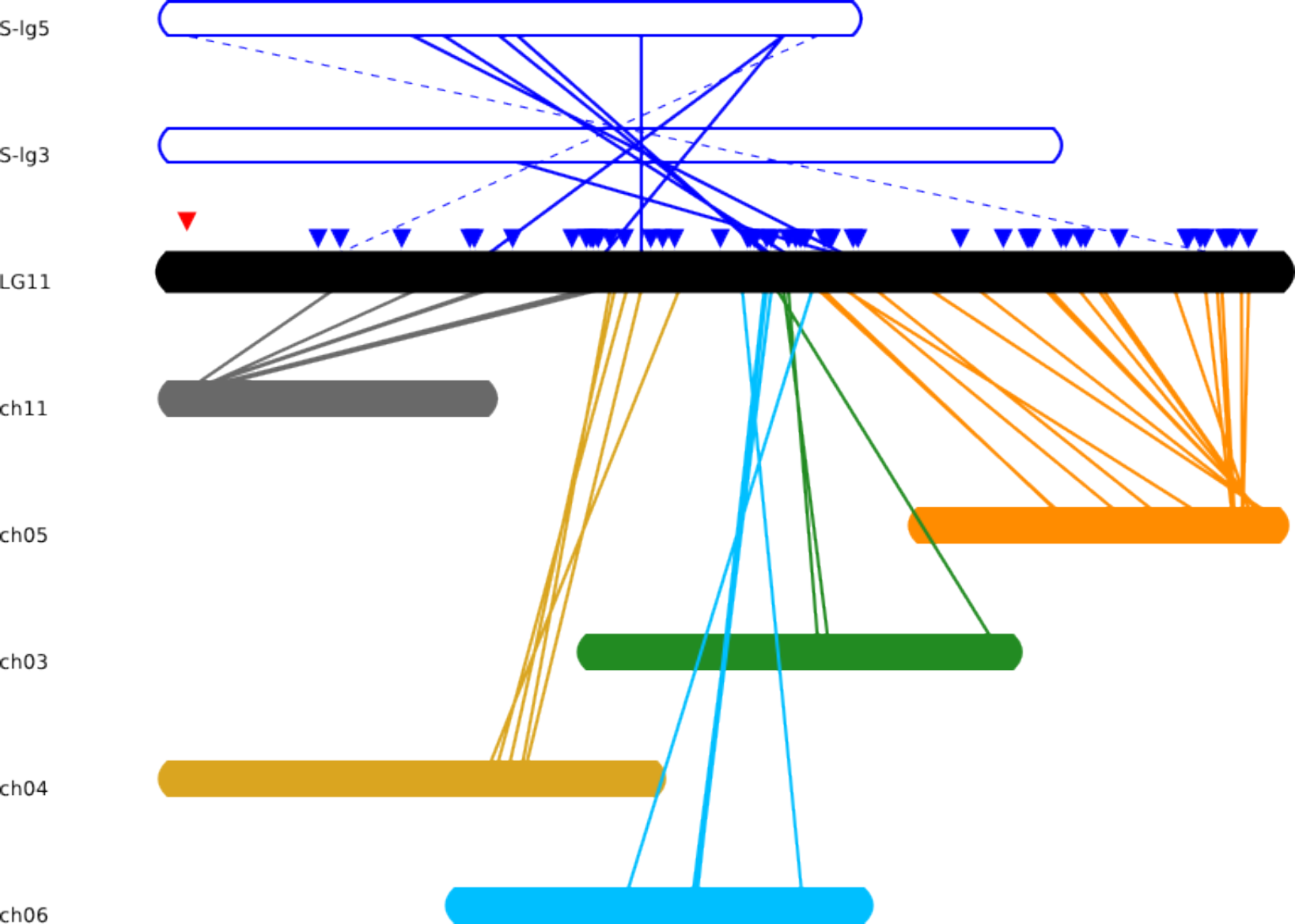

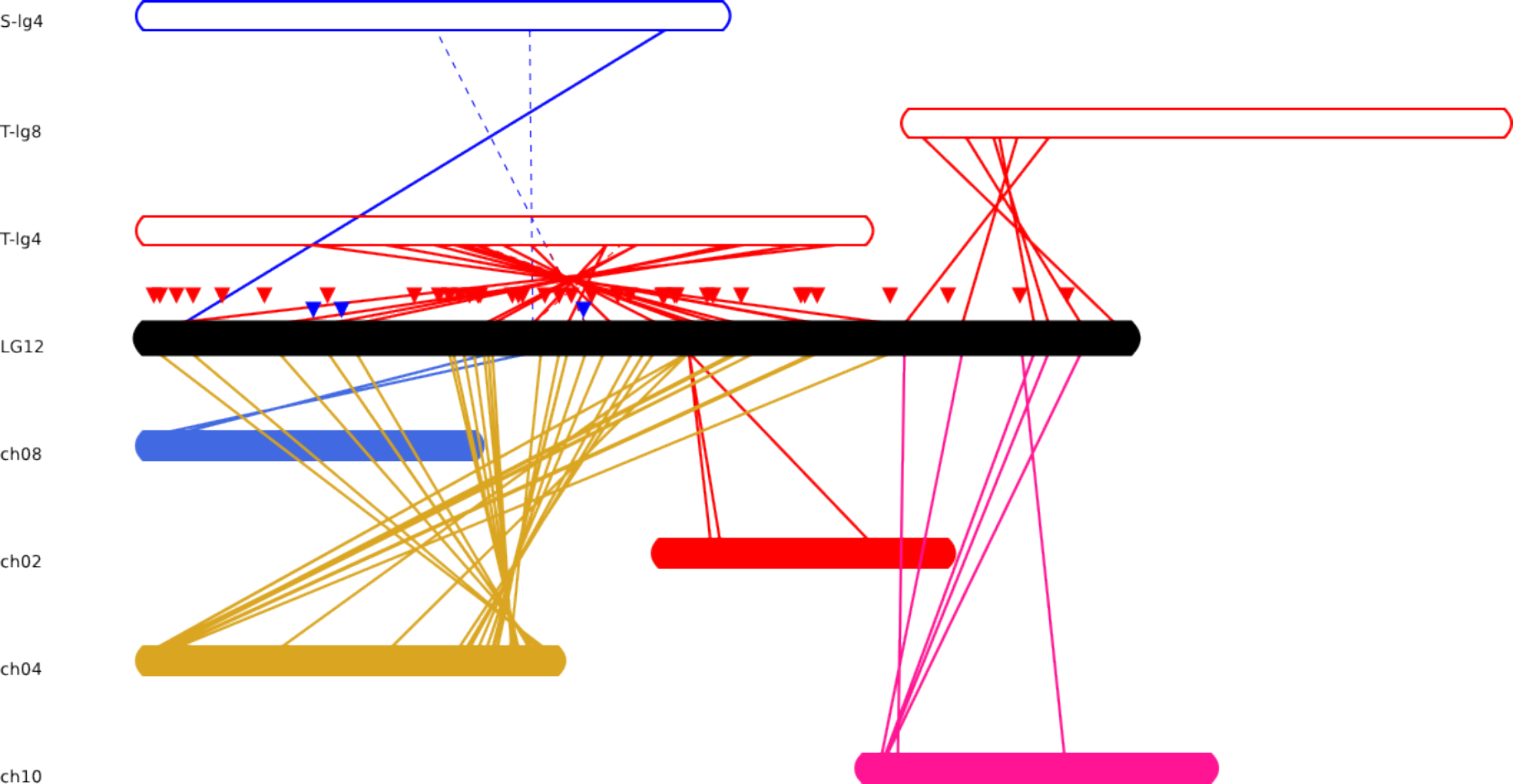

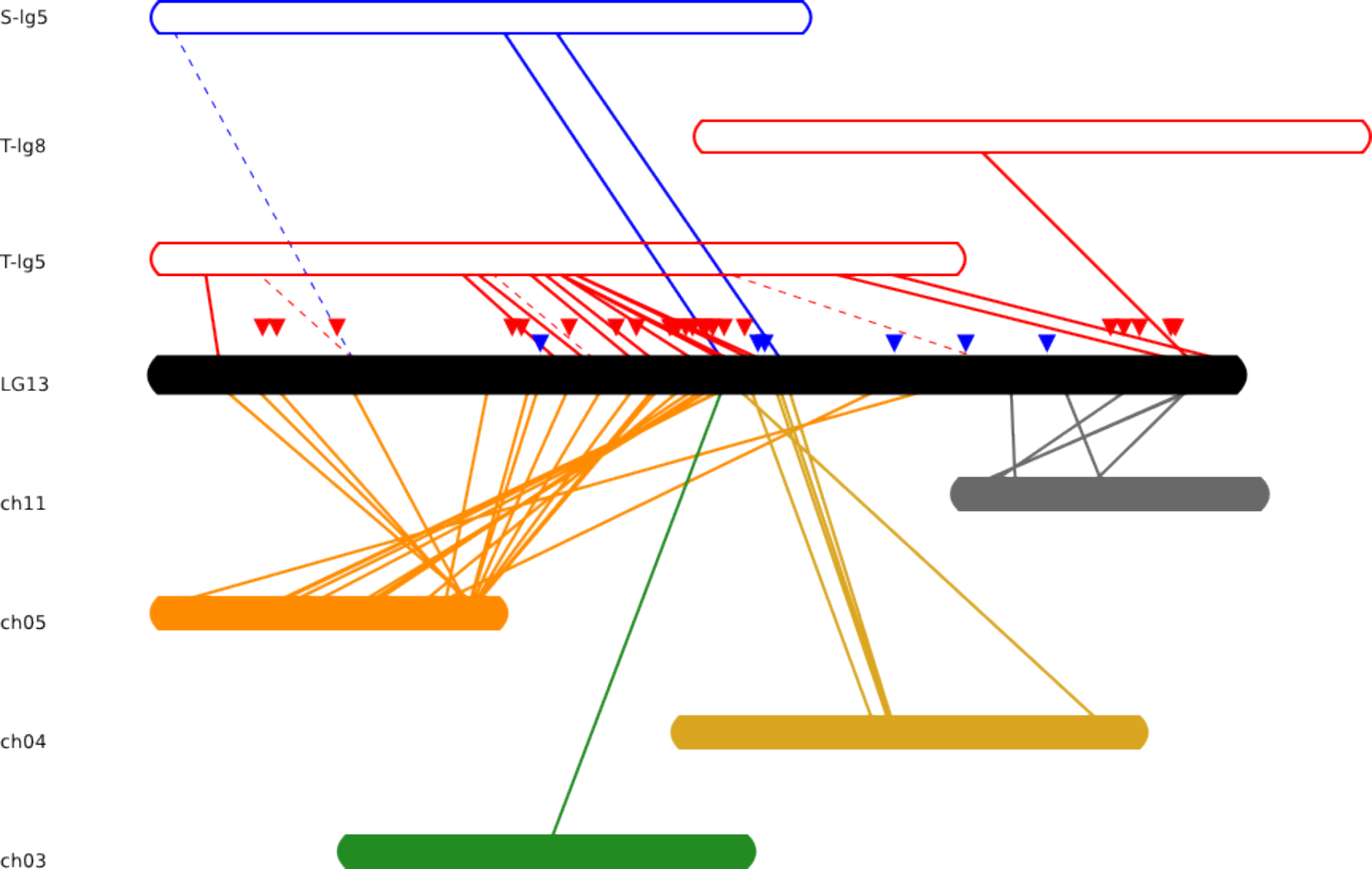

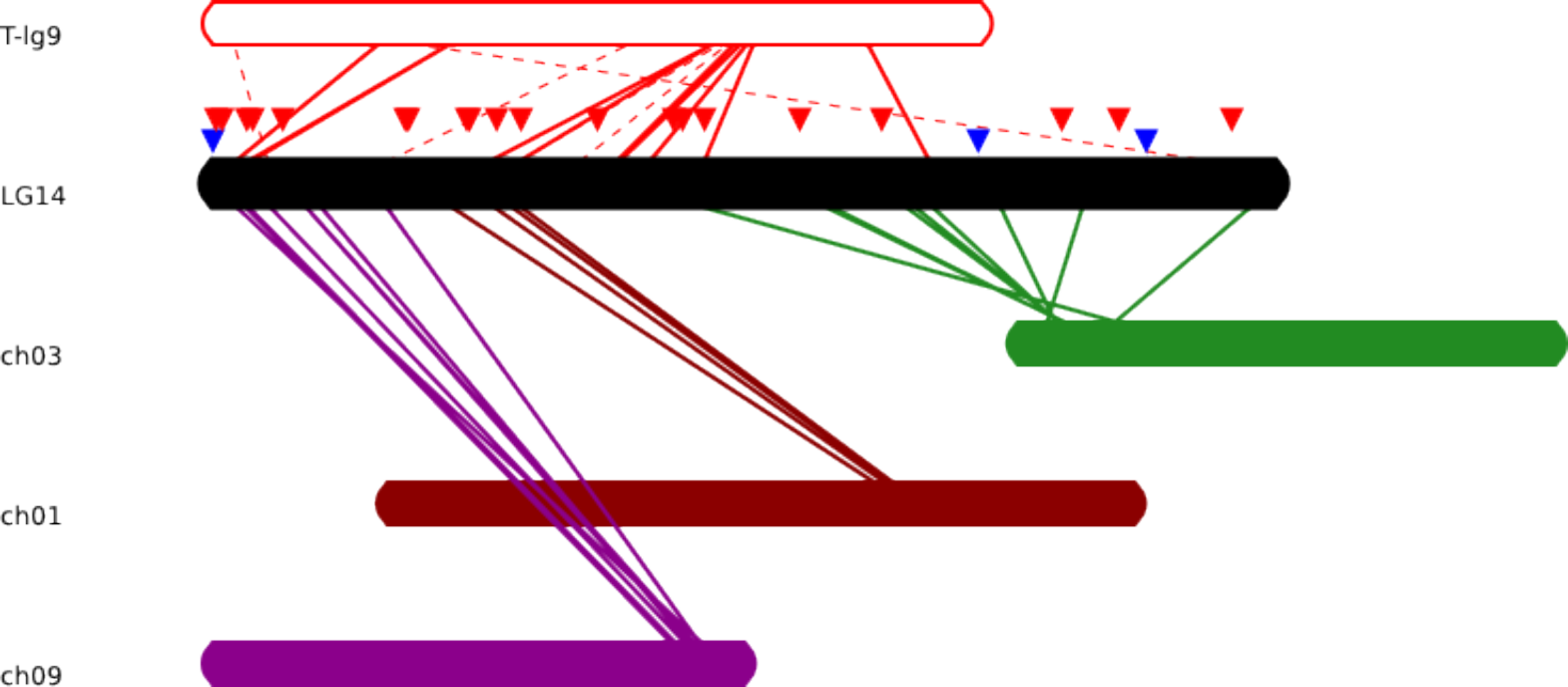

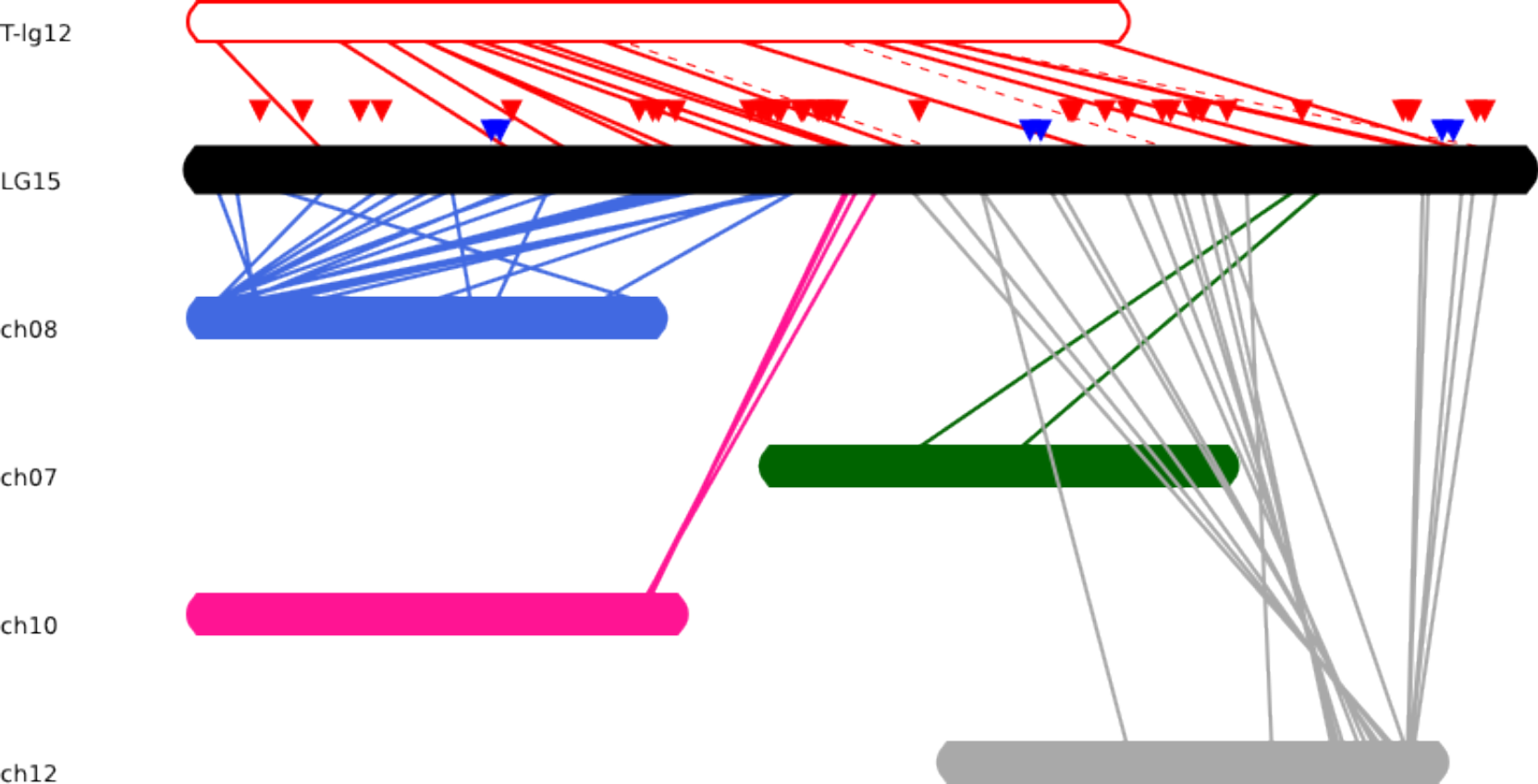

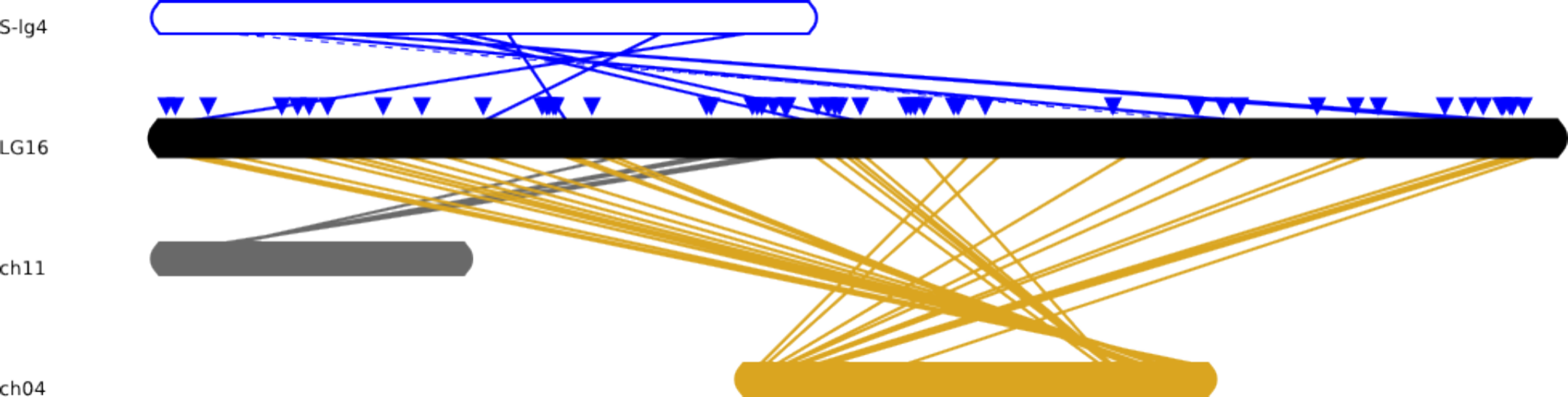

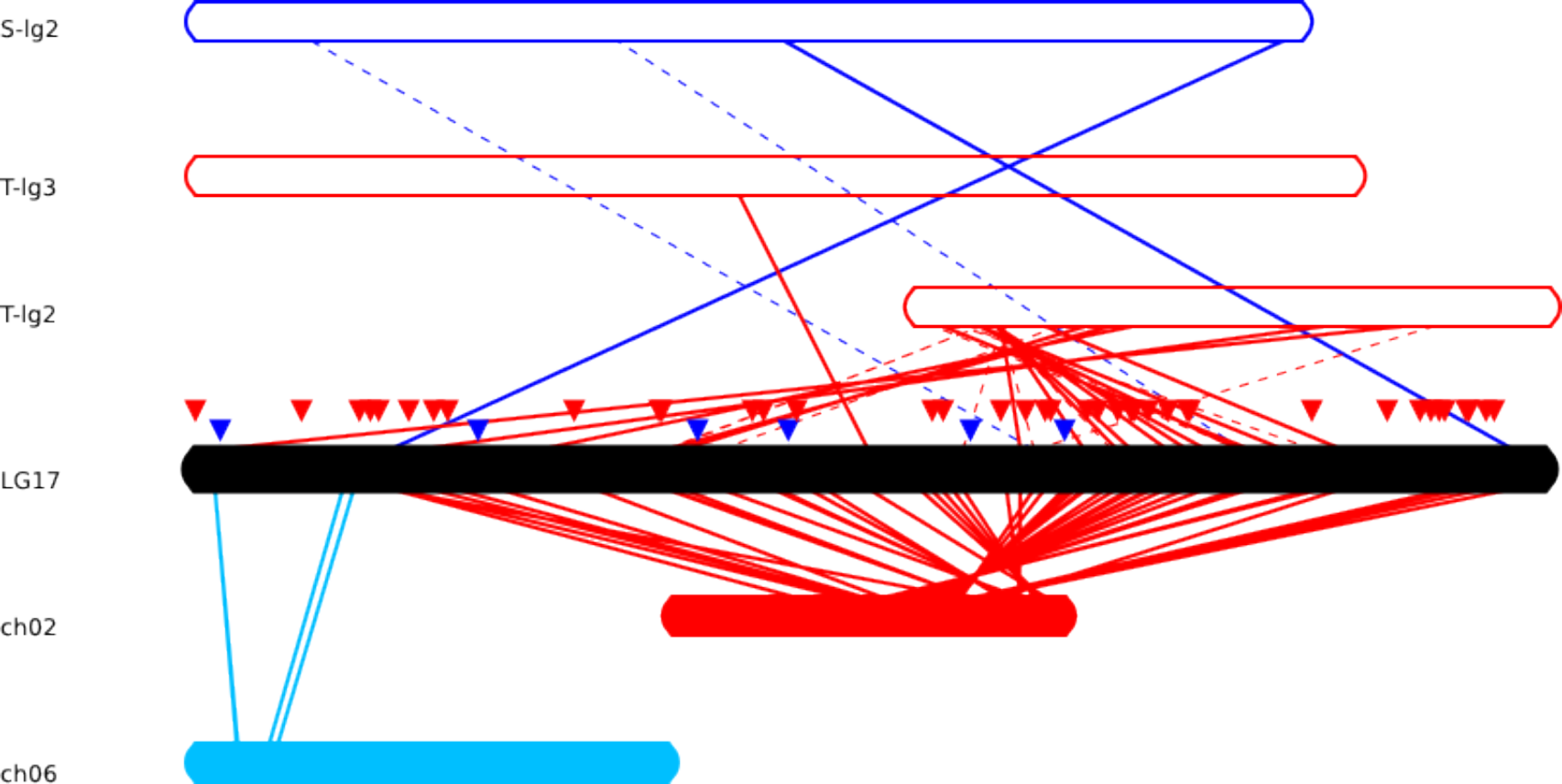

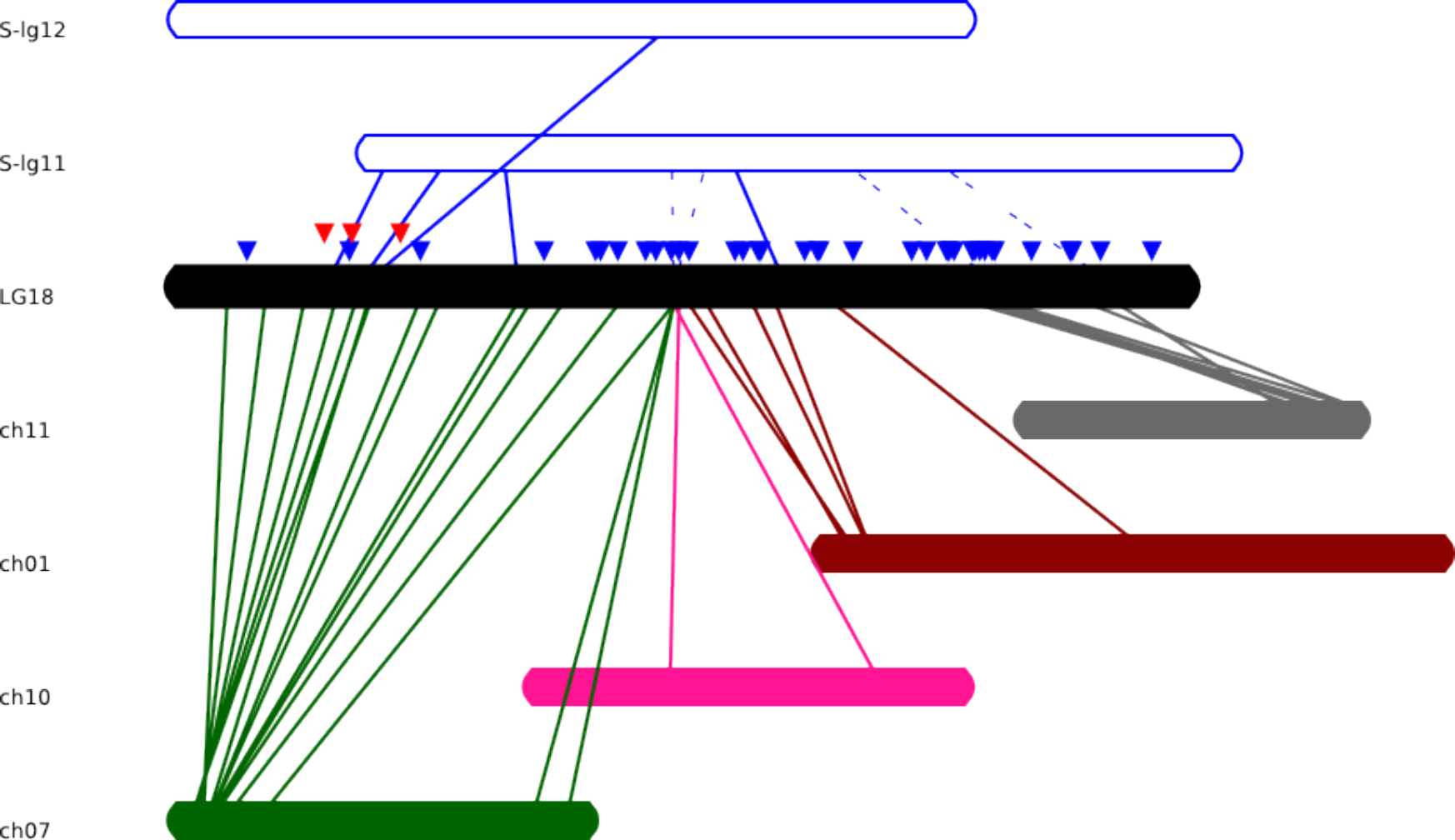

S-Ig1

T-Ig1

LG19

ch01

ch12

ch09

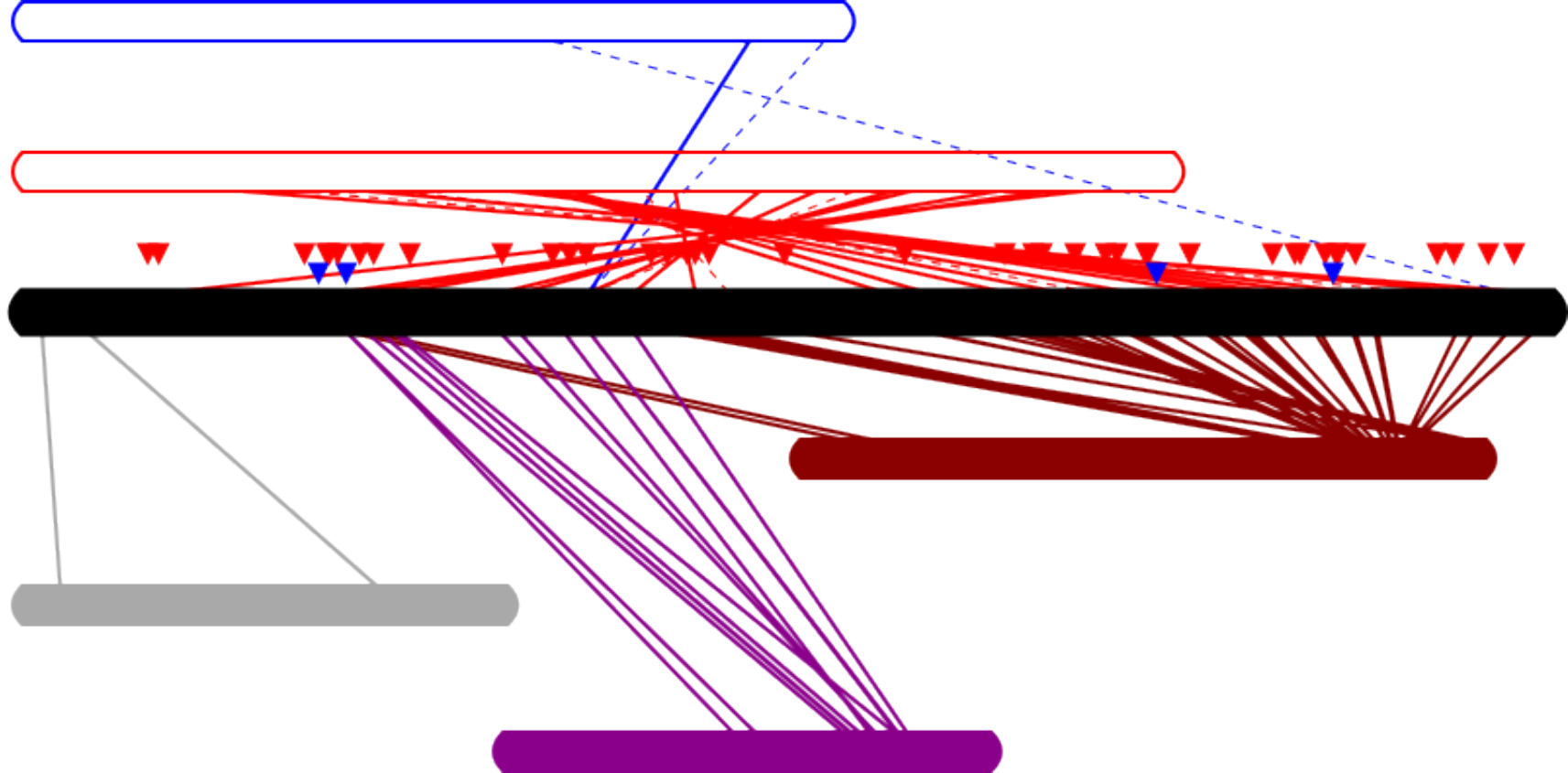

S-Ig12

LG20

ch05

ch12

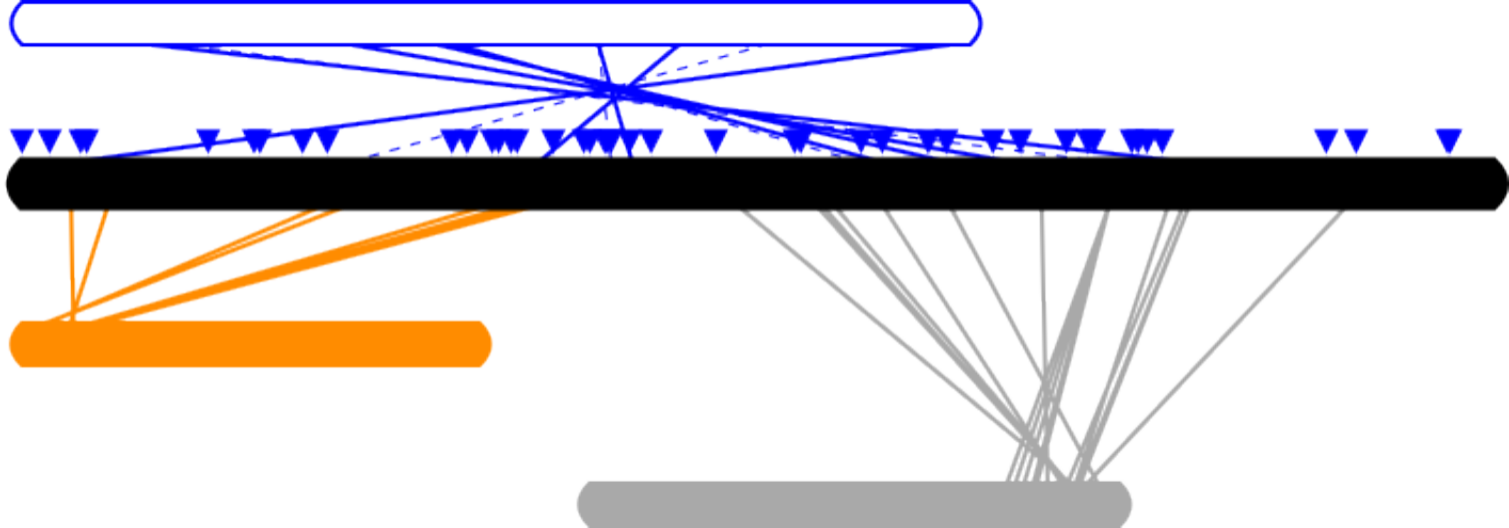

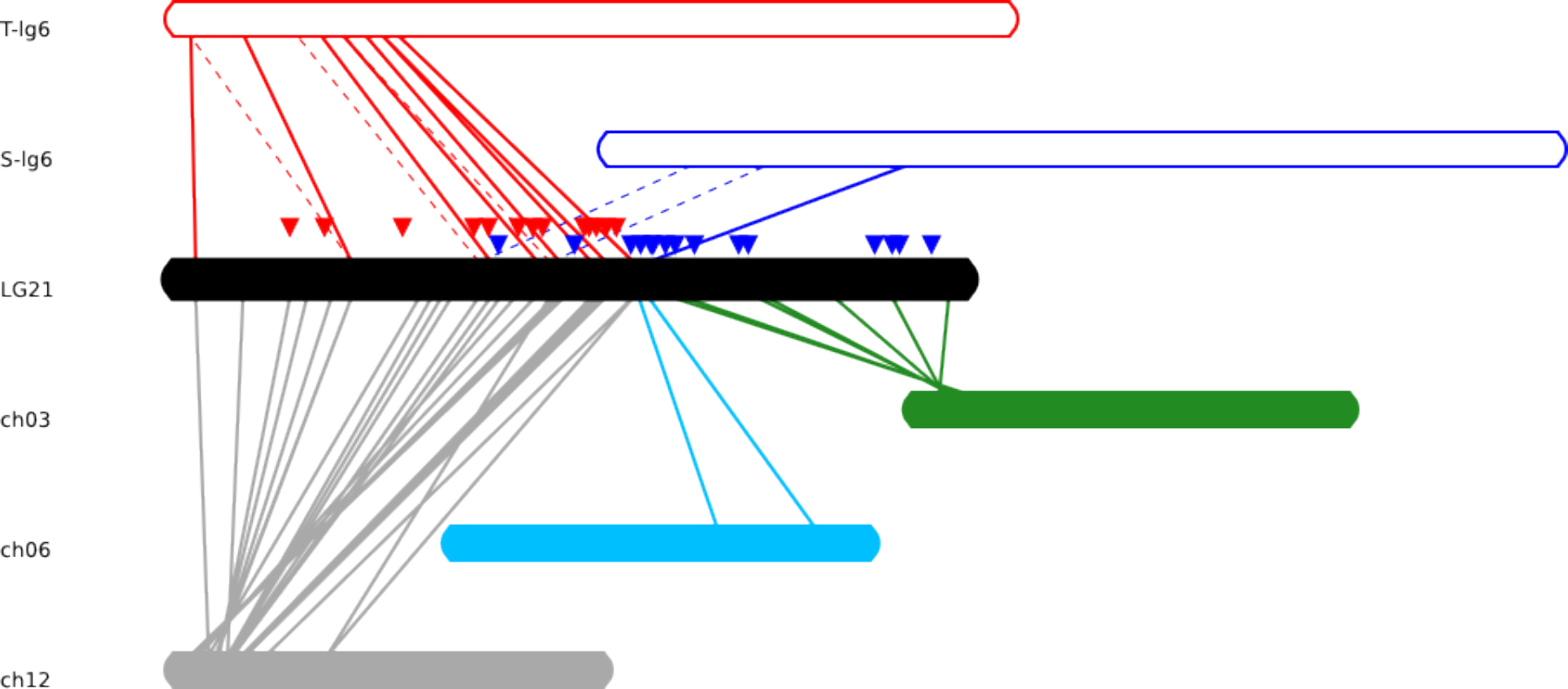

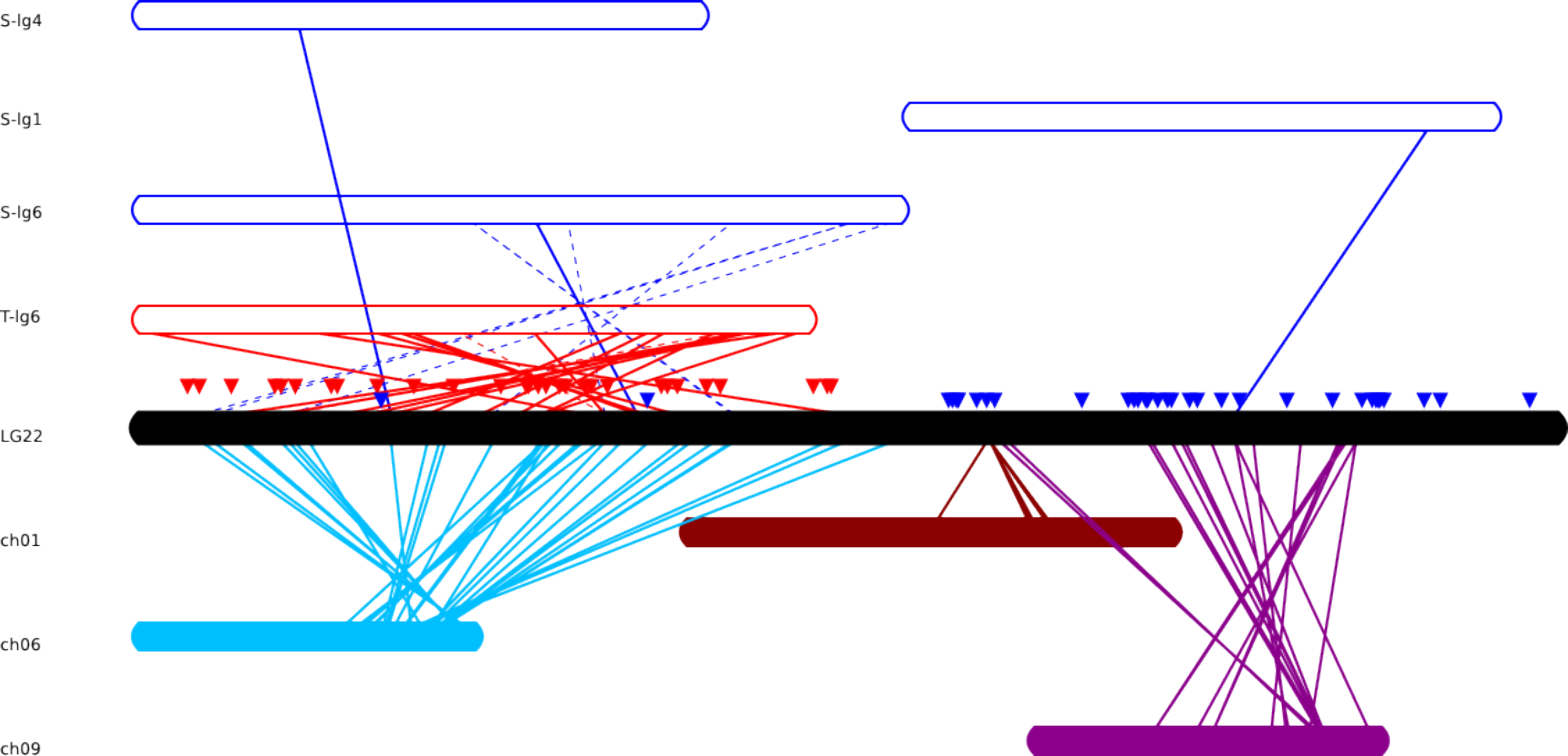

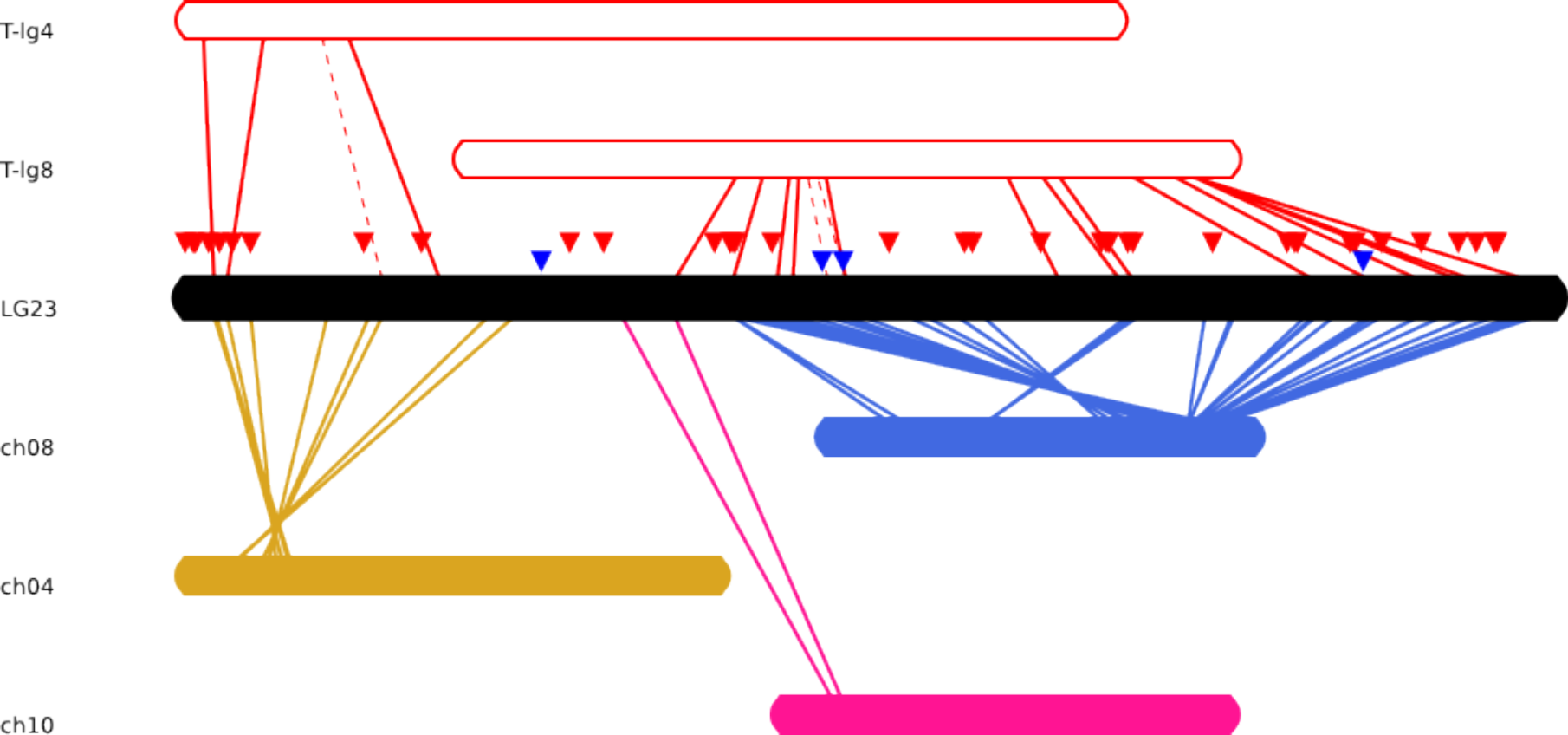

S-Ig1

T-Ig10

LG24

ch05

ch10

ch12

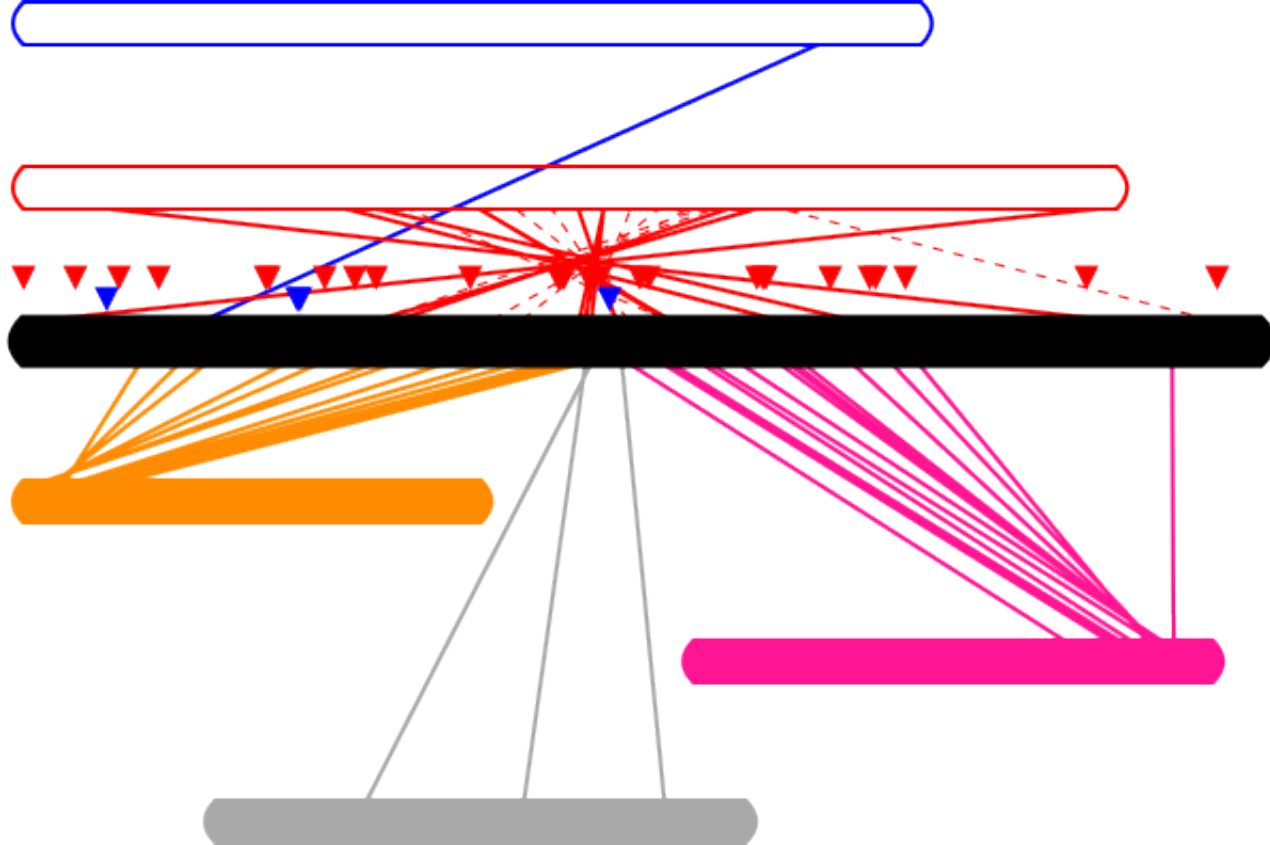

Supplement: Supplementary Data 3 — Synteny of the 24 Nicotiana tabacum linkage groups with the 12 potato chromosomes, based on potato protein mapping. [file ncomms4833-s4.pdf]

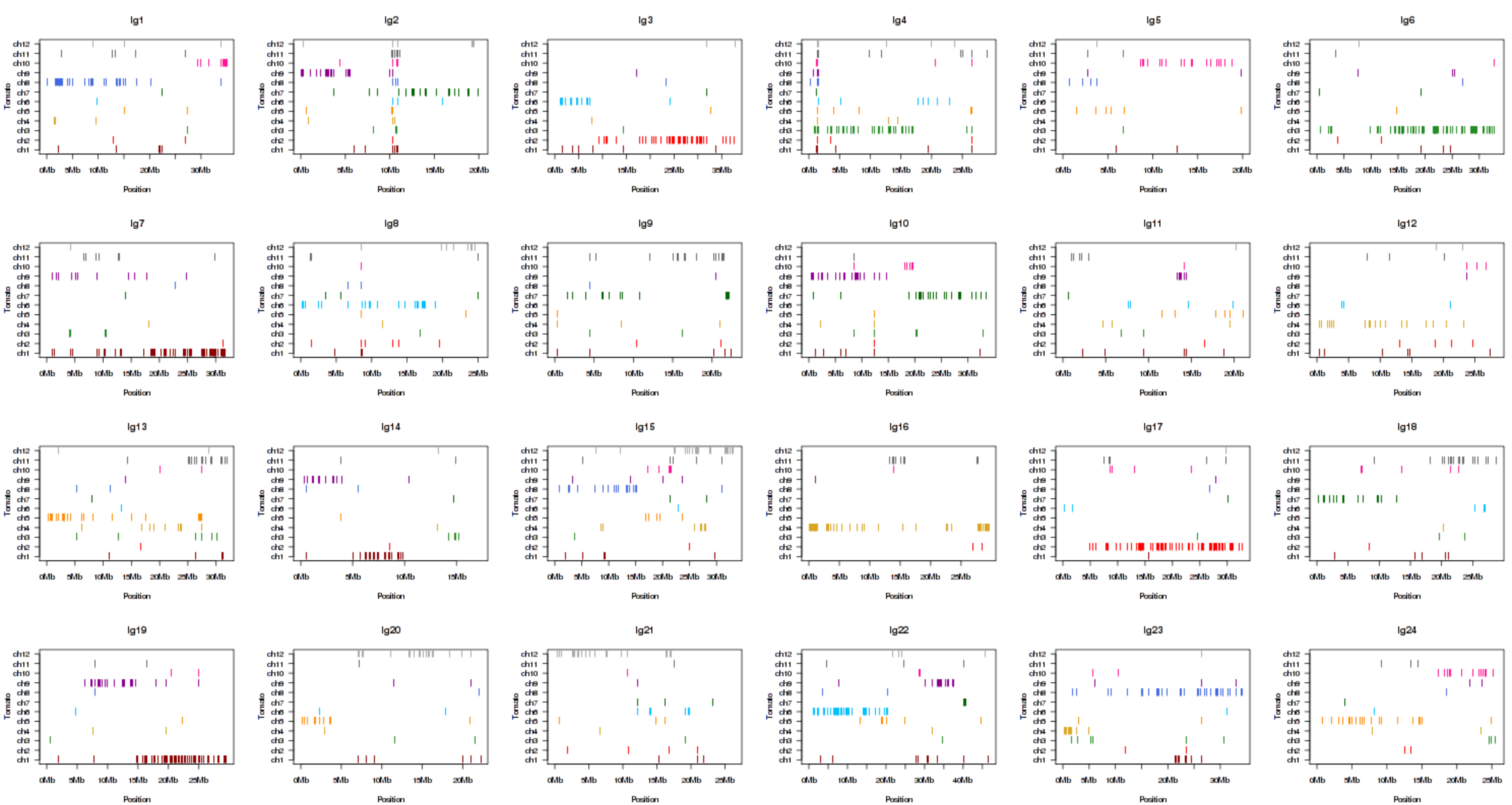

Supplement: Supplementary Data 5 — Synteny of the 24 Nicotiana tabacum linkage groups with the 12 tomato chromosomes, based on whole genome sequences. Syntenic DNA blocks in each plot are positioned on the x axis according to their location in the Nicotiana tabacum linkage group. Each line on the y axis represents one tomato chromosome. [file ncomms4833-s6.pdf]

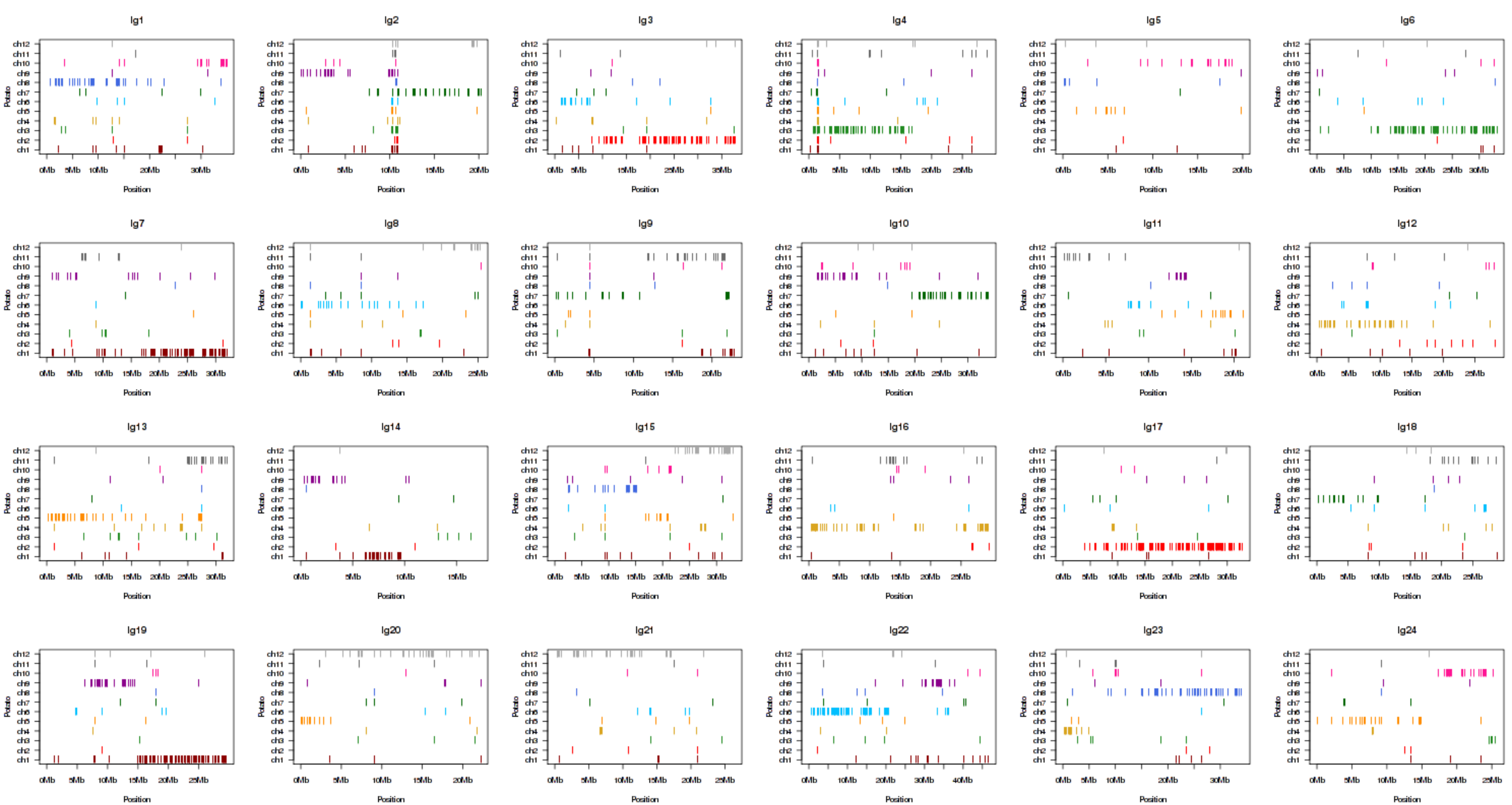

Supplement: Supplementary Data 7 — Synteny of the 24 Nicotiana tabacum linkage groups with the 12 potato chromosomes, based on whole genome sequences. Syntenic DNA blocks in each plot are positioned on the x axis according to their location in the Nicotiana tabacum linkage group. Each line on the y axis represents one potato chromosome. [file ncomms4833-s8.pdf]

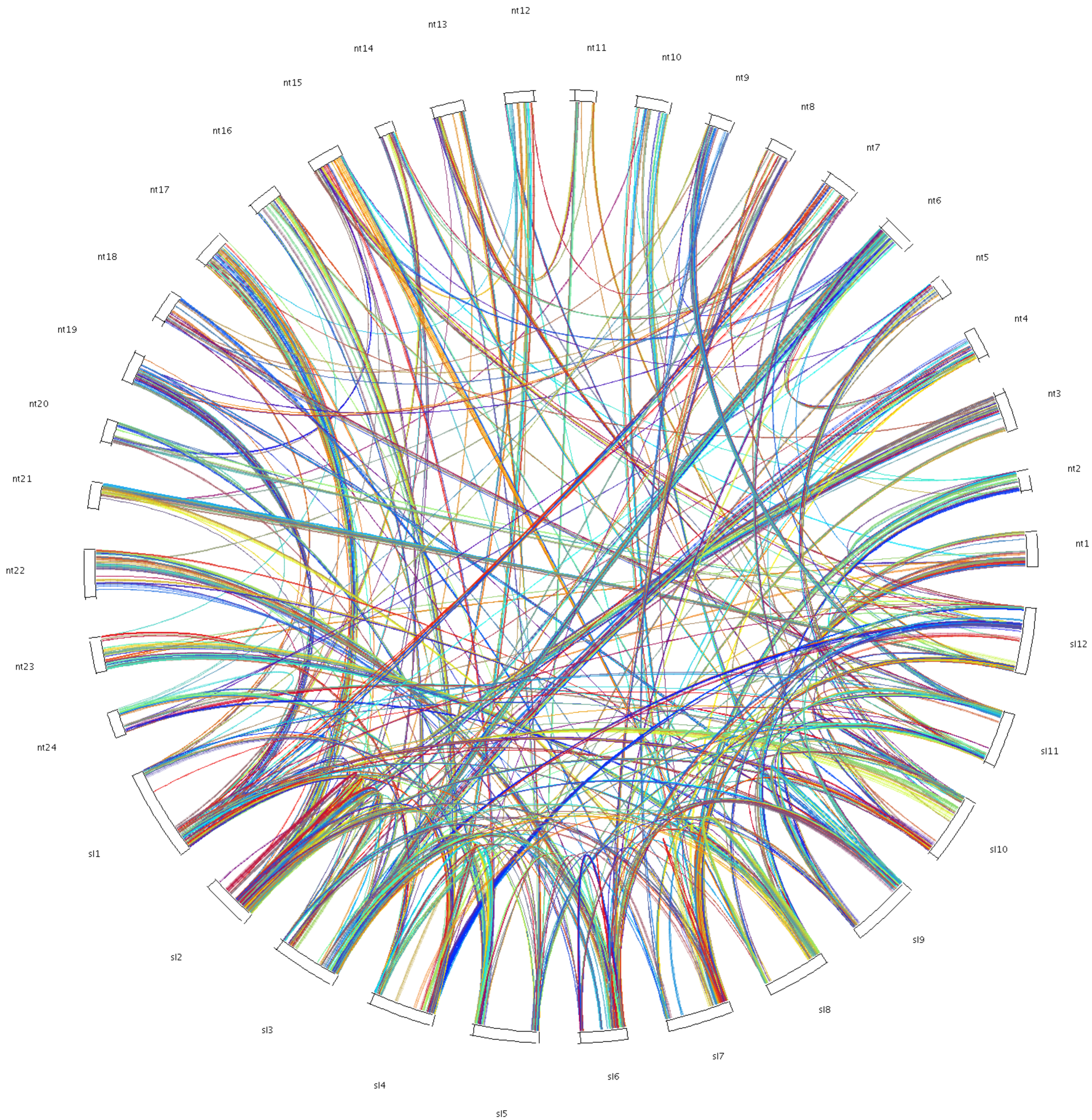

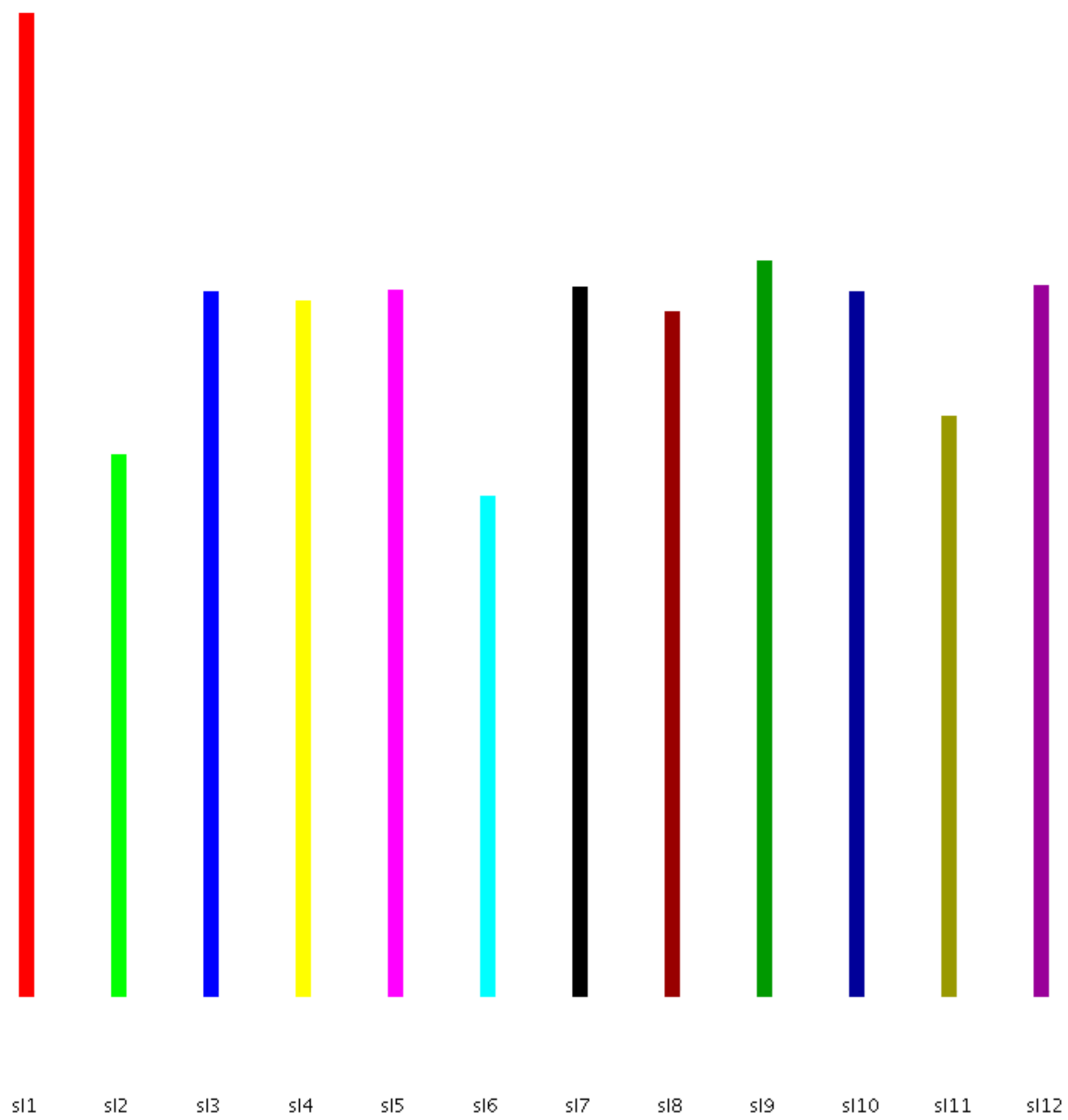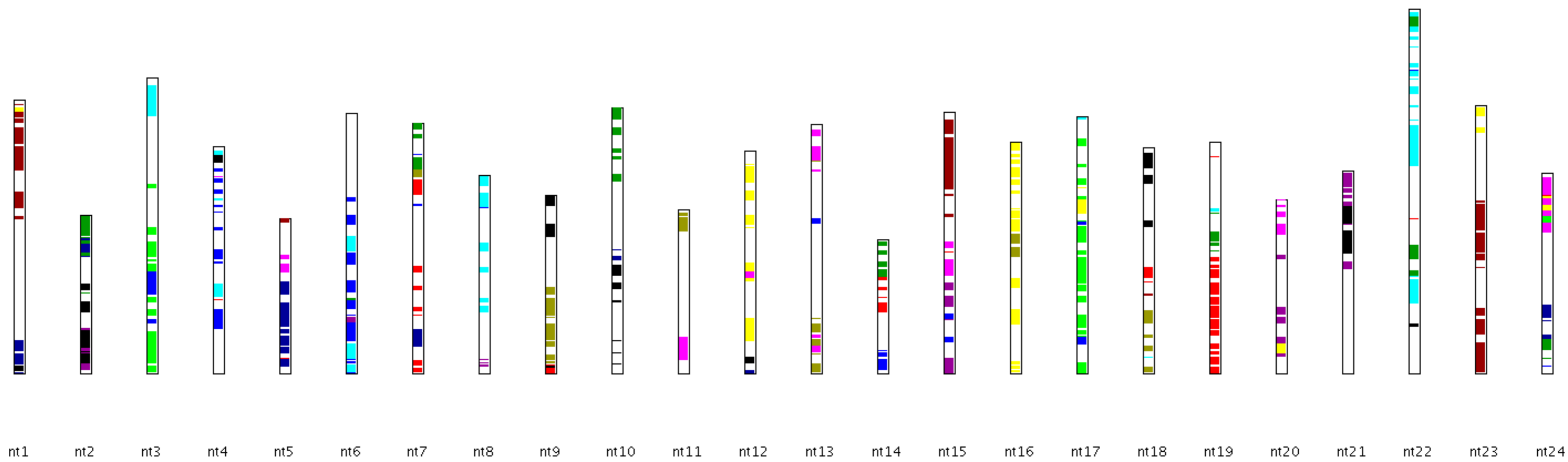

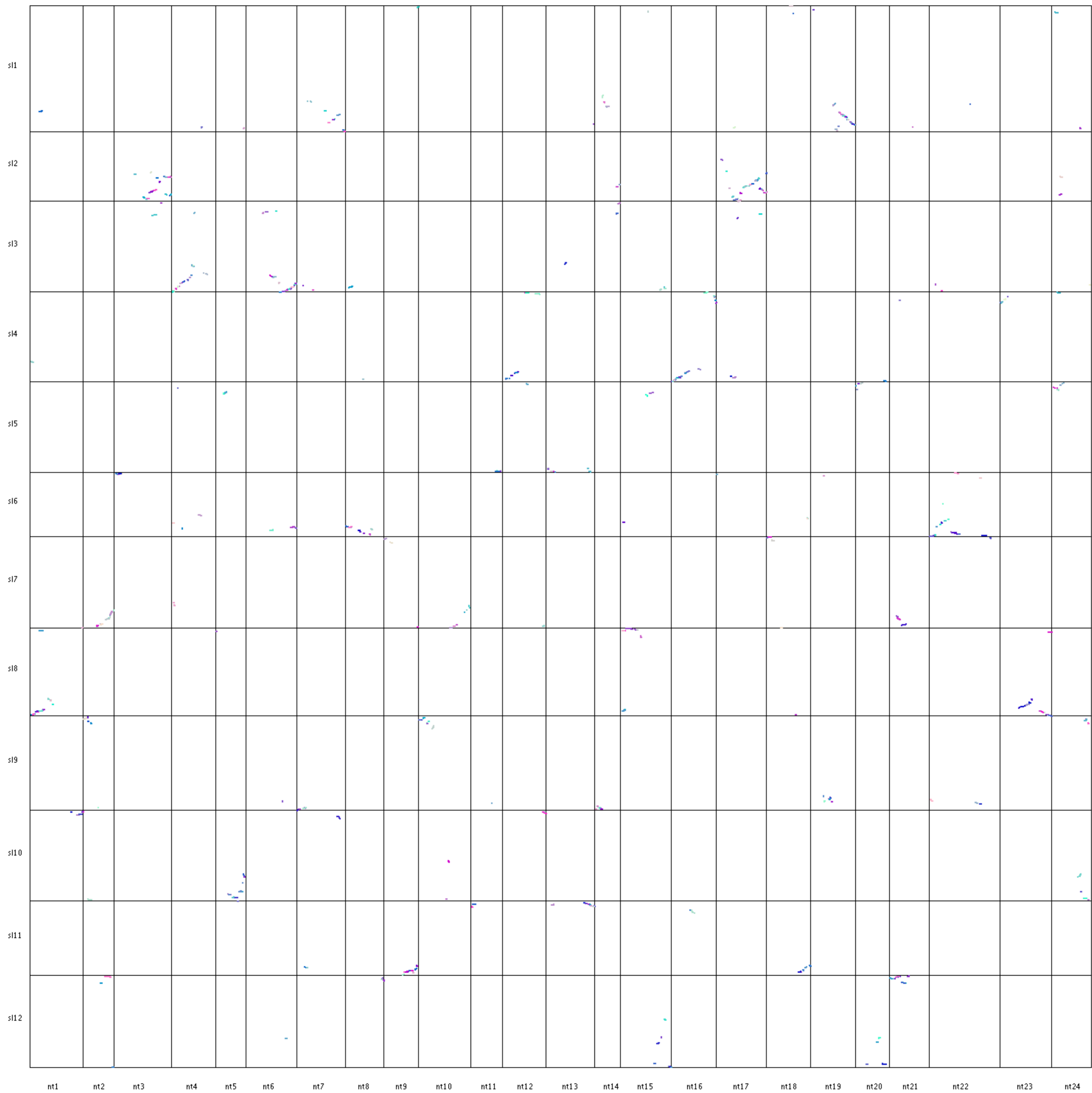

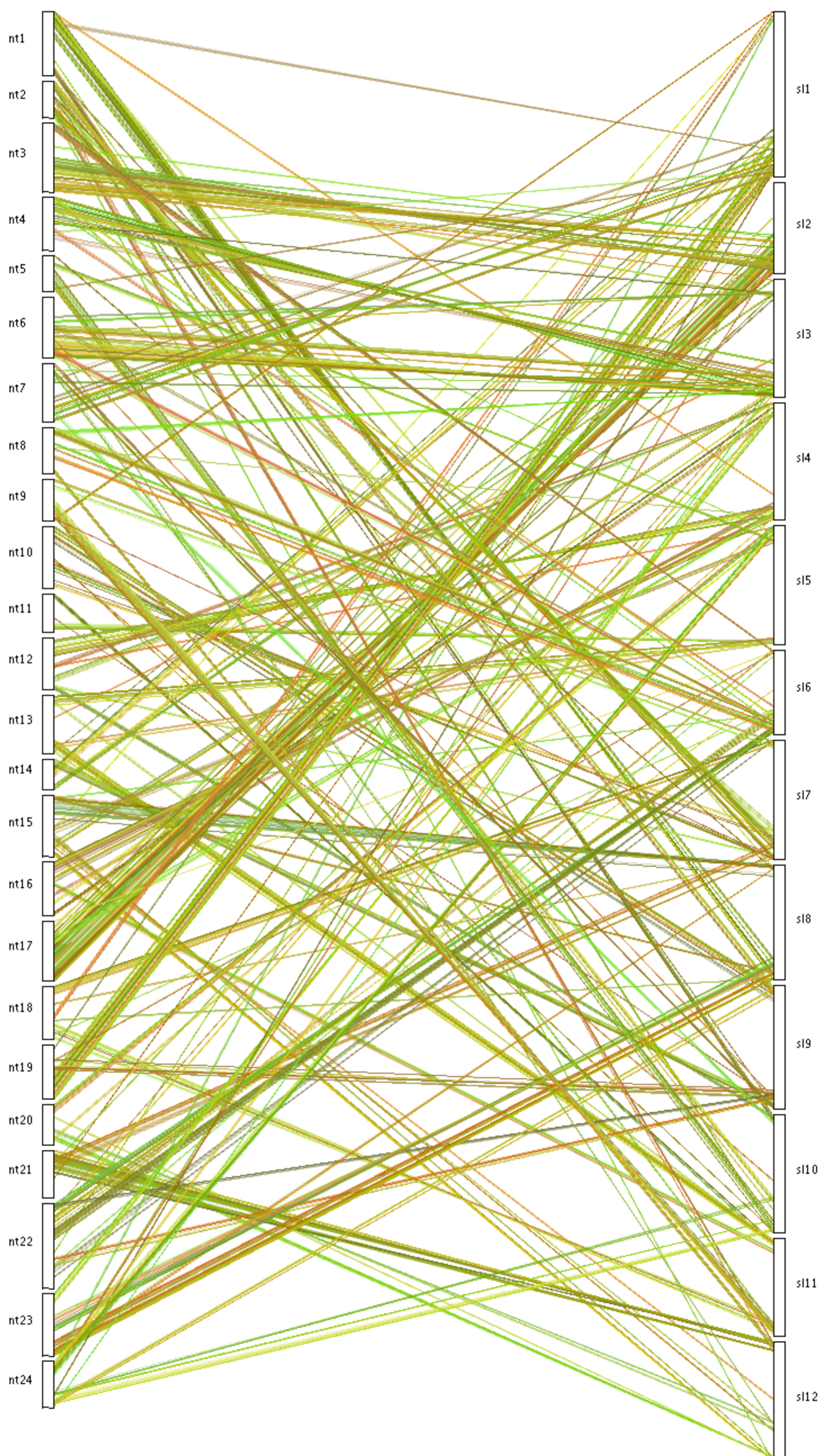

Supplement: Supplementary Data 8 — Synteny of the 24 Nicotiana tabacum linkage groups with the 12 tomato chromosomes determined using MCScanX. [file ncomms4833-s9.pdf]

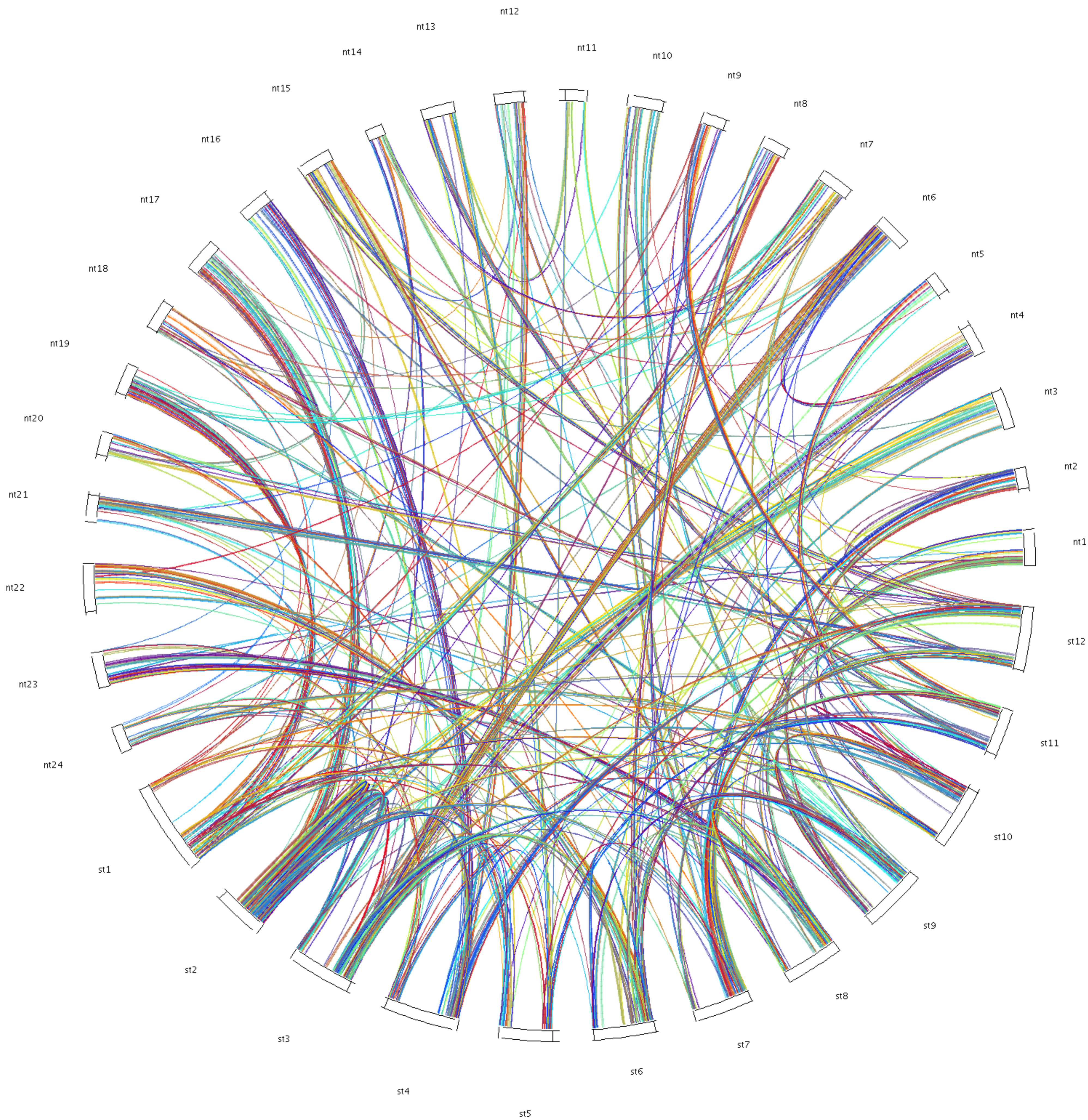

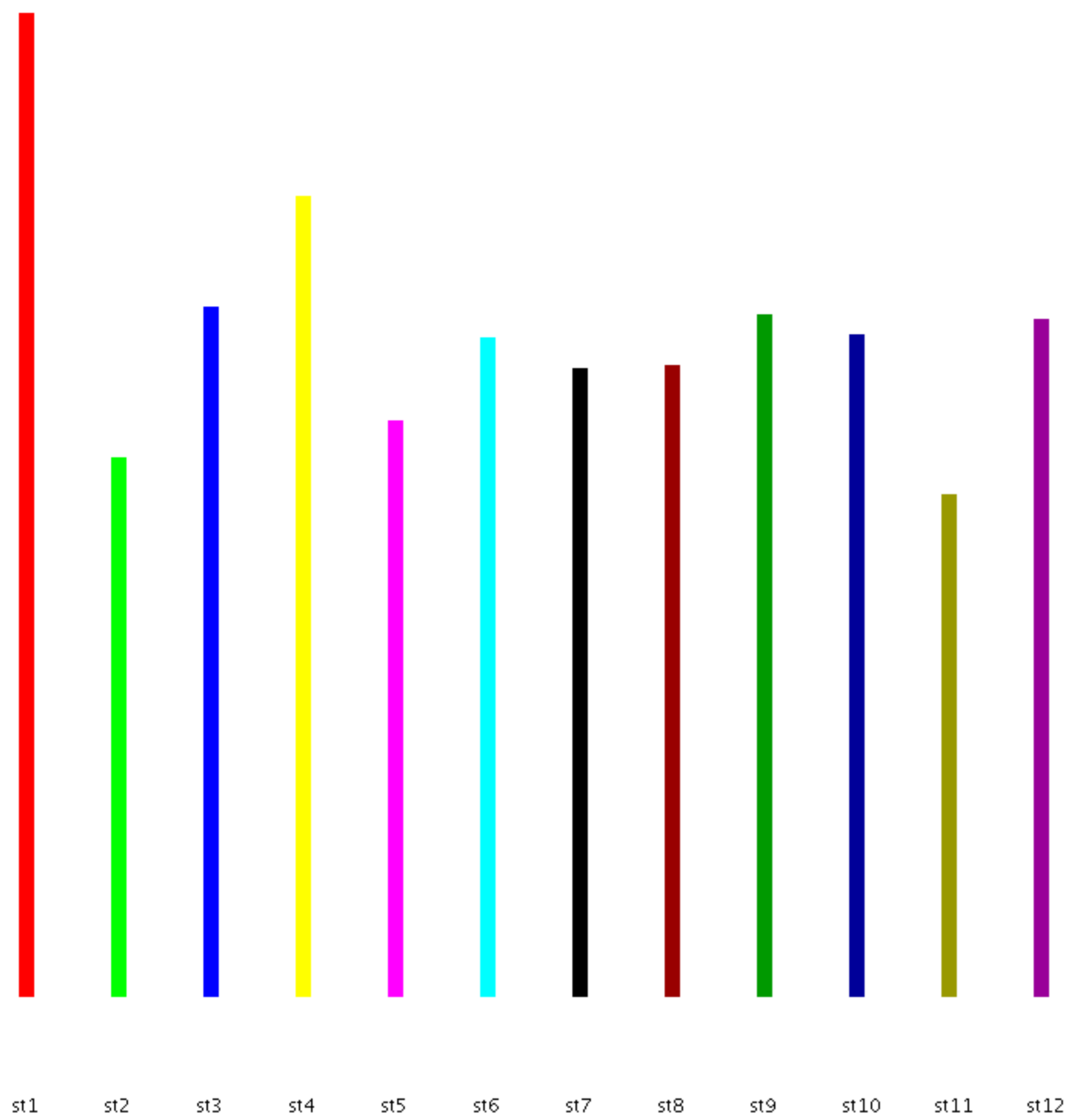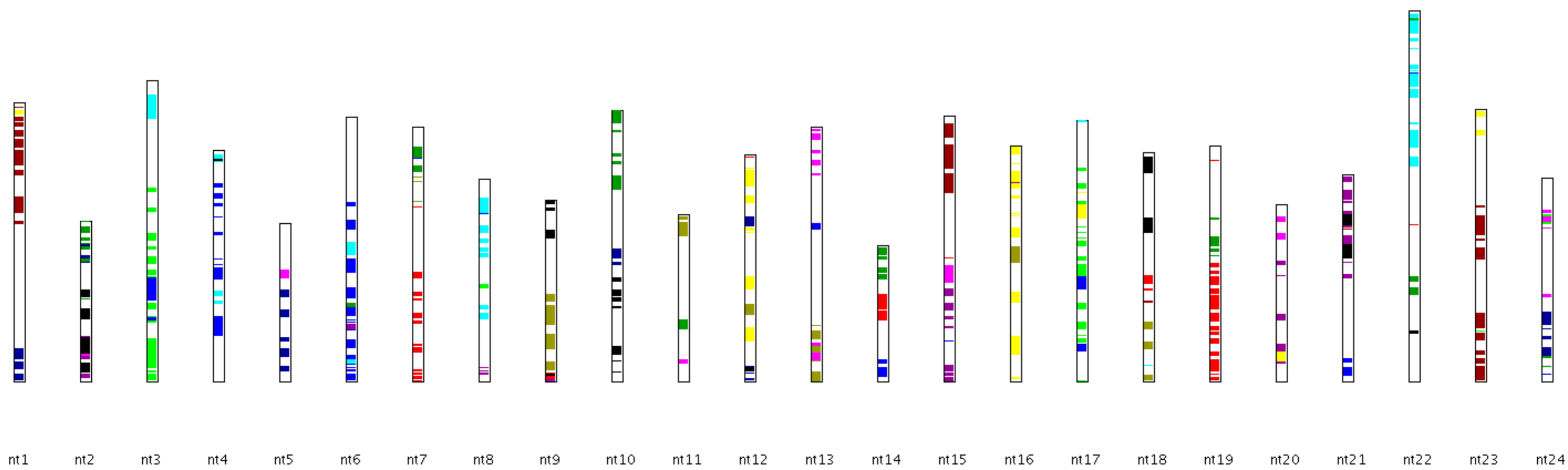

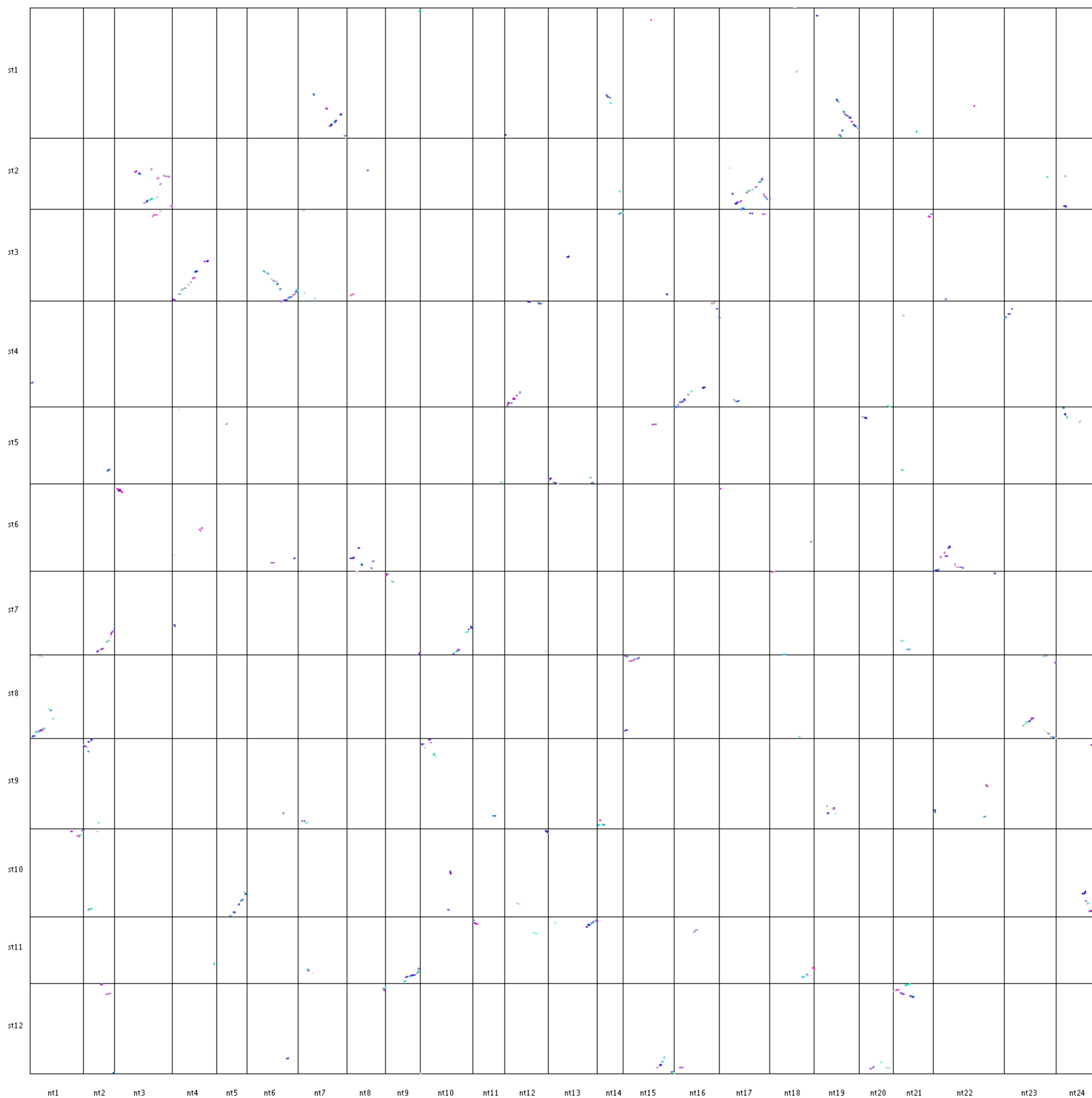

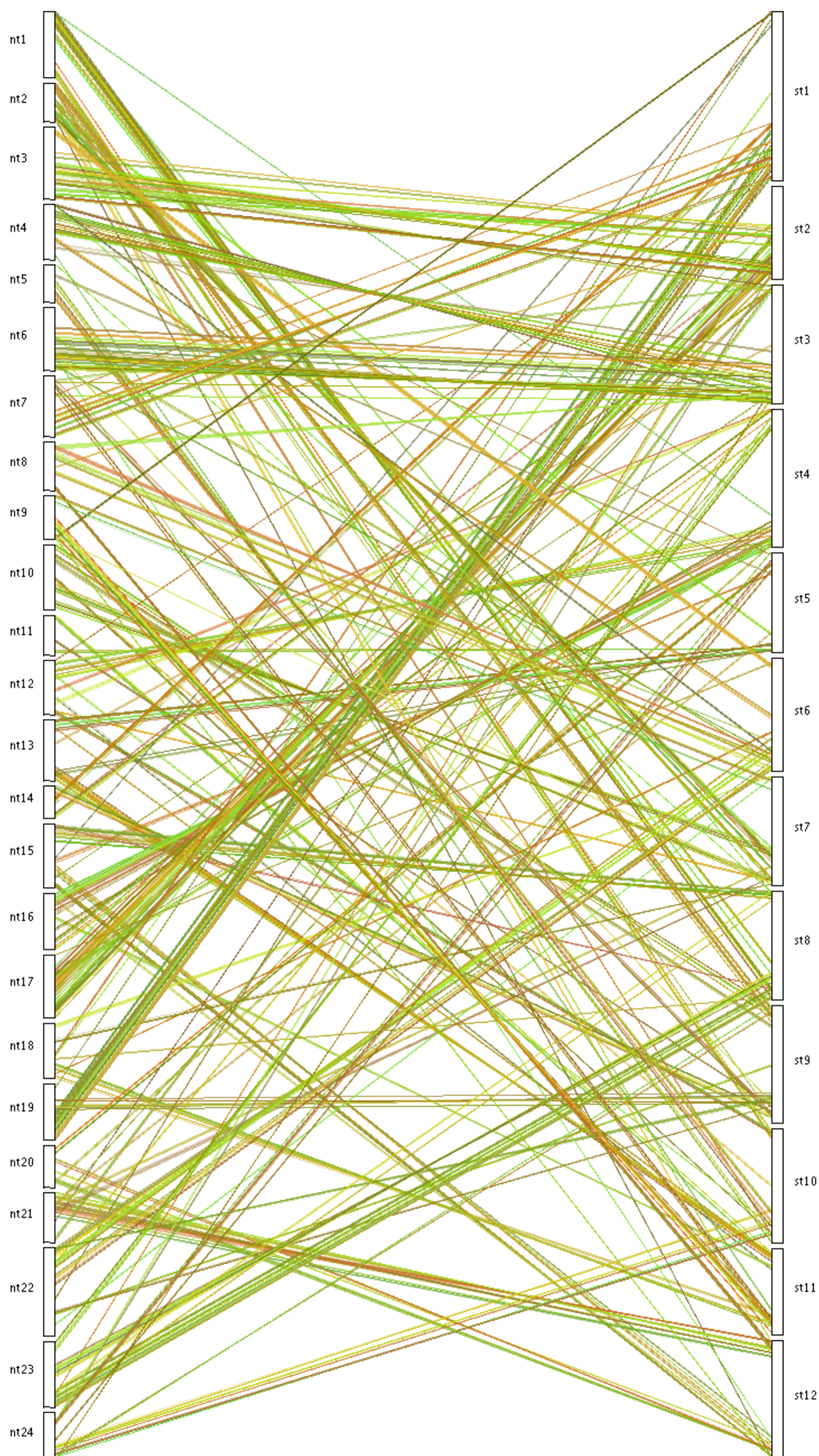

Supplement: Supplementary Data 9 — Synteny of the 24 Nicotiana tabacum linkage groups with the 12 potato chromosomes determined using MCScanX. [file ncomms4833-s10.pdf]
