# Supplementary material for: The tobacco genome sequence and its comparison with those of tomato and potato
Source: Nat Commun. 2014 May 8;5:3833. doi: 10.1038/ncomms4833 (PMC4024737; doi:10.1038/ncomms4833)
Supplement: Supplementary Data 4 — Synteny of the 24 Nicotiana tabacum linkage groups with the 12 tomato chromosomes, based on whole genome sequences. Syntenic DNA blocks in each plot are positioned on the x axis according to their location in the Nicotiana tabacum linkage group, and on the y axis according to their location on the tomato chromosome. [file ncomms4833-s5.pdf]

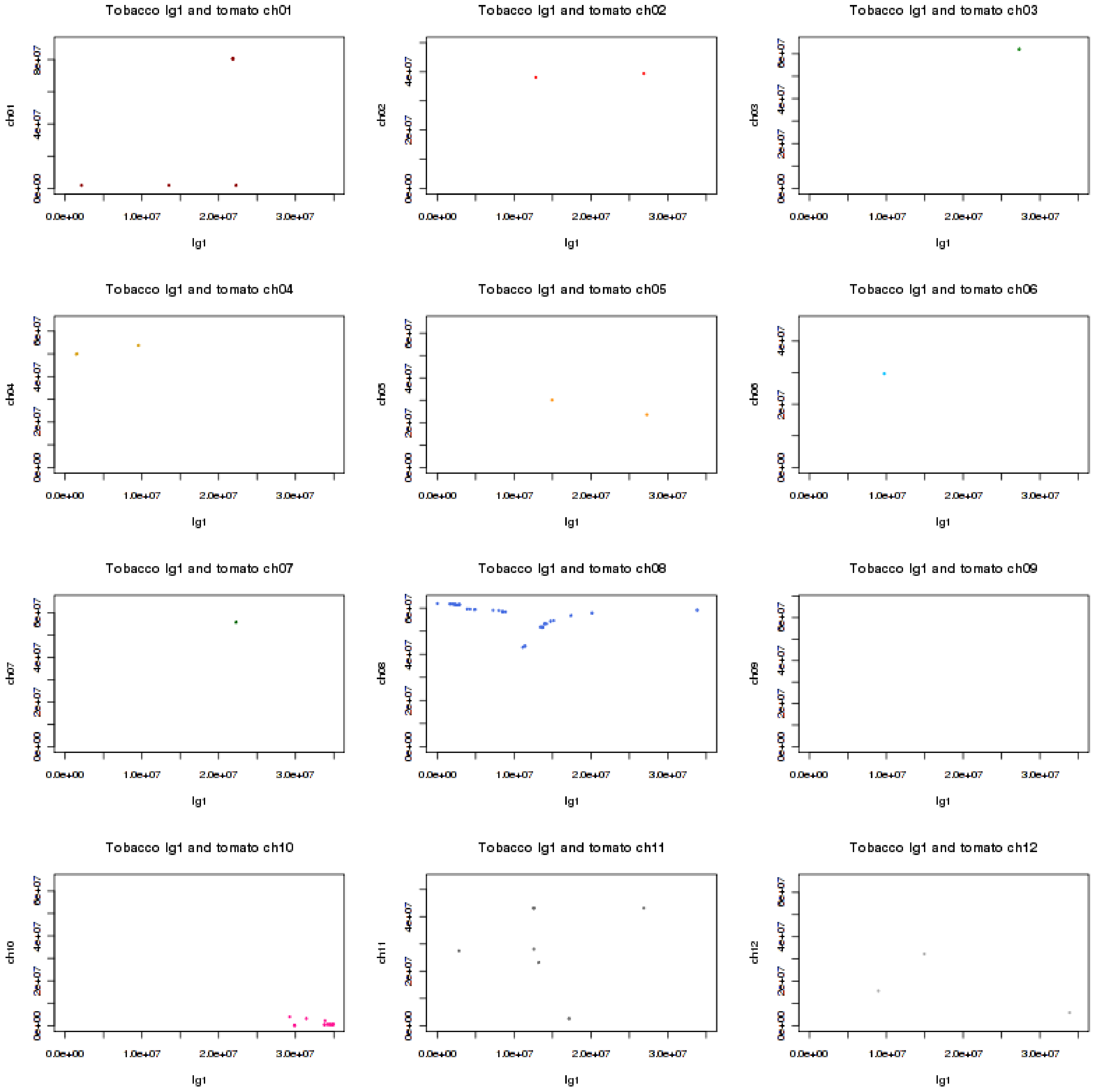
Tobacco lg1 and tomato ch01
Tobacco lg1 and tomato ch02
Tobacco lg1 and tomato ch03
Tobacco lg1 and tomato ch04
Tobacco lg1 and tomato ch05
Tobacco lg1 and tomato ch06
Tobacco lg1 and tomato ch07
Tobacco lg1 and tomato ch08
Tobacco lg1 and tomato ch09
Tobacco lg1 and tomato ch10
Tobacco lg1 and tomato ch11
Tobacco lg1 and tomato ch12
lg1
ch01
ch02
ch03
ch04
ch05
ch06
ch07
ch08
ch09
ch10
ch11
ch12
0.0e+00
1.0e+07
2.0e+07
3.0e+07
0e+00
2e+07
4e+07
6e+07
8e+07

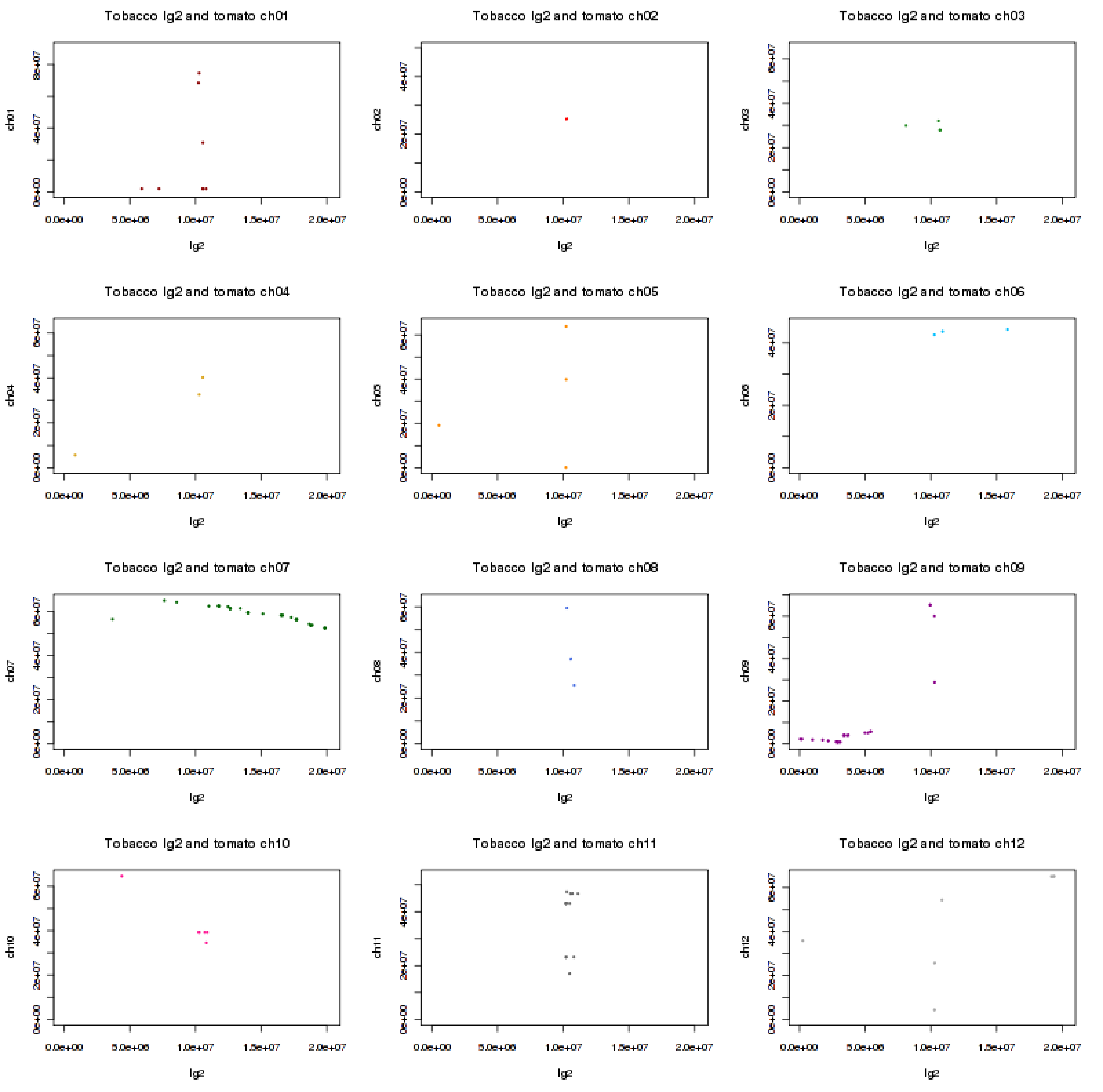

Tobacco lg2 and tomato ch01
ch01
lg2
Tobacco lg2 and tomato ch02
ch02
lg2
Tobacco lg2 and tomato ch03
ch03
lg2
Tobacco lg2 and tomato ch04
ch04
lg2
Tobacco lg2 and tomato ch05
ch05
lg2
Tobacco lg2 and tomato ch06
ch06
lg2
Tobacco lg2 and tomato ch07
ch07
lg2
Tobacco lg2 and tomato ch08
ch08
lg2
Tobacco lg2 and tomato ch09
ch09
lg2
Tobacco lg2 and tomato ch10
ch10
lg2
Tobacco lg2 and tomato ch11
ch11
lg2
Tobacco lg2 and tomato ch12
ch12
lg2
0.0e+00
5.0e+06
1.0e+07
1.5e+07
2.0e+07
0e+00
2e+07
4e+07
6e+07
8e+07

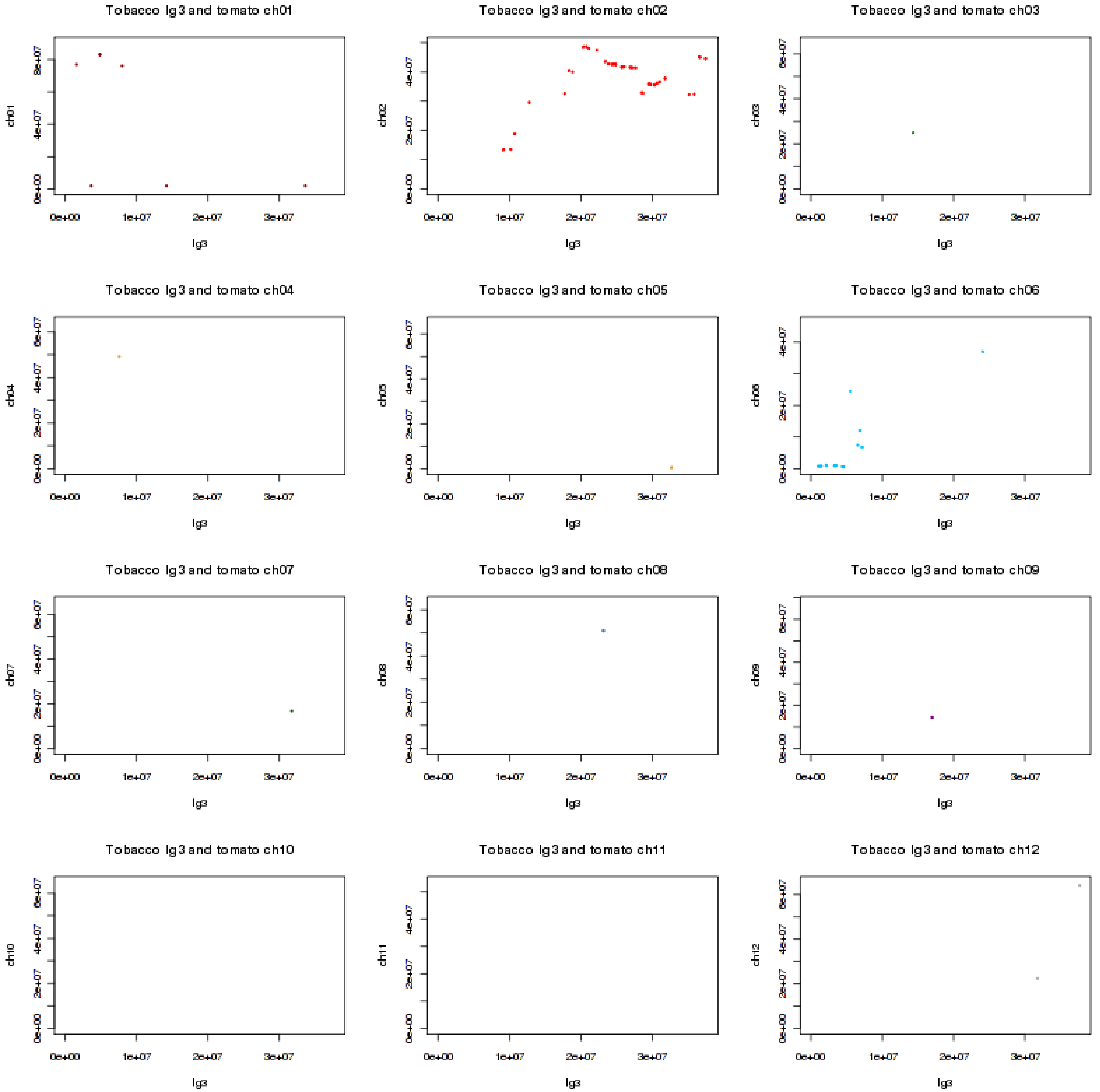
Tobacco lg3 and tomato ch01
ch01
lg3
Tobacco lg3 and tomato ch02
ch02
lg3
Tobacco lg3 and tomato ch03
ch03
lg3
Tobacco lg3 and tomato ch04
ch04
lg3
Tobacco lg3 and tomato ch05
ch05
lg3
Tobacco lg3 and tomato ch06
ch06
lg3
Tobacco lg3 and tomato ch07
ch07
lg3
Tobacco lg3 and tomato ch08
ch08
lg3
Tobacco lg3 and tomato ch09
ch09
lg3
Tobacco lg3 and tomato ch10
ch10
lg3
Tobacco lg3 and tomato ch11
ch11
lg3
Tobacco lg3 and tomato ch12
ch12
lg3
0e+00
1e+07
2e+07
3e+07
4e+07
6e+07
8e+07

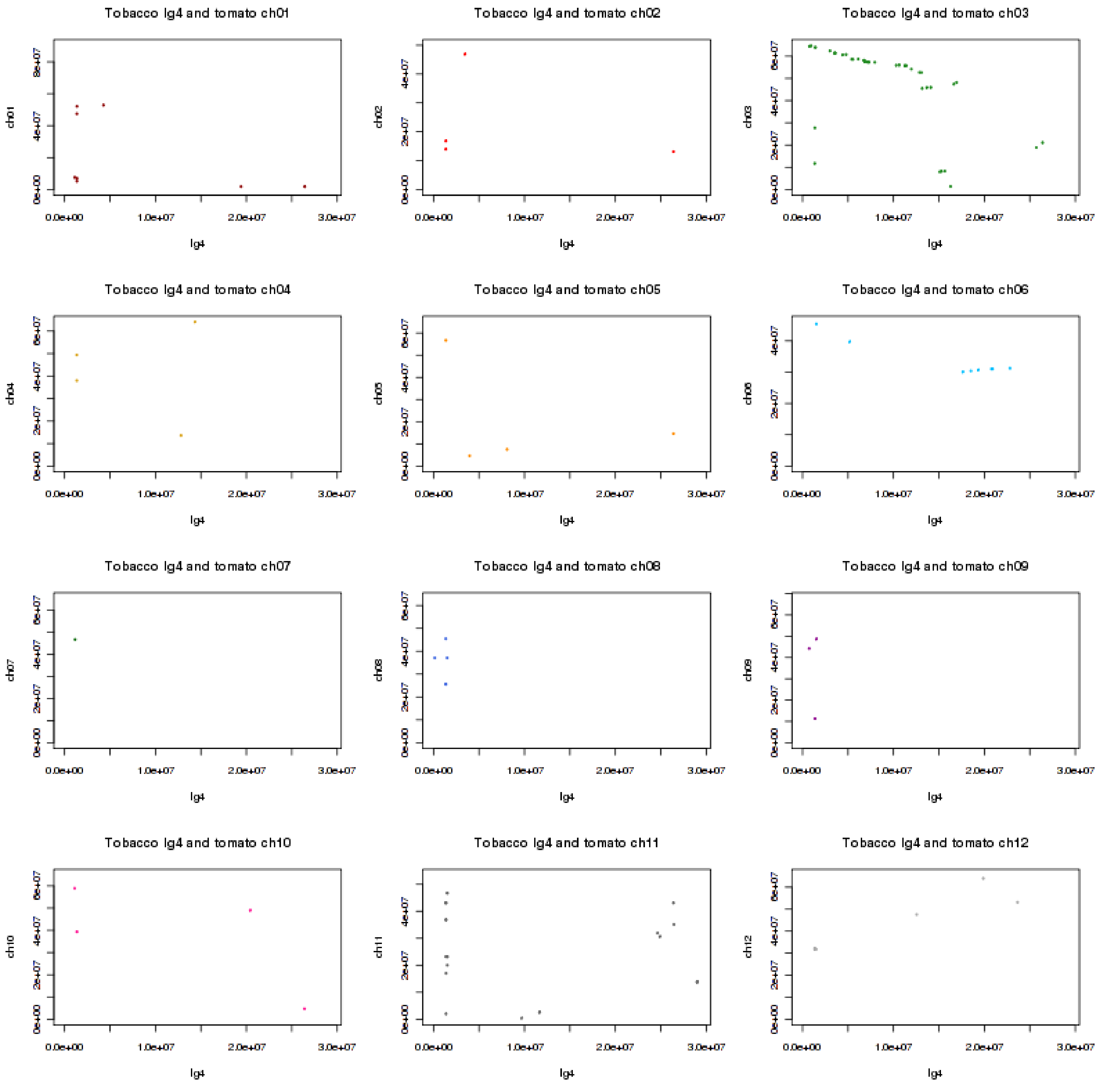
Tobacco lg4 and tomato ch01
ch01
lg4
Tobacco lg4 and tomato ch02
ch02
lg4
Tobacco lg4 and tomato ch03
ch03
lg4
Tobacco lg4 and tomato ch04
ch04
lg4
Tobacco lg4 and tomato ch05
ch05
lg4
Tobacco lg4 and tomato ch06
ch06
lg4
Tobacco lg4 and tomato ch07
ch07
lg4
Tobacco lg4 and tomato ch08
ch08
lg4
Tobacco lg4 and tomato ch09
ch09
lg4
Tobacco lg4 and tomato ch10
ch10
lg4
Tobacco lg4 and tomato ch11
ch11
lg4
Tobacco lg4 and tomato ch12
ch12
lg4
0.0e+00
1.0e+07
2.0e+07
3.0e+07
0e+00
2e+07
4e+07
6e+07
8e+07

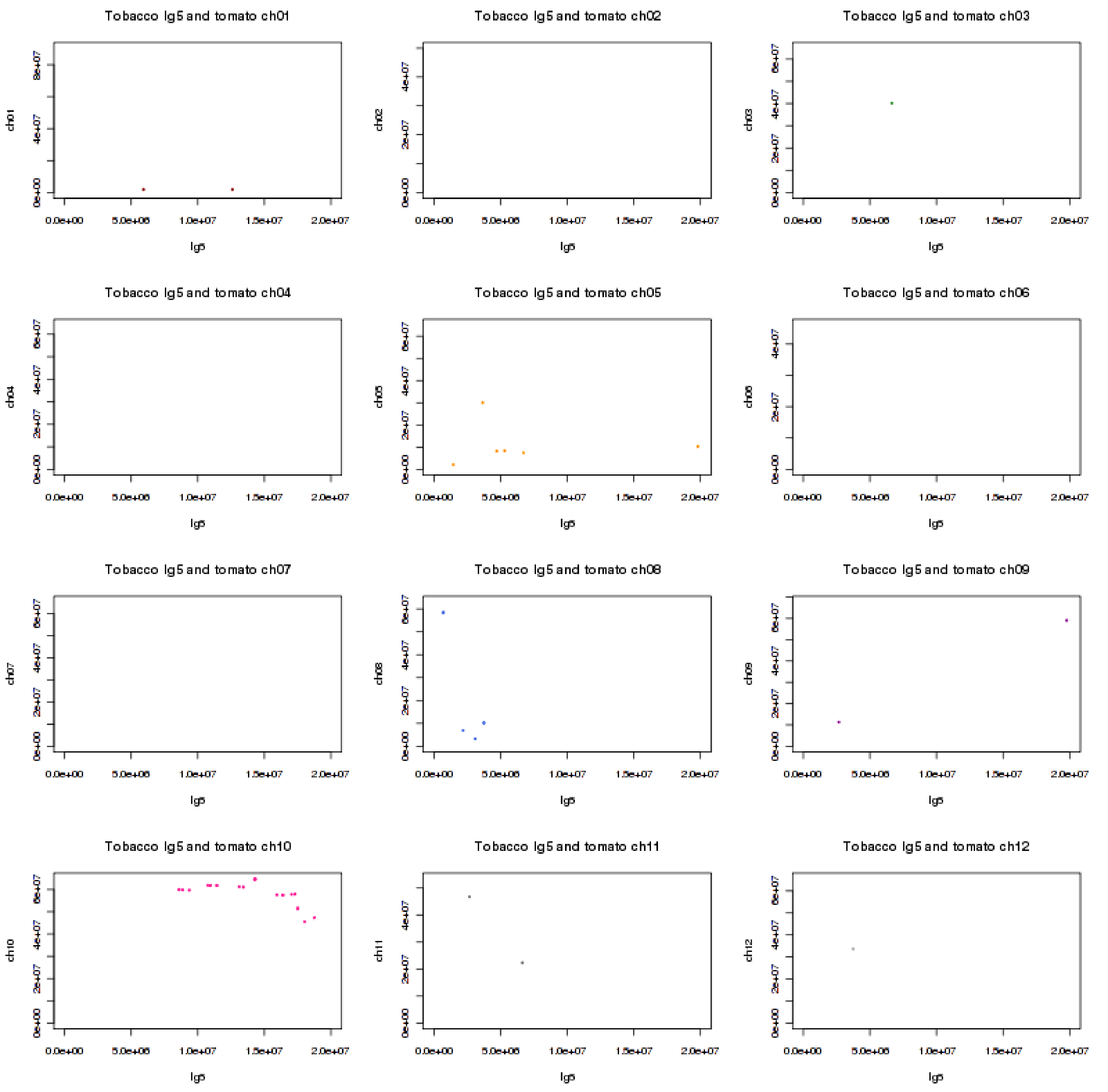
Tobacco lg5 and tomato ch01
Tobacco lg5 and tomato ch02
Tobacco lg5 and tomato ch03
Tobacco lg5 and tomato ch04
Tobacco lg5 and tomato ch05
Tobacco lg5 and tomato ch06
Tobacco lg5 and tomato ch07
Tobacco lg5 and tomato ch08
Tobacco lg5 and tomato ch09
Tobacco lg5 and tomato ch10
Tobacco lg5 and tomato ch11
Tobacco lg5 and tomato ch12
lg5
ch01
ch02
ch03
ch04
ch05
ch06
ch07
ch08
ch09
ch10
ch11
ch12
0.0e+00
5.0e+06
1.0e+07
1.5e+07
2.0e+07
0e+00
2e+07
4e+07
6e+07
8e+07

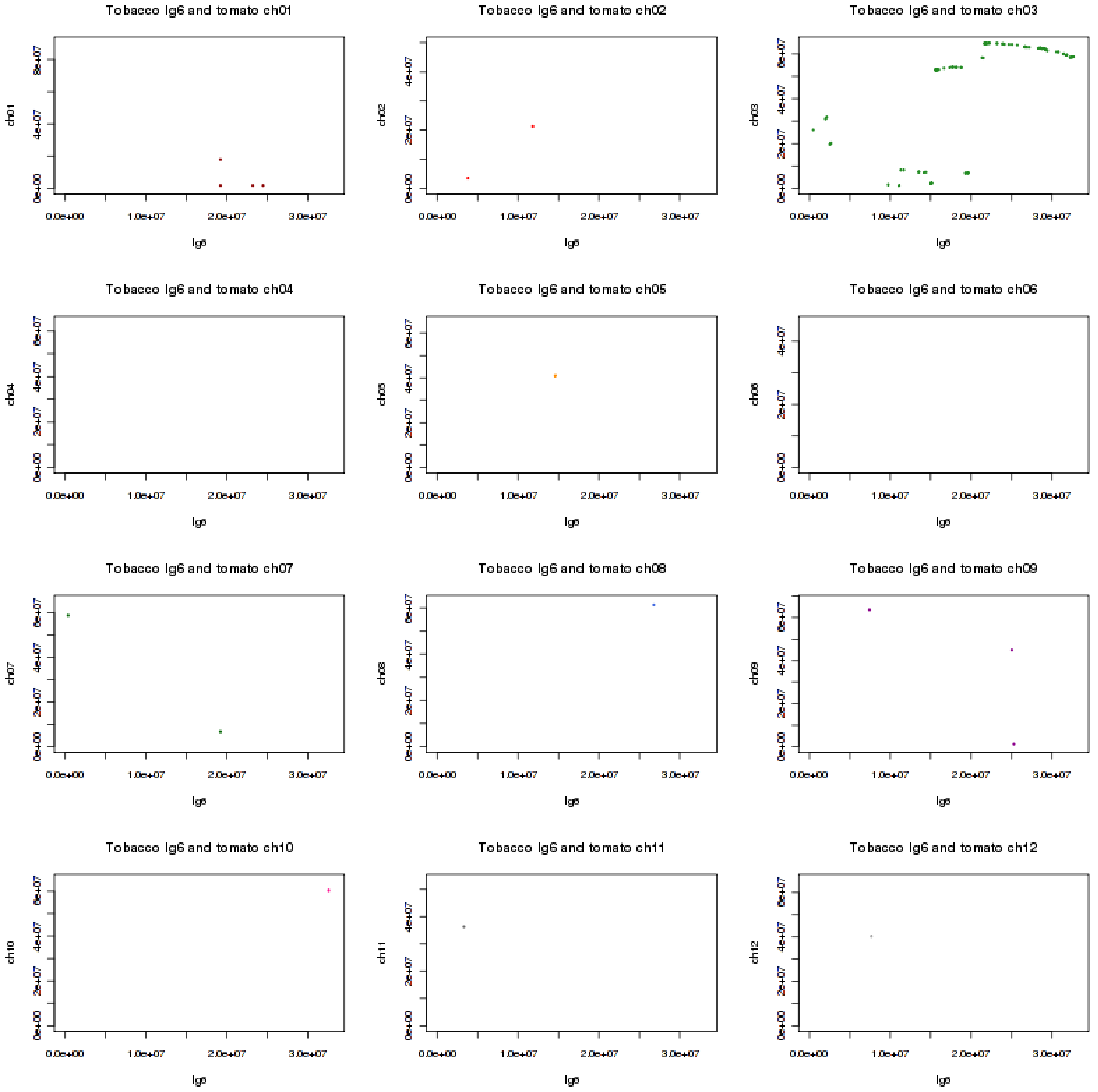

Tobacco lg6 and tomato ch01
ch01
0e+00
4e+07
8e+07
0.0e+00
1.0e+07
2.0e+07
3.0e+07
lg6
Tobacco lg6 and tomato ch02
ch02
0e+00
2e+07
4e+07
lg6
Tobacco lg6 and tomato ch03
ch03
0e+00
2e+07
4e+07
6e+07
lg6
Tobacco lg6 and tomato ch04
ch04
0e+00
2e+07
4e+07
6e+07
lg6
Tobacco lg6 and tomato ch05
ch05
0e+00
2e+07
4e+07
6e+07
lg6
Tobacco lg6 and tomato ch06
ch06
0e+00
2e+07
4e+07
lg6
Tobacco lg6 and tomato ch07
ch07
0e+00
2e+07
4e+07
6e+07
lg6
Tobacco lg6 and tomato ch08
ch08
0e+00
2e+07
4e+07
6e+07
lg6
Tobacco lg6 and tomato ch09
ch09
0e+00
2e+07
4e+07
6e+07
lg6
Tobacco lg6 and tomato ch10
ch10
0e+00
2e+07
4e+07
6e+07
lg6
Tobacco lg6 and tomato ch11
ch11
0e+00
2e+07
4e+07
lg6
Tobacco lg6 and tomato ch12
ch12
0e+00
2e+07
4e+07
6e+07
lg6

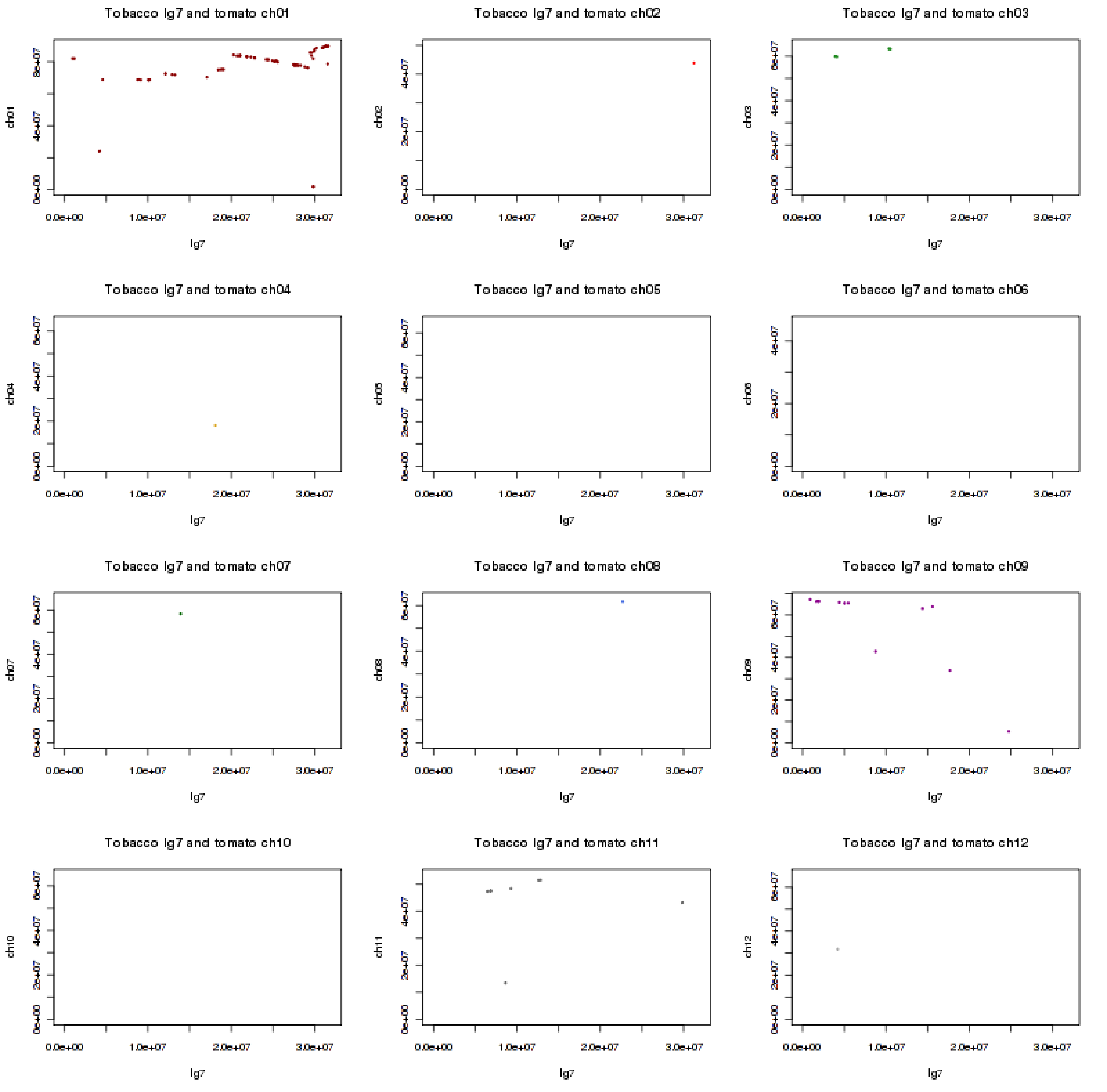

Tobacco lg7 and tomato ch01
ch01
lg7
Tobacco lg7 and tomato ch02
ch02
lg7
Tobacco lg7 and tomato ch03
ch03
lg7
Tobacco lg7 and tomato ch04
ch04
lg7
Tobacco lg7 and tomato ch05
ch05
lg7
Tobacco lg7 and tomato ch06
ch06
lg7
Tobacco lg7 and tomato ch07
ch07
lg7
Tobacco lg7 and tomato ch08
ch08
lg7
Tobacco lg7 and tomato ch09
ch09
lg7
Tobacco lg7 and tomato ch10
ch10
lg7
Tobacco lg7 and tomato ch11
ch11
lg7
Tobacco lg7 and tomato ch12
ch12
lg7
0.0e+00
1.0e+07
2.0e+07
3.0e+07
0e+00
2e+07
4e+07
6e+07
8e+07

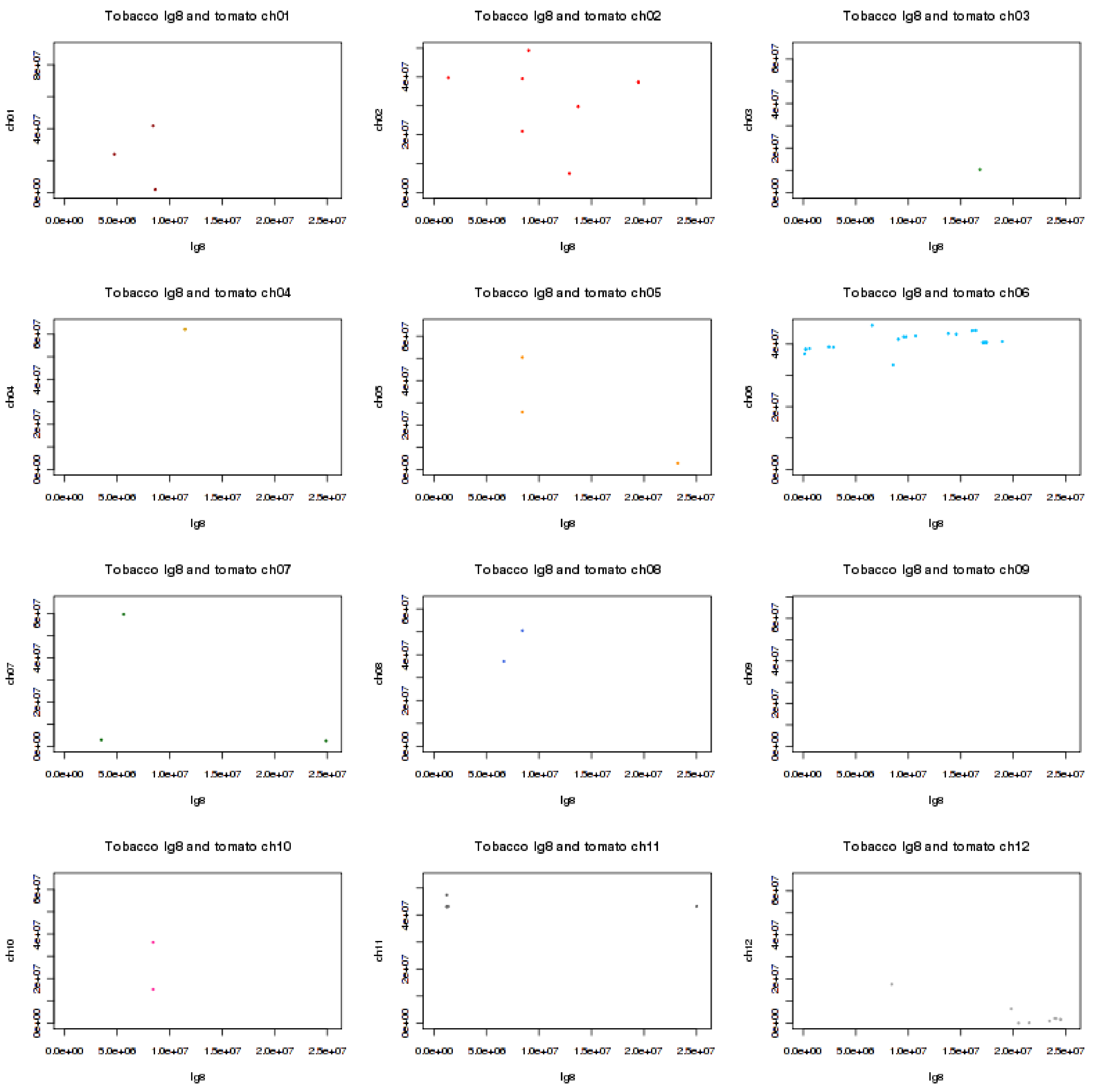
Tobacco lg8 and tomato ch01
Tobacco lg8 and tomato ch02
Tobacco lg8 and tomato ch03
Tobacco lg8 and tomato ch04
Tobacco lg8 and tomato ch05
Tobacco lg8 and tomato ch06
Tobacco lg8 and tomato ch07
Tobacco lg8 and tomato ch08
Tobacco lg8 and tomato ch09
Tobacco lg8 and tomato ch10
Tobacco lg8 and tomato ch11
Tobacco lg8 and tomato ch12
lg8
ch01
ch02
ch03
ch04
ch05
ch06
ch07
ch08
ch09
ch10
ch11
ch12
0.0e+00
5.0e+06
1.0e+07
1.5e+07
2.0e+07
2.5e+07
0e+00
2e+07
4e+07
6e+07
8e+07

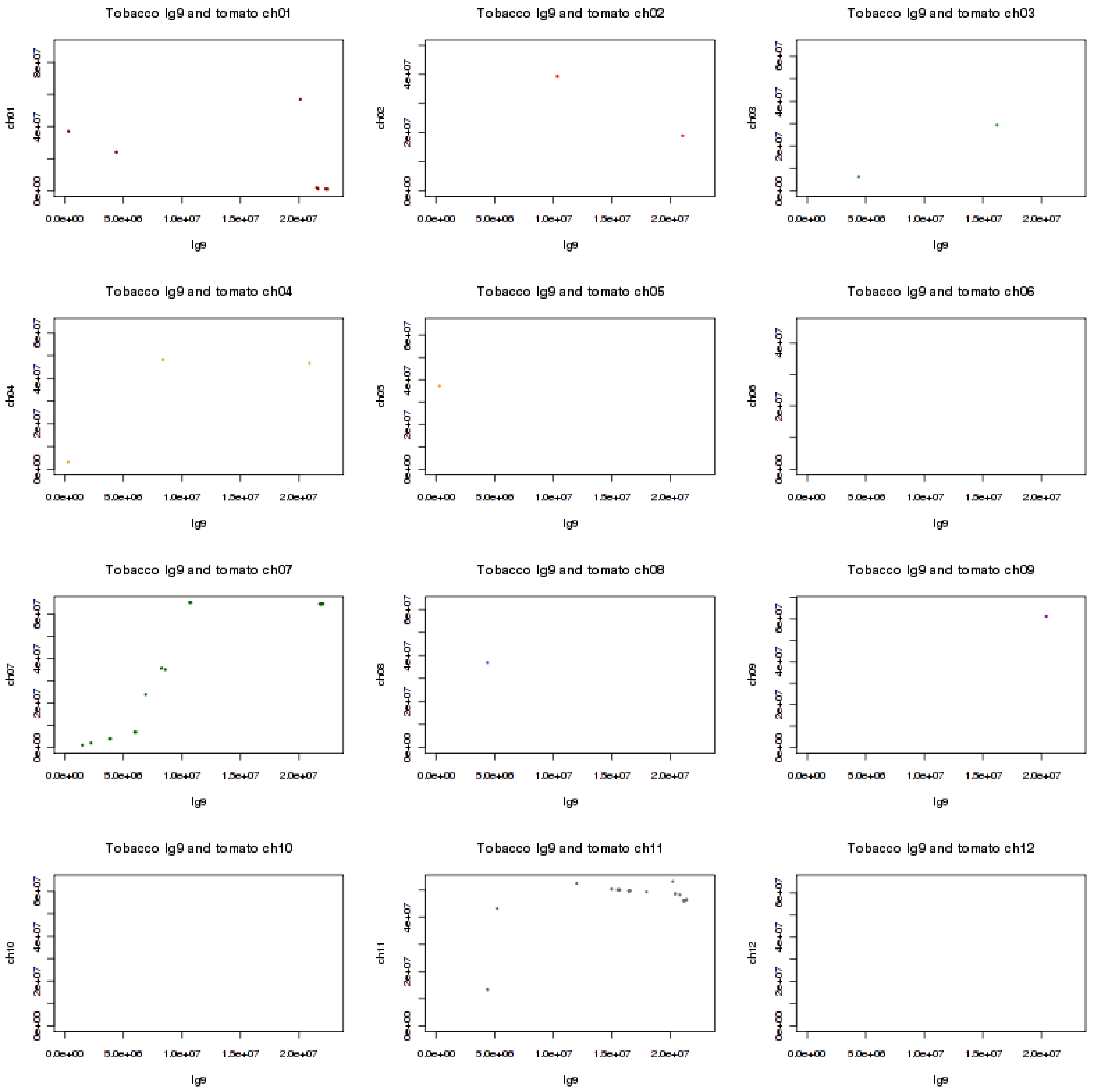
Tobacco lg9 and tomato ch01
Tobacco lg9 and tomato ch02
Tobacco lg9 and tomato ch03
Tobacco lg9 and tomato ch04
Tobacco lg9 and tomato ch05
Tobacco lg9 and tomato ch06
Tobacco lg9 and tomato ch07
Tobacco lg9 and tomato ch08
Tobacco lg9 and tomato ch09
Tobacco lg9 and tomato ch10
Tobacco lg9 and tomato ch11
Tobacco lg9 and tomato ch12
lg9
0.0e+00
5.0e+06
1.0e+07
1.5e+07
2.0e+07
ch01
ch02
ch03
ch04
ch05
ch06
ch07
ch08
ch09
ch10
ch11
ch12
0e+00
2e+07
4e+07
6e+07

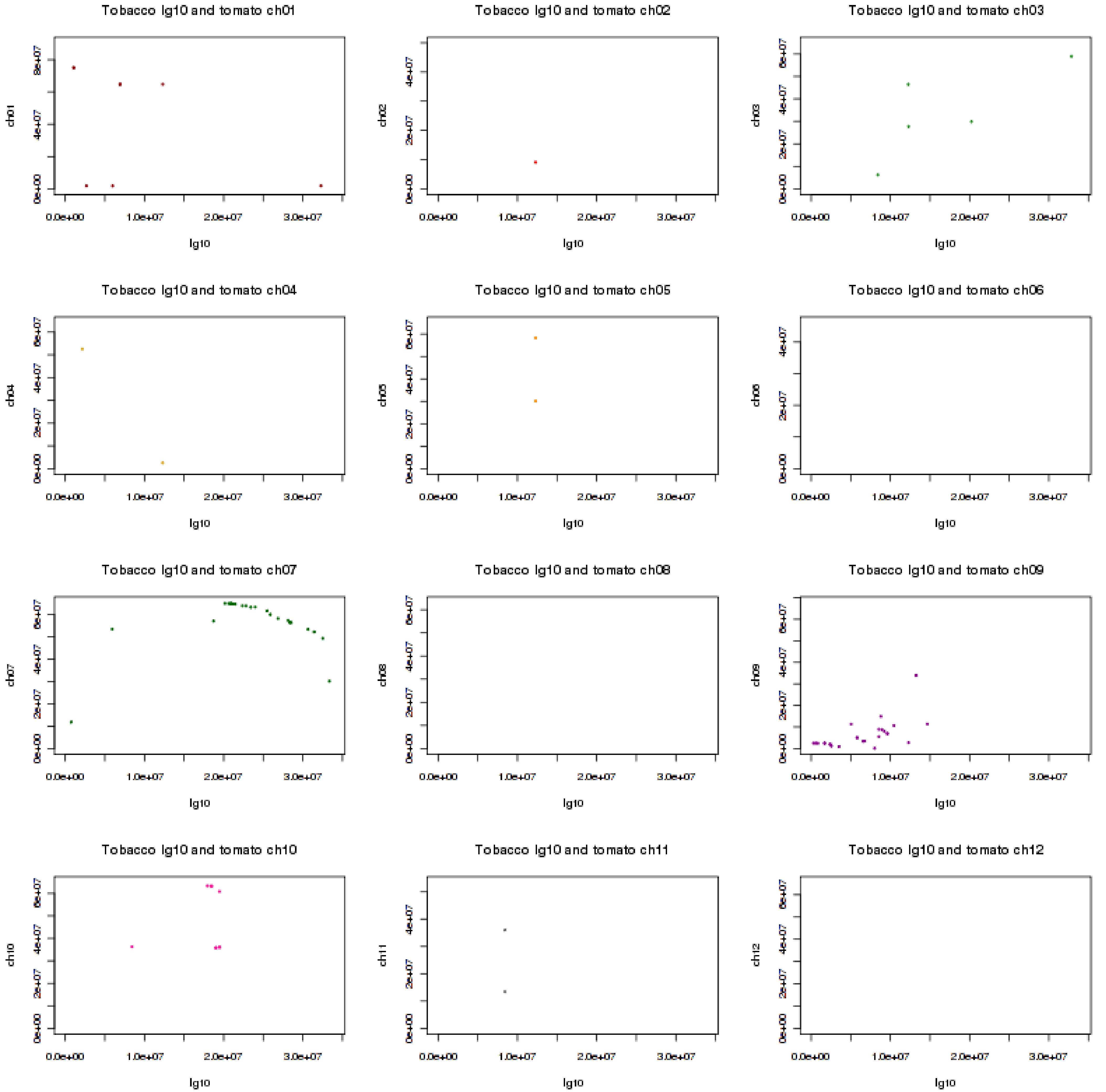
Tobacco lg10 and tomato ch01
Tobacco lg10 and tomato ch02
Tobacco lg10 and tomato ch03
Tobacco lg10 and tomato ch04
Tobacco lg10 and tomato ch05
Tobacco lg10 and tomato ch06
Tobacco lg10 and tomato ch07
Tobacco lg10 and tomato ch08
Tobacco lg10 and tomato ch09
Tobacco lg10 and tomato ch10
Tobacco lg10 and tomato ch11
Tobacco lg10 and tomato ch12
ch01
ch02
ch03
ch04
ch05
ch06
ch07
ch08
ch09
ch10
ch11
ch12
lg10
0.0e+00
1.0e+07
2.0e+07
3.0e+07
0e+00
2e+07
4e+07
6e+07
8e+07

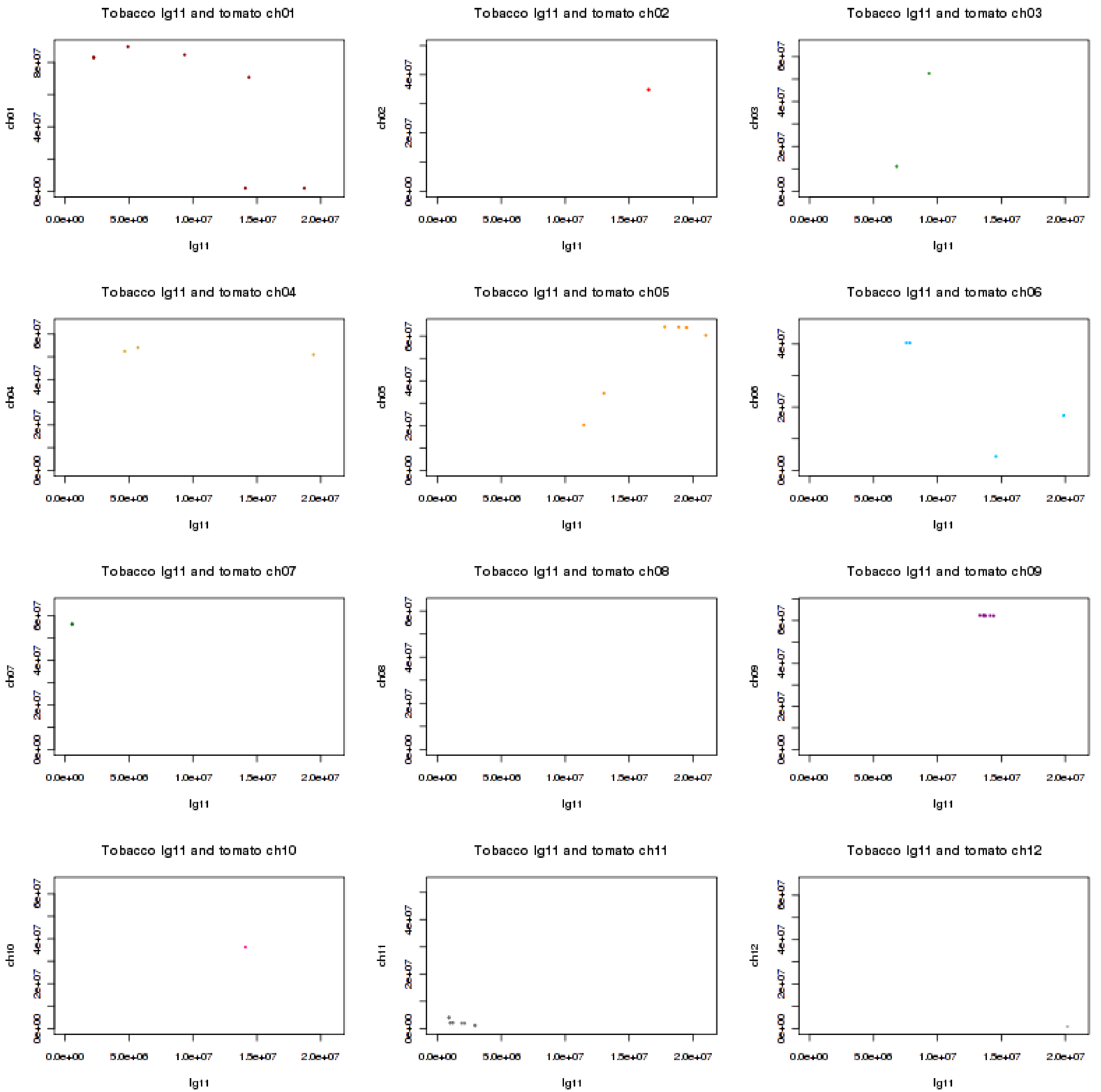
Tobacco lg11 and tomato ch01
Tobacco lg11 and tomato ch02
Tobacco lg11 and tomato ch03
Tobacco lg11 and tomato ch04
Tobacco lg11 and tomato ch05
Tobacco lg11 and tomato ch06
Tobacco lg11 and tomato ch07
Tobacco lg11 and tomato ch08
Tobacco lg11 and tomato ch09
Tobacco lg11 and tomato ch10
Tobacco lg11 and tomato ch11
Tobacco lg11 and tomato ch12
lg11
ch01
ch02
ch03
ch04
ch05
ch06
ch07
ch08
ch09
ch10
ch11
ch12
0.0e+00
5.0e+06
1.0e+07
1.5e+07
2.0e+07
0e+00
2e+07
4e+07
6e+07
8e+07

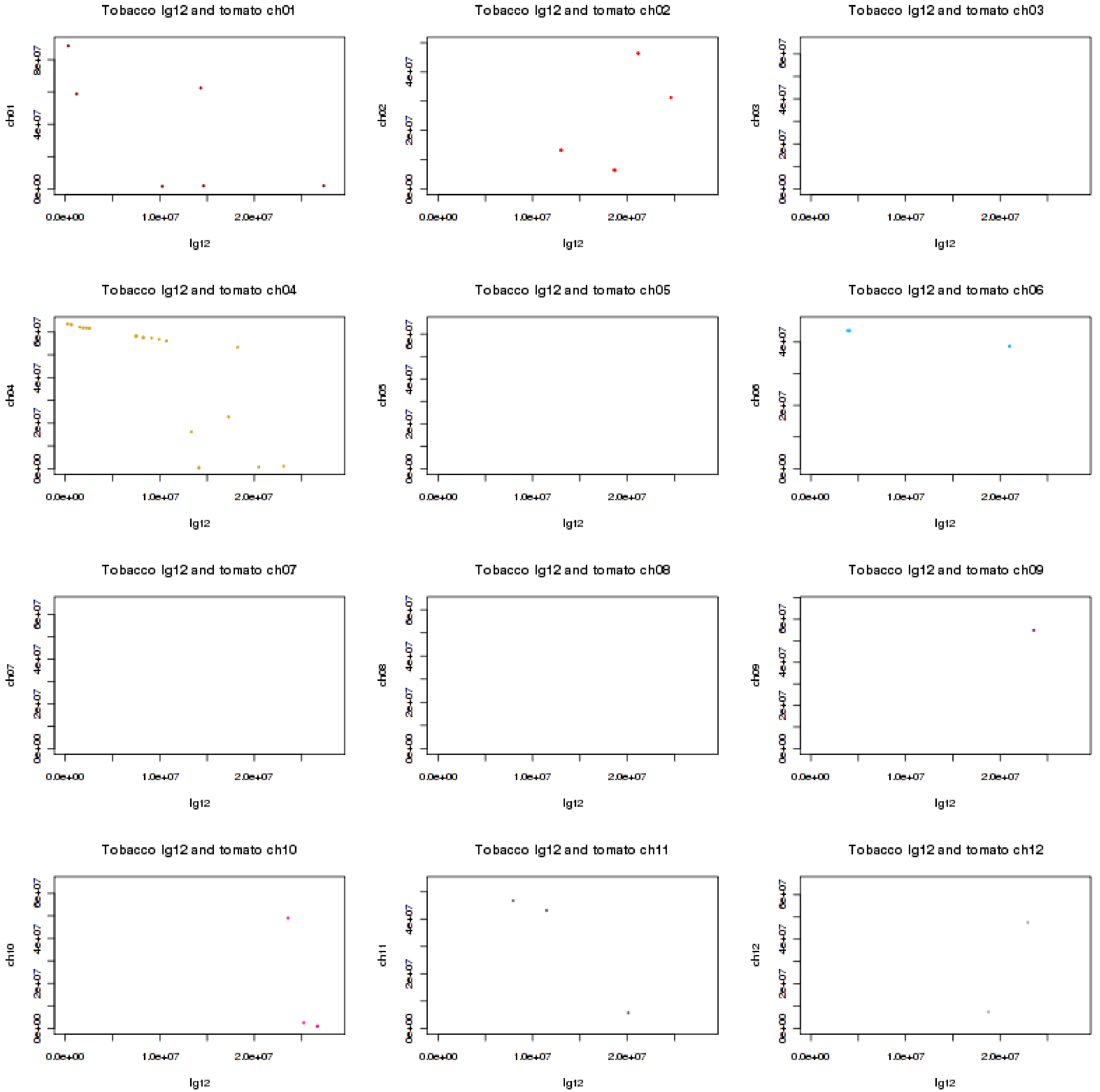
Tobacco lg12 and tomato ch01
ch01
lg12
Tobacco lg12 and tomato ch02
ch02
lg12
Tobacco lg12 and tomato ch03
ch03
lg12
Tobacco lg12 and tomato ch04
ch04
lg12
Tobacco lg12 and tomato ch05
ch05
lg12
Tobacco lg12 and tomato ch06
ch06
lg12
Tobacco lg12 and tomato ch07
ch07
lg12
Tobacco lg12 and tomato ch08
ch08
lg12
Tobacco lg12 and tomato ch09
ch09
lg12
Tobacco lg12 and tomato ch10
ch10
lg12
Tobacco lg12 and tomato ch11
ch11
lg12
Tobacco lg12 and tomato ch12
ch12
lg12
0.0e+00
1.0e+07
2.0e+07
0e+00
2e+07
4e+07
6e+07
8e+07

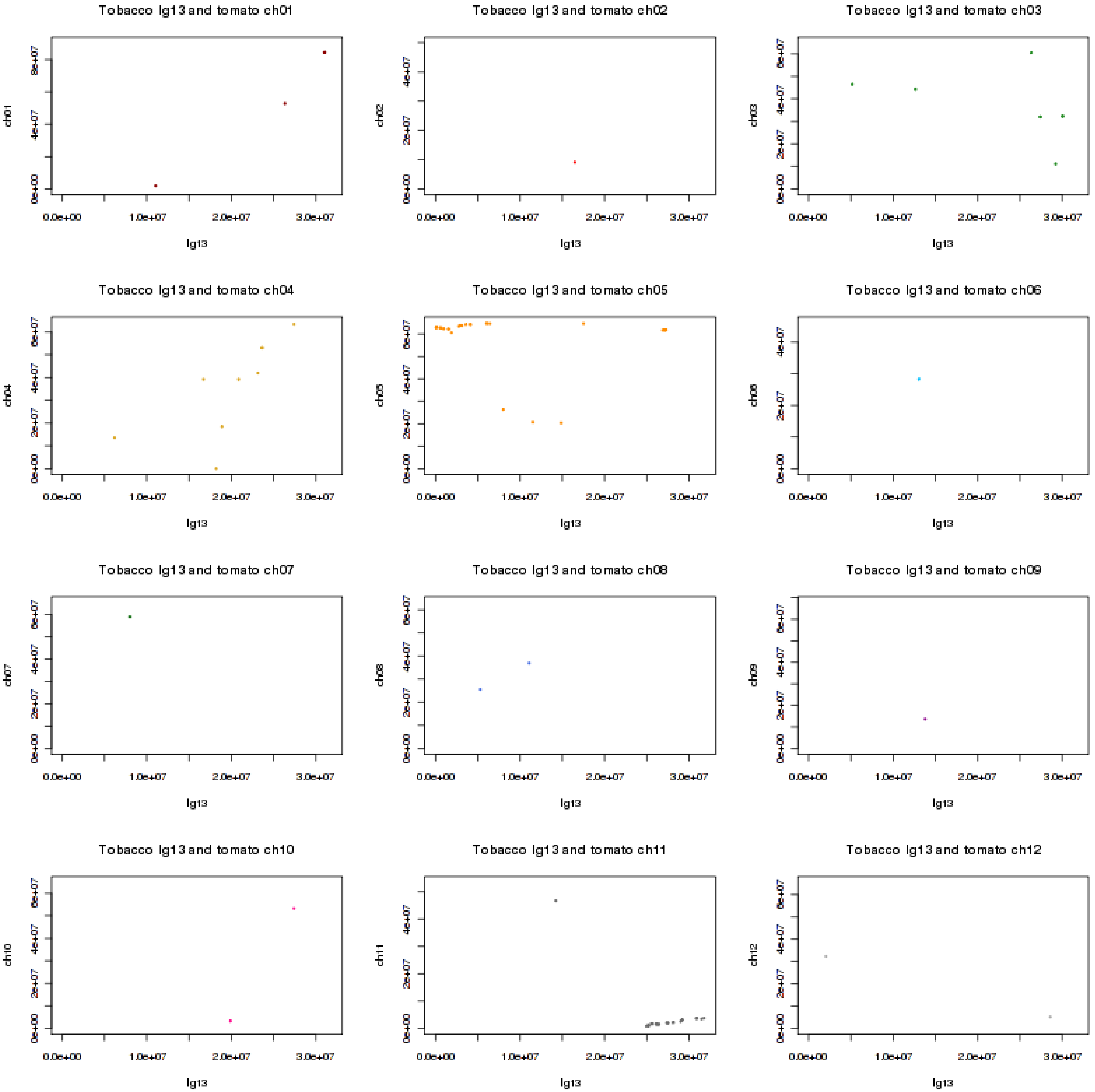
Tobacco lg13 and tomato ch01
Tobacco lg13 and tomato ch02
Tobacco lg13 and tomato ch03
Tobacco lg13 and tomato ch04
Tobacco lg13 and tomato ch05
Tobacco lg13 and tomato ch06
Tobacco lg13 and tomato ch07
Tobacco lg13 and tomato ch08
Tobacco lg13 and tomato ch09
Tobacco lg13 and tomato ch10
Tobacco lg13 and tomato ch11
Tobacco lg13 and tomato ch12
lg13
ch01
ch02
ch03
ch04
ch05
ch06
ch07
ch08
ch09
ch10
ch11
ch12
0.0e+00
1.0e+07
2.0e+07
3.0e+07
0e+00
2e+07
4e+07
6e+07
8e+07

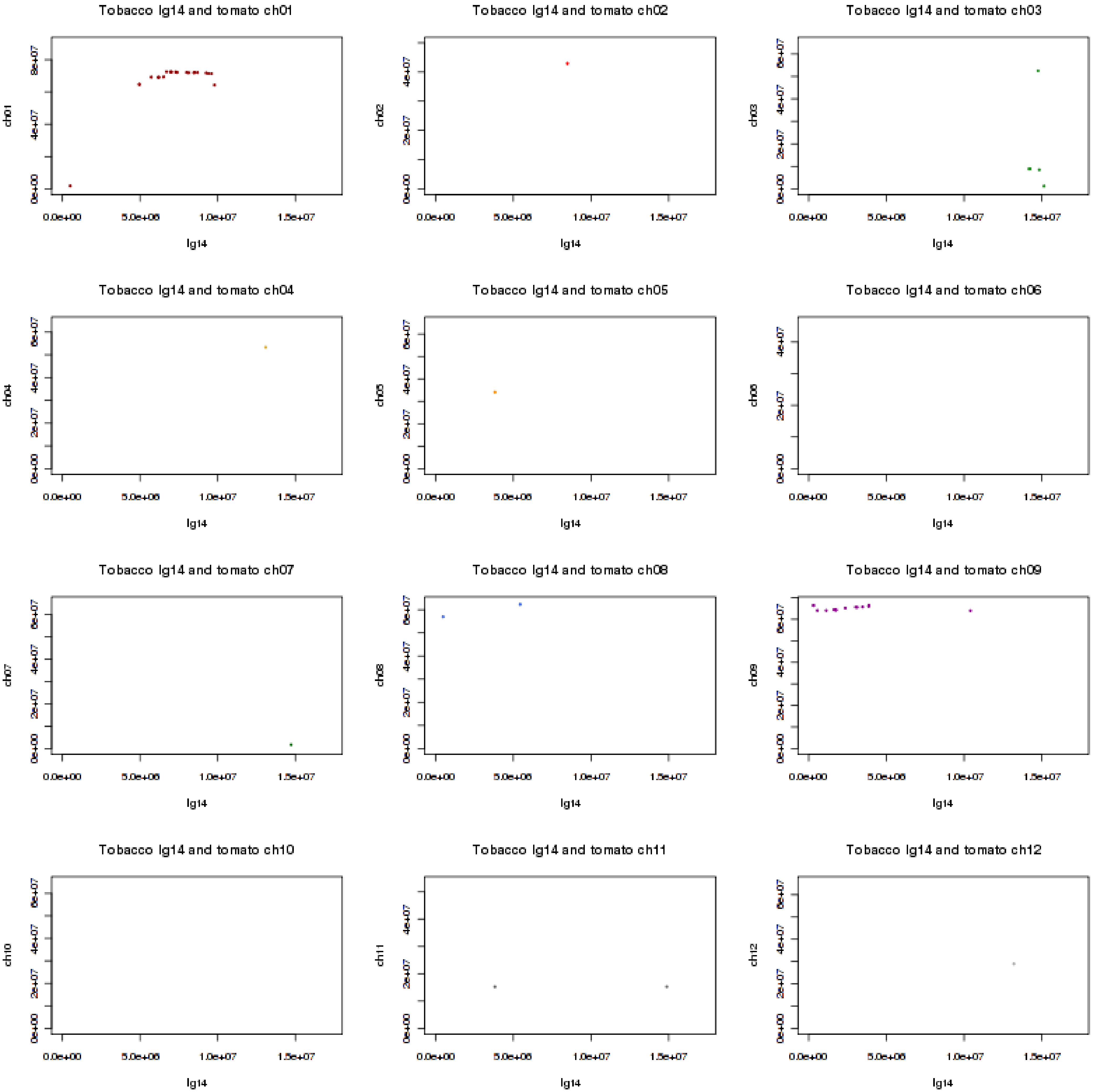

Tobacco lg14 and tomato ch01
ch01
0e+00
4e+07
8e+07
lg14
0.0e+00
5.0e+06
1.0e+07
1.5e+07
Tobacco lg14 and tomato ch02
ch02
0e+00
2e+07
4e+07
lg14
Tobacco lg14 and tomato ch03
ch03
0e+00
2e+07
4e+07
6e+07
lg14
Tobacco lg14 and tomato ch04
ch04
0e+00
2e+07
4e+07
6e+07
lg14
Tobacco lg14 and tomato ch05
ch05
0e+00
2e+07
4e+07
6e+07
lg14
Tobacco lg14 and tomato ch06
ch06
0e+00
2e+07
4e+07
lg14
Tobacco lg14 and tomato ch07
ch07
0e+00
2e+07
4e+07
6e+07
lg14
Tobacco lg14 and tomato ch08
ch08
0e+00
2e+07
4e+07
6e+07
lg14
Tobacco lg14 and tomato ch09
ch09
0e+00
2e+07
4e+07
6e+07
lg14
Tobacco lg14 and tomato ch10
ch10
0e+00
2e+07
4e+07
6e+07
lg14
Tobacco lg14 and tomato ch11
ch11
0e+00
2e+07
4e+07
lg14
Tobacco lg14 and tomato ch12
ch12
0e+00
2e+07
4e+07
6e+07
lg14

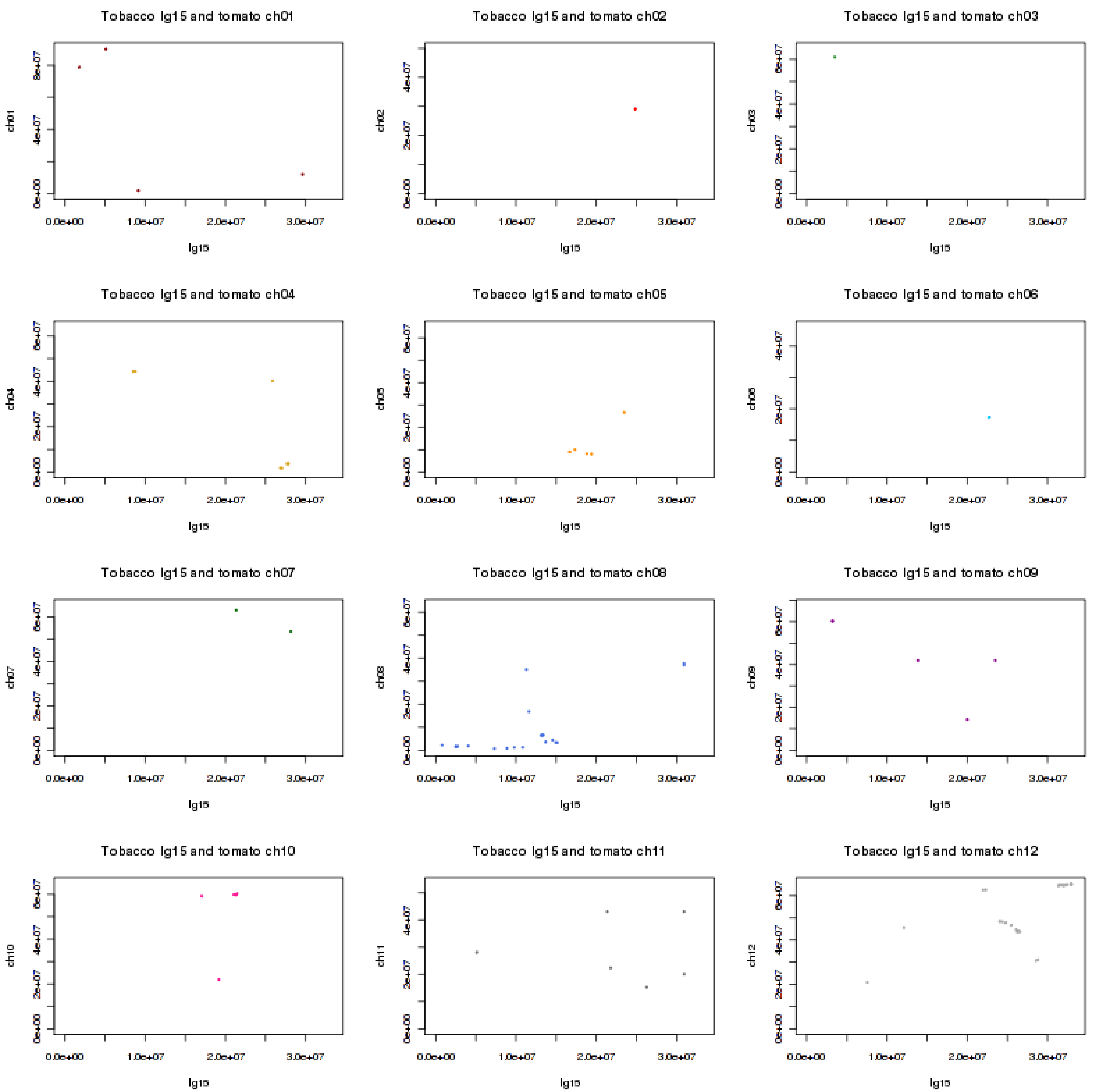
Tobacco lg15 and tomato ch01
ch01
0e+00
4e+07
8e+07
0.0e+00
1.0e+07
2.0e+07
3.0e+07
lg15
Tobacco lg15 and tomato ch02
ch02
0e+00
2e+07
4e+07
lg15
Tobacco lg15 and tomato ch03
ch03
0e+00
2e+07
4e+07
6e+07
lg15
Tobacco lg15 and tomato ch04
ch04
lg15
Tobacco lg15 and tomato ch05
ch05
lg15
Tobacco lg15 and tomato ch06
ch06
lg15
Tobacco lg15 and tomato ch07
ch07
lg15
Tobacco lg15 and tomato ch08
ch08
lg15
Tobacco lg15 and tomato ch09
ch09
lg15
Tobacco lg15 and tomato ch10
ch10
lg15
Tobacco lg15 and tomato ch11
ch11
lg15
Tobacco lg15 and tomato ch12
ch12
lg15

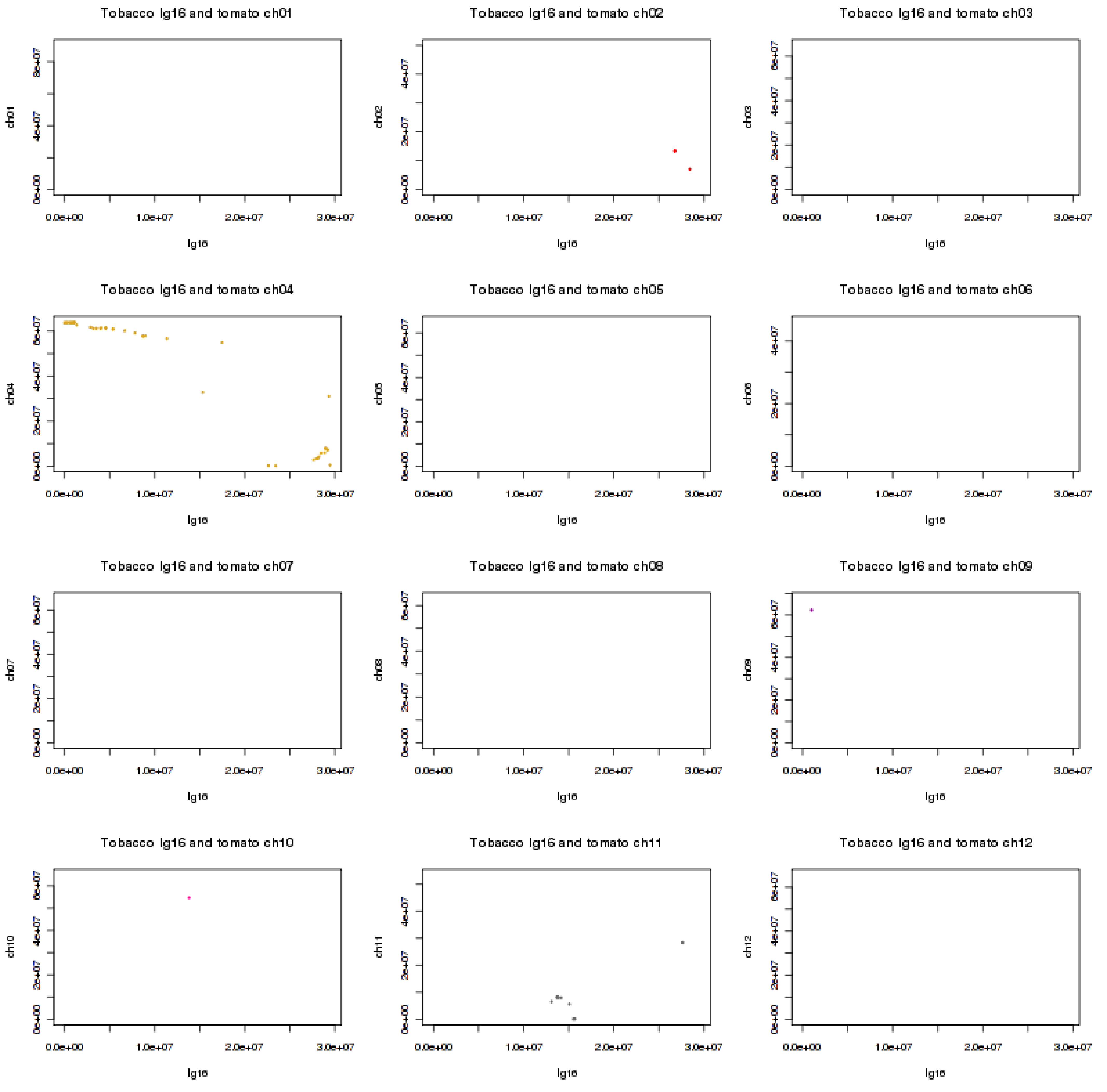

Tobacco lg16 and tomato ch01
ch01
lg16
Tobacco lg16 and tomato ch02
ch02
lg16
Tobacco lg16 and tomato ch03
ch03
lg16
Tobacco lg16 and tomato ch04
ch04
lg16
Tobacco lg16 and tomato ch05
ch05
lg16
Tobacco lg16 and tomato ch06
ch06
lg16
Tobacco lg16 and tomato ch07
ch07
lg16
Tobacco lg16 and tomato ch08
ch08
lg16
Tobacco lg16 and tomato ch09
ch09
lg16
Tobacco lg16 and tomato ch10
ch10
lg16
Tobacco lg16 and tomato ch11
ch11
lg16
Tobacco lg16 and tomato ch12
ch12
lg16
0.0e+00
1.0e+07
2.0e+07
3.0e+07
0e+00
2e+07
4e+07
6e+07
8e+07

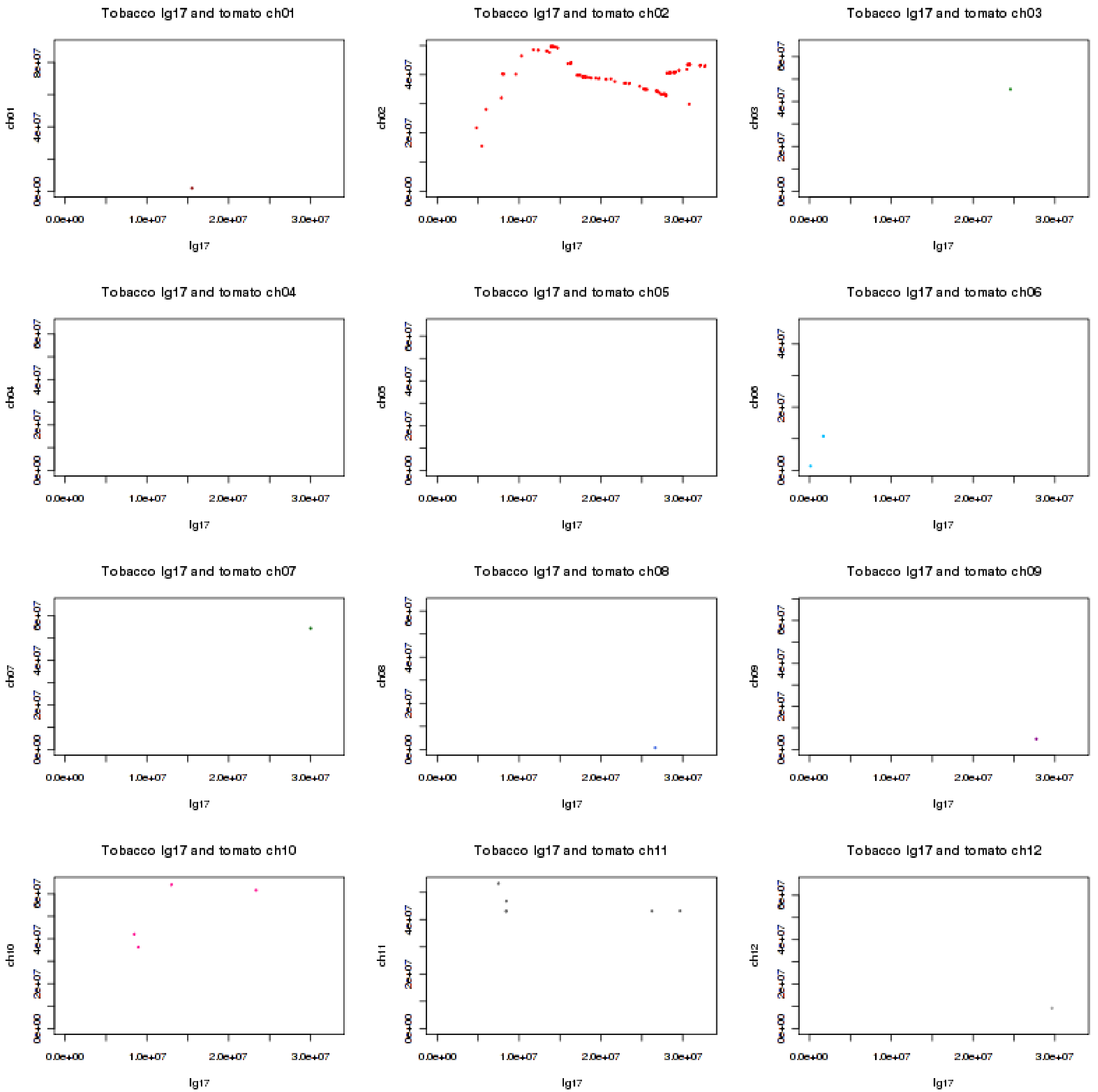
Tobacco lg17 and tomato ch01
Tobacco lg17 and tomato ch02
Tobacco lg17 and tomato ch03
Tobacco lg17 and tomato ch04
Tobacco lg17 and tomato ch05
Tobacco lg17 and tomato ch06
Tobacco lg17 and tomato ch07
Tobacco lg17 and tomato ch08
Tobacco lg17 and tomato ch09
Tobacco lg17 and tomato ch10
Tobacco lg17 and tomato ch11
Tobacco lg17 and tomato ch12
lg17
0.0e+00
1.0e+07
2.0e+07
3.0e+07
ch01
ch02
ch03
ch04
ch05
ch06
ch07
ch08
ch09
ch10
ch11
ch12
0e+00
2e+07
4e+07
6e+07
8e+07

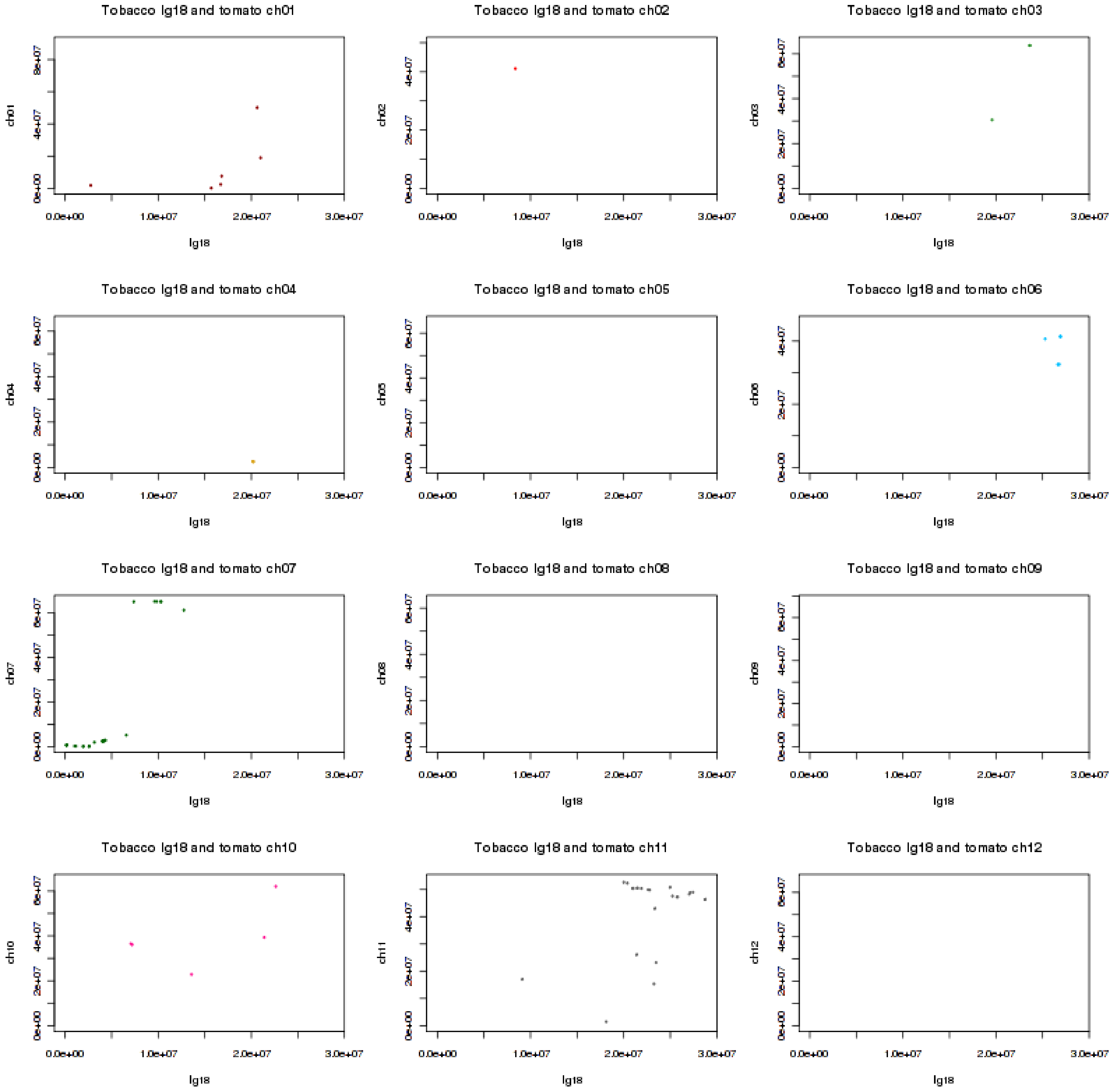
Tobacco lg18 and tomato ch01
Tobacco lg18 and tomato ch02
Tobacco lg18 and tomato ch03
Tobacco lg18 and tomato ch04
Tobacco lg18 and tomato ch05
Tobacco lg18 and tomato ch06
Tobacco lg18 and tomato ch07
Tobacco lg18 and tomato ch08
Tobacco lg18 and tomato ch09
Tobacco lg18 and tomato ch10
Tobacco lg18 and tomato ch11
Tobacco lg18 and tomato ch12
lg18
ch01
ch02
ch03
ch04
ch05
ch06
ch07
ch08
ch09
ch10
ch11
ch12
0.0e+00
1.0e+07
2.0e+07
3.0e+07
0e+00
2e+07
4e+07
6e+07
8e+07

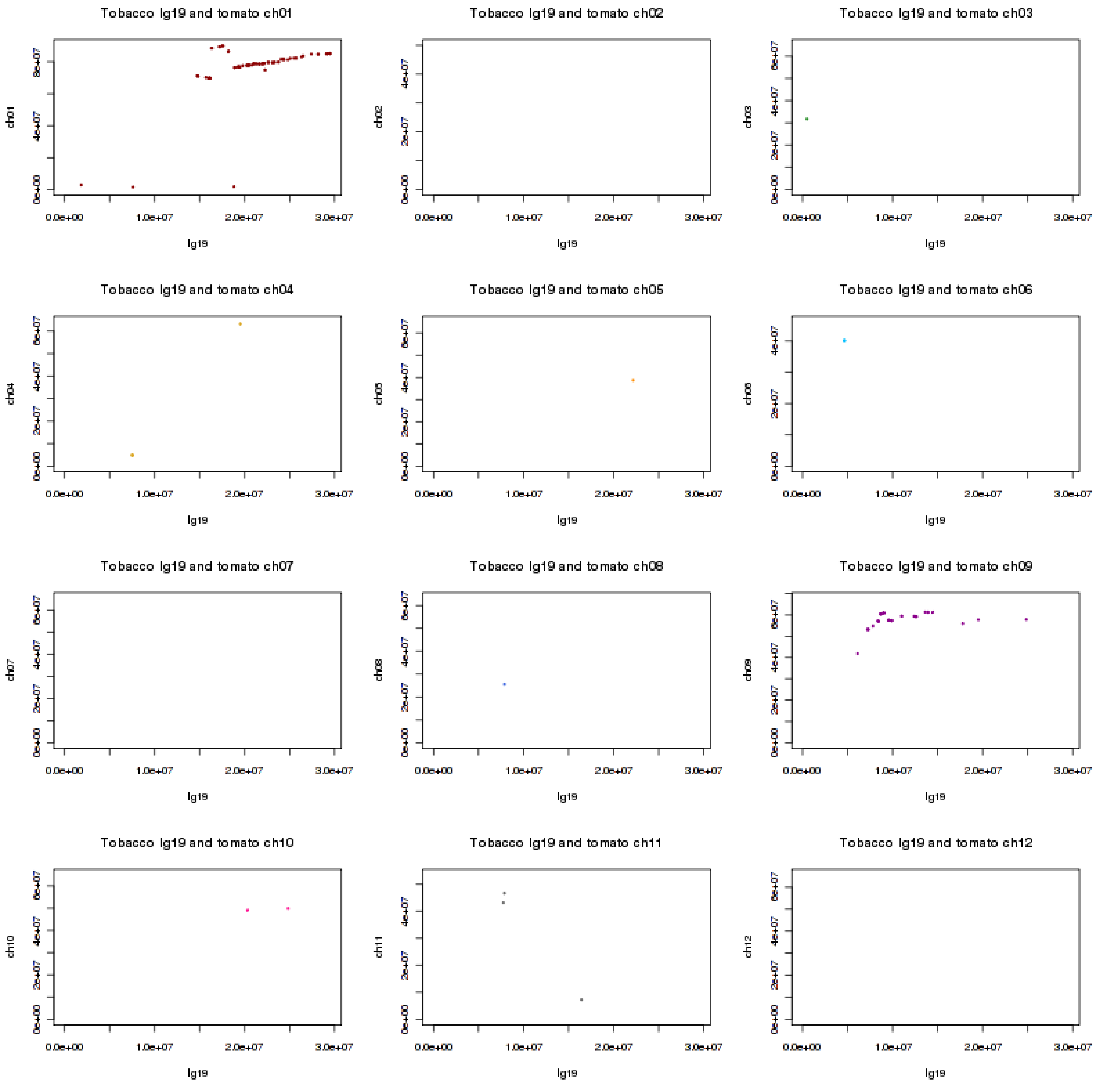
Tobacco lg19 and tomato ch01
ch01
lg19
Tobacco lg19 and tomato ch02
ch02
lg19
Tobacco lg19 and tomato ch03
ch03
lg19
Tobacco lg19 and tomato ch04
ch04
lg19
Tobacco lg19 and tomato ch05
ch05
lg19
Tobacco lg19 and tomato ch06
ch06
lg19
Tobacco lg19 and tomato ch07
ch07
lg19
Tobacco lg19 and tomato ch08
ch08
lg19
Tobacco lg19 and tomato ch09
ch09
lg19
Tobacco lg19 and tomato ch10
ch10
lg19
Tobacco lg19 and tomato ch11
ch11
lg19
Tobacco lg19 and tomato ch12
ch12
lg19

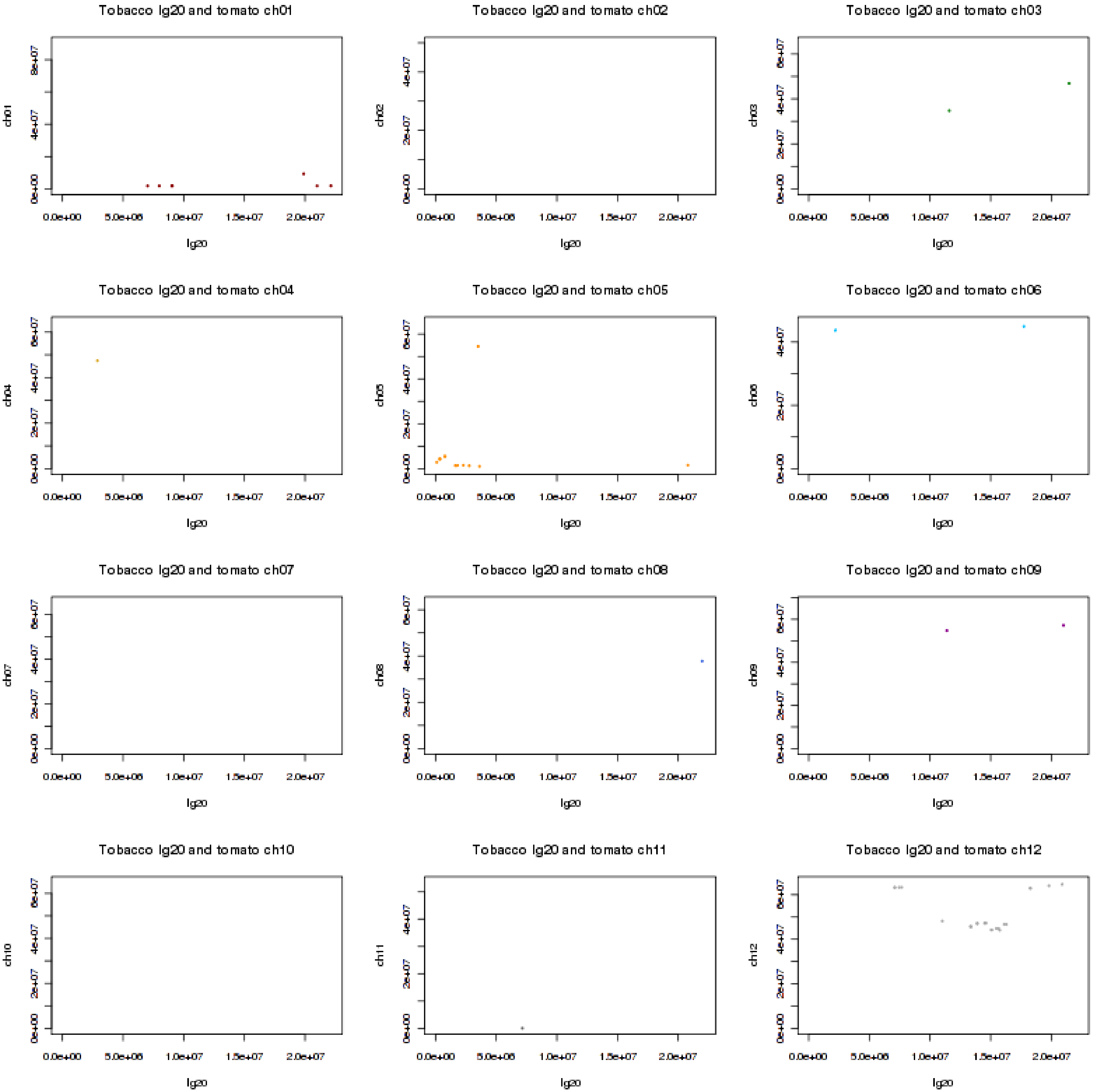
Tobacco lg20 and tomato ch01
ch01
0e+00 4e+07 8e+07
0.0e+00 5.0e+06 1.0e+07 1.5e+07 2.0e+07
lg20
Tobacco lg20 and tomato ch02
ch02
0e+00 2e+07 4e+07
0.0e+00 5.0e+06 1.0e+07 1.5e+07 2.0e+07
lg20
Tobacco lg20 and tomato ch03
ch03
0e+00 2e+07 4e+07 6e+07
0.0e+00 5.0e+06 1.0e+07 1.5e+07 2.0e+07
lg20
Tobacco lg20 and tomato ch04
ch04
0e+00 2e+07 4e+07 6e+07
0.0e+00 5.0e+06 1.0e+07 1.5e+07 2.0e+07
lg20
Tobacco lg20 and tomato ch05
ch05
0e+00 2e+07 4e+07 6e+07
0.0e+00 5.0e+06 1.0e+07 1.5e+07 2.0e+07
lg20
Tobacco lg20 and tomato ch06
ch06
0e+00 2e+07 4e+07
0.0e+00 5.0e+06 1.0e+07 1.5e+07 2.0e+07
lg20
Tobacco lg20 and tomato ch07
ch07
0e+00 2e+07 4e+07 6e+07
0.0e+00 5.0e+06 1.0e+07 1.5e+07 2.0e+07
lg20
Tobacco lg20 and tomato ch08
ch08
0e+00 2e+07 4e+07 6e+07
0.0e+00 5.0e+06 1.0e+07 1.5e+07 2.0e+07
lg20
Tobacco lg20 and tomato ch09
ch09
0e+00 2e+07 4e+07 6e+07
0.0e+00 5.0e+06 1.0e+07 1.5e+07 2.0e+07
lg20
Tobacco lg20 and tomato ch10
ch10
0e+00 2e+07 4e+07 6e+07
0.0e+00 5.0e+06 1.0e+07 1.5e+07 2.0e+07
lg20
Tobacco lg20 and tomato ch11
ch11
0e+00 2e+07 4e+07
0.0e+00 5.0e+06 1.0e+07 1.5e+07 2.0e+07
lg20
Tobacco lg20 and tomato ch12
ch12
0e+00 2e+07 4e+07 6e+07
0.0e+00 5.0e+06 1.0e+07 1.5e+07 2.0e+07
lg20

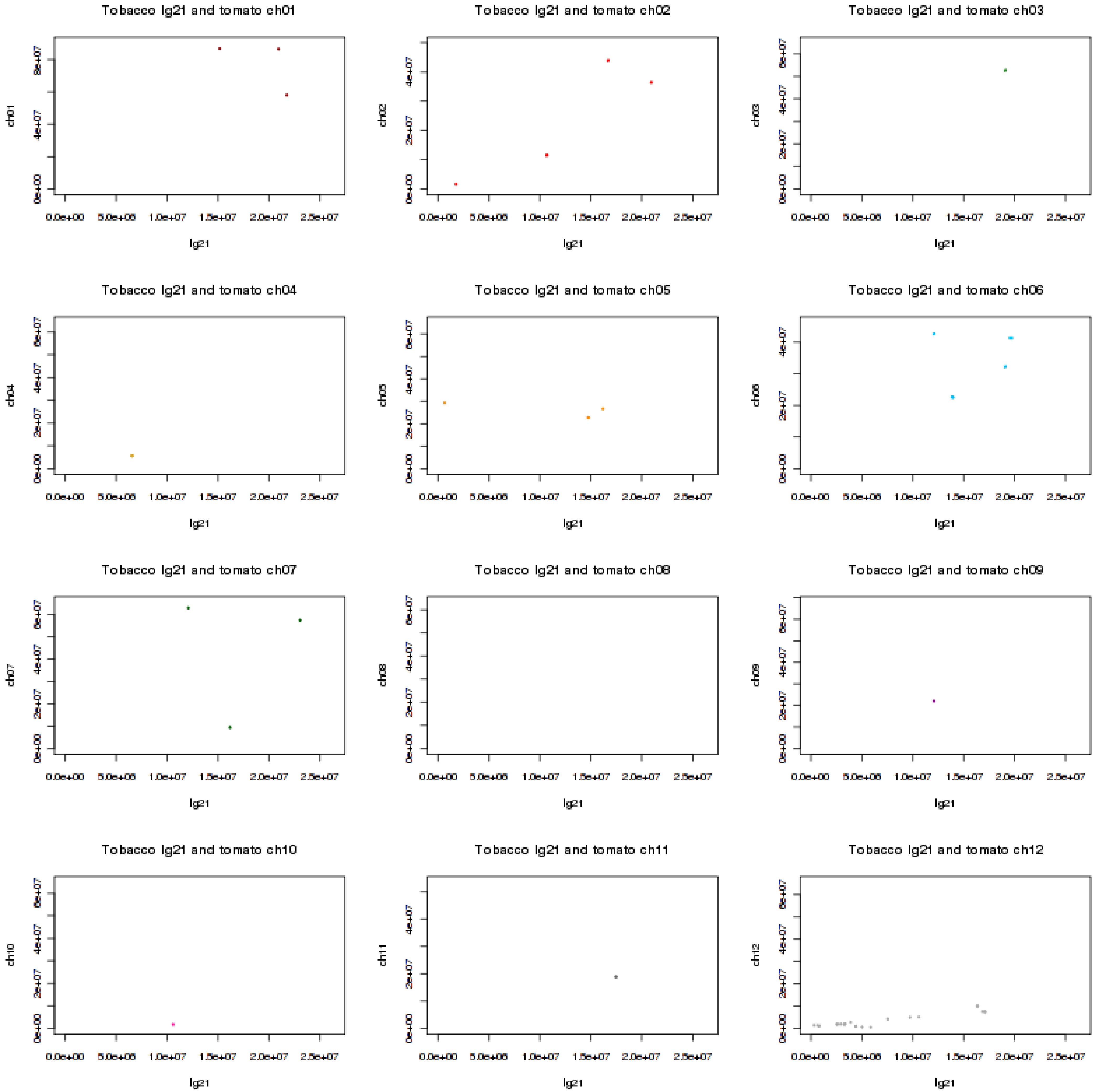

Tobacco lg21 and tomato ch01
Tobacco lg21 and tomato ch02
Tobacco lg21 and tomato ch03
Tobacco lg21 and tomato ch04
Tobacco lg21 and tomato ch05
Tobacco lg21 and tomato ch06
Tobacco lg21 and tomato ch07
Tobacco lg21 and tomato ch08
Tobacco lg21 and tomato ch09
Tobacco lg21 and tomato ch10
Tobacco lg21 and tomato ch11
Tobacco lg21 and tomato ch12
ch01
ch02
ch03
ch04
ch05
ch06
ch07
ch08
ch09
ch10
ch11
ch12
lg21
0.0e+00
5.0e+06
1.0e+07
1.5e+07
2.0e+07
2.5e+07
0e+00
2e+07
4e+07
6e+07
8e+07

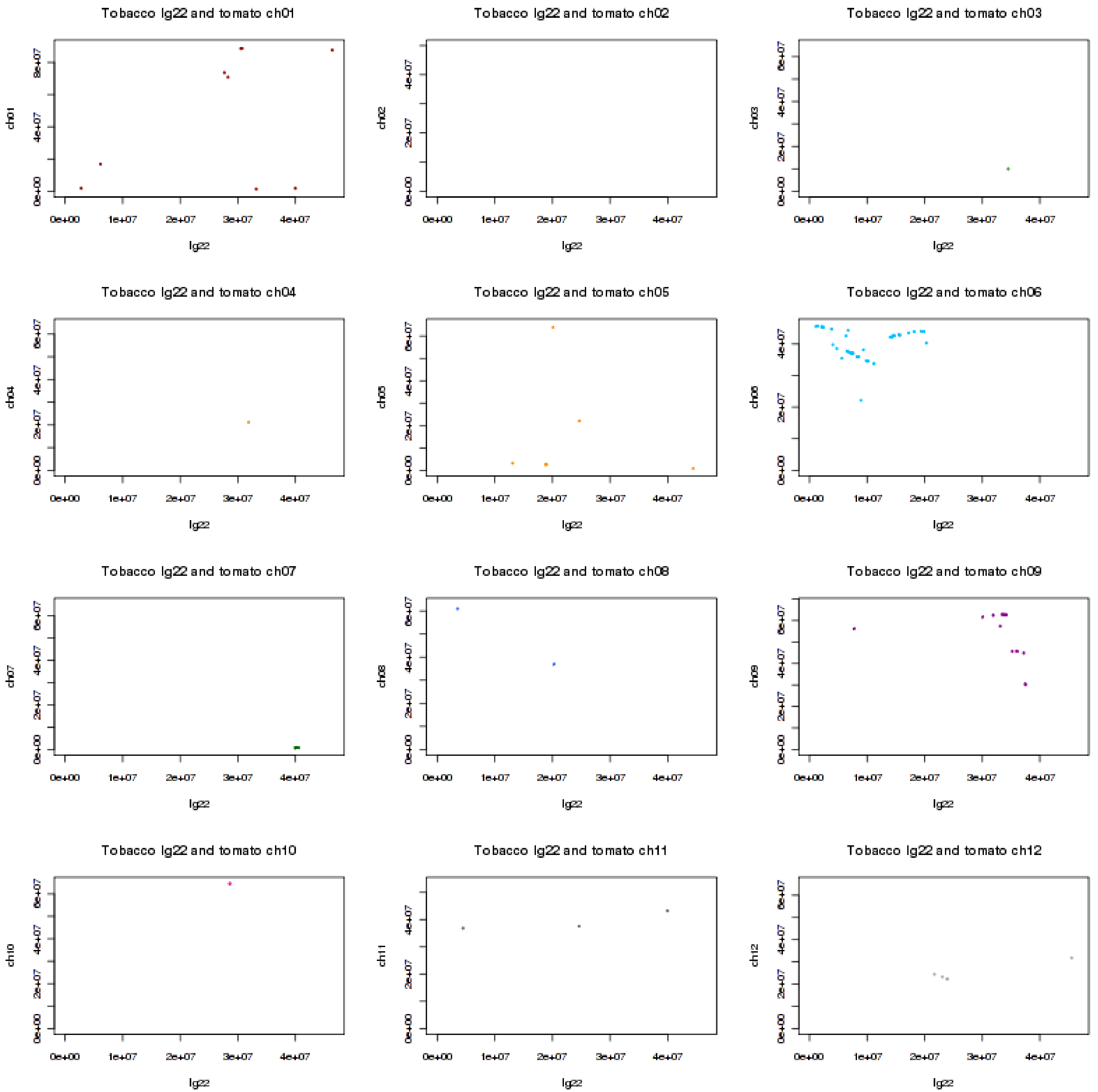

Tobacco lg22 and tomato ch01
Tobacco lg22 and tomato ch02
Tobacco lg22 and tomato ch03
Tobacco lg22 and tomato ch04
Tobacco lg22 and tomato ch05
Tobacco lg22 and tomato ch06
Tobacco lg22 and tomato ch07
Tobacco lg22 and tomato ch08
Tobacco lg22 and tomato ch09
Tobacco lg22 and tomato ch10
Tobacco lg22 and tomato ch11
Tobacco lg22 and tomato ch12
lg22
ch01
ch02
ch03
ch04
ch05
ch06
ch07
ch08
ch09
ch10
ch11
ch12
0e+00
1e+07
2e+07
3e+07
4e+07
6e+07
8e+07

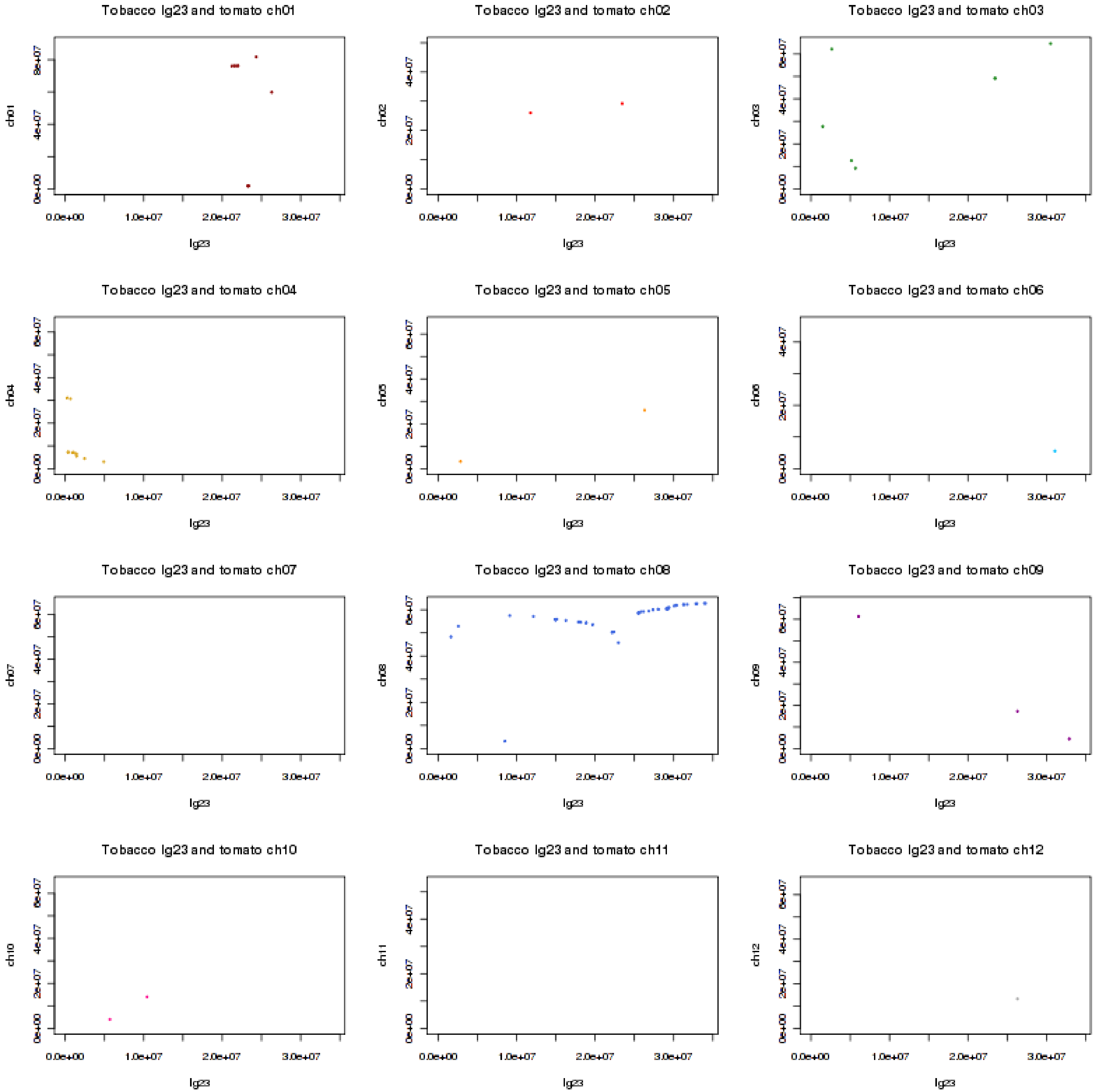

Tobacco lg23 and tomato ch01
Tobacco lg23 and tomato ch02
Tobacco lg23 and tomato ch03
Tobacco lg23 and tomato ch04
Tobacco lg23 and tomato ch05
Tobacco lg23 and tomato ch06
Tobacco lg23 and tomato ch07
Tobacco lg23 and tomato ch08
Tobacco lg23 and tomato ch09
Tobacco lg23 and tomato ch10
Tobacco lg23 and tomato ch11
Tobacco lg23 and tomato ch12
lg23
ch01
ch02
ch03
ch04
ch05
ch06
ch07
ch08
ch09
ch10
ch11
ch12
0.0e+00
1.0e+07
2.0e+07
3.0e+07
0e+00
2e+07
4e+07
6e+07
8e+07

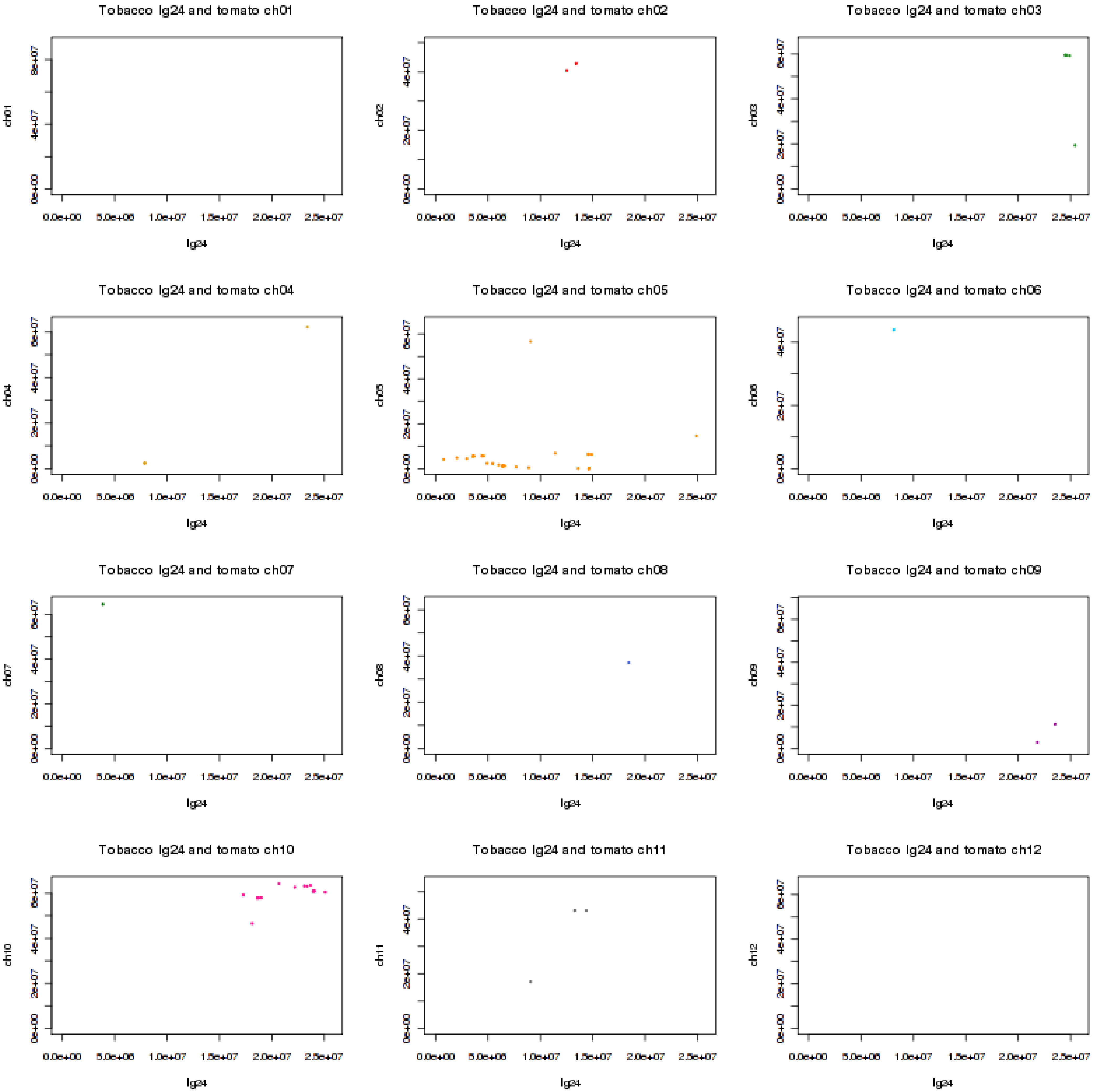
Tobacco lg24 and tomato ch01
Tobacco lg24 and tomato ch02
Tobacco lg24 and tomato ch03
Tobacco lg24 and tomato ch04
Tobacco lg24 and tomato ch05
Tobacco lg24 and tomato ch06
Tobacco lg24 and tomato ch07
Tobacco lg24 and tomato ch08
Tobacco lg24 and tomato ch09
Tobacco lg24 and tomato ch10
Tobacco lg24 and tomato ch11
Tobacco lg24 and tomato ch12
lg24
0.0e+00
5.0e+06
1.0e+07
1.5e+07
2.0e+07
2.5e+07
