# Supplementary material for: The tobacco genome sequence and its comparison with those of tomato and potato
Source: Nat Commun. 2014 May 8;5:3833. doi: 10.1038/ncomms4833 (PMC4024737; doi:10.1038/ncomms4833)
Supplement: Supplementary Data 6 — Synteny of the 24 Nicotiana tabacum linkage groups with the 12 potato chromosomes, based on whole genome sequences. Syntenic DNA blocks in each plot are positioned on the x axis according to their location in the Nicotiana tabacum linkage group, and on the y axis according to their location on the potato chromosome. [file ncomms4833-s7.pdf]

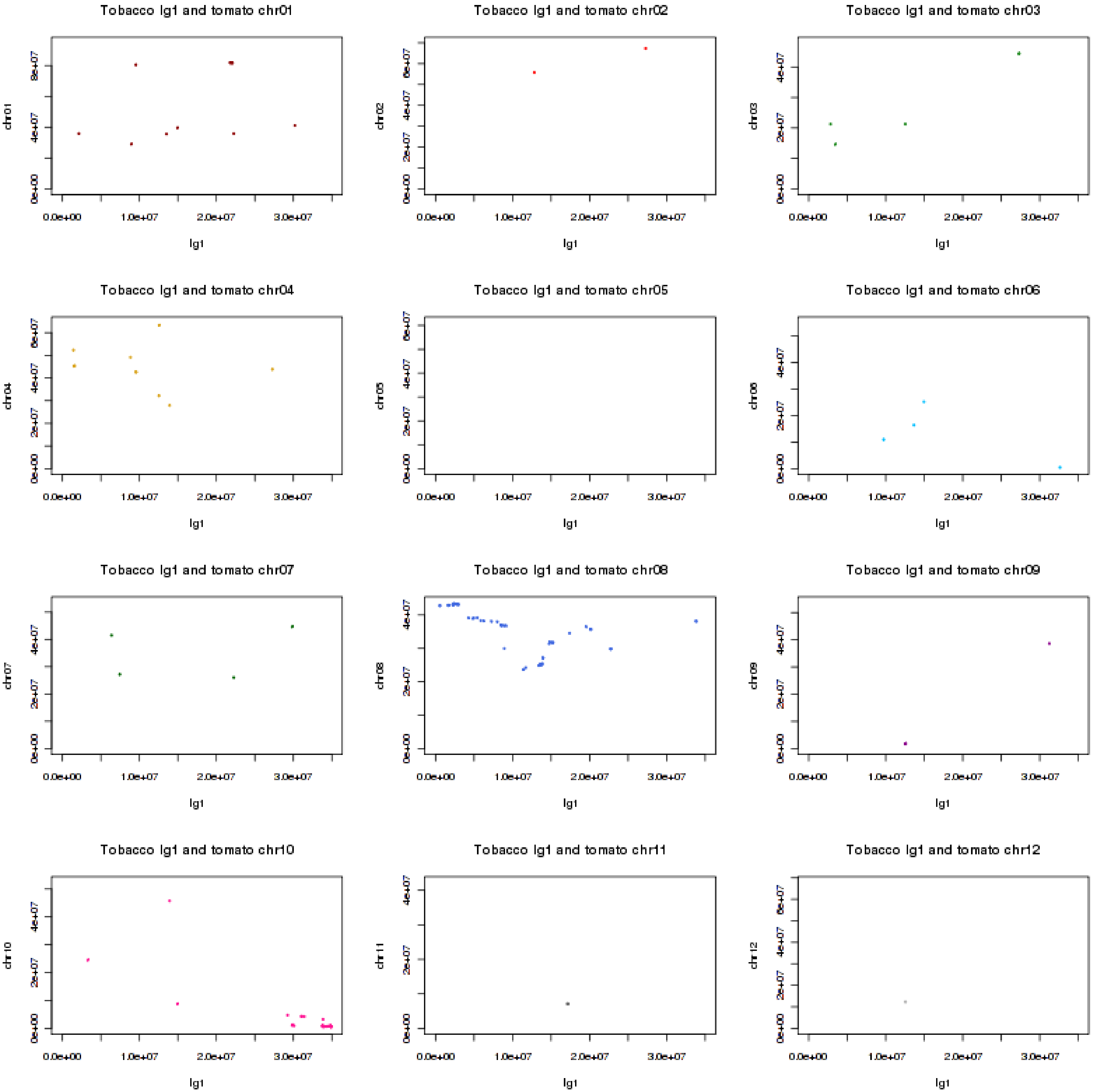
Tobacco lg1 and tomato chr01
Tobacco lg1 and tomato chr02
Tobacco lg1 and tomato chr03
Tobacco lg1 and tomato chr04
Tobacco lg1 and tomato chr05
Tobacco lg1 and tomato chr06
Tobacco lg1 and tomato chr07
Tobacco lg1 and tomato chr08
Tobacco lg1 and tomato chr09
Tobacco lg1 and tomato chr10
Tobacco lg1 and tomato chr11
Tobacco lg1 and tomato chr12
lg1
chr01
chr02
chr03
chr04
chr05
chr06
chr07
chr08
chr09
chr10
chr11
chr12
0.0e+00
1.0e+07
2.0e+07
3.0e+07
0e+00
2e+07
4e+07
6e+07
8e+07

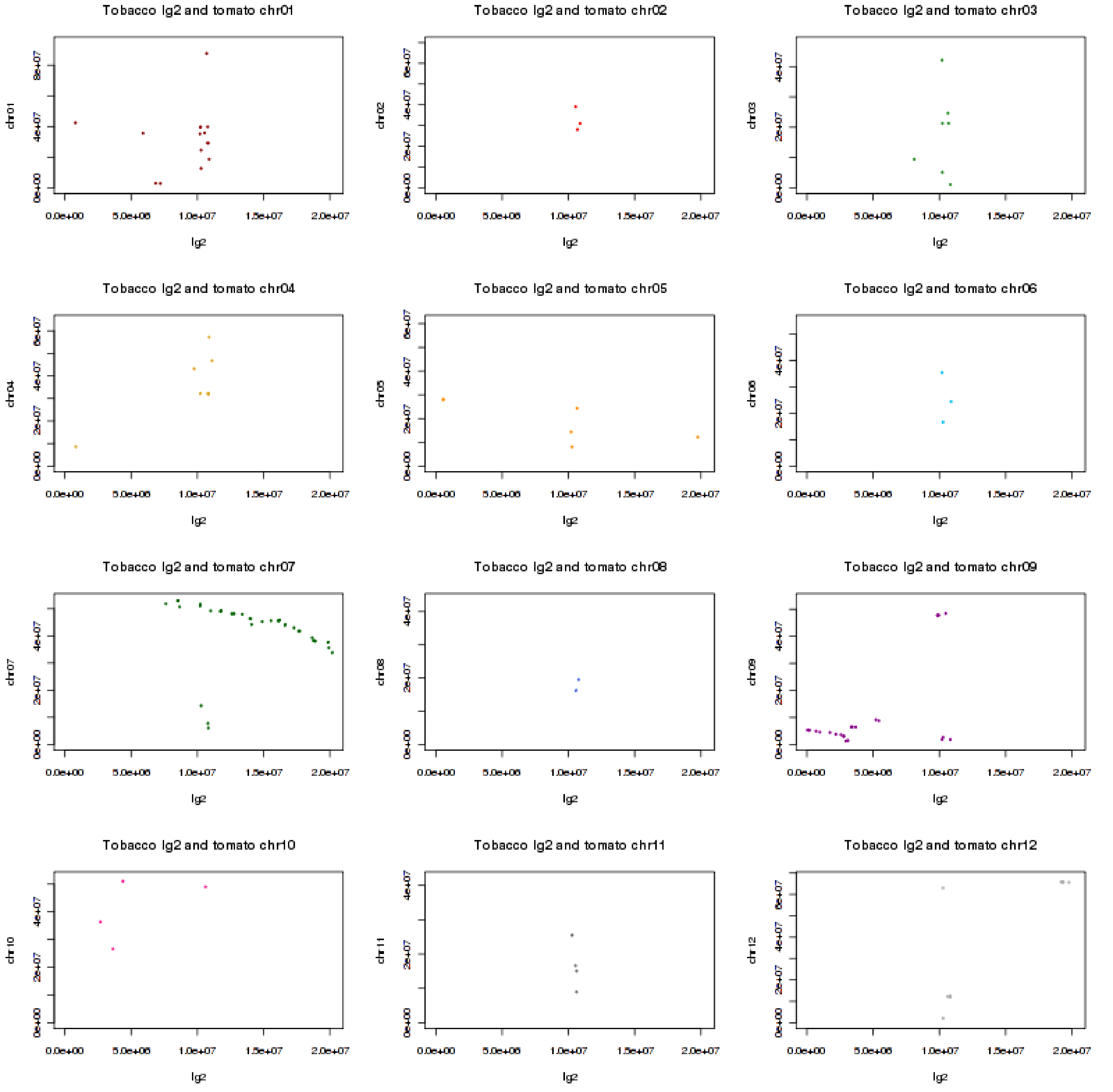
Tobacco lg2 and tomato chr01
chr01
lg2
Tobacco lg2 and tomato chr02
chr02
lg2
Tobacco lg2 and tomato chr03
chr03
lg2
Tobacco lg2 and tomato chr04
chr04
lg2
Tobacco lg2 and tomato chr05
chr05
lg2
Tobacco lg2 and tomato chr06
chr06
lg2
Tobacco lg2 and tomato chr07
chr07
lg2
Tobacco lg2 and tomato chr08
chr08
lg2
Tobacco lg2 and tomato chr09
chr09
lg2
Tobacco lg2 and tomato chr10
chr10
lg2
Tobacco lg2 and tomato chr11
chr11
lg2
Tobacco lg2 and tomato chr12
chr12
lg2
0.0e+00
5.0e+06
1.0e+07
1.5e+07
2.0e+07

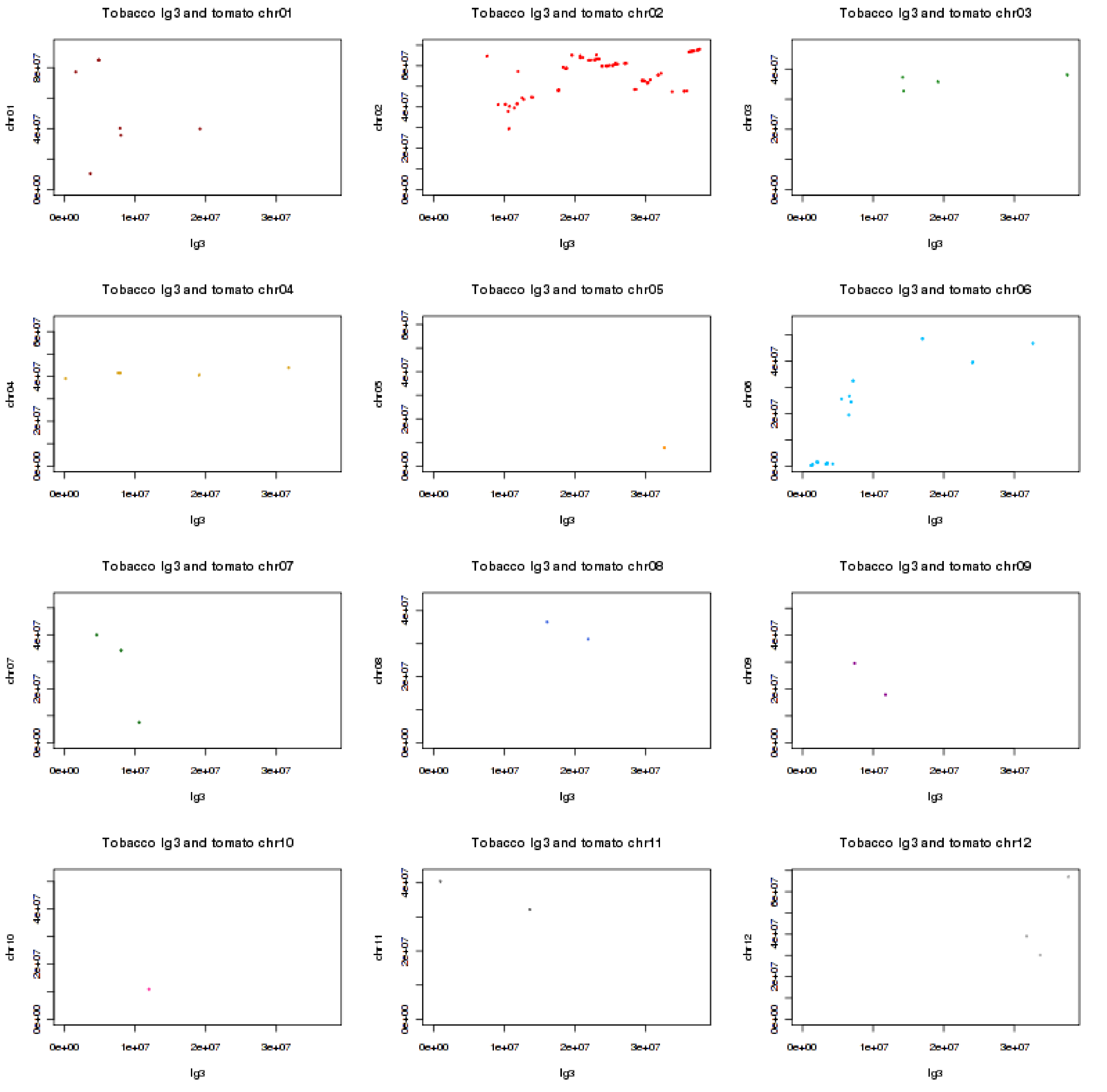
Tobacco lg3 and tomato chr01
Tobacco lg3 and tomato chr02
Tobacco lg3 and tomato chr03
Tobacco lg3 and tomato chr04
Tobacco lg3 and tomato chr05
Tobacco lg3 and tomato chr06
Tobacco lg3 and tomato chr07
Tobacco lg3 and tomato chr08
Tobacco lg3 and tomato chr09
Tobacco lg3 and tomato chr10
Tobacco lg3 and tomato chr11
Tobacco lg3 and tomato chr12
lg3
chr01
chr02
chr03
chr04
chr05
chr06
chr07
chr08
chr09
chr10
chr11
chr12
0e+00
1e+07
2e+07
3e+07

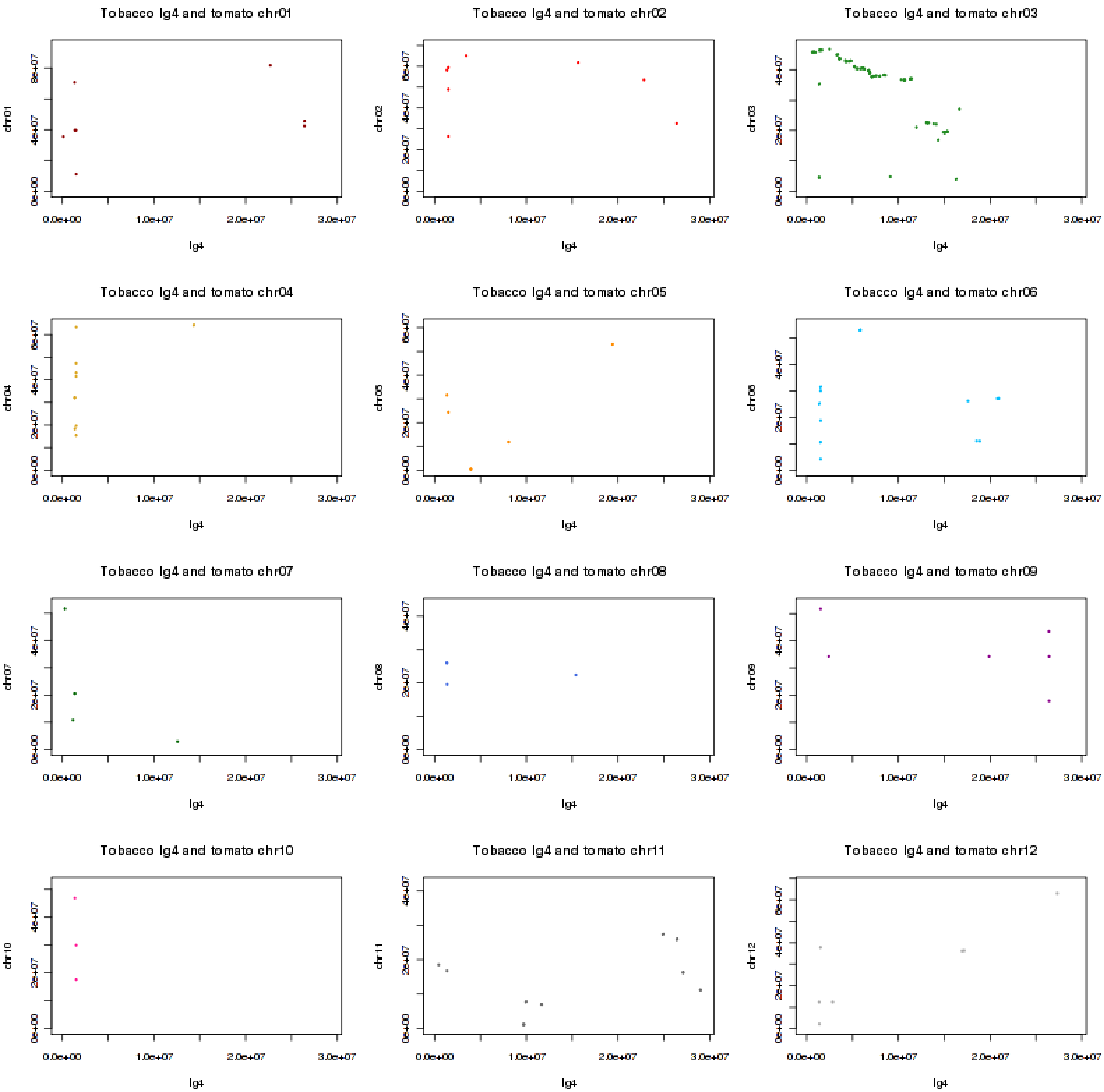
Tobacco lg4 and tomato chr01
chr01
lg4
Tobacco lg4 and tomato chr02
chr02
lg4
Tobacco lg4 and tomato chr03
chr03
lg4
Tobacco lg4 and tomato chr04
chr04
lg4
Tobacco lg4 and tomato chr05
chr05
lg4
Tobacco lg4 and tomato chr06
chr06
lg4
Tobacco lg4 and tomato chr07
chr07
lg4
Tobacco lg4 and tomato chr08
chr08
lg4
Tobacco lg4 and tomato chr09
chr09
lg4
Tobacco lg4 and tomato chr10
chr10
lg4
Tobacco lg4 and tomato chr11
chr11
lg4
Tobacco lg4 and tomato chr12
chr12
lg4

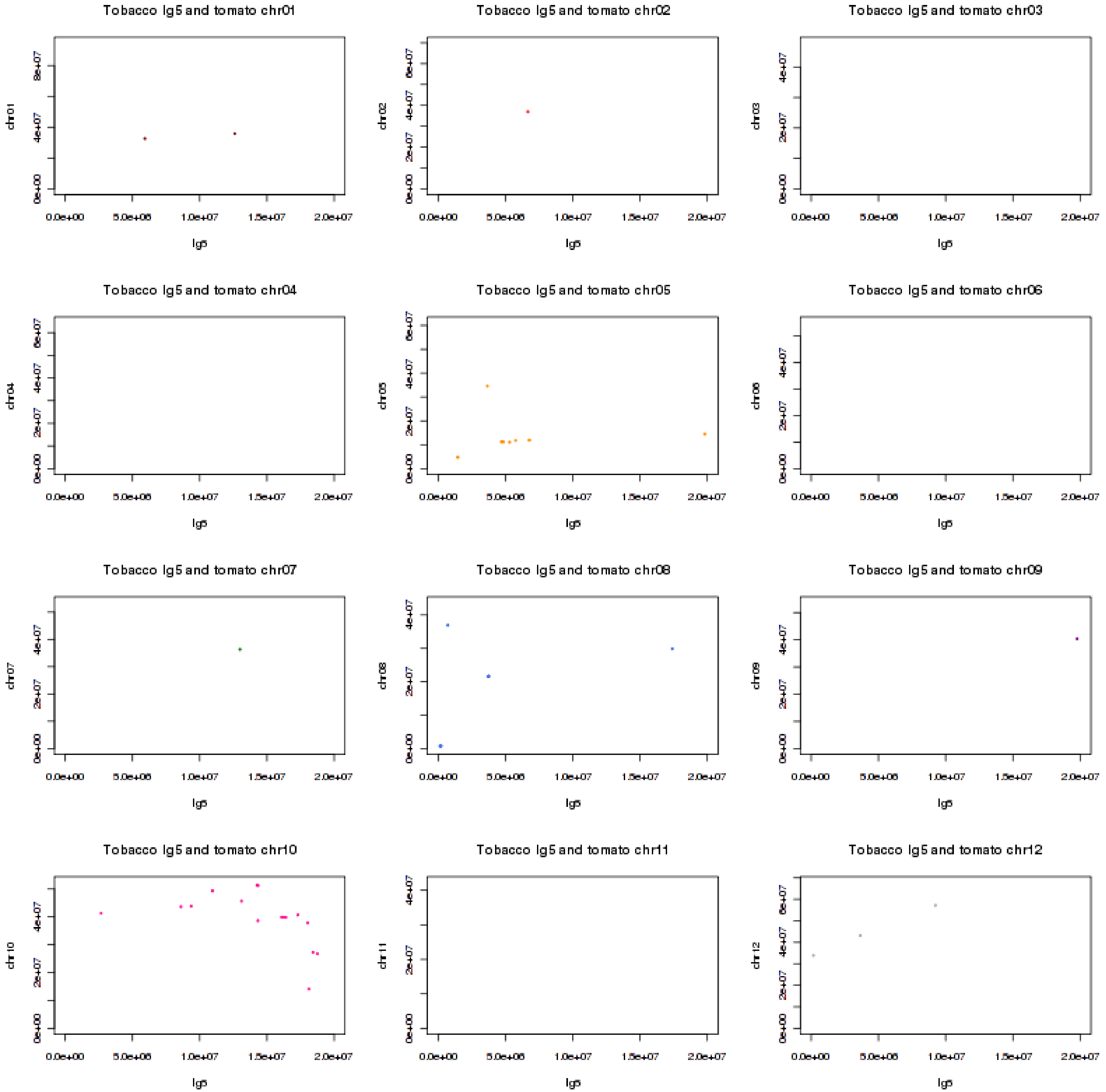
Tobacco lg5 and tomato chr01
chr01
lg5
Tobacco lg5 and tomato chr02
chr02
lg5
Tobacco lg5 and tomato chr03
chr03
lg5
Tobacco lg5 and tomato chr04
chr04
lg5
Tobacco lg5 and tomato chr05
chr05
lg5
Tobacco lg5 and tomato chr06
chr06
lg5
Tobacco lg5 and tomato chr07
chr07
lg5
Tobacco lg5 and tomato chr08
chr08
lg5
Tobacco lg5 and tomato chr09
chr09
lg5
Tobacco lg5 and tomato chr10
chr10
lg5
Tobacco lg5 and tomato chr11
chr11
lg5
Tobacco lg5 and tomato chr12
chr12
lg5
0.0e+00
5.0e+06
1.0e+07
1.5e+07
2.0e+07

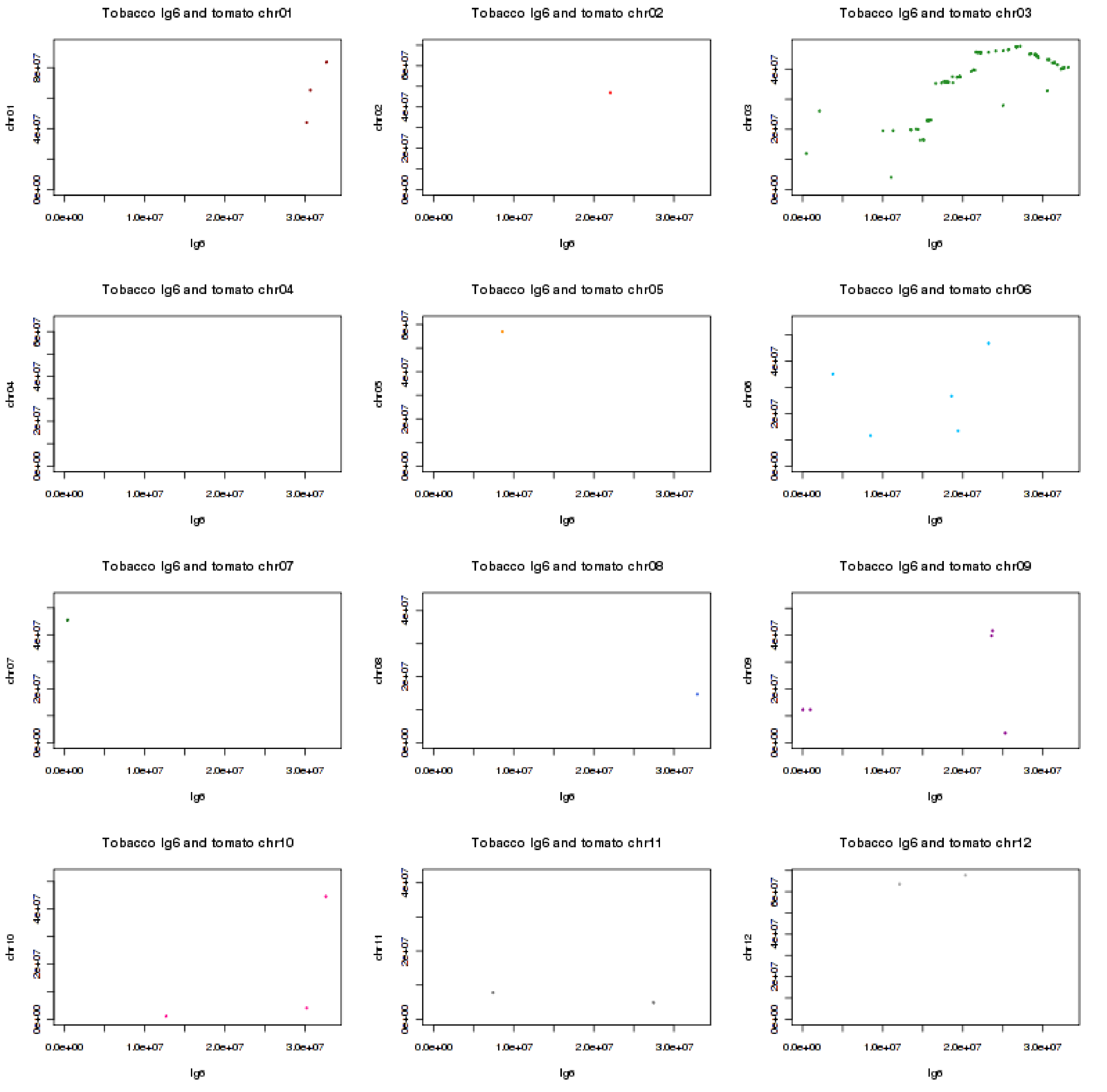

Tobacco lg6 and tomato chr01
chr01
lg6
0e+00
4e+07
8e+07
Tobacco lg6 and tomato chr02
chr02
lg6
0e+00
2e+07
4e+07
6e+07
Tobacco lg6 and tomato chr03
chr03
lg6
0e+00
2e+07
4e+07
Tobacco lg6 and tomato chr04
chr04
lg6
0e+00
2e+07
4e+07
6e+07
Tobacco lg6 and tomato chr05
chr05
lg6
0e+00
2e+07
4e+07
6e+07
Tobacco lg6 and tomato chr06
chr06
lg6
0e+00
2e+07
4e+07
Tobacco lg6 and tomato chr07
chr07
lg6
0e+00
2e+07
4e+07
Tobacco lg6 and tomato chr08
chr08
lg6
0e+00
2e+07
4e+07
Tobacco lg6 and tomato chr09
chr09
lg6
0e+00
2e+07
4e+07
Tobacco lg6 and tomato chr10
chr10
lg6
0e+00
2e+07
4e+07
Tobacco lg6 and tomato chr11
chr11
lg6
0e+00
2e+07
4e+07
Tobacco lg6 and tomato chr12
chr12
lg6
0e+00
2e+07
4e+07
6e+07
0.0e+00
1.0e+07
2.0e+07
3.0e+07

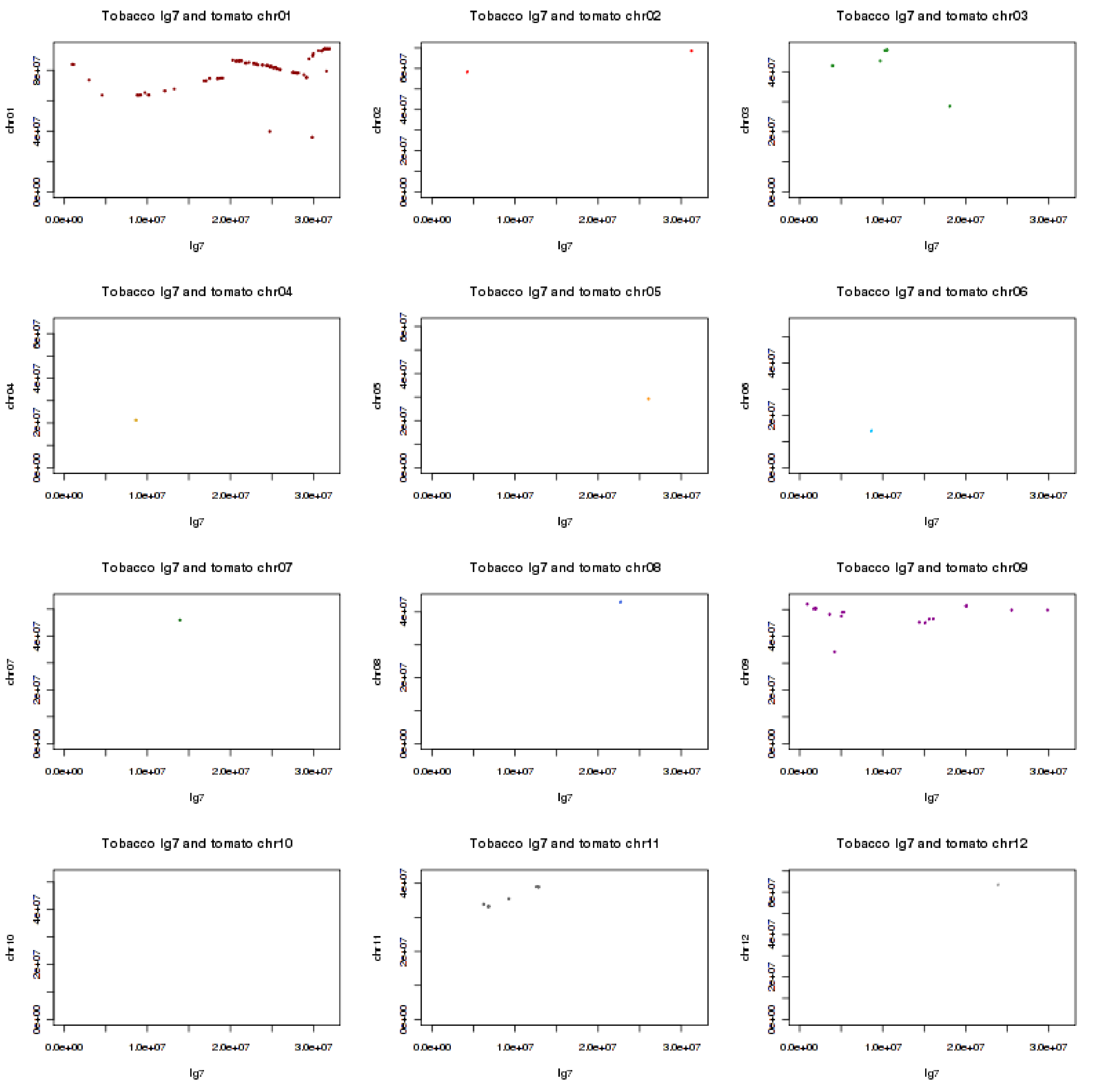
Tobacco lg7 and tomato chr01
Tobacco lg7 and tomato chr02
Tobacco lg7 and tomato chr03
Tobacco lg7 and tomato chr04
Tobacco lg7 and tomato chr05
Tobacco lg7 and tomato chr06
Tobacco lg7 and tomato chr07
Tobacco lg7 and tomato chr08
Tobacco lg7 and tomato chr09
Tobacco lg7 and tomato chr10
Tobacco lg7 and tomato chr11
Tobacco lg7 and tomato chr12
lg7
0.0e+00
1.0e+07
2.0e+07
3.0e+07

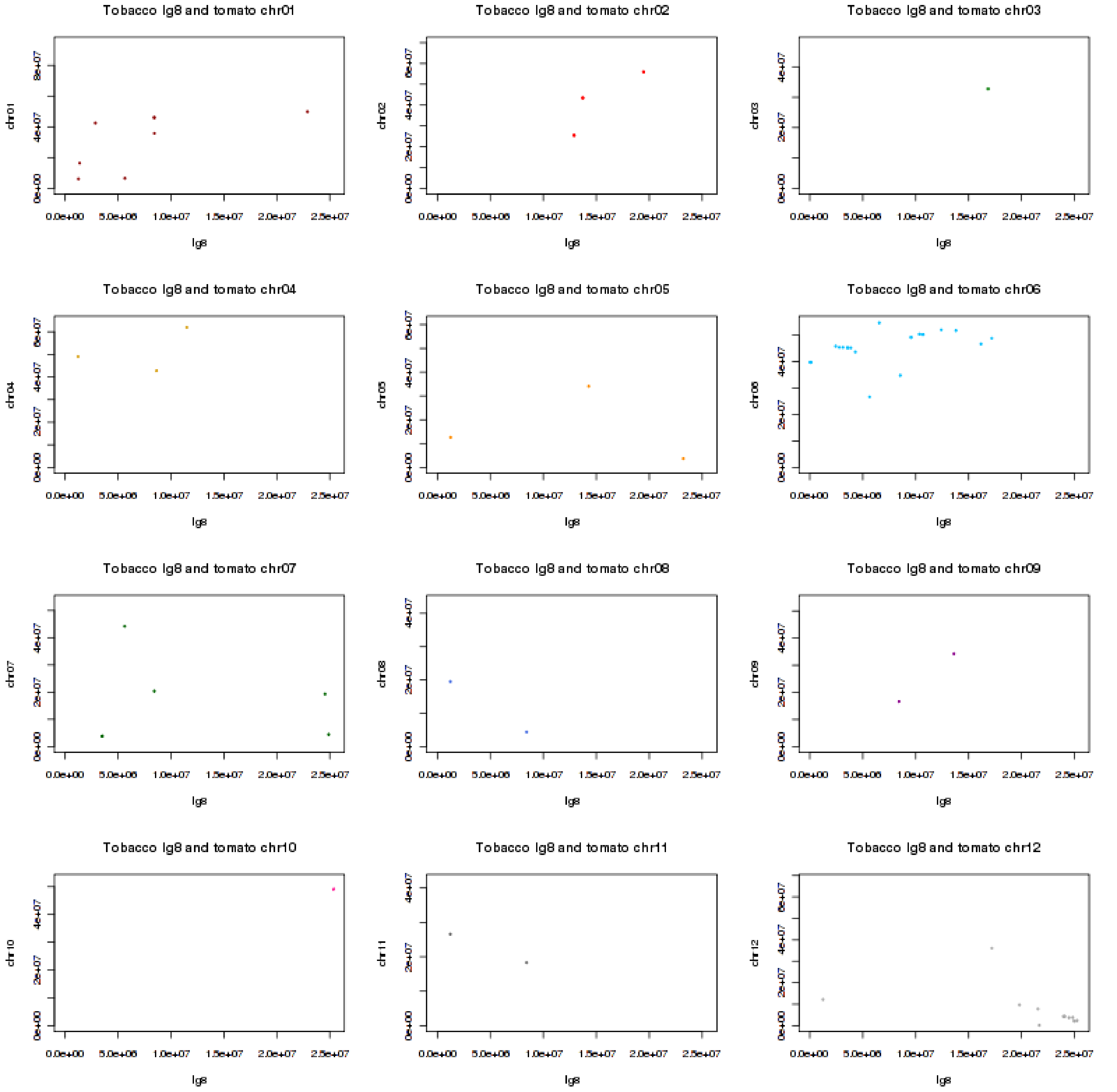

Tobacco lg8 and tomato chr01
Tobacco lg8 and tomato chr02
Tobacco lg8 and tomato chr03
Tobacco lg8 and tomato chr04
Tobacco lg8 and tomato chr05
Tobacco lg8 and tomato chr06
Tobacco lg8 and tomato chr07
Tobacco lg8 and tomato chr08
Tobacco lg8 and tomato chr09
Tobacco lg8 and tomato chr10
Tobacco lg8 and tomato chr11
Tobacco lg8 and tomato chr12
lg8
chr01
chr02
chr03
chr04
chr05
chr06
chr07
chr08
chr09
chr10
chr11
chr12
0.0e+00
5.0e+06
1.0e+07
1.5e+07
2.0e+07
2.5e+07
0e+00
2e+07
4e+07
6e+07
8e+07

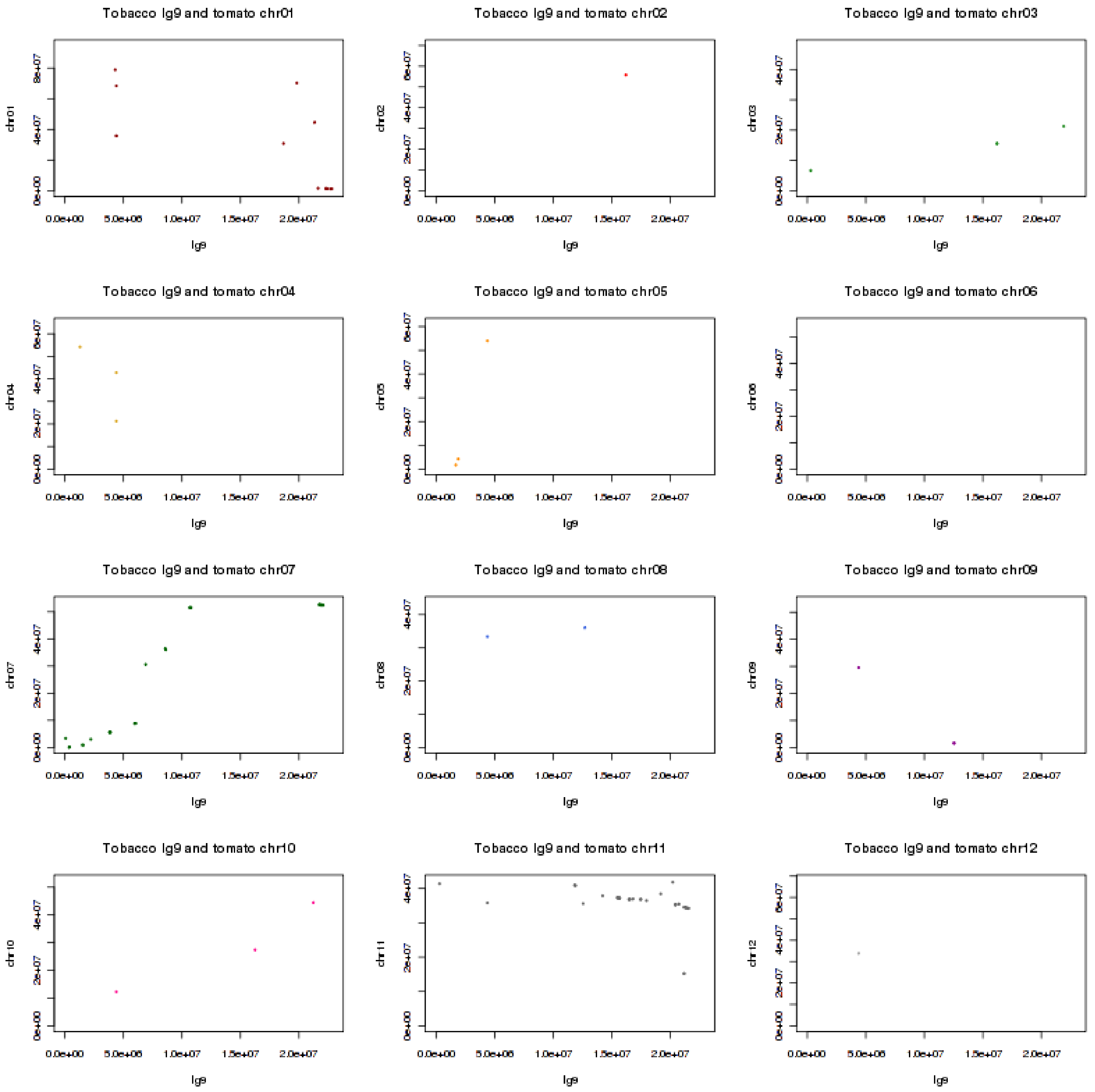
Tobacco lg9 and tomato chr01
chr01
lg9
Tobacco lg9 and tomato chr02
chr02
lg9
Tobacco lg9 and tomato chr03
chr03
lg9
Tobacco lg9 and tomato chr04
chr04
lg9
Tobacco lg9 and tomato chr05
chr05
lg9
Tobacco lg9 and tomato chr06
chr06
lg9
Tobacco lg9 and tomato chr07
chr07
lg9
Tobacco lg9 and tomato chr08
chr08
lg9
Tobacco lg9 and tomato chr09
chr09
lg9
Tobacco lg9 and tomato chr10
chr10
lg9
Tobacco lg9 and tomato chr11
chr11
lg9
Tobacco lg9 and tomato chr12
chr12
lg9

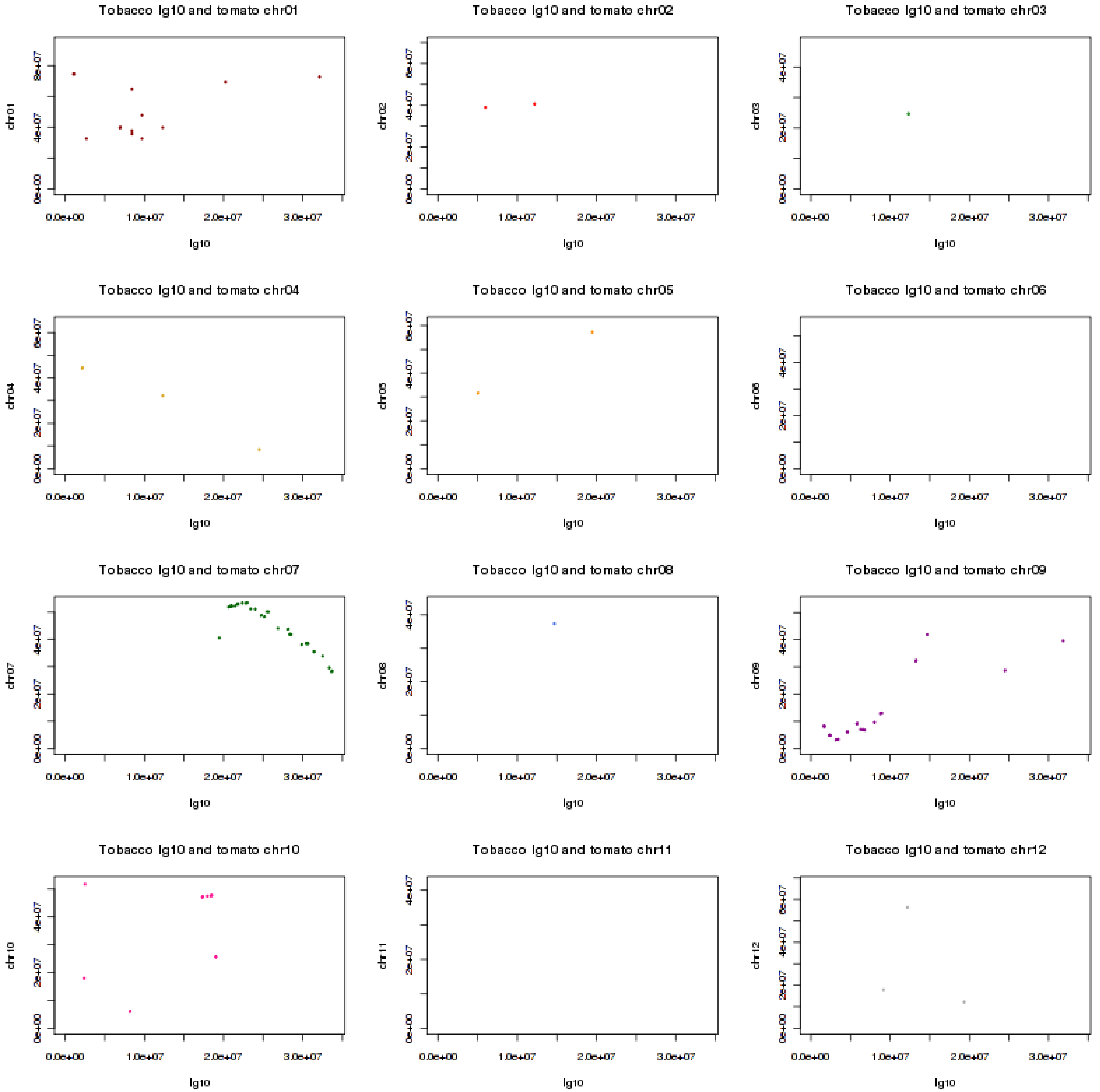
Tobacco lg10 and tomato chr01
Tobacco lg10 and tomato chr02
Tobacco lg10 and tomato chr03
Tobacco lg10 and tomato chr04
Tobacco lg10 and tomato chr05
Tobacco lg10 and tomato chr06
Tobacco lg10 and tomato chr07
Tobacco lg10 and tomato chr08
Tobacco lg10 and tomato chr09
Tobacco lg10 and tomato chr10
Tobacco lg10 and tomato chr11
Tobacco lg10 and tomato chr12
lg10
chr01
chr02
chr03
chr04
chr05
chr06
chr07
chr08
chr09
chr10
chr11
chr12
0.0e+00
1.0e+07
2.0e+07
3.0e+07
0e+00
2e+07
4e+07
6e+07
8e+07

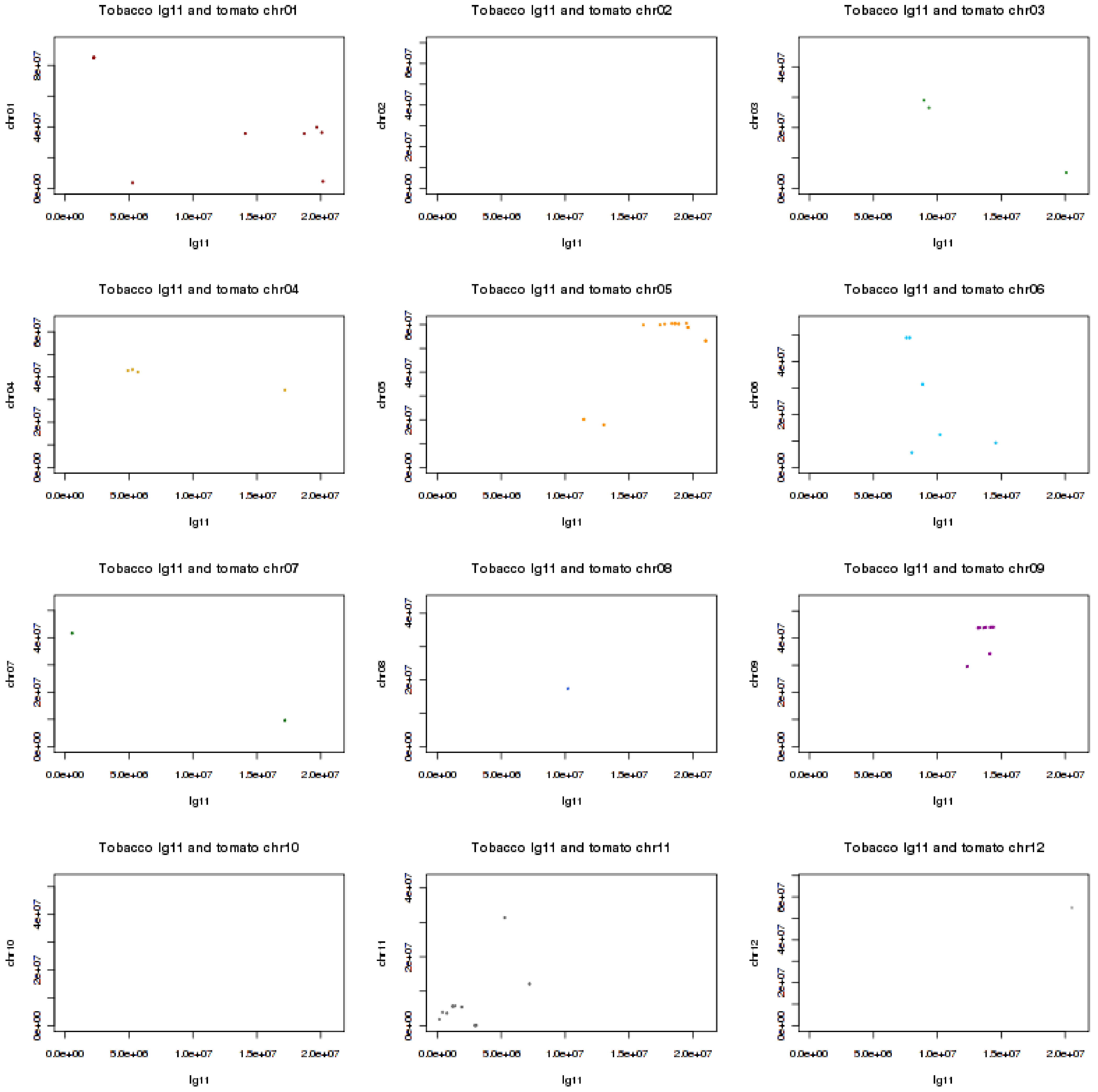
Tobacco lg11 and tomato chr01
chr01
lg11
Tobacco lg11 and tomato chr02
chr02
lg11
Tobacco lg11 and tomato chr03
chr03
lg11
Tobacco lg11 and tomato chr04
chr04
lg11
Tobacco lg11 and tomato chr05
chr05
lg11
Tobacco lg11 and tomato chr06
chr06
lg11
Tobacco lg11 and tomato chr07
chr07
lg11
Tobacco lg11 and tomato chr08
chr08
lg11
Tobacco lg11 and tomato chr09
chr09
lg11
Tobacco lg11 and tomato chr10
chr10
lg11
Tobacco lg11 and tomato chr11
chr11
lg11
Tobacco lg11 and tomato chr12
chr12
lg11

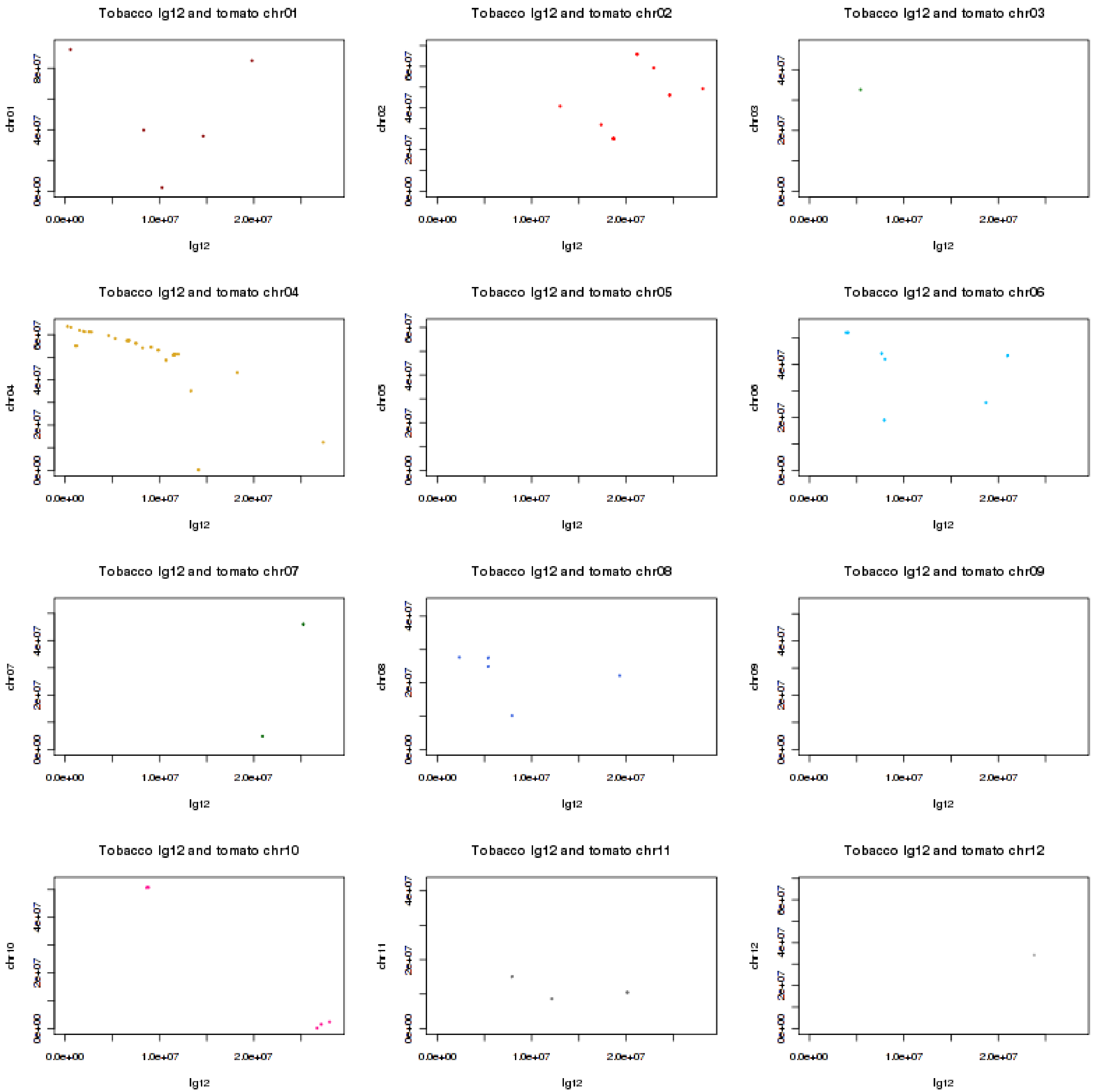
Tobacco lg12 and tomato chr01
Tobacco lg12 and tomato chr02
Tobacco lg12 and tomato chr03
Tobacco lg12 and tomato chr04
Tobacco lg12 and tomato chr05
Tobacco lg12 and tomato chr06
Tobacco lg12 and tomato chr07
Tobacco lg12 and tomato chr08
Tobacco lg12 and tomato chr09
Tobacco lg12 and tomato chr10
Tobacco lg12 and tomato chr11
Tobacco lg12 and tomato chr12
lg12
chr01
chr02
chr03
chr04
chr05
chr06
chr07
chr08
chr09
chr10
chr11
chr12
0.0e+00
1.0e+07
2.0e+07
0e+00
2e+07
4e+07
6e+07
8e+07

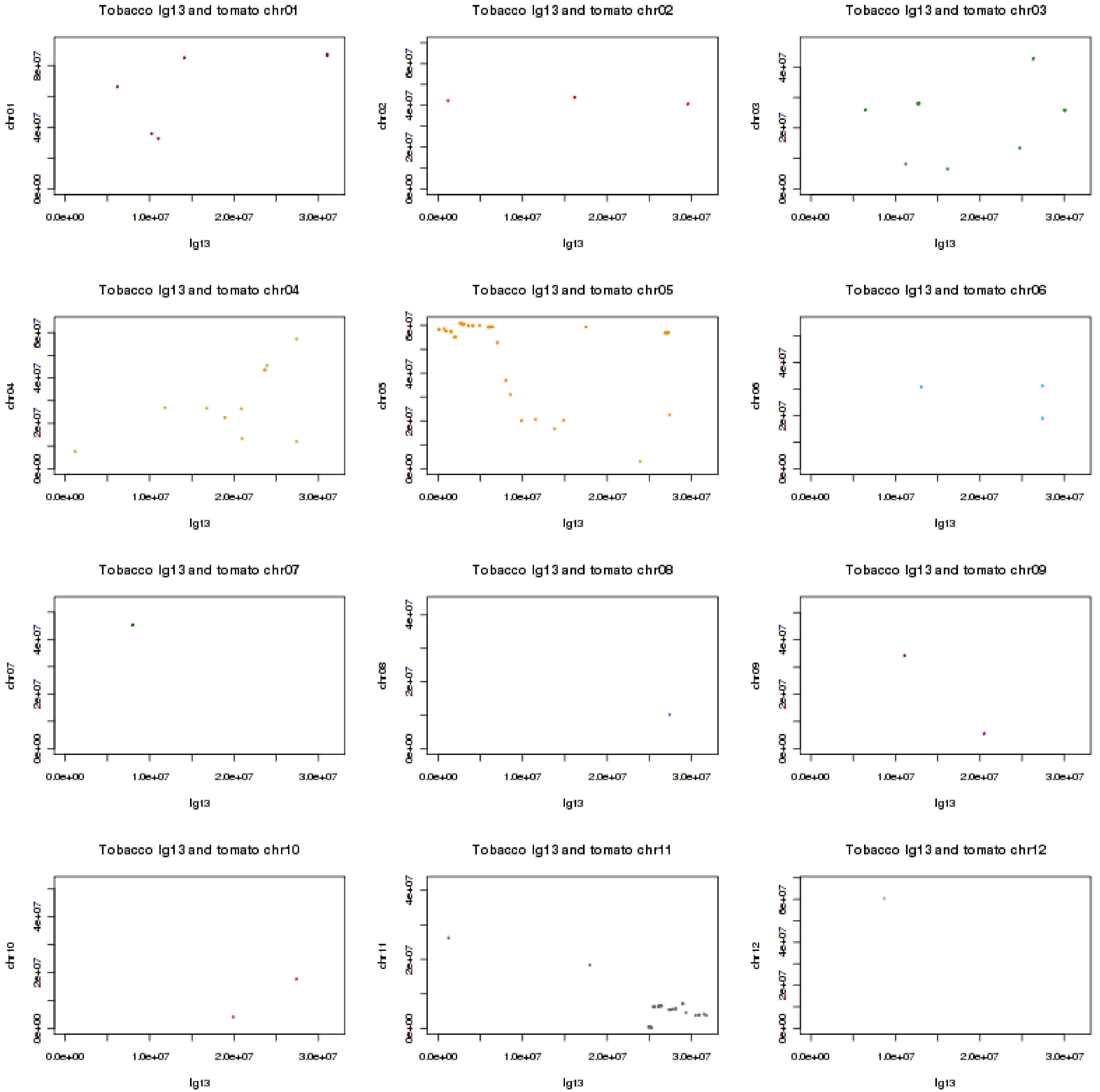
Tobacco lg13 and tomato chr01
chr01
lg13
Tobacco lg13 and tomato chr02
chr02
lg13
Tobacco lg13 and tomato chr03
chr03
lg13
Tobacco lg13 and tomato chr04
chr04
lg13
Tobacco lg13 and tomato chr05
chr05
lg13
Tobacco lg13 and tomato chr06
chr06
lg13
Tobacco lg13 and tomato chr07
chr07
lg13
Tobacco lg13 and tomato chr08
chr08
lg13
Tobacco lg13 and tomato chr09
chr09
lg13
Tobacco lg13 and tomato chr10
chr10
lg13
Tobacco lg13 and tomato chr11
chr11
lg13
Tobacco lg13 and tomato chr12
chr12
lg13
0.0e+00
1.0e+07
2.0e+07
3.0e+07

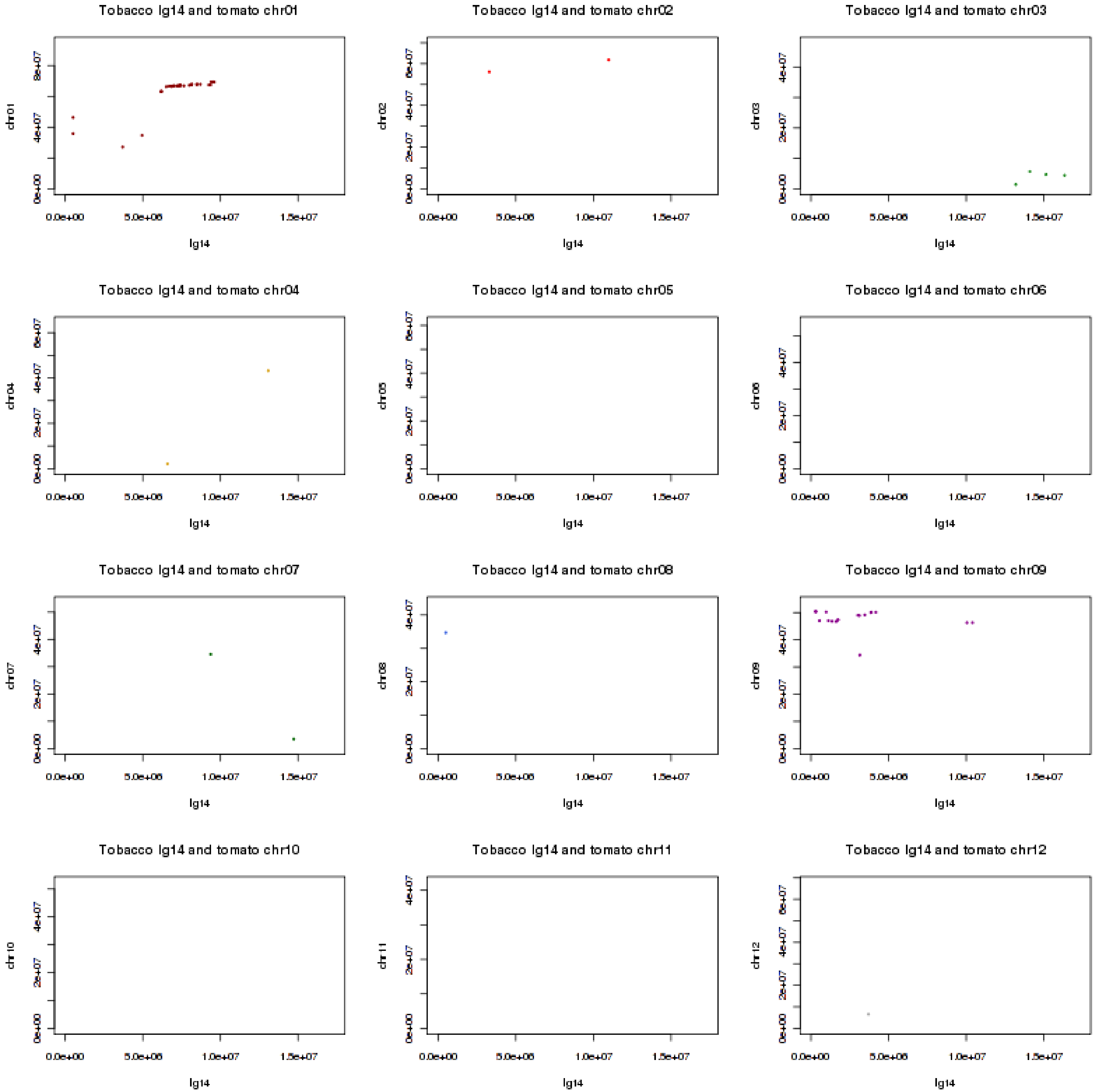

Tobacco lg14 and tomato chr01
Tobacco lg14 and tomato chr02
Tobacco lg14 and tomato chr03
Tobacco lg14 and tomato chr04
Tobacco lg14 and tomato chr05
Tobacco lg14 and tomato chr06
Tobacco lg14 and tomato chr07
Tobacco lg14 and tomato chr08
Tobacco lg14 and tomato chr09
Tobacco lg14 and tomato chr10
Tobacco lg14 and tomato chr11
Tobacco lg14 and tomato chr12
chr01
chr02
chr03
chr04
chr05
chr06
chr07
chr08
chr09
chr10
chr11
chr12
lg14
0.0e+00
5.0e+06
1.0e+07
1.5e+07
0e+00
2e+07
4e+07
6e+07
8e+07

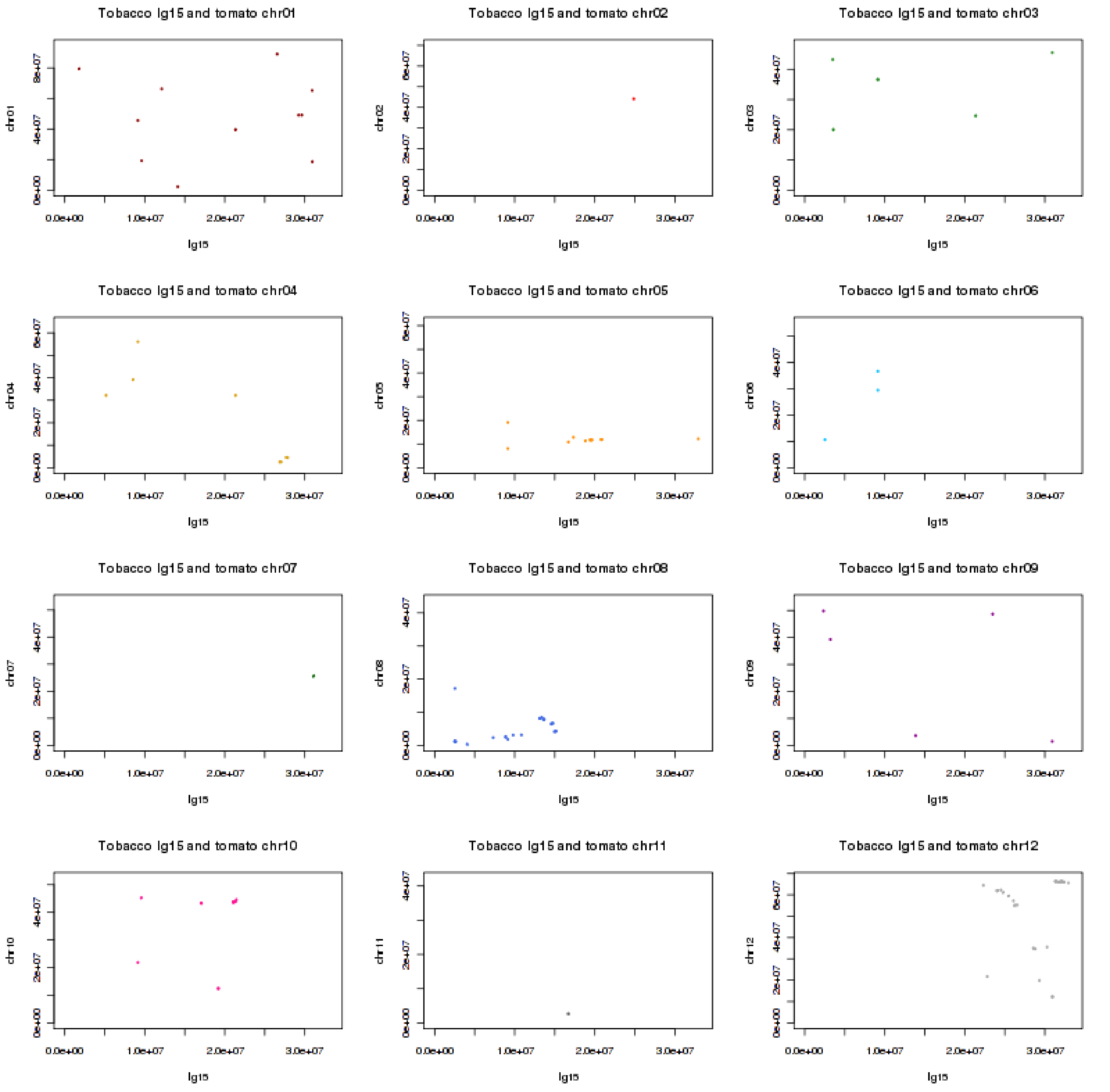
Tobacco lg15 and tomato chr01
chr01
lg15
Tobacco lg15 and tomato chr02
chr02
lg15
Tobacco lg15 and tomato chr03
chr03
lg15
Tobacco lg15 and tomato chr04
chr04
lg15
Tobacco lg15 and tomato chr05
chr05
lg15
Tobacco lg15 and tomato chr06
chr06
lg15
Tobacco lg15 and tomato chr07
chr07
lg15
Tobacco lg15 and tomato chr08
chr08
lg15
Tobacco lg15 and tomato chr09
chr09
lg15
Tobacco lg15 and tomato chr10
chr10
lg15
Tobacco lg15 and tomato chr11
chr11
lg15
Tobacco lg15 and tomato chr12
chr12
lg15
0.0e+00
1.0e+07
2.0e+07
3.0e+07

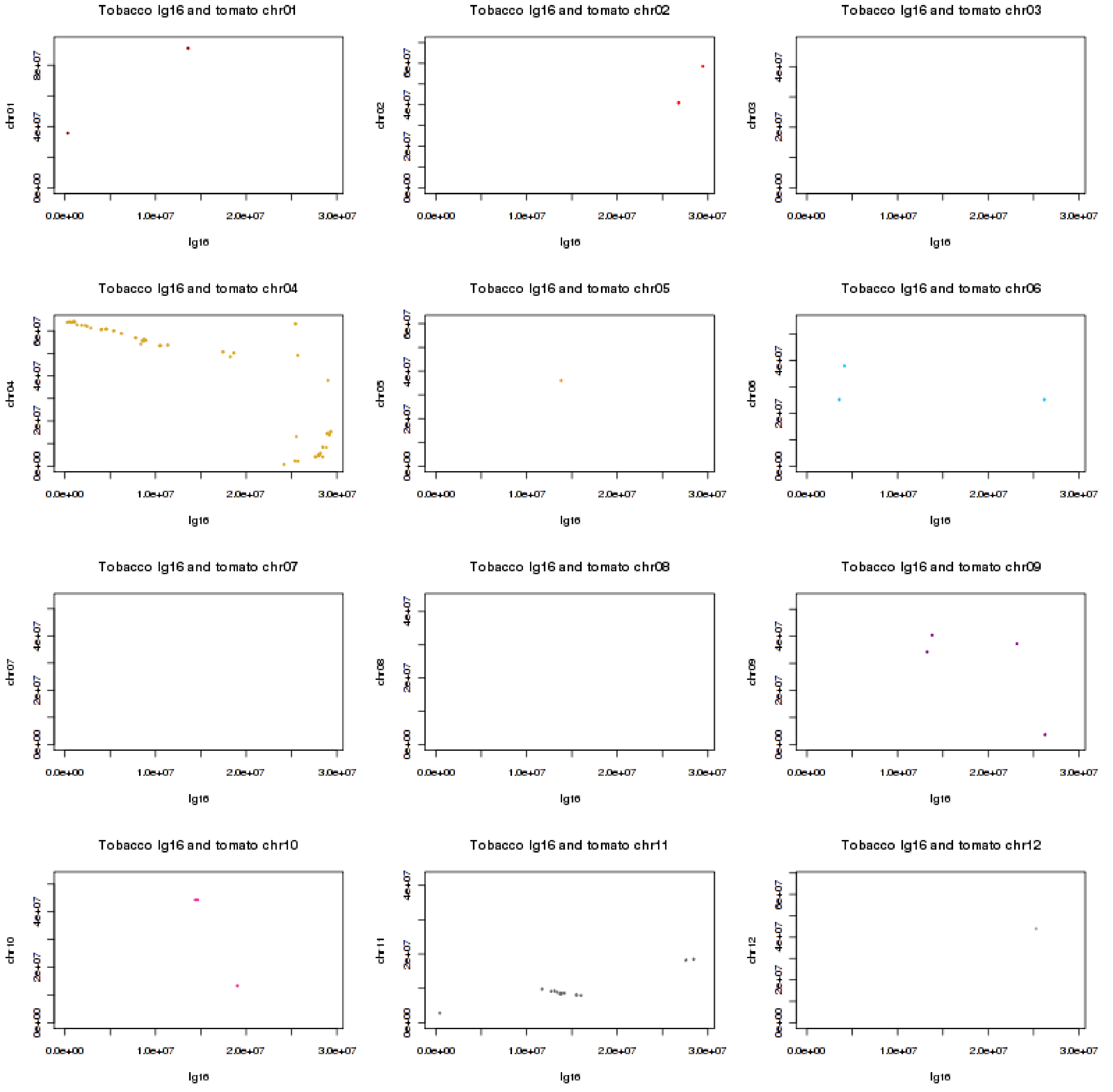
Tobacco lg16 and tomato chr01
chr01
lg16
Tobacco lg16 and tomato chr02
chr02
lg16
Tobacco lg16 and tomato chr03
chr03
lg16
Tobacco lg16 and tomato chr04
chr04
lg16
Tobacco lg16 and tomato chr05
chr05
lg16
Tobacco lg16 and tomato chr06
chr06
lg16
Tobacco lg16 and tomato chr07
chr07
lg16
Tobacco lg16 and tomato chr08
chr08
lg16
Tobacco lg16 and tomato chr09
chr09
lg16
Tobacco lg16 and tomato chr10
chr10
lg16
Tobacco lg16 and tomato chr11
chr11
lg16
Tobacco lg16 and tomato chr12
chr12
lg16
0.0e+00
1.0e+07
2.0e+07
3.0e+07
0e+00
2e+07
4e+07
6e+07
8e+07

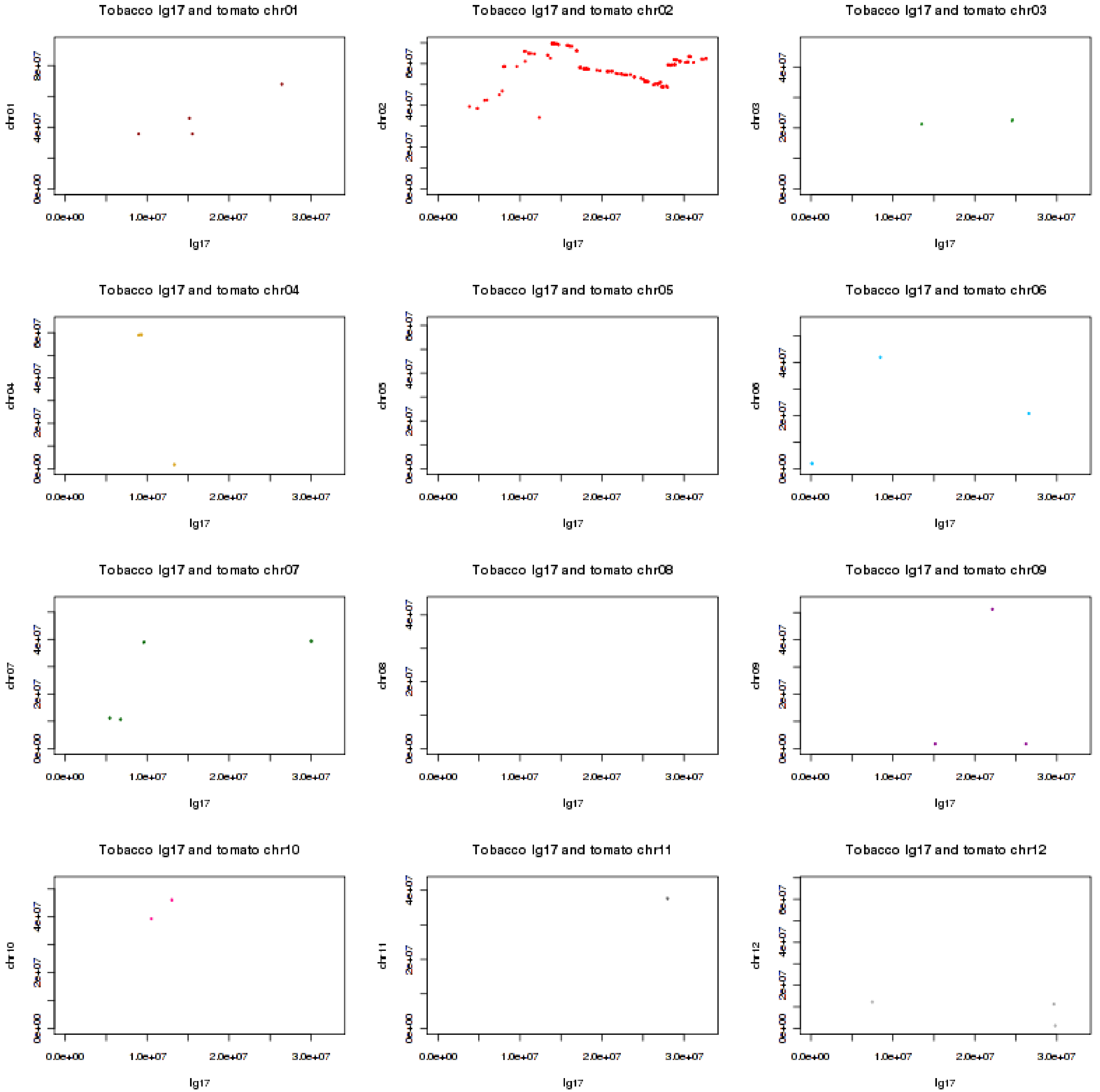
Tobacco lg17 and tomato chr01
Tobacco lg17 and tomato chr02
Tobacco lg17 and tomato chr03
Tobacco lg17 and tomato chr04
Tobacco lg17 and tomato chr05
Tobacco lg17 and tomato chr06
Tobacco lg17 and tomato chr07
Tobacco lg17 and tomato chr08
Tobacco lg17 and tomato chr09
Tobacco lg17 and tomato chr10
Tobacco lg17 and tomato chr11
Tobacco lg17 and tomato chr12
lg17
chr01
chr02
chr03
chr04
chr05
chr06
chr07
chr08
chr09
chr10
chr11
chr12
0.0e+00
1.0e+07
2.0e+07
3.0e+07

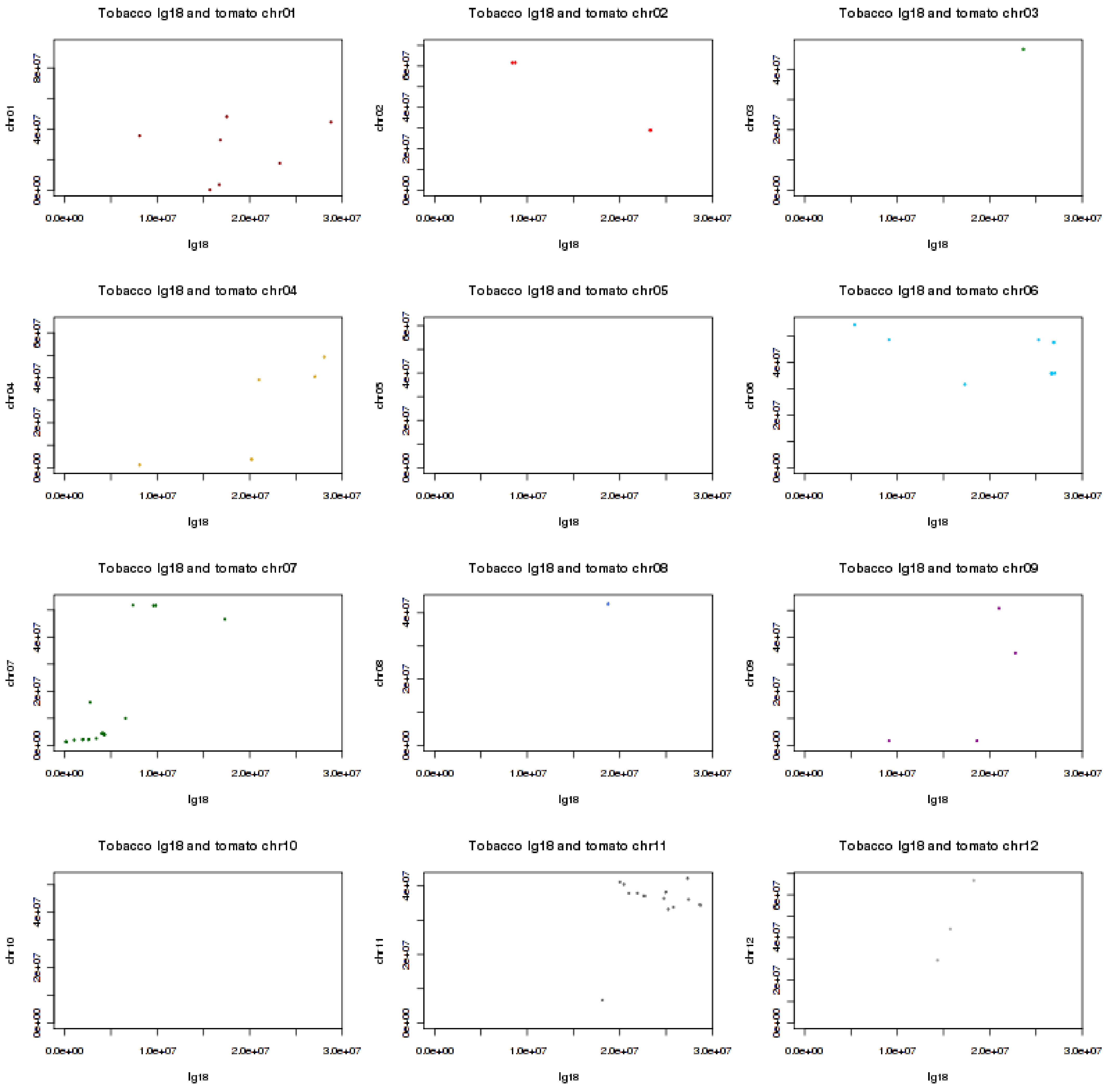
Tobacco lg18 and tomato chr01
Tobacco lg18 and tomato chr02
Tobacco lg18 and tomato chr03
Tobacco lg18 and tomato chr04
Tobacco lg18 and tomato chr05
Tobacco lg18 and tomato chr06
Tobacco lg18 and tomato chr07
Tobacco lg18 and tomato chr08
Tobacco lg18 and tomato chr09
Tobacco lg18 and tomato chr10
Tobacco lg18 and tomato chr11
Tobacco lg18 and tomato chr12
chr01
chr02
chr03
chr04
chr05
chr06
chr07
chr08
chr09
chr10
chr11
chr12
lg18
0.0e+00
1.0e+07
2.0e+07
3.0e+07
0e+00
2e+07
4e+07
6e+07
8e+07

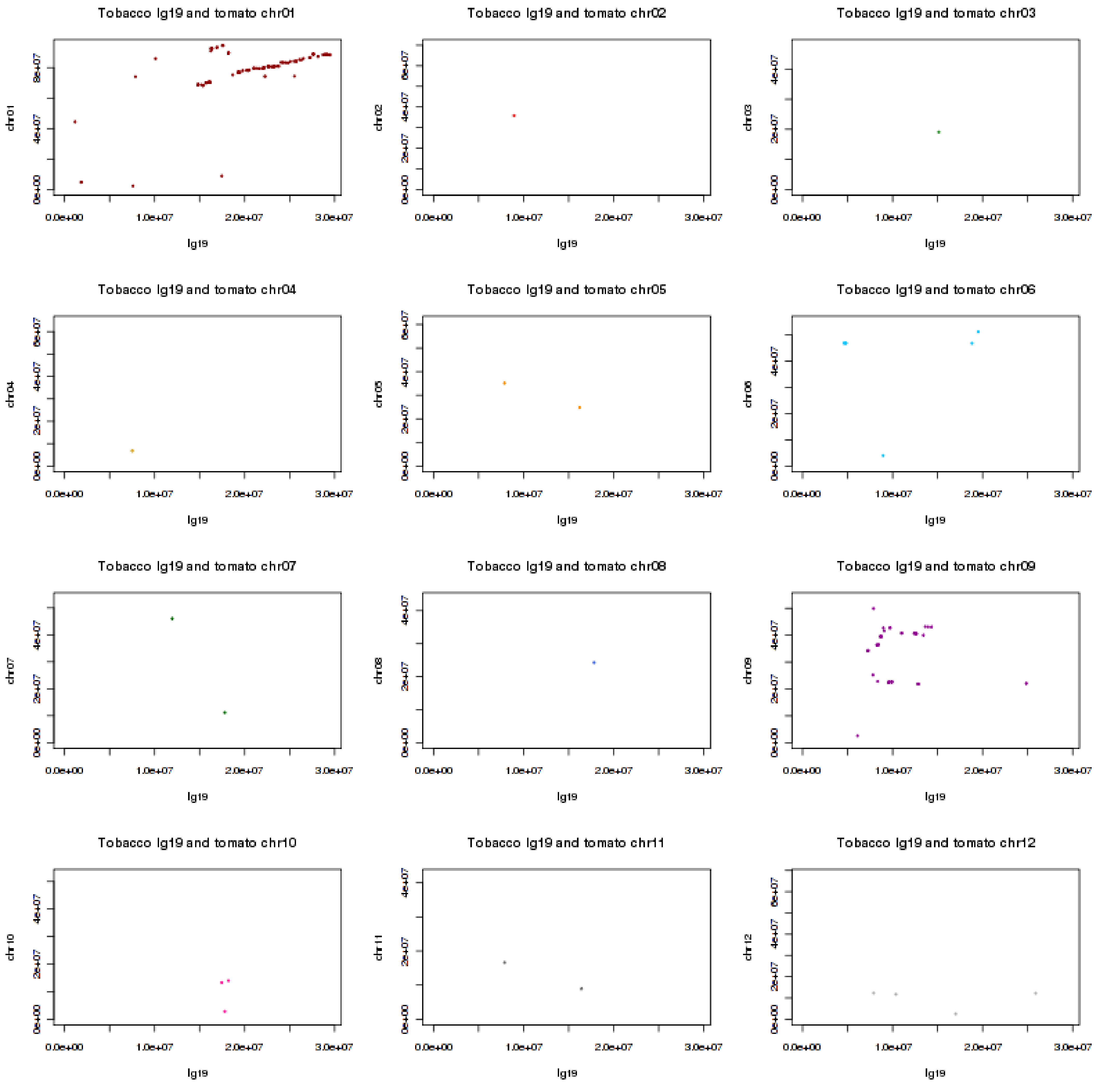

Tobacco lg19 and tomato chr01
chr01
0e+00
4e+07
8e+07
lg19
0.0e+00
1.0e+07
2.0e+07
3.0e+07
Tobacco lg19 and tomato chr02
chr02
0e+00
2e+07
4e+07
6e+07
Tobacco lg19 and tomato chr03
chr03
0e+00
2e+07
4e+07
Tobacco lg19 and tomato chr04
chr04
Tobacco lg19 and tomato chr05
chr05
Tobacco lg19 and tomato chr06
chr06
Tobacco lg19 and tomato chr07
chr07
Tobacco lg19 and tomato chr08
chr08
Tobacco lg19 and tomato chr09
chr09
Tobacco lg19 and tomato chr10
chr10
Tobacco lg19 and tomato chr11
chr11
Tobacco lg19 and tomato chr12
chr12

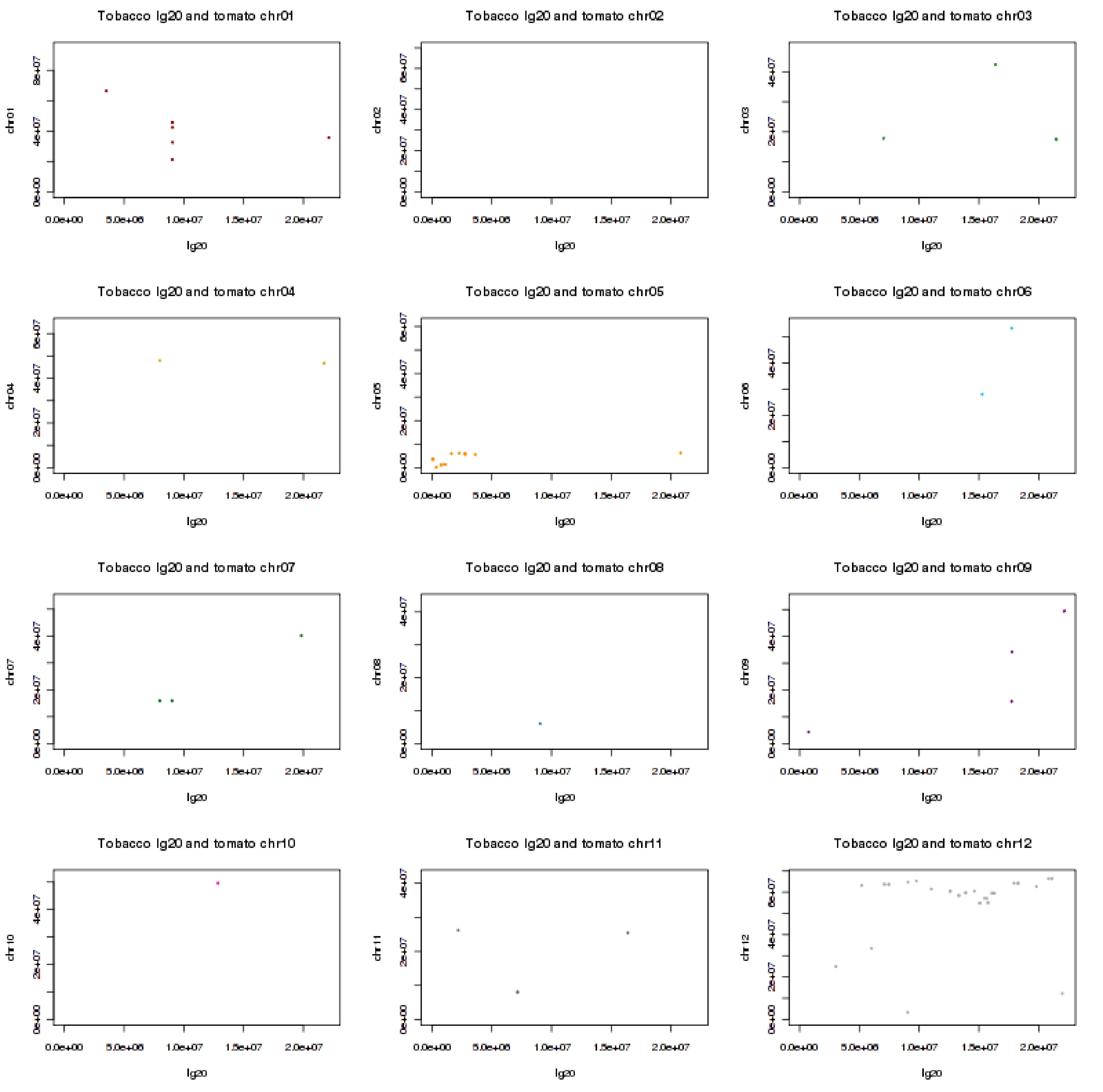
Tobacco lg20 and tomato chr01
chr01
lg20
Tobacco lg20 and tomato chr02
chr02
lg20
Tobacco lg20 and tomato chr03
chr03
lg20
Tobacco lg20 and tomato chr04
chr04
lg20
Tobacco lg20 and tomato chr05
chr05
lg20
Tobacco lg20 and tomato chr06
chr06
lg20
Tobacco lg20 and tomato chr07
chr07
lg20
Tobacco lg20 and tomato chr08
chr08
lg20
Tobacco lg20 and tomato chr09
chr09
lg20
Tobacco lg20 and tomato chr10
chr10
lg20
Tobacco lg20 and tomato chr11
chr11
lg20
Tobacco lg20 and tomato chr12
chr12
lg20
0.0e+00
5.0e+06
1.0e+07
1.5e+07
2.0e+07

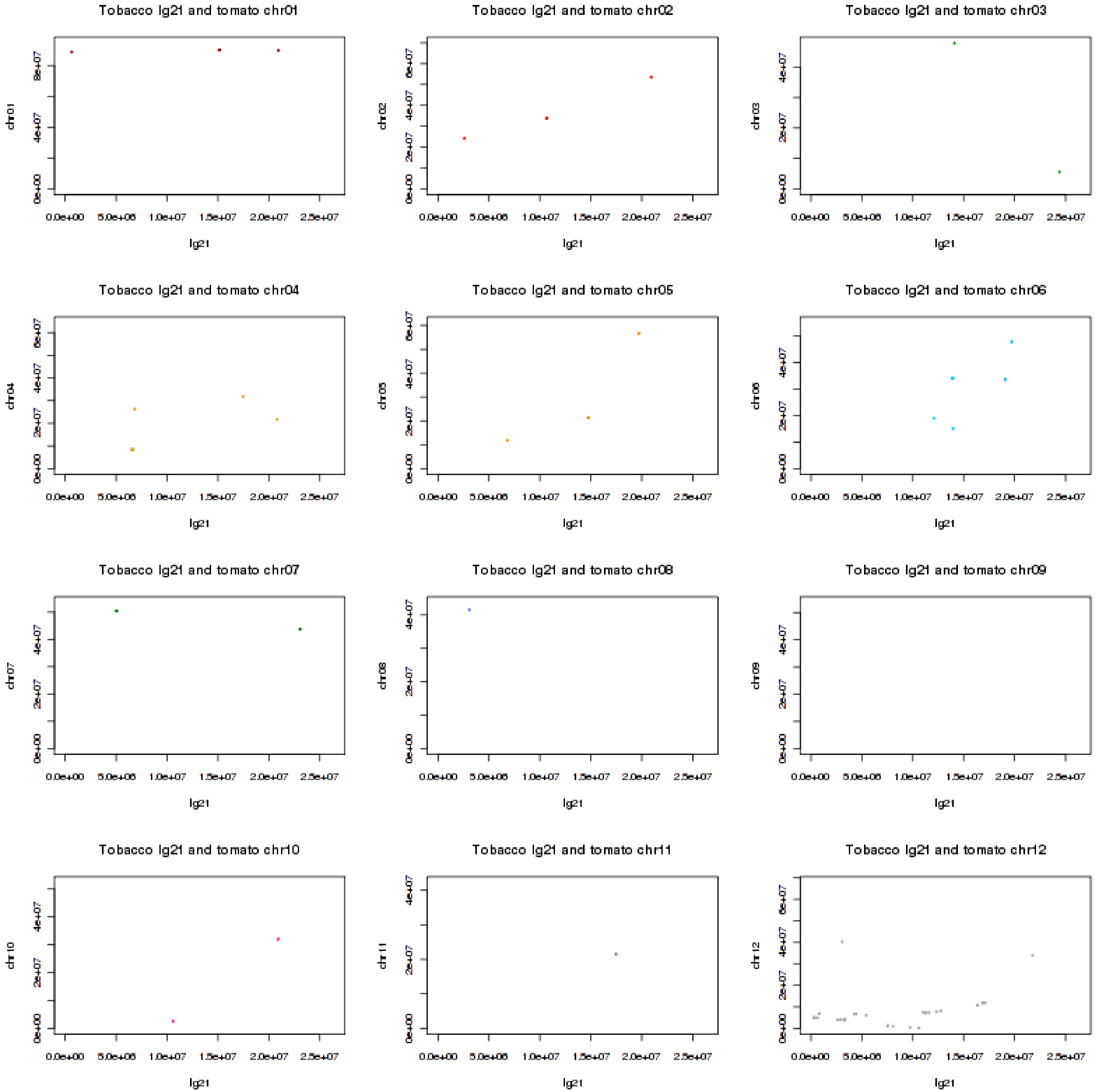
Tobacco lg21 and tomato chr01
chr01
lg21
Tobacco lg21 and tomato chr02
chr02
lg21
Tobacco lg21 and tomato chr03
chr03
lg21
Tobacco lg21 and tomato chr04
chr04
lg21
Tobacco lg21 and tomato chr05
chr05
lg21
Tobacco lg21 and tomato chr06
chr06
lg21
Tobacco lg21 and tomato chr07
chr07
lg21
Tobacco lg21 and tomato chr08
chr08
lg21
Tobacco lg21 and tomato chr09
chr09
lg21
Tobacco lg21 and tomato chr10
chr10
lg21
Tobacco lg21 and tomato chr11
chr11
lg21
Tobacco lg21 and tomato chr12
chr12
lg21
0.0e+00
5.0e+06
1.0e+07
1.5e+07
2.0e+07
2.5e+07

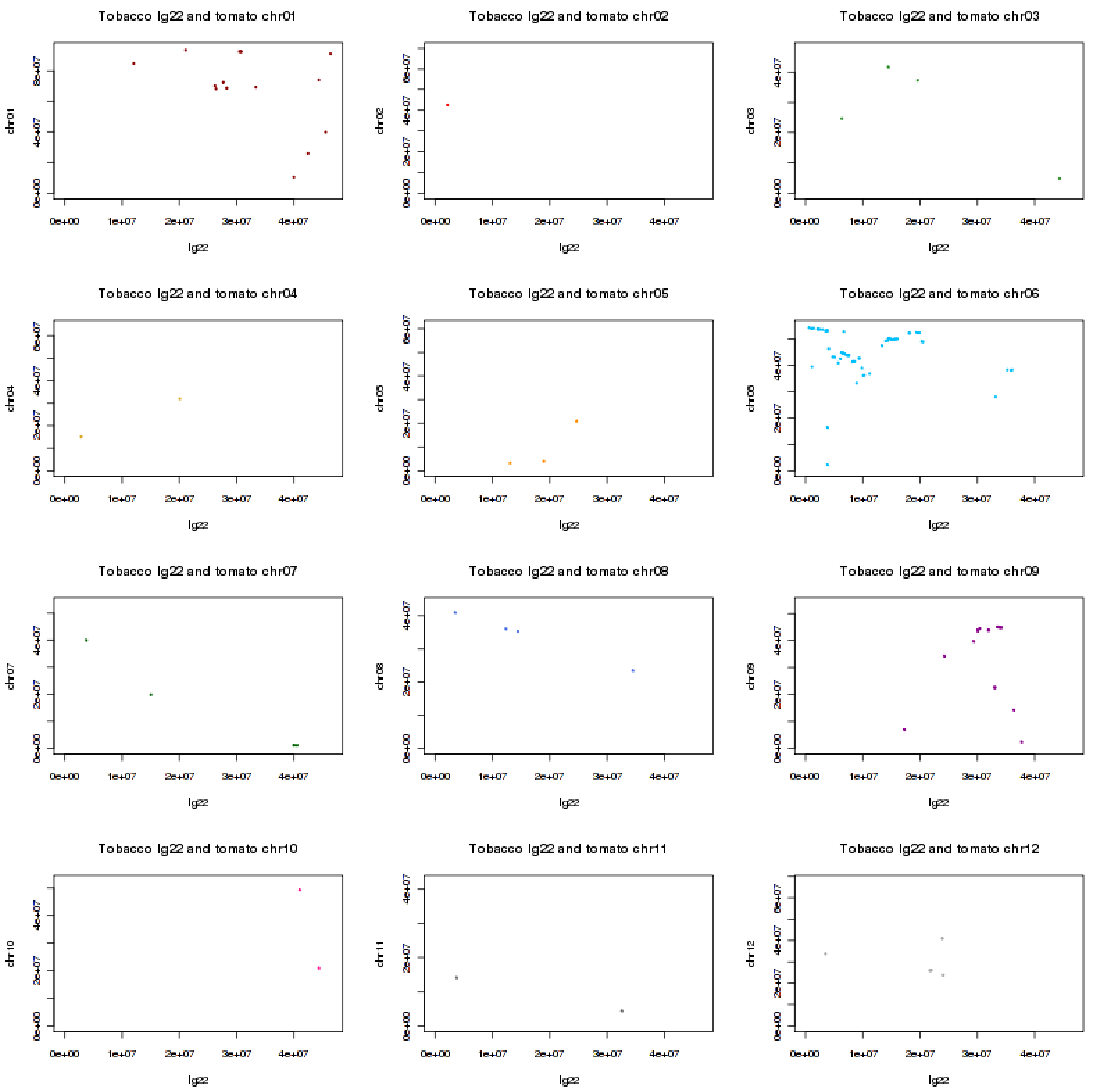
Tobacco lg22 and tomato chr01
chr01
lg22
Tobacco lg22 and tomato chr02
chr02
lg22
Tobacco lg22 and tomato chr03
chr03
lg22
Tobacco lg22 and tomato chr04
chr04
lg22
Tobacco lg22 and tomato chr05
chr05
lg22
Tobacco lg22 and tomato chr06
chr06
lg22
Tobacco lg22 and tomato chr07
chr07
lg22
Tobacco lg22 and tomato chr08
chr08
lg22
Tobacco lg22 and tomato chr09
chr09
lg22
Tobacco lg22 and tomato chr10
chr10
lg22
Tobacco lg22 and tomato chr11
chr11
lg22
Tobacco lg22 and tomato chr12
chr12
lg22
0e+00
1e+07
2e+07
3e+07
4e+07

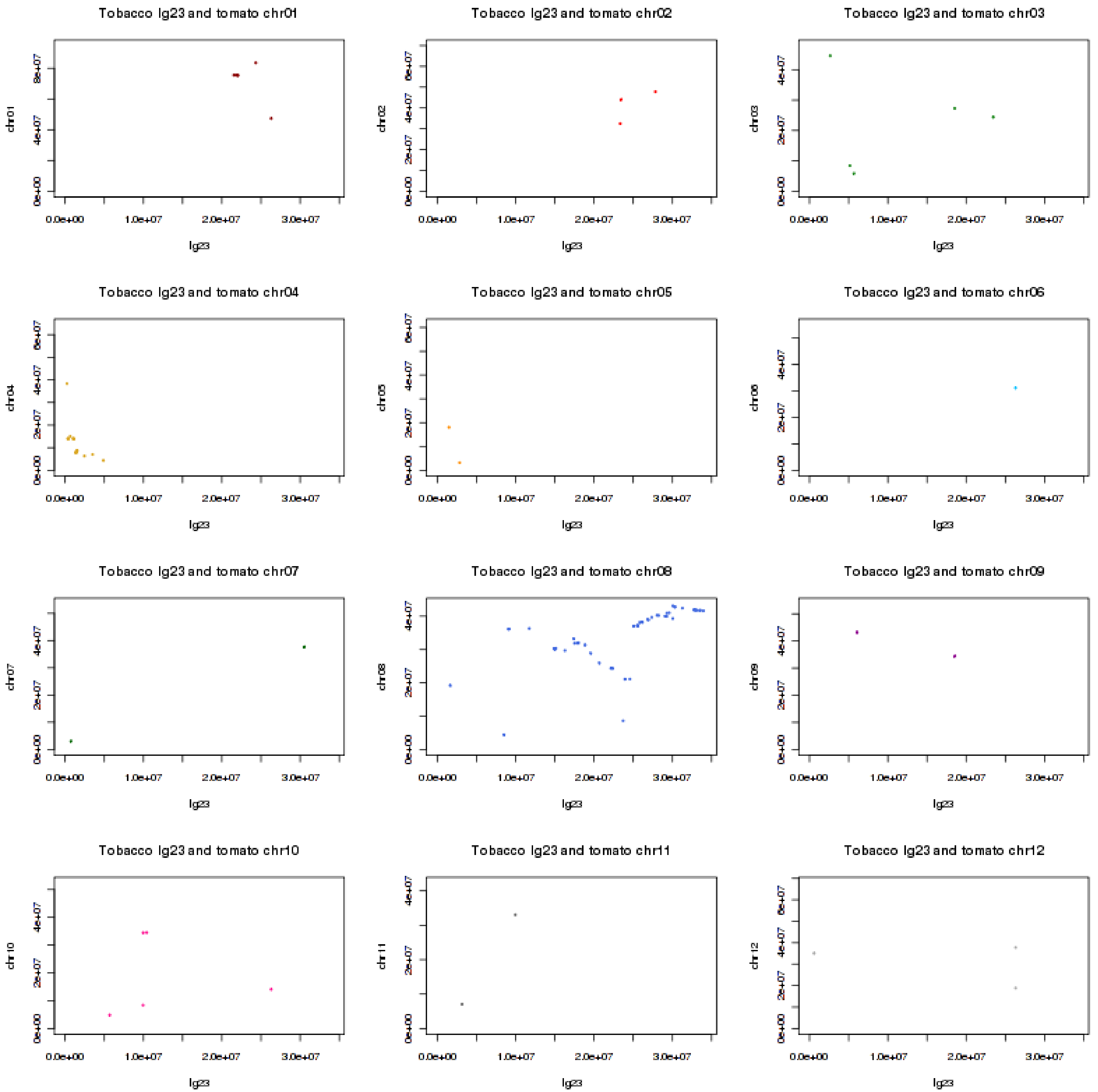
Tobacco lg23 and tomato chr01
chr01
lg23
Tobacco lg23 and tomato chr02
chr02
lg23
Tobacco lg23 and tomato chr03
chr03
lg23
Tobacco lg23 and tomato chr04
chr04
lg23
Tobacco lg23 and tomato chr05
chr05
lg23
Tobacco lg23 and tomato chr06
chr06
lg23
Tobacco lg23 and tomato chr07
chr07
lg23
Tobacco lg23 and tomato chr08
chr08
lg23
Tobacco lg23 and tomato chr09
chr09
lg23
Tobacco lg23 and tomato chr10
chr10
lg23
Tobacco lg23 and tomato chr11
chr11
lg23
Tobacco lg23 and tomato chr12
chr12
lg23
0.0e+00
1.0e+07
2.0e+07
3.0e+07

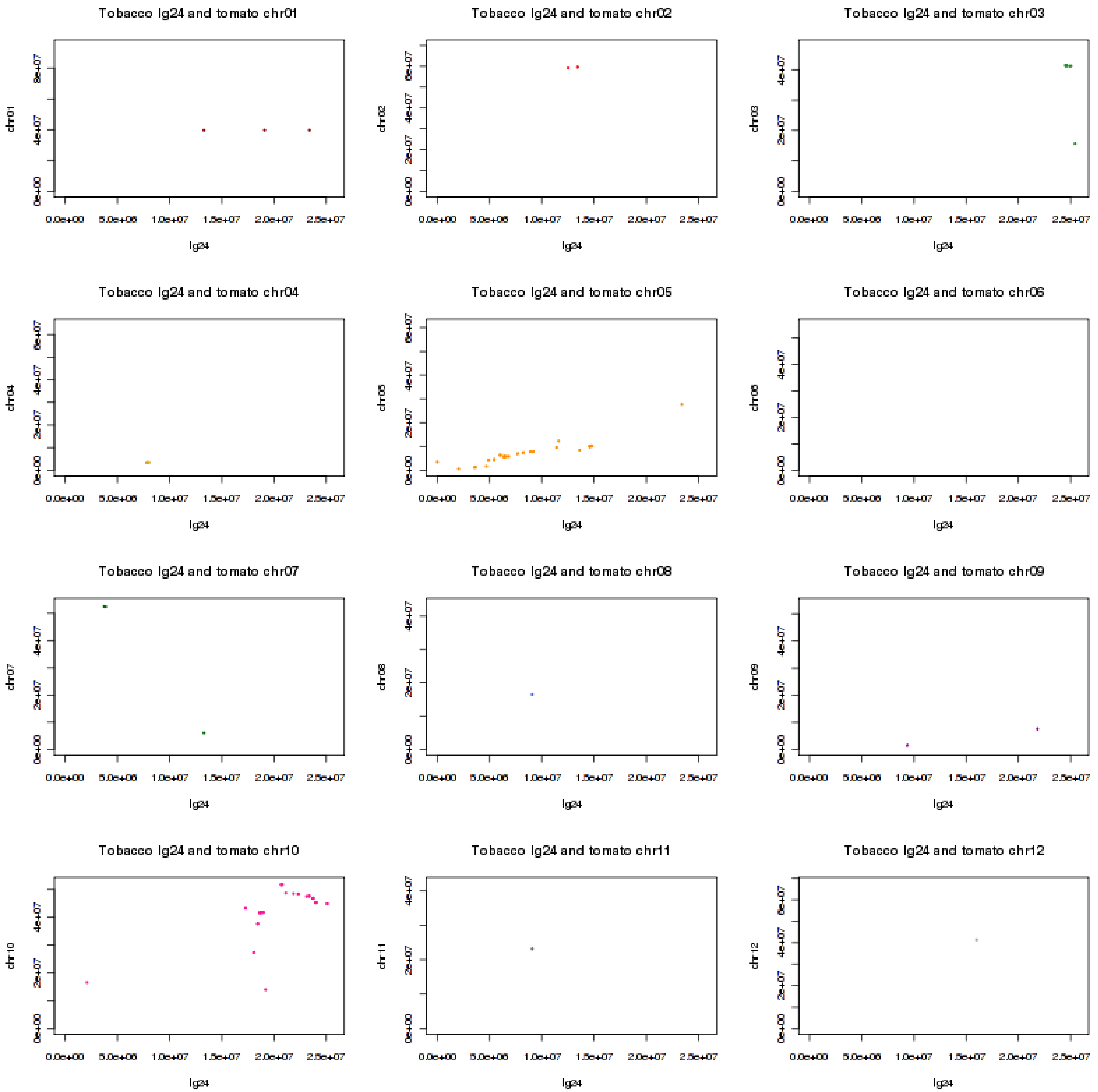
Tobacco lg24 and tomato chr01
Tobacco lg24 and tomato chr02
Tobacco lg24 and tomato chr03
Tobacco lg24 and tomato chr04
Tobacco lg24 and tomato chr05
Tobacco lg24 and tomato chr06
Tobacco lg24 and tomato chr07
Tobacco lg24 and tomato chr08
Tobacco lg24 and tomato chr09
Tobacco lg24 and tomato chr10
Tobacco lg24 and tomato chr11
Tobacco lg24 and tomato chr12
lg24
chr01
chr02
chr03
chr04
chr05
chr06
chr07
chr08
chr09
chr10
chr11
chr12
0.0e+00
5.0e+06
1.0e+07
1.5e+07
2.0e+07
2.5e+07
0e+00
2e+07
4e+07
6e+07
8e+07
